# Mechanism of copper-free Sonogashira reaction operates through palladium-palladium transmetallation

**DOI:** 10.1038/s41467-018-07081-5

**Published:** 2018-11-16

**Authors:** Martin Gazvoda, Miha Virant, Balazs Pinter, Janez Košmrlj

**Affiliations:** 1 Faculty of Chemistry and Chemical Technology, University of Ljubljana, Večna pot 113, SI-1000 Ljubljana, Slovenia; 2 Departamento de Química, Universidad Técnico Federico Santa María, Av. España 1680, 2390123 Valparaíso, Chile; 3 Eenheid Algemene Chemie, Vrije Universiteit Brussel, Pleinlaan 2, B-1050 Brussels, Belgium

## Abstract

The seminal contributions by Sonogashira, Cassar and Heck in mid 1970s on Pd/Cu- and Pd-catalysed (copper-free) coupling of acetylenes with aryl or vinyl halides have evolved in myriad applications. Despite the enormous success both in academia and in industry, however, critical mechanistic questions of this cross-coupling process remain unresolved. In this study, experimental evidence and computational support is provided for the mechanism of copper-free Sonogashira cross-coupling reaction. In contrast to the consensus monometallic mechanism, the revealed pathway proceeds through a tandem Pd/Pd cycle linked via a multistep transmetallation process. This cycle is virtually identical to the Pd/Cu tandem mechanism of copper co-catalysed Sonogashira cross-couplings, but the role of Cu^I^ is played by a set of Pd^II^ species. Phosphine dissociation from the square-planar reactants to form transient three-coordinate Pd species initiates transmetallation and represents the rate-determining step of the process.

## Abstract (web summary)

Although the Sonogashira coupling of acetylenes and vinyl/aryl halides has been applied for decades, many mechanistic questions have remained unanswered. Here, the authors provide experimental and theoretical support for two interlinked palladium cycles operating in the debated Sonogashira coupling mechanism.

## Introduction

**Fig. 1:**
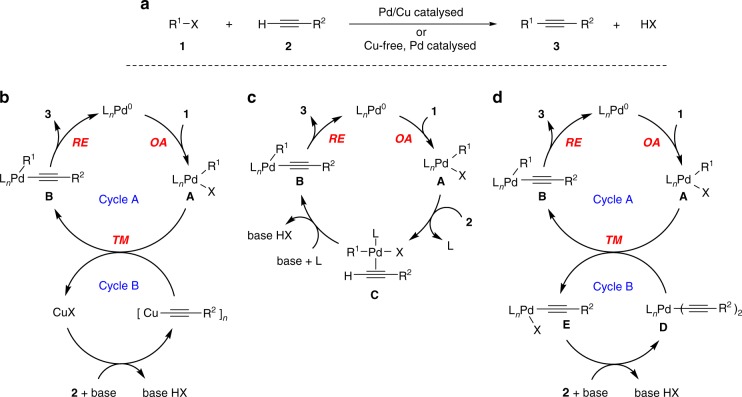
The Sonogashira reaction. **a** General representation of Pd/Cu catalysed and Cu-free Sonogashira reaction. **b** Textbook mechanism for the Pd/Cu catalysed Sonogashira cross-coupling reaction that is synergistically catalysed by Pd and Cu. **c** Textbook mechanism for Cu-free Sonogashira reaction. **d** Our mechanistic proposal for Cu-free Sonogashira reaction. OA oxidative addition, TM transmetallation, RE reductive elimination (*cis*–*trans* isomerization steps are omitted for clarity)

Over recent decades, palladium-catalysed cross-coupling reactions have gained an enormous power in the art of synthetic organic chemistry by providing a fundamental tool for the formation of a carbon–carbon bond in many relevant academic and industrial applications^1–5^. In the array of cross-couplings, the reaction between aryl or vinyl halides and terminal alkynes has become the most general, reliable, and effective method to prepare substituted alkynes (Fig. 1a)^1,4,6–14^. It is known as the Sonogashira reaction—less often, as the Sonogashira–Hagihara reaction. Industrial applications of the Sonogashira reaction are well documented^4,9^. There are two main characteristically distinct protocols for Pd-catalysed alkynylations differing profoundly in the use of co-catalysts. The original Sonogashira reaction requires a copper(I) salt as a co-catalyst in combination with the palladium source. Although beneficial for the effectiveness, the usage of copper as a co-catalyst in Pd/Cu catalysed Sonogashira reaction entails several drawbacks including the application of environmentally unfriendly reagents, the formation of undesirable alkyne homocoupling side products, and the necessity of strict oxygen exclusion in the reaction mixture^8^. Efforts to overcome these unsought circumstances have led to amazing developments in the field of Cu-free Sonogashira reaction, also known as the Heck–Cassar coupling or Heck alkynylation.

According to the consensus mechanism depicted in Fig. 1b, the Pd/Cu catalysed Sonogashira reaction comprises oxidative addition, transmetallation and reductive elimination, and proceeds along two synergistically operating catalytic cycles^15^. In Cycle A, the Pd^0^ species undergoes oxidative addition of the C(*sp*^2^)–X (X = halide) bond of aryl or vinyl halide to provide Pd^II^ complex **A**. Ligand X is then replaced by the acetylene group of a copper acetylide reagent in the transmetallation step to generate σ-alkynylpalladium(II) species **B**. The copper acetylide reagent is produced from the acetylene substrate in the second reaction sequence shown in Cycle B. Finally, species **B** undergoes reductive elimination releasing acetylene derivative and regenerating the starting Pd^0^ species. Although some specifics of the transmetallation step and Cycle B are not fully established, the mechanism of the Pd/Cu catalysed Sonogashira reaction from Fig. 1b is generally accepted in the chemical community^1,2,6,8,10,13,14,16–18^. With some modifications, the oxidative addition–transmetallation–reductive elimination cycle is common to other palladium-catalysed cross-couplings, such as the Suzuki–Miyaura, Stille–Migita–Kosugi, Negishi, Kumada–Tamao–Corriu, and Hiyama–Denmark reactions, where the auxiliary metal, i.e. boron, tin, zinc, magnesium, and silicon, respectively, is essential to assist the transmetallation^1,16^.

Although the first report on the Cu-free Sonogashira reaction dates more than 4 decades ago^19,20^, its mechanism remains elusive. It was tentatively proposed by the group of Soheili in 2003 to consist of the oxidative addition and the reductive elimination steps, as depicted in Fig. 1c^21^. It has been argued that the Cu-free variant cannot build on a transmetallation process. Instead, the formation of **B** was proposed to take place through a reversible π-coordination of the alkyne reagent to complex **A** into η^2^-alkyne–palladium intermediate **C** and subsequent base mediated deprotonation of the terminal acetylenic proton. Although great deal of experimental and theoretical effort has been undertaken in support of this mechanism^22–31^, numerous questions are still open and the proposed model remains unconfirmed. Adversely, recent thorough computational investigations revealed a relatively high activation barrier for the formation of π-complex **C** from the acetylene and the oxidative adduct **A**, for example, refs. ^26,27,31^.

In contrast to the currently accepted mechanism, we hypothesize that the Cu-free Sonogashira reaction proceeds through a tandem Pd/Pd double-cycle shown in Fig. 1d. This pathway is practically identical to the Pd/Cu catalysed mechanism from Fig. 1b, but the role of the copper co-catalyst is taken by a Pd complex. This concept stems from our recent endeavour in the field^32^. The experimental evidence and computational investigation presented in this study convincingly support the operation of a general tandem Pd/Pd cycle in the coupling of aryl halides and terminal alkynes under various conditions.

## Results

### Model reactions and conditions

**Fig. 2:**
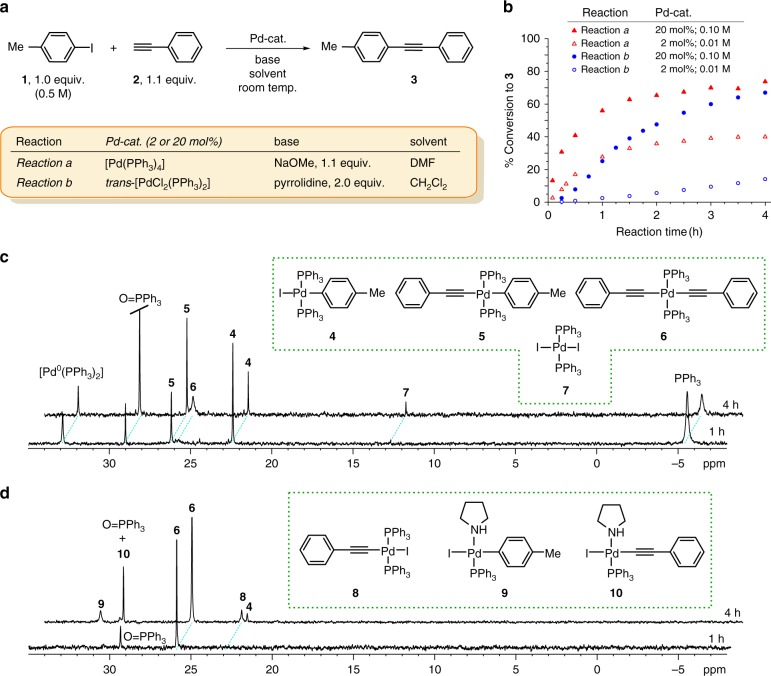
Model reactions and conditions with the proposed phosphorous containing species. **a**, **b** Reaction conditions (**a**) and product **3** build-up as determined by ^1^H NMR spectroscopy (**b**). **c** ^31^P NMR spectra of aliquots from *Reaction a* (with 20 mol% of Pd-cat.), after 1 and 4 h. **d** ^31^P NMR spectra of aliquots from *Reaction b* (with 20 mol% of Pd-cat.), after 1 and 4 h. Relative intensities of the resonances do not correspond to the concentrations of the species. For spectra showing more frequent sampling, see Supplementary Figs. 1–8

To genuinely map out the pathway of the Cu-free Sonogashira mechanism, it is essential to identify pertinent model reactions and conditions. For the experimental analysis, we tentatively selected 4-iodotoluene (**1**) and phenylacetylene (**2**) as archetypal substrates, and triphenylphosphine-based palladium pre-catalysts. Triphenylphosphine, along with other bulky phosphines, is a widely used ligand, with [Pd^0^(PPh_3_)_4_] and *trans*-[Pd^II^(PPh_3_)_2_Cl_2_] being the most common catalyst precursors for the Sonogashira reaction^1^. It is well documented that the choice of the catalysts precursor is highly specific to the selection of base, solvent, and reaction temperature, prompting us to consider two discrete reaction conditions shown in Fig. 2a, which were taken directly from literature. *Reaction a* employs [Pd^0^(PPh_3_)_4_] as a Pd^0^ pre-catalyst, *N*,*N*-dimethylformamide (DMF) as a polar solvent and sodium methoxide as a base^19^, complementary, for *Reaction b* we selected *trans*-[Pd^II^Cl_2_(PPh_3_)_2_] as a Pd^II^ pre-catalyst with pyrrolidine base in apolar dichloromethane^27^. Both reactions were run at 2 mol% (0.01 M) and 20 mol% (0.1 M) loadings of the palladium catalyst at room temperature, and where applicable, the results in terms of tolan formation were consistent with the literature reports^27^. The phosphine-containing pre-catalyst made ^31^P NMR spectroscopy a sensitive and effective probe for monitoring the reactions under this study; however, satisfactory quality of the spectra was obtained only at higher 20 mol% loadings (Supplementary Note 1 and Supplementary Figs. 1–8). Combined with other NMR spectroscopic and mass-spectrometric techniques, as well as independent preparation of intermediates, our synergic analysis enabled the unambiguous identification of all detected species (Supplementary Table 1, Supplementary Note 2 and Supplementary Figs. 9–14).

Analyses of *Reaction a* after 1 and 4 h by ^31^P NMR spectroscopy (20 mol% catalyst loading, Fig. 2c) revealed the presence of the following phosphorous containing species: *trans*-[Pd^II^I(*p*-tolyl)(PPh_3_)_2_] (**4**), *trans*-[Pd^II^(C≡CPh)(*p*-tolyl)(PPh_3_)_2_] (**5**), *trans*-[Pd^II^(C≡CPh)_2_(PPh_3_)_2_] (**6**), *trans*-[Pd^II^I_2_(PPh_3_)_2_] (**7**), [Pd^0^(PPh_3_)_2_], PPh_3_, and O = PPh_3_ (Supplementary Notes 1–2). Later in the reaction, deceleration is evident from Fig. 2b, which can be explained by the presence of increasing amounts of PPh_3_ liberated from the pre-catalyst (vide infra). A completely different reaction course was realized for *Reaction b*. Early in the reaction (1 h), complex *trans*-[Pd^II^(C≡CPh)_2_(PPh_3_)_2_] (**6**) was detected in the ^31^P NMR spectra as the only relevant phosphorous containing species (Fig. 2d). At longer times (4 h), the complexity of the reaction mixture increased, with resonances for species **4**, **8**, [Pd^II^I(*p*-tolyl)(PPh_3_)(pyrrolidine)] (**9**), and [Pd^II^I(C≡CPh)(PPh_3_)(pyrrolidine)] (**10**) appearing in the spectra. The formation of **9** and **10** was rationalized by a PPh_3_ ligand exchange to pyrrolidine in **4** and **8**, respectively, as confirmed by independent experiments (Supplementary Figs. 13–14), and consistent with the literature data for related Pd-species^23,33^. In both, *Reaction a* and *Reaction b*, after 4 h homocoupled acetylene dimer (Glaser–Hay product) was detected in negligible amounts (<5% by ^1^H NMR).

**Fig. 3:**
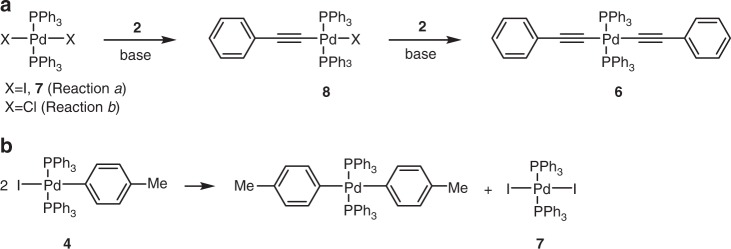
Formation of some key catalytic intermediates. **a** Formation of acetylides **6** and **8**. **b** Homocoupling of **4** to give *trans*-[Pd^II^I_2_(PPh_3_)_2_] (**7**)

In view of the mechanistic proposal in Fig. 1d, the above observations could be interpreted as follows: intermediate **4** corresponds to the product of the oxidative addition step (*trans*-**A**), **5** and **8** are the direct transmetallation products (*trans*-**B** and *trans*-**E**, respectively), while complex **6** serves as *trans*-**D** delivering acetylene to **4** in the transmetallation step. To close the Cycle B in Fig. 1d, the bisalkynylpalladium complex **6** (*trans*-**D**) is regenerated in a base mediated reaction between **8** (*trans*-**E**) and **2**. Indeed, the high propensity of **8** to combine with **2** into **6** in the presence of a base (Fig. 3a) was confirmed by an independent experimental work (Supplementary Note 2).

The analysed reaction mixtures recorded for *Reaction a* and *Reaction b* in Fig. 2, reveal characteristically different courses of reactions. Namely, in *Reaction a*, the successive dissociation of PPh_3_ ligands from the [Pd^0^(PPh_3_)_4_] pre-catalyst generates the catalytically active Pd^0^ species ([Pd^0^(PPh_3_)_2_] or [Pd^0^PPh_3_])^1,34^, which undergoes oxidative addition with **1** to form **4** (*trans*-**A**). Along with **4** (*trans*-**A**), bisalkynylpalladium **6** (*trans*-**D**) is essential for the tandem catalytic cycle to operate. The latter complex is formed via homocoupling of **4** to give *trans*-[Pd^II^I_2_(PPh_3_)_2_] (**7**) (Fig. 3b), which subsequently combines with acetylene **2** via the intermediacy of **8** (Fig. 3a, X = I). The ability of **4** to undergo homocoupling has been confirmed independently (Supplementary Note 4 and Supplementary Fig. 24). Due to the high reactivity of **8** towards acetylene **2** to form **6** we could not detect this species, unlike **7**, in the reaction mixture of *Reaction a* (Fig. 2c). The fact that *trans*-**5** (*trans*-**B**) could only be detected in *Reaction a* indicates its relatively slow reductive elimination into **3** that can be well explained by the presence of excess PPh_3_, which has a decelerating effect, as previously noticed by Stille et al.^35^ and discussed below.

In contrast to *Reaction a*, combining acetylene **2** and *trans*-[Pd^II^Cl_2_(PPh_3_)_2_] reagents in *Reaction b* initially results in the accumulation of complex **6** (*trans*-**D**). Its partial reductive elimination, likely through the intermediacy of *cis*-**6**, affords the butadiyne by-product (PhC≡C)_2_, and the active Pd^0^ species, e.g. [Pd^0^(PPh_3_)_2_]^36^. The latter, undergoing oxidative addition with **1**, initiates Cycle A in Fig. 1d. With the progress of *Reaction b*, sufficient amounts of **4**, **8**, **9** and **10** are accumulated in the reaction mixture to be detected by NMR (Fig. 2d). In summary, the detected species in both the reaction mixtures of *Reaction a* and *Reaction b* are fully consistent with the hypothesized tandem Pd/Pd mechanism.

### Transmetallation

**Fig. 4:**
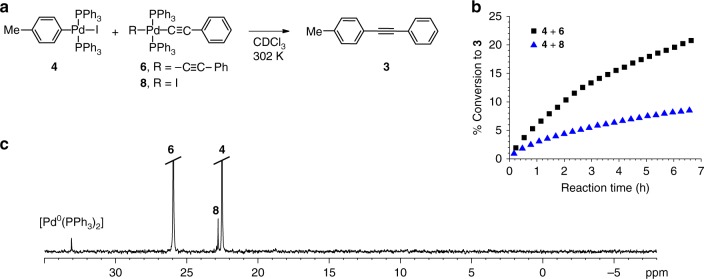
Probing transmetallation. **a** Reaction between **4** (1.0 equiv., 0.013 M) and acetylide **8** or **6** (1.2 equiv., 0.016 M). **b** Tolan **3** build-up. **c** ^31^P NMR analysis of the reaction mixture between **4** and **6** incubated for 90 min (Supplementary Note 3 and Supplementary Figs. 15–16)

The key step of the proposed mechanism is transmetallation between two palladium species **A** and **D** interconnecting the two catalytic Pd-cycles from Fig. 1d. To demonstrate the feasibility of such a transformation, we let independently prepared **4** and **6** to react as shown in Fig. 4a. As pointed out by Amatore and Jutand, reactions performed on isolated putative catalytic cycle segments may proceed quite differently to those under the catalytic conditions^37^. It is thus important to note that the composition of the reaction mixture from Fig. 4c is highly reminiscent to that of *Reaction a* and *Reaction b* (Fig. 2) confirming the presence of key intermediates from our mechanistic proposal shown in Fig. 1d. Accordingly, complex **8** (*trans*-**E**) is the result of transmetallation between **4** (*trans*-**A**) and **6** (*trans*-**D**), while [Pd^0^(PPh_3_)_2_] (Fig. 4c) originates from the reductive elimination of **5** (**B**). Along with **6**, monoalkynylpalladium **8** should also be considered as a potential alkynyl carrier in the transmetallation with **4**. Indeed, we have confirmed that **4** and **8** also yield tolan **3**, albeit in a more sluggish process (Fig. 4b). Although the progress of the reactions shown in Figs. 2 and 4 (both 0.01 M in Pd) appear similar, a more precise comparison is not in place due to the apparently different reaction conditions. Nevertheless, the progress of transmetallation reaction of **4** and **6** is in the same range as in *Reaction a* and *Reaction b* with a similar concentration of Pd-cat. (0.01 M), and is comparable with the literature data^27^. The formation of homocoupled acetylene dimer (Glaser–Hay product) could not be detected by ^1^H NMR.

**Fig. 5:**
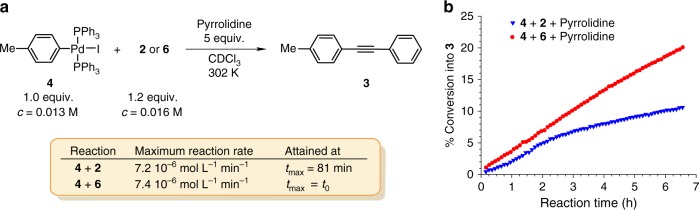
Putting on trial the mono-metallic mechanism from Fig. 1c. **a** Independent reaction between authentic **4** and **6** or acetylene **2** in the presence of pyrrolidine. **b** Tolan **3** build-up (Supplementary Note 3 and Supplementary Figs. 17–22)

An independent reaction between the authentic oxidative addition product **4** (*trans*-**A**) and acetylene **2** in the presence of excess base (Fig. 5a) should mimic, at least at the onset, the segment from the mono-metallic proposal from Fig. 1c where the acetylene π-coordination into **C** is followed by a base assisted deprotonation into **B**. As a result, however, slow progress towards tolan **3** with an apparent induction period was noticed (Fig. 5b). The induction period in the reaction of **2** with **4** is fully consistent with a lack of another species in the reaction medium that is of key importance to the catalytic cycle to operate, i.e. Pd-acetylide **6** (or **8**, vide supra). This can arise by partial decomposition of **4** through the reaction sequences shown in Fig. 3. Progression of these reactions in the induction period, including the transmetallation-reductive elimination events, result in the concentration build-up of **6**, and, accordingly, the reaction is gaining in rate. Should, however, the Cu-free Sonogashira reaction proceed via intermediate **C**, i.e. through the mono-metallic proposal from Fig. 1c, one would expect the kinetic profile with an initial maximum reaction rate that is absent of an induction period.

Under identical reaction conditions, in CDCl_3_ and in the presence of pyrrolidine (Fig. 5), the maximum rate of the reaction of **4** with **2** (7.2 × 10^−6^ mol L^−1^ min^−1^) was compared to that of **4** with **6** (7.4 × 10^−6^ mol L^−1^ min^−1^). In contrast to the latter, where the maximum reaction rate corresponds to the initial rate and is absent of the induction period, the maximum rate in the reaction of **4** with **2** was attained at 81 min. Practically identical maximum rates in these two reactions indicate the transmetallation event in the reaction of **4** with **2**, and, accordingly, in the copper-free Sonogashira reaction.

Finally, the revealed induction period cannot be ignored in *Reaction a* and *Reaction b* (Fig. 2b, Supplementary Note 5 and Supplementary Figs. 29–31). As presented above, in *Reaction a* the oxidative addition intermediate that is generated from the catalytically active Pd^0^ species [Pd^0^(PPh_3_)_2_], formed by PPh_3_ dissociation from [Pd^0^(PPh_3_)_4_], and aryl iodide cannot proceed towards tolan **3** in the absence of Pd-acetylide **6**, whereas the induction period in *Reaction b* is due to the build-up of **4**. It should be noted that the appearance of the induction period in the Cu-free Sonogashira reaction strongly depends on the reaction conditions, and can easily remain unnoticed. Nevertheless, it is apparent from some previous mechanistic studies where the reactions were intentionally made sluggish for the monitoring purposes^29^.

An attempt was made to determine the order in palladium by running *Reaction a* at different loadings of Pd(PPh_3_)_4_ (3, 4, and 5 mol%) while monitoring the formation of tolan **3** over time (experimental details are provided in Supplementary Note 5). For each Pd loading, the first derivative of the sigmoid curves that resulted from the experimental data fitting process returned the maximum reaction rates. In a log–log graph, these were plotted against Pd concentrations, returning tentative first-order kinetics in palladium (Supplementary Fig. 32 and Supplementary Table 2). Since the oxidative addition of 4-iodotoluene (**1**) with Pd(PPh_3_)_4_ into **4** is under the investigated conditions nearly instant, as confirmed by independent experiments (Supplementary Fig. 25) one could interpret this result by either reductive elimination from Cycle A or palladium bis-acetylide **6** formation from Cycle B as the potential rate-limiting steps. On the other hand, if the concentration of **4** is always much higher than **6**, which is an acceptable presumption in case of prompt oxidative addition, and consistent with the results shown in Supplementary Fig. 2, then the rate of transmetallation virtually depends on the concentration of **6**. In this case, transmetallation can be well approximated as a pseudo-1st-order kinetics, and hence it should not be excluded from the list of possible rate-determining steps based on our preliminary kinetic study.

### Computational studies

**Fig. 6:**
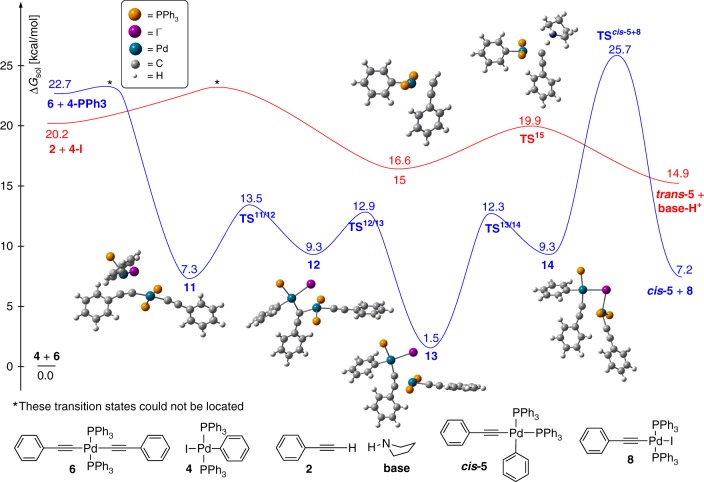
DFT calculations. Computed reaction profile and solution-state Gibbs free energies of stationary points for the transmetallation process, **4** + **6** → ***cis*-5** + **8**, and for the direct activation of phenylacetylene (**2**) by **4** through a monometallic mechanism

To gain a molecular-level insight into the mechanism of the transmetallation event, we computed the most plausible reaction trajectories using the parent models of the experimentally used complexes, *trans*-[Pd^II^(C≡CPh)_2_(PPh_3_)_2_] (**6**) and *trans*-[Pd^II^I(phenyl)(PPh_3_)_2_] (**4**) (Fig. 6 as well as Supplementary Figs. 33–41). The most likely pathway is illustrated in Fig. 6 (blue) together with the direct activation of phenylacetylene by **4** (red). As anticipated in Fig. 6, the onset of transmetallation is phosphine dissociation from **4** to form the three-coordinate Pd^II^I(phenyl)(PPh_3_) (**4-PPh3**) with a relative solution-state free energy of 22.7 kcal mol^−1^. We perceive this step to be the rate-determining step of the process (vide infra). Once **4-PPh3** is formed its rapid association with **6** yields the bi-metallic intermediate **11**, in which the bridging PhC≡C^−^ functionality binds to the Pd centres of **6** with its σ-lone pair (η^1^) whereas to the palladium of **4-PPh3** in η^2^-fashion through the π-system of the C≡C triple bond. The key motive of transmetallation is the transformation of **11** into intermediate **13**, in which the linking acetylide ligand already binds to the **4-PPh3** fragment with its σ-lone pair and to the **6** derived Pd^II^(C≡CPh)(PPh_3_)_2_ fragment with its π-system. This migration of acetylide takes place in two steps via the intermediacy of **12** and traversing transition states **TS**^**11/12**^ and **TS**^**12/13**^. The key structural changes in the latter TSs are the swinging of the migrating PhC≡C^−^ group from one palladium centre to the other. The structure at the midway of this transition, i.e. the quasi-symmetric Pd–C–Pd core, appears as a local minimum (**12**) on the potential energy surface. The key significance of the exposed mechanism is the low energy nature of this central transmetallation process with stable transition states and transient intermediates, which are indeed the features of efficient catalytic processes. We attribute this balanced energy landscape and facile ligand migration to the almost ideal square-planar arrangements around both Pd centres throughout the entire process. The iodide ion that stays in the proximity of both Pd^2+^ centres also massively contributes to the stabilization via electrostatics.

The next step **13** → **TS**^**13/14**^ → **14** initiates the dissociation of the fragments; the η^2^–type PhC≡C^…^Pd bond breaks and this Pd centre shifts towards the iodide and develops an I–Pd interaction in **14**. Our analysis indicates that the direct dissociation of **14** to **8** and three-coordinate ***cis*-5-PPh3** (27 kcal mol^−1^) might be possible under certain circumstances. To form stable square-planar species, however, a coordinating substrate needs to react with **14**; the associative mechanism for PPh_3_ attack to yield ***cis*-5** and **8** is illustrated in Fig. 6, traversing transitions state **TS**^***cis*-5+8**^. Although this dissociation event is associated with a relatively high computed barrier (25.7 kcal mol^−1^), the error associated with conformational diversity^38^ is expectedly in the order of 10–12 kcal mol^−1^ in this transition state rendering this process to be operative at room temperature. Other dissociation routes from **14** might be operational as well, leading to a myriad of transmetallation products and eventually to ***trans-*** and ***cis***-**5** through subsequent ligand exchange processes.

In order to put the transmetallation process into context with the same function of monometallic mechanisms (to deliver **5**), we also scrutinized direct activation of phenylacetylene (**2**) by **4**. This analysis was necessary because we could not directly compare our results to earlier computational findings due to the applied oversimplifications of systems, e.g. using PH_3_ ligands, and unrealistic assumptions, e.g. spontaneous deprotonation of phenylacetylene by pyrrolidine^27^. Similarly to the earlier proposed ionic mechanism, the monometallic pathway shown in Fig. 6 also begins with the dissociation of iodide from **4** to yield **4-I** with solution-state Gibbs free energy of 20.2 kcal mol^−1^. This cationic species might bind phenylacetylene through the π-system of its H–C≡C functionality. Rapid deprotonation by pyrrolidine takes place in the formed intermediate **15** through **TS**^**15**^ (19.9 kcal mol^−1^) to generate **base-H**^**+**^ and ***trans*-5**, which converts to ***cis*-5** via ligand exchange processes before initiating reductive elimination. We revealed a much slower deprotonation and less probable product formation for phosphine dissociation initiated activation of acetylene (Supplementary Note 6 and Supplementary Fig. 34).

The computed activation parameters imply that both mechanisms are plausible under the experimental setup and, accordingly, the Cu-free Sonogashira reaction might proceed via several, often competing mechanisms and certain substrates, solvents, ligands, substituents, bases, and other factors might favour one pathway over another. As we found the alternative direct associative mechanisms to have higher activation energies (Supplementary Fig. 33), than the dissociative pathways, we predict the formation of the three-coordinate species, **4-PPh3** and **4-I**, through ligand dissociation to be the rate-determining step of the corresponding transmetallation and monometallic processes. Since the association of the three-coordinated **4-PPh3** and **4-I** with **6** and **2**, respectively, are considered to be barrierless processes^39^, the stability of the transient intermediates **4-PPh3** (22.7 kcal mol^−1^) and **4-I** (20.2 kcal mol^−1^) determines the activation barriers of the corresponding processes. While density functional theory (DFT) cannot realistically differentiate between the plausibility of the formation of these two species^40^, experimental studies demonstrated phosphine dissociation from square-planar Pd^II^ species^41^ as well as stable three-coordinate Pd(II) species containing only one bulky phosphine group^42,43^. In addition, we found that pyrrolidine preferably substitutes phosphine rather than iodine in **4**, forming complex [Pd^II^(phenyl)(PPh_3_)(pyrrolidine)I] (**9**), which is in complete agreement with the literature data^23,33^ (Supplementary Fig. 13). The accessibility of three-coordinated T-shaped Pd^II^ intermediates, like **4-PPh3**, has been assessed computationally^44^ and such species have already been considered explicitly in mechanistic studies^45^. The extrusion of bulky phosphines is mostly driven to ease steric strain leading to such short-lived three-coordinate palladium centres, which, implied by our findings, might play a hitherto unrevealed, critical role in other transformations as well. In conclusion, these findings support that phosphine dissociation initiated transmetallation is plausible under standard experimental conditions confirming the prediction of Lledós and Espinet and co-workers that ‘This [bulky groups] will eventually switch the substitution and transmetallation steps of the catalytic cycles (for instance cross-coupling reactions) from the classical associative pathways to dissociative mechanisms’^44^.

In line with the above-perceived reaction trajectories for the transmetallation, previous experimental and computational reports established alkynyl ligand transfer from several other metals including Cu, Ag, Au, and Pt to arylpalladium intermediates^46–56^. The migration of the acetylide was found to be reversible, passing through bimetallic intermediates/transition states with Pd coordinated to the π-bond of the metal-acetylide. Reversible alkynyl ligand transfer between two Pd^II^ complexes, *trans*-[Pd^II^(C≡CPh)(*p*-tolyl)(PEt_3_)_2_] (PEt_3_ analogue of **5**) and *trans*-[Pd^II^I(*p*-MeO-C_6_H_4_)(PEt_3_)_2_] (close analogue of **4**), to give *trans*-[Pd^II^I(*p*-tolyl)(PEt_3_)_2_] (PEt_3_ analogue of **4**) and *trans*-[Pd^II^(C≡CPh)(*p*-MeO-C_6_H_4_)(PEt_3_)_2_] (close analogue of **5**) has been observed by Osakada and Yamamoto, but reductive elimination has not been reported^57,58^, and this process has never been put into the context of the copper-free Sonogashira reaction. Importantly, in alkynyl ligand transfer between an alkynylcopper and *trans*-[Pd^II^I(aryl)(PEt_3_)_2_] species, upon addition of excess PPh_3_ to the reaction mixture the authors observed a decelerating effect and formation of *trans*-[Pd^II^(C≡CPh)(aryl)(PEt_3_)_2_] (close analogue of *trans*-**5**). This was not observed in the absence of the coordinative ligand. Taking into account these results^57^, those by Stille et al.^35^, Espinet et al.^59,60^, as well as our experimental evidence, there is a dynamic, PPh_3_-mediated equilibrium process between *cis*-**5**/*trans*-**5** isomers with the latter being the resting state, as also predicted by DFT (***trans-*5** is more stable than ***cis-*5** by 2.0 kcal mol^−1^).

## Discussion

We provided a detailed experimental and computational scrutiny for plausible transmetallation in the copper-free Sonogashira reaction, i.e. in the palladium-catalysed cross-coupling of acetylenes with aryl halides. In addition, in contrast to the earlier proposed mono-metallic mechanism, our systematic experiments intuitively revealed a tandem Pd/Pd catalytic cycle, analogous to the tandem Pd/Cu mechanism of the copper co-catalysed Sonogashira reaction. Although alternative mechanisms are plausible, our experimental results imply that the transmetallation-centred tandem Pd/Pd mechanism holds true under distinct characteristic conditions of the copper-free variant of the Sonogashira cross-coupling. Solution-state DFT simulations are in accord with these notions revealing a low-energy pathway for acetylide migration through a multi-step transmetallation process. Dissociation of the bulky phosphine ligand, PPh_3_, from the Pd^II^I(phenyl)(PPh_3_)_2_ reactant to form the three-coordinate active species and initiate transmetallation is predicted to be the rate-determining slow process of the investigated transmetallation event. Our computational analysis based on the full models of experimental species also puts forward a plausible, potentially competing monometallic pathway for the direct activation of phenylacetylene, also initiated by ligand dissociation. The evidence of the bimetallic pathway, which is the first identified palladium–palladium cross-coupling reaction, shall inspire new design principles and new coupling reactions.

## Methods

### General procedure for transmetallation reactions

Oxidative adduct **4** (0.0136 M, 1 equiv.) and the source of acetylene (palladium bis-acetylide **6**, palladium mono-acetylide **8** or acetylene **2**) (0.0163 M, 1.2 equiv.) were let to react in an oven-dried NMR tube in chloroform-*d* (0.70 mL) under argon atmosphere at 302 K for a given time. The reactions were monitored by NMR spectroscopy. The formation of the product **3** over time is presented in Figs. 4 and 5. All reactions were conducted at least in triplicates, always returning consistent results.

### General procedure for *Reaction a* and *Reaction b*

To a stirred mixture of 4-iodotoluene (**1**, 545 mg, 2.5 mmol), phenylacetylene (**2**, 281 mg, 2.75 mmol), and appropriate base (1,3,5-trimethoxybenzene was added as internal standard) in given solvent (5 mL) palladium catalyst (0.50 mmol, 20 mol% or 0.05 mmol, 2 mol% of Pd) was added at room temperature under argon atmosphere. Stirring was continued at room temperature. After given time, an aliquot (50 μL) was directly diluted with CDCl_3_ (0.6 mL), transferred into NMR tube, and ^1^H and ^31^P NMR were recorded immediately. It has been confirmed that this workup completely stops the reaction by re-acquiring the ^1^H NMR spectrum of the same sample after being aged in the NMR tube for 1 h, with the same result. The conversion into product **3** was determined by ^1^H NMR spectroscopy and is shown in Fig. 2b. Both reactions were conducted at least in triplicates, always returning consistent results.

### Computational investigations

All calculations were carried out using DFT as implemented in the Gaussian09 program package^61^. In this in silico study, we used the parent models of the complexes investigated experimentally, meaning that we only replaced the methyl substituent in *p*-tolyl to hydrogen (i.e. *p*-tolyl to phenyl). Final geometry optimizations were performed using the hybrid-meta-GGA TPSSh^62^ functional in combination with the relativistic core potential containing cc-pVDZ-PP basis set for Pd and I^63^ whereas the cc-pVDZ basis set for light atoms^64^. Analytical vibrational frequency calculations were carried out at the same level of theory to confirm that the optimized structures correspond to either minima or first-order saddle points (transition state) of the potential energy surface. Dispersion was taken into account in all calculations, including geometry optimizations, using Grimme’s D3 method^65^ with the original D3 damping function and with SR6 and S8 parameters of 1.660 and 1.105, respectively, originally recommended for TPSS. The energies of the optimized structures were reevaluated using the triple-ζ basis set cc-pVTZ(-PP) (-PP applies for Pd and I)^66^. Solvation energies for DMF were also computed at triple-ζ basis set (TPSSh/cc-pVTZ(-PP)) using the SMD implicit solvation model^67^. We used the Solvent Accessible Surface (SAS) method to create the molecular surface of the solute–solvent boundary where the atomic radii used to generate the solute surface were the followings: H (1.400 Å), P (2.500 Å), C (2.300 Å), I (2.600 Å), and Pd (1.800 Å) while the radius of solvent (DMF) was set to be 1.80 Å. In all of these calculations, an ultrafine grid has been used. As computing precisely the solvation energy of small charged ions, such as I^−^, is challenging^68^, we used a solvation energy of −55.0 kcal mol^−1^ for bare I^−^ in DMF derived from experimental Gibbs free energy of hydration (−59.9 kcal mol^−1^) and Gibbs free energy of transition from water to DMF (+4.9 kcal mol^−1^)^68^.

Detailed synthetic procedures for preparation and characterization of compounds **3**–**10** are provided in Supplementary Note 2, along with the copies of NMR spectra (Supplementary Figs. 42–75) and additional details of computational investigations are provided in Supplementary Note 6 and Supplementary Dataset.

## Electronic supplementary material

Supplementary Information

Supplementary Data 1

## Data Availability

The data that support the findings of this study are available within the article and Supplementary Information files, and are also available from the corresponding authors upon reasonable request.
