# Supplementary material for: Mechanism of copper-free Sonogashira reaction operates through palladium-palladium transmetallation
Source: Nat Commun. 2018 Nov 16;9:4814. doi: 10.1038/s41467-018-07081-5 (PMC6240041; doi:10.1038/s41467-018-07081-5)
Supplement: Supplementary file 1 — Supplementary Information [file 41467_2018_7081_MOESM1_ESM.pdf]

SUPPLEMENTARY INFORMATION

Mechanism of copper-free Sonogashira reaction operates through palladium-palladium transmetallation

Gazvoda et al.

SUPPLEMENTARY METHODS

General information

All reactions were performed in oven-dried glassware under argon atmosphere. All reagents were used as purchased without further purification unless otherwise noted.

^{1}H, ^{31}P and ^{13}C spectra were recorded with a Bruker Avance III 500 MHz NMR (500 MHz, 202 MHz and 126 MHz) instrument at 300 K or with a Bruker Avance DPX 300 spectrometer (300 MHz, 122 MHz and 76 MHz) at 302 K. Proton spectra were referenced to TMS as an internal standard. Carbon chemical shifts were determined relative to the ^{13}C signal of $CDCl_3$ (77.0 ppm). ^{31}P NMR spectra were referenced to external 85% phosphoric acid (δ = 0 ppm) and were acquired with a Bruker ^{31}P composite pulse decoupling (CPD) program. Assignments of some proton, carbon and phosphorous resonances were performed by 2D NMR techniques (^{1}H–^{1}H *gs*-COSY, ^{1}H–^{13}C *gs*-HSQC, ^{1}H–^{13}C *gs*-HMBC and ^{1}H–^{31}P *gs*-HMBC). Coupling constants (*J*) are given in Hz. Multiplicities are indicated as follows: s (singlet), d (doublet), t (triplet), m (multiplet) or br (broadened).

High resolution mass spectra (HRMS) were recorded on a time-of-flight (TOF) mass spectrometer equipped with a double orthogonal electrospray source at atmospheric pressure ionization (ESI) coupled to an HPLC instrument.

Silica gel column chromatography was carried out on silica gel 60N.

All reactions (*Reaction a*, *Reaction b* and transmetallation reactions) were conducted at least in triplicates, always returning consistent results.

The details of the theoretical study are in the section Computational Investigations.

Supplementary Table 1 | ^{31}P NMR chemical shifts of phosphorous containing compounds.

Entry	Compound	δ_P (ppm)	Literature δ_P (ppm)
1	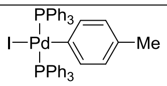 **4**	+22.5	+22.5, Ref.[1]<br>+23.1, Ref.[2]
2	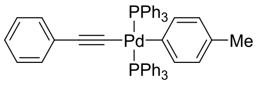 **5**	+26.3	
3	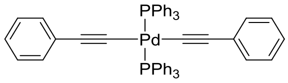 **6**	+25.9	+25.58, Ref.[3]
4	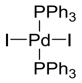 **7**	+12.8	+13.3, Ref.[4]<br>+12.78, Ref.[5]
5	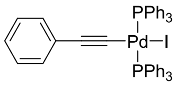 **8**	+22.9	+23.6, Ref.[6]
6	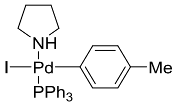 **9**	+31.5	
7	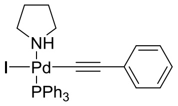 **10**	+30.3	
8	$Pd^0(PPh_3)_2$	+33.0	
9	$Pd^0(PPh_3)_3$	(+11.9 - –5.3)[#]	+0.61, −5.17, Ref.[7]
10	PPh_3	−5.4	
11	$O{=}PPh_3$	+29.0	

[#] Depending on concentration and composition.

Complexes **4**, **5**, **6**, **7**, **8**, **9** and **10** were identified by their characteristic ^{31}P NMR chemical shifts and comparison with authentic independently prepared samples in case of **4**, **6**, **7** and **8**. Complexes **5**, **9** and **10** were characterized in the reaction mixture by NMR and ESI HRMS techniques. The resonances for PPh_3 (δ = –5.4 ppm) and $O{=}PPh_3$ (δ = +29.0 ppm) were assigned by independently prepared $CDCl_3$ solutions of commercially available triphenylphospine and triphenyphospine oxide.

The ^{31}P resonances at δ = –5.5 ppm ($[Pd^0(PPh_3)_3]$) and δ = +33.0 ppm ($[Pd^0(PPh_3)_2]$) belong to the corresponding Pd^0 species in a fast equilibrium, and result from successive dissociations of PPh_3 ligands from $[Pd^0(PPh_3)_4]$. This assignment is in line with the literature data,[7] as well as an independent NMR experiment where ^{31}P NMR of $[Pd^0(PPh_3)_4]$, freshly dissolved in $CDCl_3$, revealed the same two resonances as indicated above, along with that for the starting complex at δ = 27.7 ppm. The chemical shift also strongly depends on the overall concentration of the complex.[8,9] Pd(II) in phosphine complexes can be reduced by adventitious water to Pd(0) with concurrent phosphine oxidation to form $O=PPh_3$.[9] $O=PPh_3$ can also be a result of PPh_3 oxidation with trace amounts of oxygen that could not be totally excluded from the reaction mixture. Broad resonance of **6** in spectra for *Reaction a* is due to fluxionality with PPh_3 ligand as demonstrated in independent experiment (Supplementary Fig. 23).

Supplementary Note 1. Model reactions

Reaction a

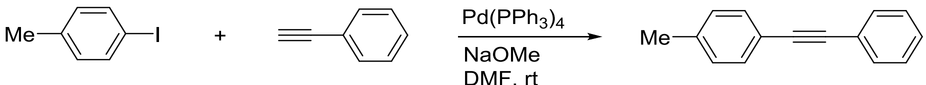

1 2 3

Reaction conditions for *Reaction a* were similar to those from the literature.[10]

To a stirred mixture of 4-iodotoluene (**1**, 545 mg, 2.5 mmol), phenylacetylene (**2**, 281 mg, 2.75 mmol), sodium methoxide (149 mg, 2.75 mmol) and dry *N*,*N*-dimethylformamide (5 mL), $Pd(PPh_3)_4$ (578 mg, 0.5 mmol, 20 mol% of Pd or 58 mg, 0.05 mmol, 2.0 mol% of Pd) was added under argon atmosphere at room temperature (1,3,5-trimethoxybenzene was added as internal standard). Stirring was continued at room temperature. After given time an aliquot (50 μL) was directly diluted with deuterated chloroform (0.6 mL), transferred into NMR tube, and ^{1}H and ^{31}P NMR were recorded immediately. It has been confirmed that this workup completely stops the reaction by re-acquiring the ^{1}H NMR spectrum of the same sample after being aged in the NMR tube for 1 h, with the same result. The conversion into product **3** was determined by qNMR from integrals of methyl protons of product **3** using 1,3,5-trimethoxybenzene as internal standard and is shown in Fig. 2b. The reaction was repeated 4-times, always returning consistent results.

Product **3** was used as a standard and was for this purpose isolated by silica gel column chromatography using hexanes/ethyl acetate 10/1 as a mobile phase.

1-Methyl-4-(phenylethynyl)benzene (**3**):

^{1}H NMR (500 MHz, $CDCl_3$): δ 7.54–7.50 (m, 2H), 7.42 (d, J = 7.8 Hz, 2H), 7.38–7.30 (m, 3H), 7.14 (d, J = 7.8 Hz, 2H), 2.37 (s, 3H). The data are in agreement with those from the literature[11].

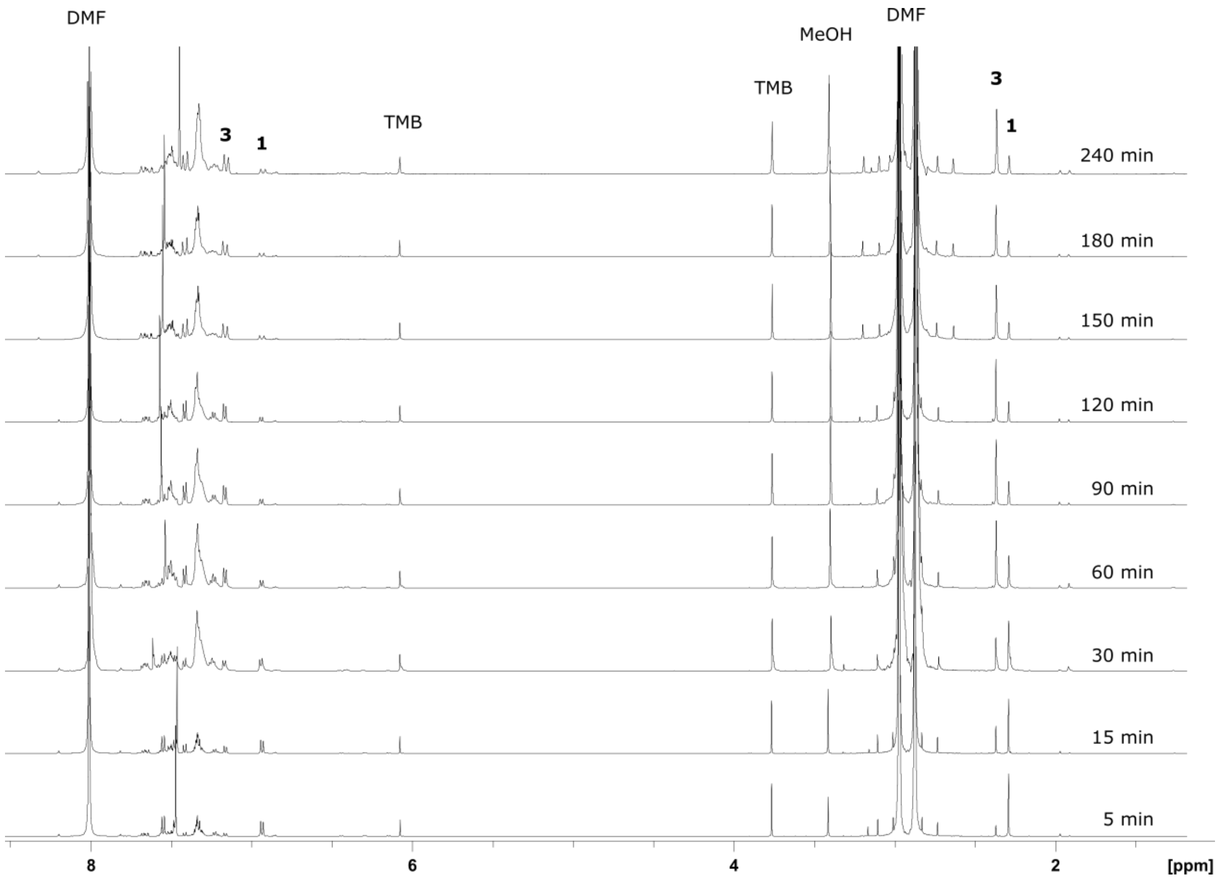

Supplementary Figure 1 | Stacking of ^{1}H NMR spectra of aliquots of *Reaction a* with 20 mol% loading of Pd ([Pd] = 0.1 M) over time. TMB = 1,3,5-trimethoxybenzene; **1** = 4-iodotoluene; **3** = 1-methyl-4-(phenylethynyl)benzene.

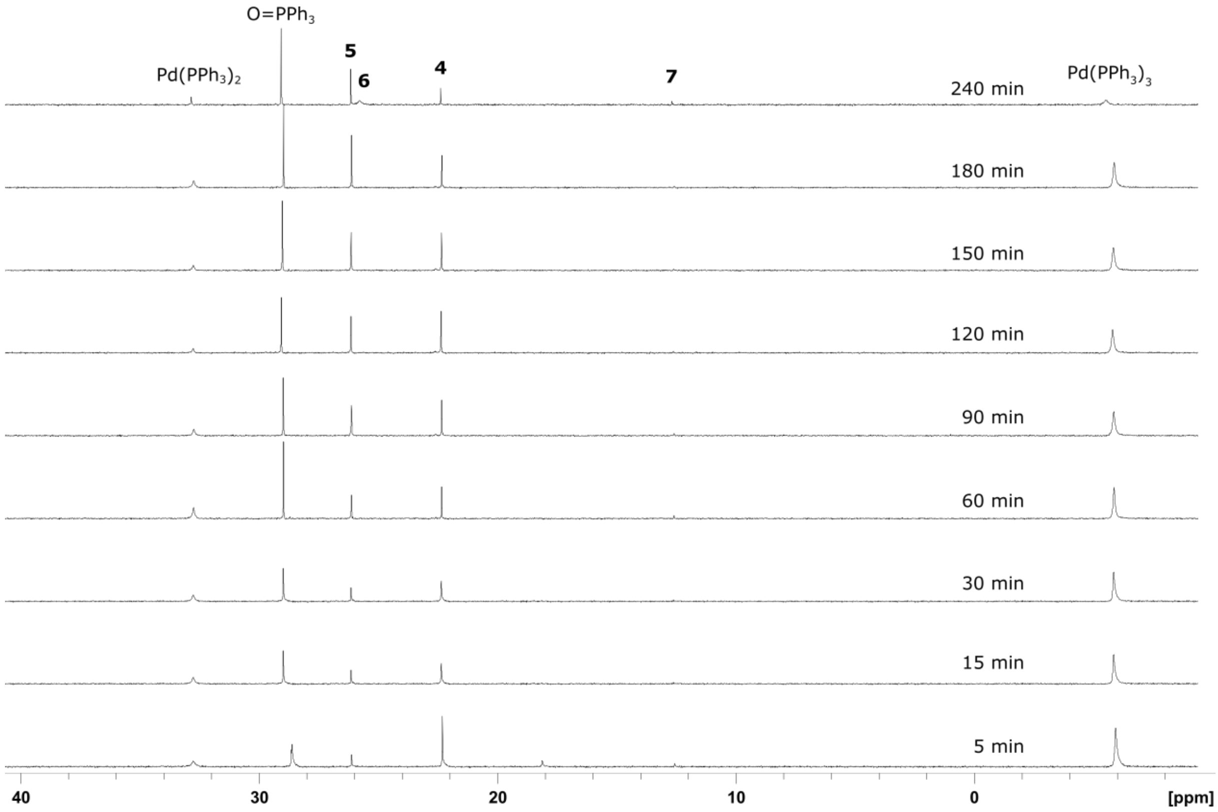

Supplementary Figure 2 | Stacking of ^{31}P NMR spectra of aliquots of *Reaction a* with 20 mol% loading of Pd ([Pd] = 0.1 M) over time. For structures of **4**, **5**, **6**, **7**, please see Supplementary Table 1. Snapshots after 1 h and 4 h are presented in Fig. 2c.

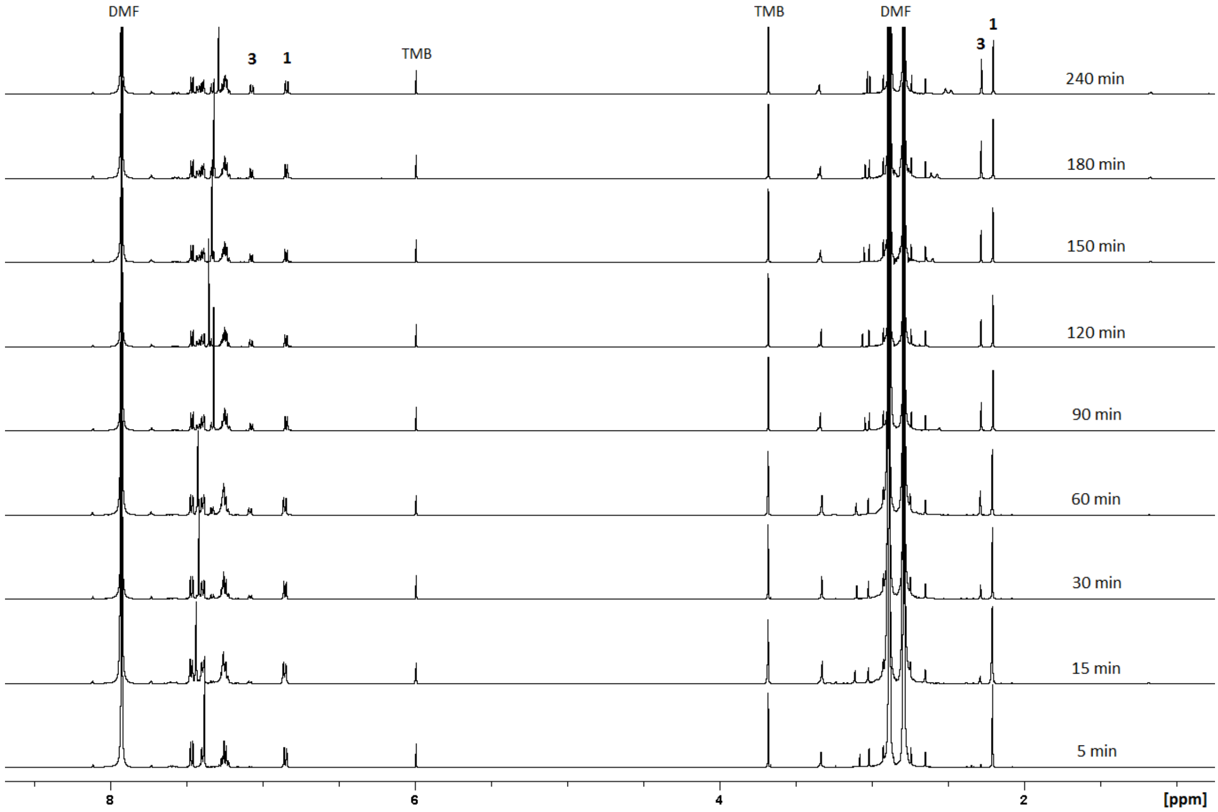

Supplementary Figure 3 | Stacking of ^{1}H NMR spectra of aliquots of *Reaction a* with 2 mol% loading of Pd ([Pd] = 0.01 M) over time. TMB = 1,3,5-trimethoxybenzene; **1** = 4-iodotoluene; **3** = 1-methyl-4-(phenylethynyl)benzene.

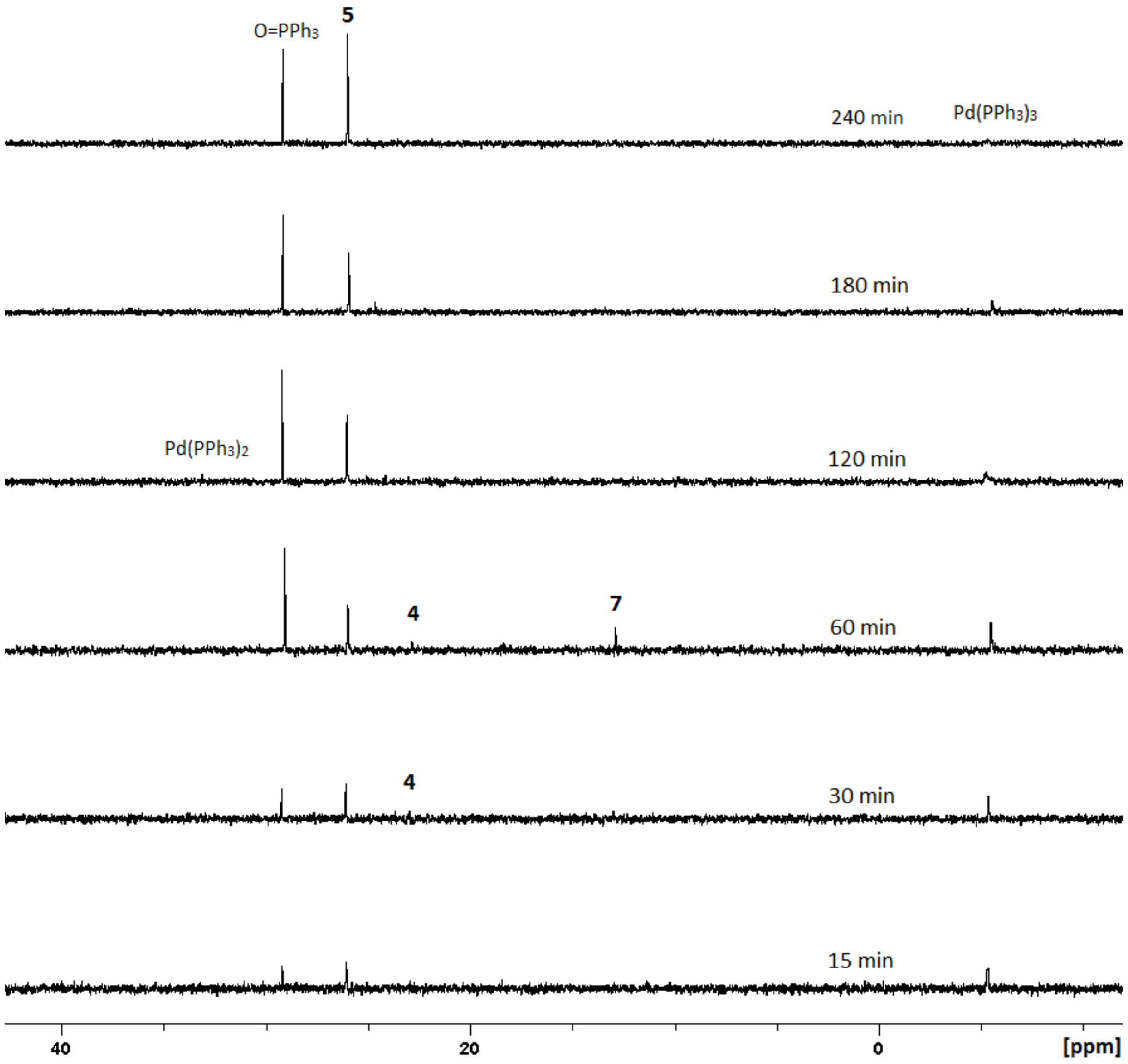

Supplementary Figure 4 | Stacking of ^{31}P NMR spectra of aliquots of *Reaction a* with 2 mol% loading of Pd ([Pd] = 0.01 M) over time. For structures of **4**, **5**, **7**, please see Supplementary Table 1. ^{31}P NMR spectra were acquired in decoupled mode (ns = 256).

<u>*Reaction b*</u>

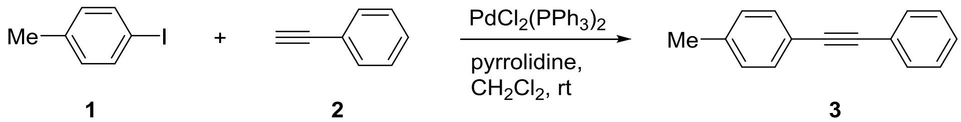

1 **2** **3**

Reaction conditions for *Reaction b* were taken from the literature.[12]

To a stirred mixture of 4-iodotoluene (**1**, 545 mg, 2.5 mmol), phenylacetylene (**2**, 302 µL, 281 mg, 2.75 mmol), pyrrolidine (420 µL, 355 mg, 5 mmol) in dry dichloromethane (5 mL), bis(triphenylphosphine)palladium(II) dichloride (351 mg, 0.50 mmol, 20 mol % of Pd or 35 mg, 0.05 mmol, 2.0 mol% of Pd) was added at room temperature under an argon atmosphere (1,3,5-trimethoxybenzene was added as internal standard). Stirring was continued at room temperature. After given time an aliquot (50 µL) was directly diluted with deuterated chloroform (0.6 mL), transferred into NMR tube, and ^{1}H and ^{31}P NMR were recorded immediately. It has been confirmed that this workup completely stops the reaction by re-acquiring the ^{1}H NMR spectrum of the same sample after being aged in the NMR tube for 1 h, with the same result. The conversion into product **3** was determined by qNMR from integrals of methyl protons of product **3** using 1,3,5-trimethoxybenzene as internal standard and is shown in Fig. 2b. The reaction was repeated 3-times, always returning consistent results.

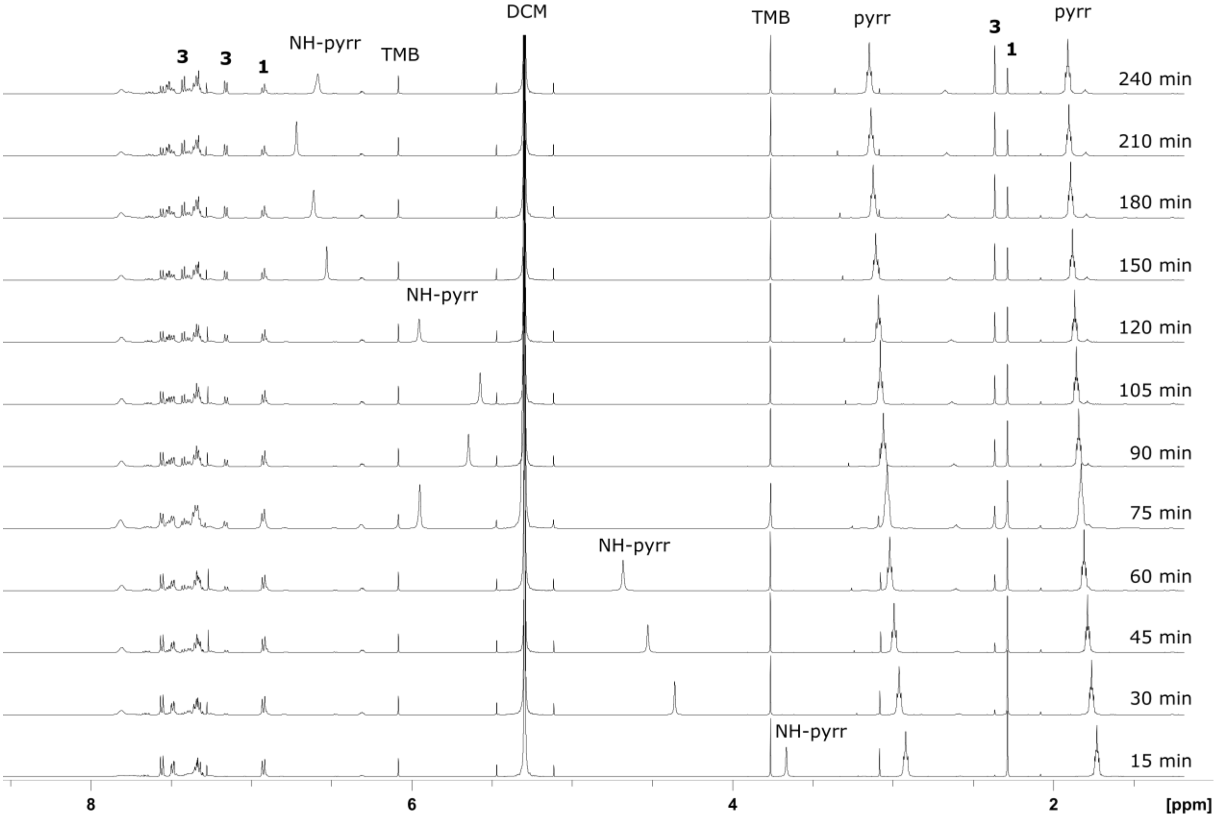

Supplementary Figure 5 | Stacking of ^{1}H NMR spectra of aliquots of *Reaction b* with 20 mol% of Pd ([Pd] = 0.1 M) over time. TMB = 1,3,5-trimethoxybenzene; **1** = 4-iodotoluene; **3** = 1-methyl-4-(phenylethynyl)benzene, pyrr = pyrrolidine; DCM = dicholormethane.

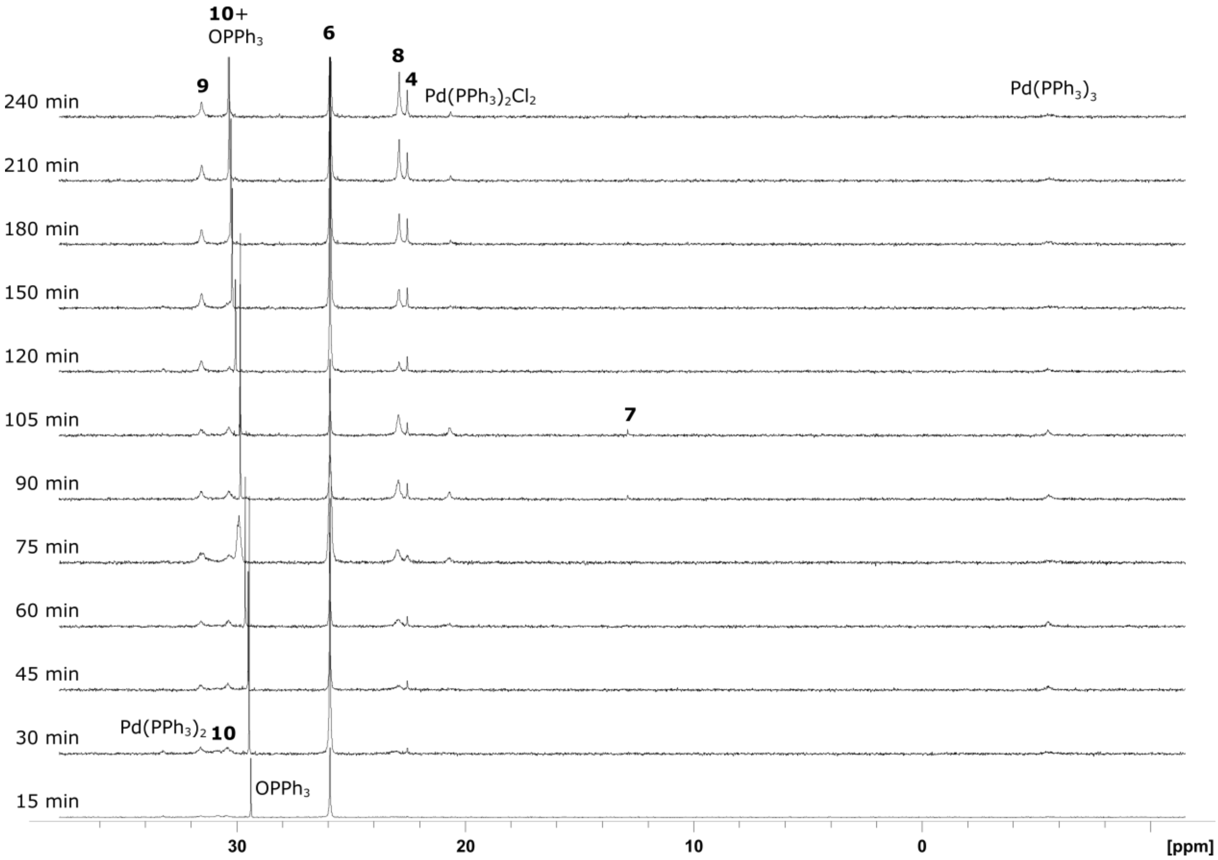

Supplementary Figure 6 | Stacking of ^{31}P NMR spectra of aliquots of *Reaction b* with 20 mol% loading of Pd ([Pd] = 0.1 M) over time. For structures of **4**, **6**, **8**, **9**, **10** please see Supplementary Table 1. Snapshots after 1 h and 4 h are presented in Fig. 2d.

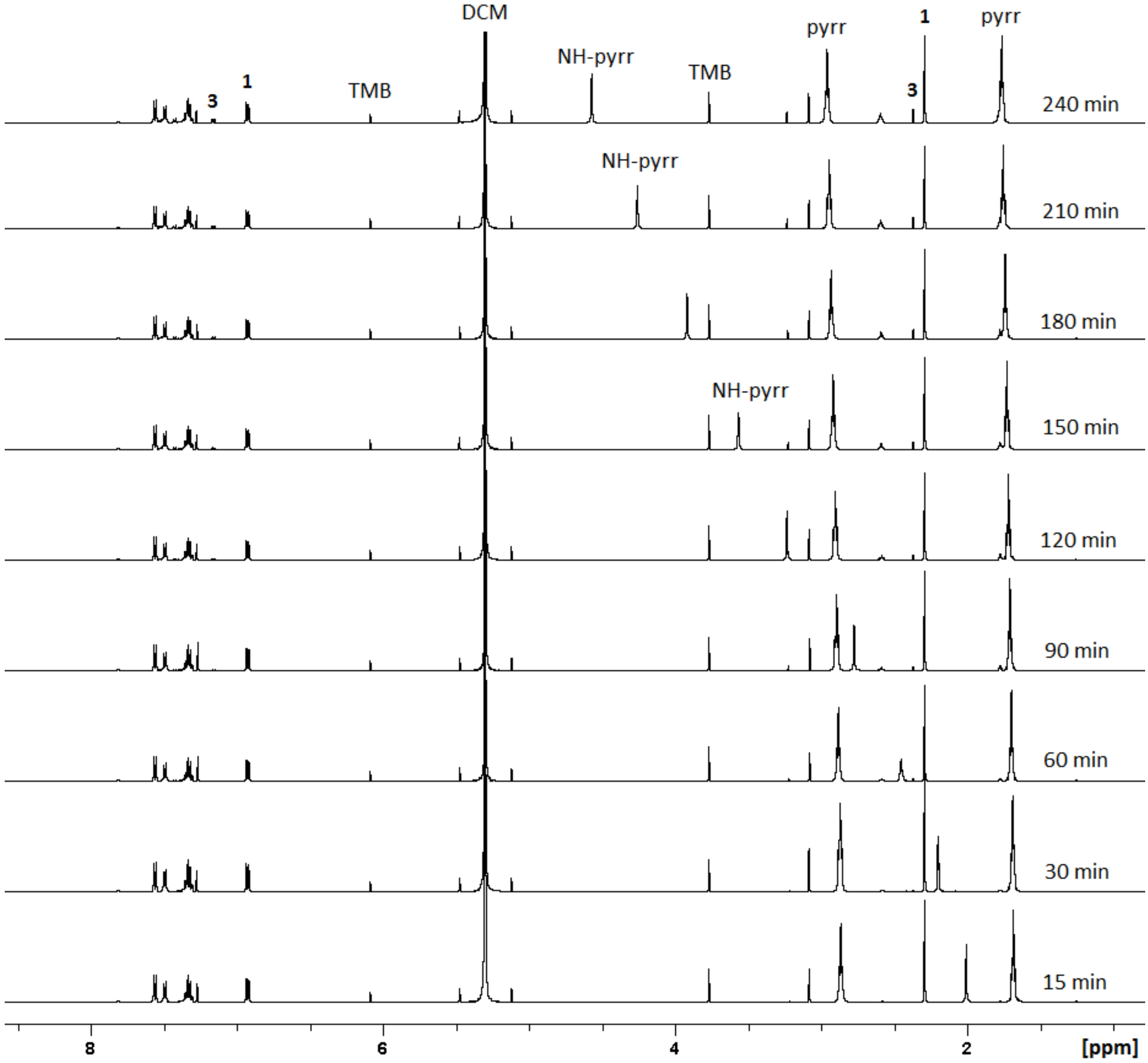

Supplementary Figure 7 | Stacking of ^{1}H NMR spectra of aliquots of *Reaction b* with 2 mol% loading of Pd ([Pd] = 0.01 M) over time. TMB = 1,3,5-trimethoxybenzene; **1** = 4-iodotoluene; **3** = 1-methyl-4-(phenylethynyl)benzene, pyrr = pyrrolidine; DCM = dicholormethane.

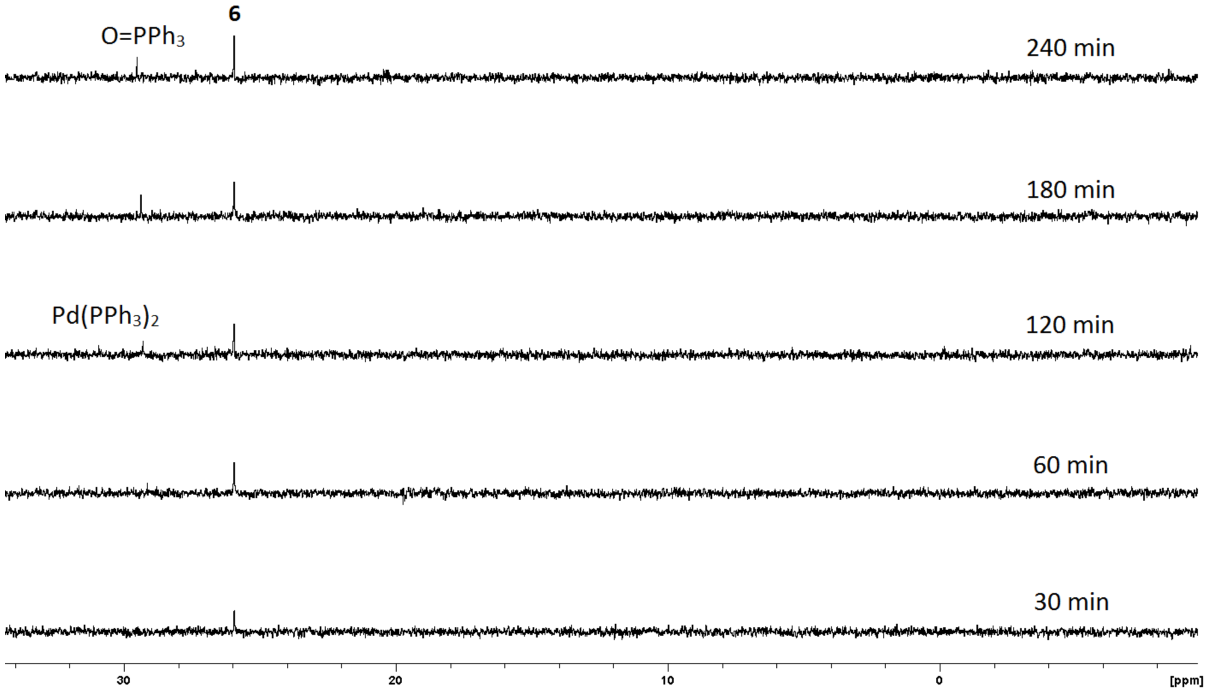

Supplementary Figure 8 | Stacking of ^{31}P NMR spectra of aliquots of *Reaction b* with 2 mol% loading of Pd ([Pd] = 0.01 M) over time. For the structure of **6** please, see Supplementary Table 1. ^{31}P NMR spectra were acquired in decoupled mode (ns = 256).

Supplementary Note 2. Preparation and characterization of palladium compounds 4–10

Spectroscopic data (^{1}H, ^{13}C and ^{31}P NMR spectra) for compounds **4**, **6**, **7** and **8** were in agreement with the literature reports. For some of these compounds there was no full spectroscopic characterization available in the literature. Because of their importance for the presented work, and for comparison reasons for determination of compound **5**, proton and carbon resonances of compounds **4**, **6** and **8** were assigned by 1D and 2D NMR techniques (^{1}H NMR, ^{13}C NMR, ^{31}P NMR, ^{1}H–^{1}H *gs*-COSY, ^{1}H–^{13}C *gs*-HSQC, ^{1}H–^{13}C *gs*-HMBC and ^{1}H–^{31}P *gs*-HMBC). H2'–H6' and C1'–C6' denotes proton and carbon resonances of the phenyl parts of $P(Ph_3)$ groups in the molecules.

Compound **4** (oxidative adduct, *trans*-**A**)

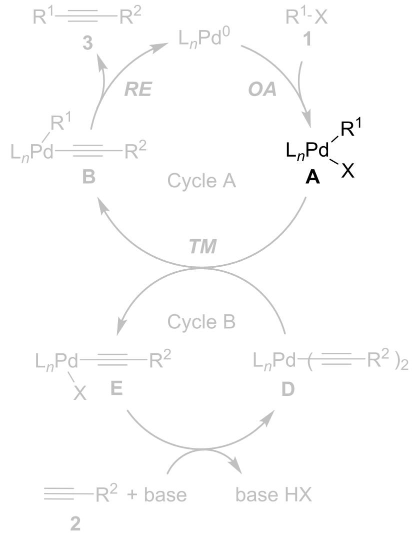

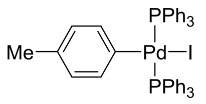

4

trans-**A**

(R^1= 4-Me-C_6H_4-, X = I, L = PPh_3, *n* = 2)

Oxidative adduct **4** was synthesized following the literature procedure.[13]

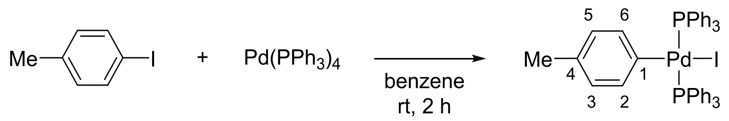

1 **4**

A mixture of 4-iodotoluene (**1**, 150 mg, 0.69 mmol), tetrakis(triphenylphosphine)palladium(0) (320 mg, 0.28 mmol) and benzene was stirred under argon atmosphere in dark at room temperature. Then, the reaction mixture was concentrated under reduced pressure and the crude product was triturated with diethyl ether to obtain pure product **4** (228 mg, 96%) as a white solid. Crude

product was additionally crystallized from chloroform/hexane to obtain pale crystals (130 mg, 55%).

4:

^{1}H NMR (500 MHz, $CDCl_3$): δ 7.54–7.46 (m, 12H, H2' and H6'), 7.34–7.28 (m, 6H, H4'), 7.26–7.19 (m, 12H, H3' and H5'), 6.44–6.39 (m, 2H, H2 and H6), 6.07 (d, J = 7.6 Hz, 2H, H3 and H5), 1.91 (s, 3H, CH_3);

^{13}C NMR (126 MHz, $CDCl_3$): δ 152.7 (t, J = 2.4 Hz, C1, C-Pd), 135.5 (t, J = 5.1 Hz, C2 and C6), 134.9 (t, J = 6.3 Hz, C2' and C6'), 132.2 (t, J = 22.9 Hz, C1'), 129.6 (C4'), 130.9 (C4), 128.9 (C3 and C5), 127.7 (t, J = 5.0 Hz, C3' and C5'), 20.1 (CH_3);

^{31}P NMR (202 MHz, $CDCl_3$): δ +22.5 (s). Lit.:[1] δ + 22.5 (s) (C_6D_6). Lit.:[2] δ + 23.1 (s) ($CDCl_3$).

<u>Compound **5** (transmetallation product, *trans*-**B**)</u>

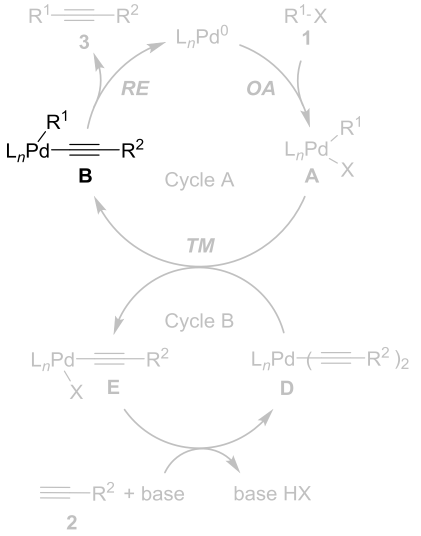

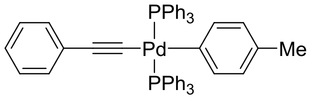

5

trans-**B**
(R^1= 4-Me-C_6H_4-, R^2 = Ph, L = PPh_3, n = 2)

Compound **5** was characterized in reaction mixture (see procedure bellow) by 1D and 2D NMR spectroscopy and HRMS.

Independent preparation of **5**

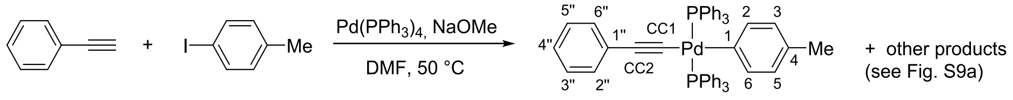

1 **2** **5**

A mixture of 4-iodotoluene (**1**, 109 mg, 0.5 mmol), phenylacetylene (**2**, 66 mg, 0.65 mmol), tetrakis(triphenylphosphine)palladium(0) (289 mg, 0.25 mmol, 50 mol%), sodium methoxide (35 mg, 0.65 mmol) and *N*,*N*-dimethylformamide (0.25 mL) was

stirred under argon atmosphere at 50 °C for 15 min. An aliquot (ca 50 μL) of the reaction mixture was diluted with $CDCl_3$ (0.7 mL), washed with water (3 × 0.5 mL), dried over sodium sulphate and filtered. ^1H NMR spectrum revealed the presence of starting compounds **1**, **2**, product **3**, oxidative adduct **4**, transmetallation product **5**, PPh_3 and $O{=}PPh_3$. ^{31}P NMR spectrum indicated the presence of $O{=}PPh_3$, **4**, **5**, **7** and PPh_3 (Supplementary Fig. 9a).

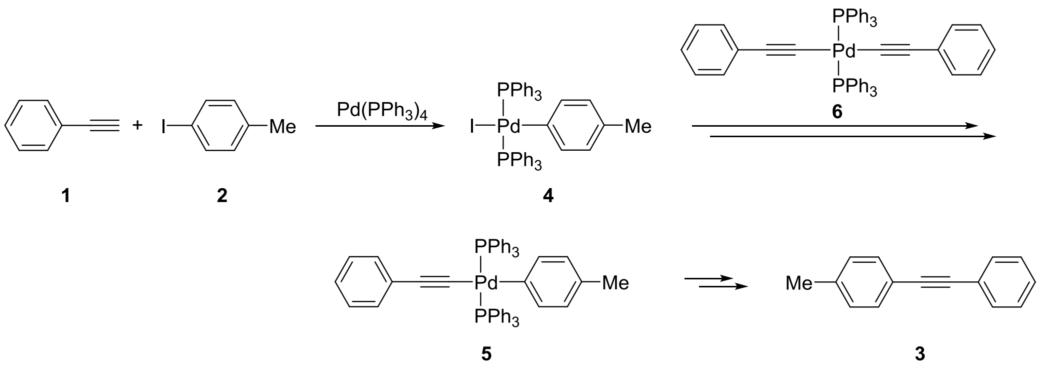

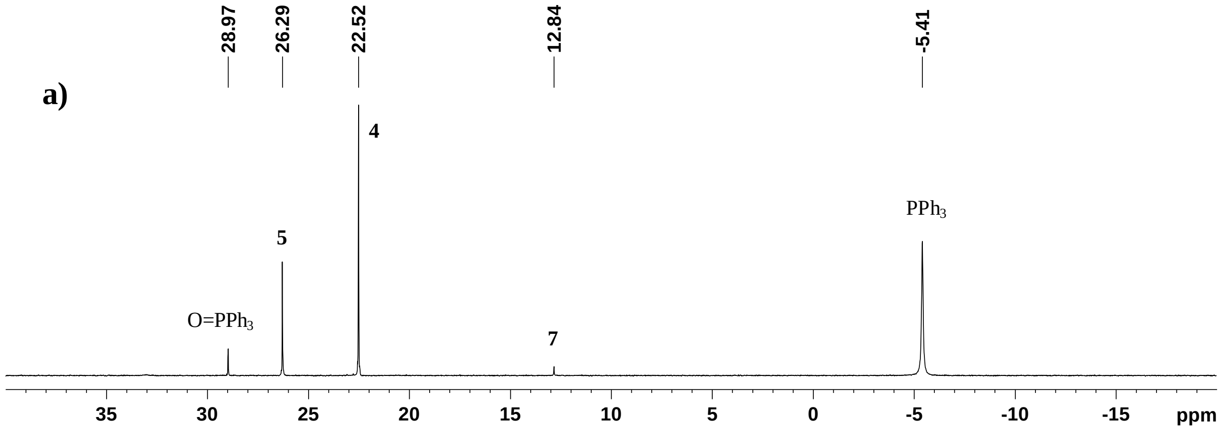

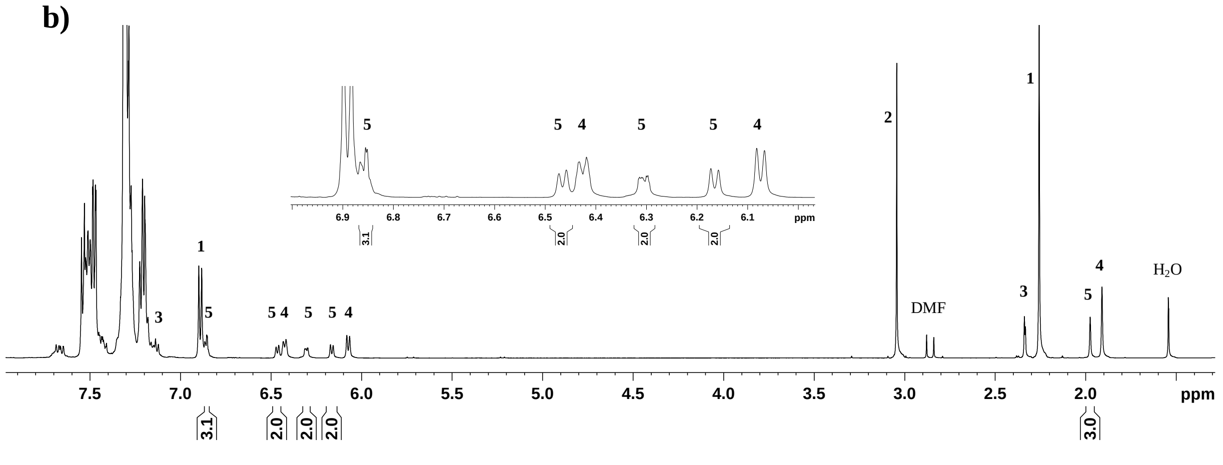

Supplementary Figure 9 | Spectroscopic data for characterization of compound **5. a**, ^{31}P NMR spectrum. **b**, ^1H NMR spectrum of the mixture prepared as described above.

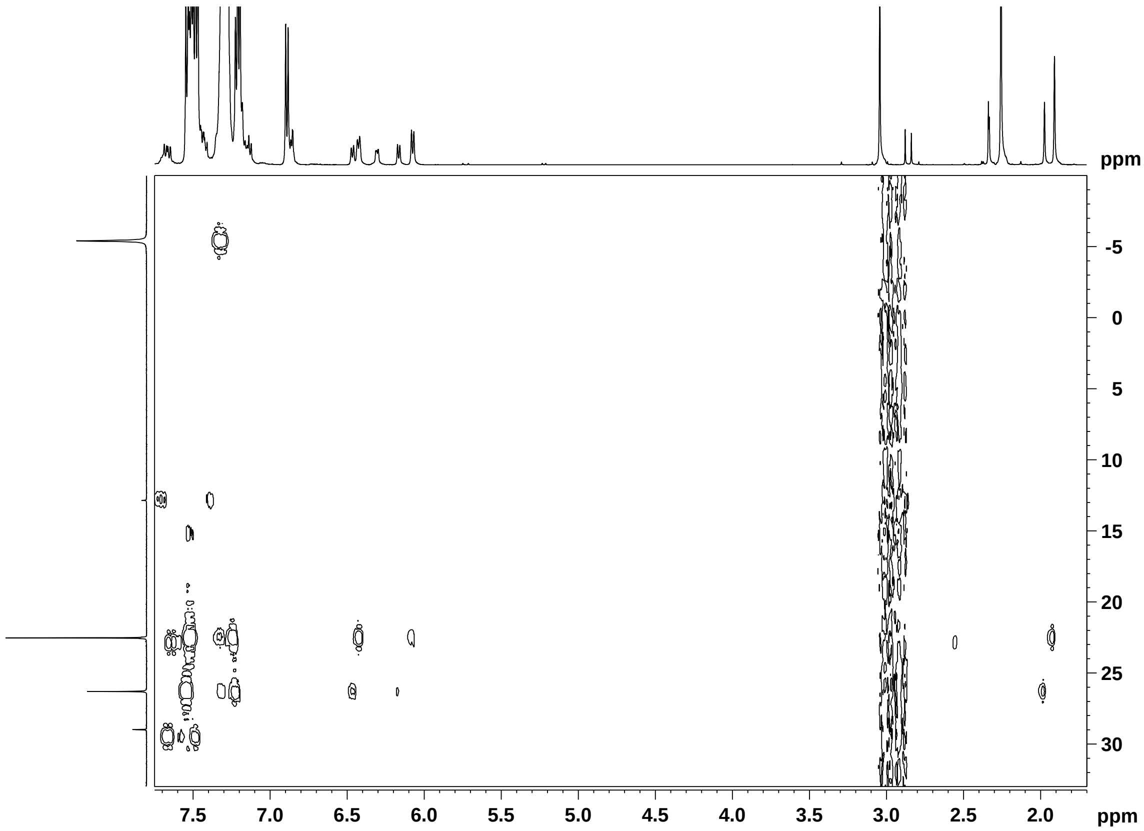

Supplementary Figure 10 | ^{1}H-^{31}P HMBC (long range $^{n}J_{H\text{-}P}$ = 10 Hz) spectrum for characterization of compound **5** (^{31}P NMR δ = +26.3 ppm). Mixture prepared as described above.

The structure of compound **5** was determined by ^{1}H NMR, ^{13}C NMR, ^{31}P NMR, ^{1}H–^{1}H *gs*-COSY, ^{1}H–^{13}C *gs*-HSQC, ^{1}H–^{13}C *gs*-HMBC and ^{1}H–^{31}P *gs*-HMBC NMR techniques (spectra are enclosed in the NMR spectra section).

Key observations: Triplet shaped C1 carbon resonance (Pd-C of aryl substituent) indicated *trans*-configuration of PPh_3 substituents[14]. Aryl and acetylene protons of **5** were assigned by ^{1}H–^{31}P *gs*-HMBC and ^{1}H–^{1}H *gs*-COSY. Similarly, carbon resonances of **5** were assigned by ^{1}H–^{13}C *gs*-HSQC and ^{1}H–^{13}C *gs*-HMBC. It is important to note that the proton and the carbon resonances of **5** are similar to those of **4** (for the aryl part of the molecule) and **6** (for the acetylene part of the molecule).

5:

^{1}H NMR (500 MHz, $CDCl_3$): *δ* 6.87–6.84 (m, 3H, H3", H4" and H5"), 6.47 (d, *J* = 7.4 Hz, 2H, H2 and H6), 6.33–6.28 (m, 2H, H2" and H6"), 6.16 (d, *J* = 7.4 Hz, 2H, H3 and H5), 1.98 (s, 3H, CH_3),

$P(Ph_3)$ resonances of **5** were overleaped with $P(Ph_3)$ resonances from other compounds in the mixture, although ^{1}H-^{31}P *gs*-HMBC indicated three cross-peaks of ^{31}P NMR (+26.3) with ^{1}H NMR (*orto, para, meta* protons of $P(Ph_3)$) at *δ*: 7.55, 7.32 and 7.23 ppm;

^{13}C NMR (126 MHz, $CDCl_3$): *δ* 156.0 (t, *J* = 6.3 Hz, C1, C-Pd), 138.0 (t, *J* = 3.8 Hz, C2 and C6), 130.6 (C2" and C6"), 130.2 (C4), 128.0 (C1"), 127.8 (C3 and C5), 127.0 (C3" and C5"), 124.0 (C4"), 20.5 (CH_3), intensities of CC1 and CC2 were too low to be detected.

^{31}P NMR (202 MHz, $CDCl_3$): *δ* +26.3 (s),

HMRS (ESI+) (*m*/*z*): $[M + H]^+$ calcd for $C_{51}H_{43}P_2Pd^+$: 823.1869; found: 823.1885.

Compound **6** (palladium bis-acetylide, *trans*-**D**)

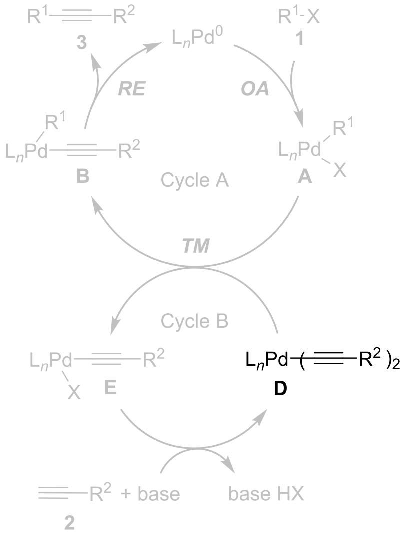

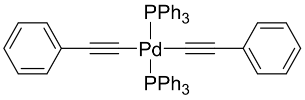

6

trans-**D**
(R^2 = Ph, L = PPh_3, *n* = 2)

Reaction of 8 with 2 into 6 (Cycle B)

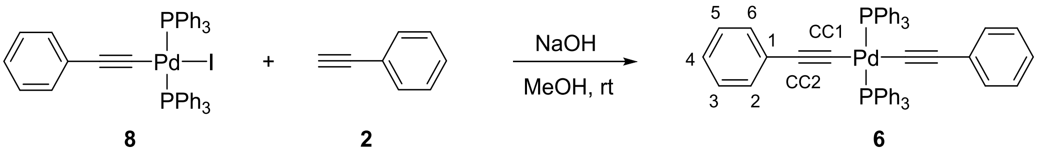

A mixture of **8** (120 mg, 0.14 mmol), phenylacetylene (**2**, 197 µL, 183 mg, 1.79 mmol) and 0.2 M solution of NaOH in methanol (3.5 mL) was stirred at room temperature in dark. After 16 h the precipitate was filtered off, washed with water (5 mL), methanol (5 mL) and dried *in vaccuo* to afford pure **6** (111 mg, 95%) as a white solid. The crude product was additionally crystallized from benzene/ethanol to give purified **6** (97 mg, 83%).

6:

^{1}H NMR (500 MHz, $CDCl_3$): δ 7.84–7.78 (m, 12H, H2’ and H6’), 7.42–7.35 (m, 6H, H4'), 7.36–7.30 (m, 12H, H3' and H5'), 6.92–6.88 (m, 6H, H3, H4 and H5), 6.34–6.29 (m, 4H, H2 and H6);

^{13}C NMR (126 MHz, $CDCl_3$): δ 135.0 (t, J = 6.4 Hz, C2' and C6'), 132.5 (t, J = 24.6 Hz, C1'), 130.8 (C2 and C6), 129.9 (C4'), 128.1 (C1), 127.9 (t, J = 5.3 Hz, C3' and C5'), 127.1 (C3 and C5), 124.7 (C4), 114.8 (t, J = 4.3 Hz, CC2), 113.6 (t, J =16.8 Hz, CC1, C-Pd);

^{31}P NMR (202 MHz, $CDCl_3$): δ +25.9 (s). Lit.:[3] δ +26.58 (s) $CDCl_3$.

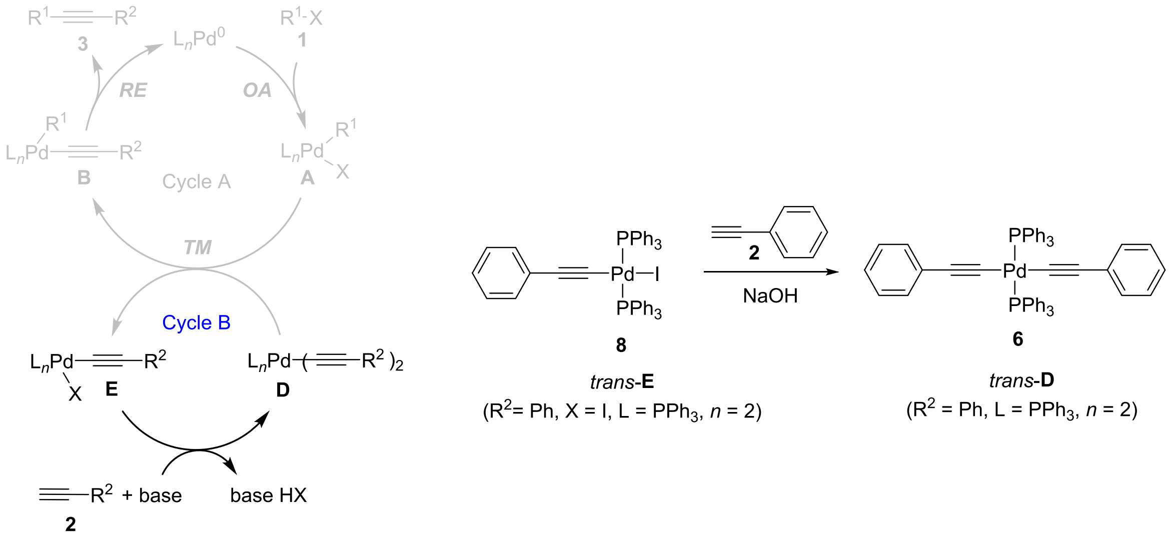

Supplementary Figure 11 | Cycle B. Regeneration of **6** from **8** with acetylene and base.

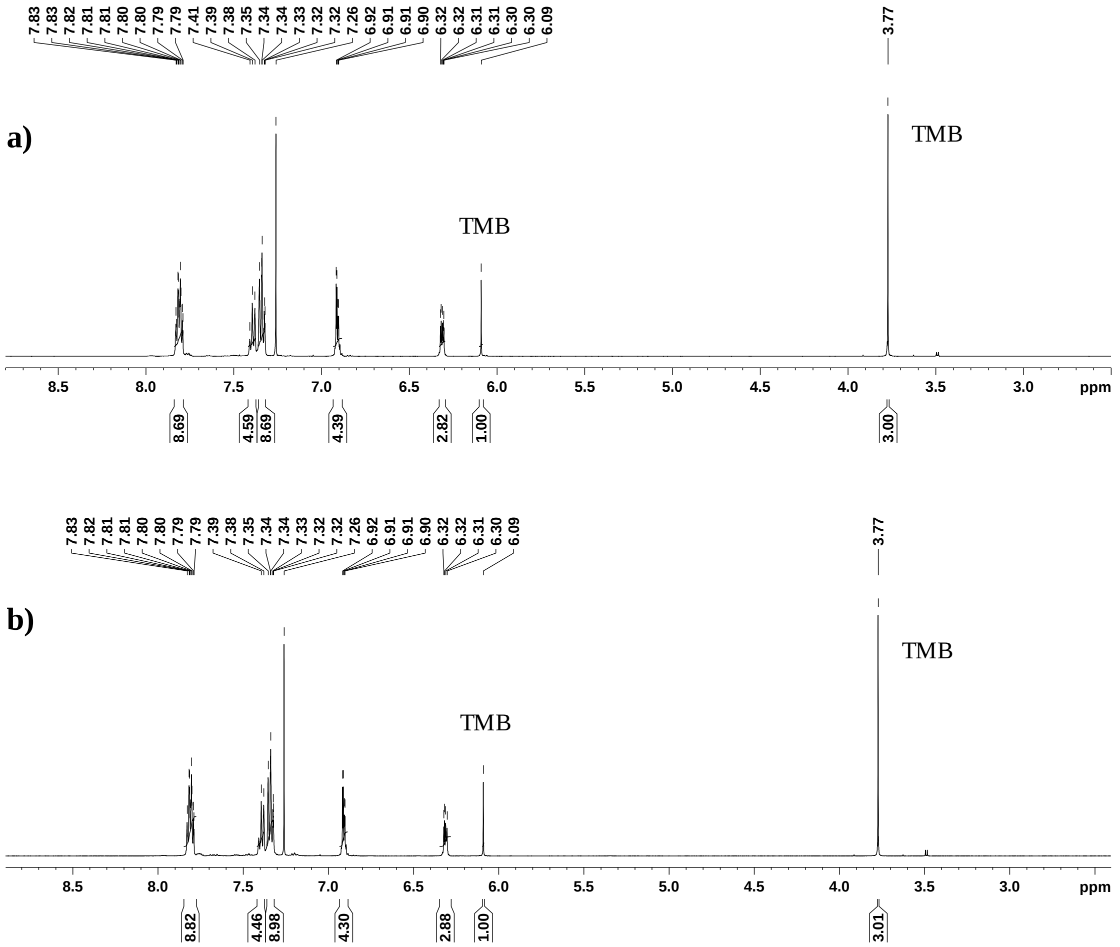

c)

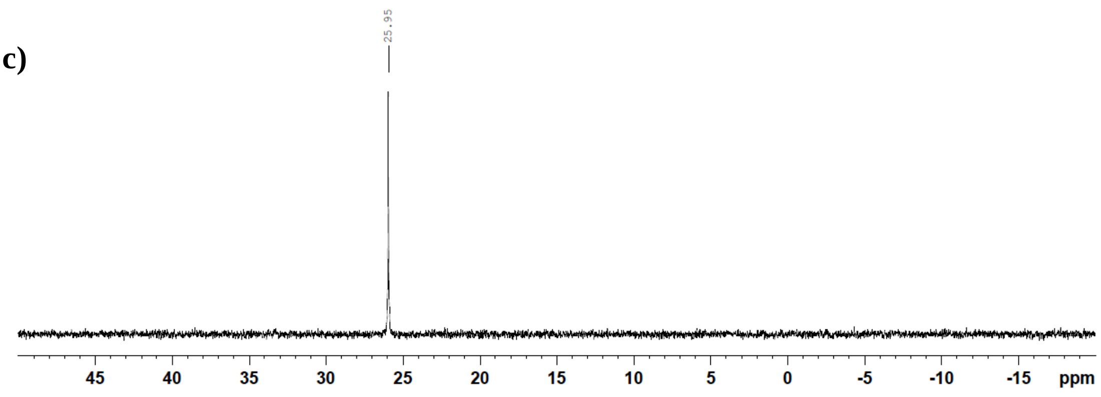

d)

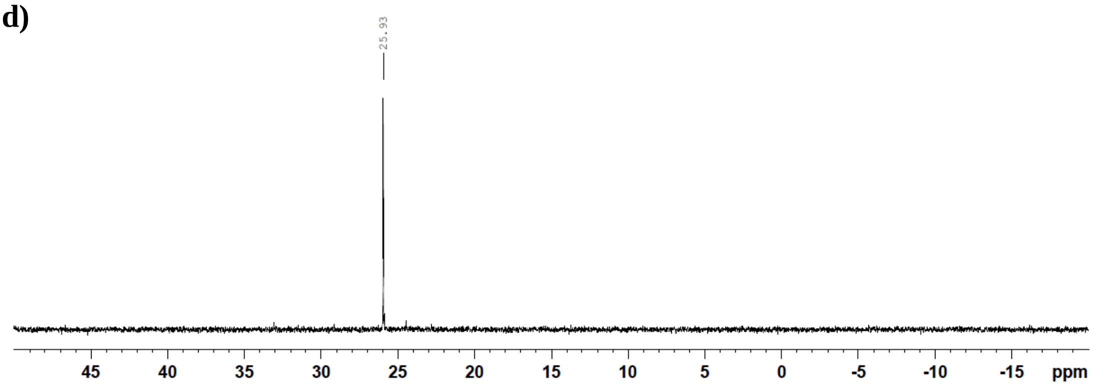

Supplementary Figure 12 | ^{1}H NMR spectra of solution of **6** (7.6 mg) in $CDCl_3$ (0.7 mL) after **a,** 10 minutes and **b,** 24 h; and ^{31}P NMR spectra of this solution after **c,** 10 minutes and **d,** 24 h. Compound **6** is stable in solution as demonstrated by integration using 1,3,5-trimethoxybenzene (TMB) as internal standard.

Compound **7**

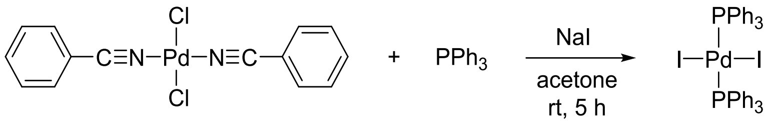

7

Compound **7** was prepared following a modified literature procedure[4].

To a mixture of bis(benzonitrile)palladium(II) chloride (95 mg, 0.25 mmol) and acetone (1.5 mL), triphenylphospine (131 mg, 0.5 mmol) and sodium iodide (1.12 g, 7.5 mmol) were added at 0 °C. Then, the mixture was stirred at room temperature for 5 h. The solvent was removed under reduced pressure and dichloromethane was added (30 mL). The suspension was filtered through a short pad of Celite to give red solution, which was concentrated to smaller volume (10 mL). Hexane (5 mL) was added to this solution, which was left standing at 4 °C. After 2 days red needles, product **7** (28 mg, 13%), were collected by filtration.

7:

^{1}H NMR (500 MHz, $CDCl_3$): δ 7.73–7.66 (m, 12H, H2' and H6'), 7.40–7.35 (m, 18 H, H3', H5' and H4');

^{13}C NMR (126 MHz, $CDCl_3$): δ 135.2 (t, J = 5.7 Hz, C2' and C6'), 134.0 (t, J = 25.3 Hz, C1'), 130.5 (C2 and C6), 130.2 (C4'), 127.6 (t, J = 5.4 Hz, C3' and C5');

^{31}P NMR (202 MHz, $CDCl_3$): δ +12.8 (s). Lit.:[5] +12.78 (s) $CDCl_3$. Lit.:[4] δ +13.3 (s) $CDCl_3$.

Compound **8** (transmetallation product, *trans*-**E**)

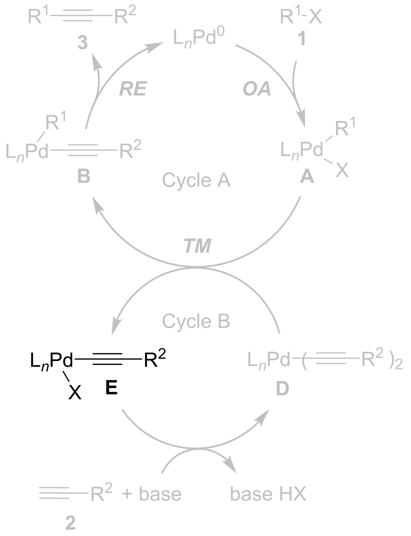

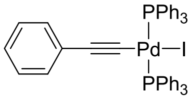

8

trans-**E**
(R^2= Ph, X = I, L = PPh_3, *n* = 2)

Compound **8** was synthesized following the literature procedure[6].

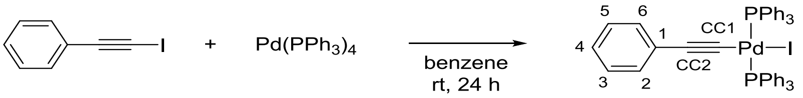

8

A mixture of (iodoethynyl)benzene[15] (125 mg, 0.55 mmol), tetrakis(triphenylphosphine)palladium(0) (578 mg, 0.5 mmol) and benzene (7.5 mL) was stirred under argon atmosphere at room temperature in dark for 24 h. The precipitate was filtered and washed with diethyl ether (2 × 5 mL). The crude product (237 mg, 55% yield) was additionally crystalized from tetrahydrofuran/diethyl ether to obtain pure **8** (145 mg, 34%) as a yellow solid.

8:

^{1}H NMR (500 MHz, $CDCl_3$): δ 7.79–7.73 (m, 12H, H2' and H6'), 7.41–7.31 (m, 18H, H3', H5' and H4'), 6.91–6.87 (m, 1H, H4), 6.86–6.81 (m, 2H, H3 and H5), 6.09–6.06 (m, 2H, H2 and H6);

^{13}C NMR (126 MHz, $CDCl_3$): δ 135.0 (t, *J* = 6.3 Hz, C2' and C6'), 132.7 (t, *J* = 25.2 Hz, C1'), 130.5 (C2 and C6), 130.1 (C4'), 127.2 (C1), 127.8 (t, *J* = 5.4 Hz, C3' and C5'), 127.0 (C3 and C5), 125.1 (C4), 109.5 (t, *J* = 6.8 Hz, CC2), 101.4 (t, *J* =13.8 Hz, CC1, C-Pd);

^{31}P NMR (202 MHz, $CDCl_3$): δ +22.8 (s). Lit.:[6] δ +23.6 (s), $CDCl_3$.

Compound **9** (Equilibrium between **4**, pyrolidine, **9**, and triphenylphospine)

Compound **9** is in equilibrium with oxidative adduct **4** in the presence of pyrrolidine, as shown in the Figure below.

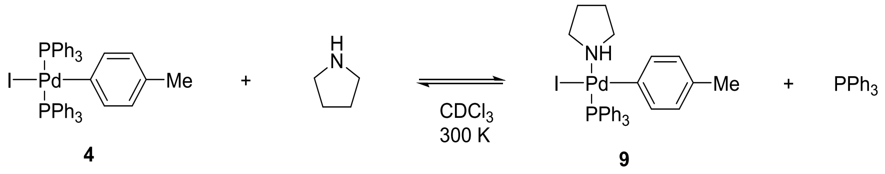

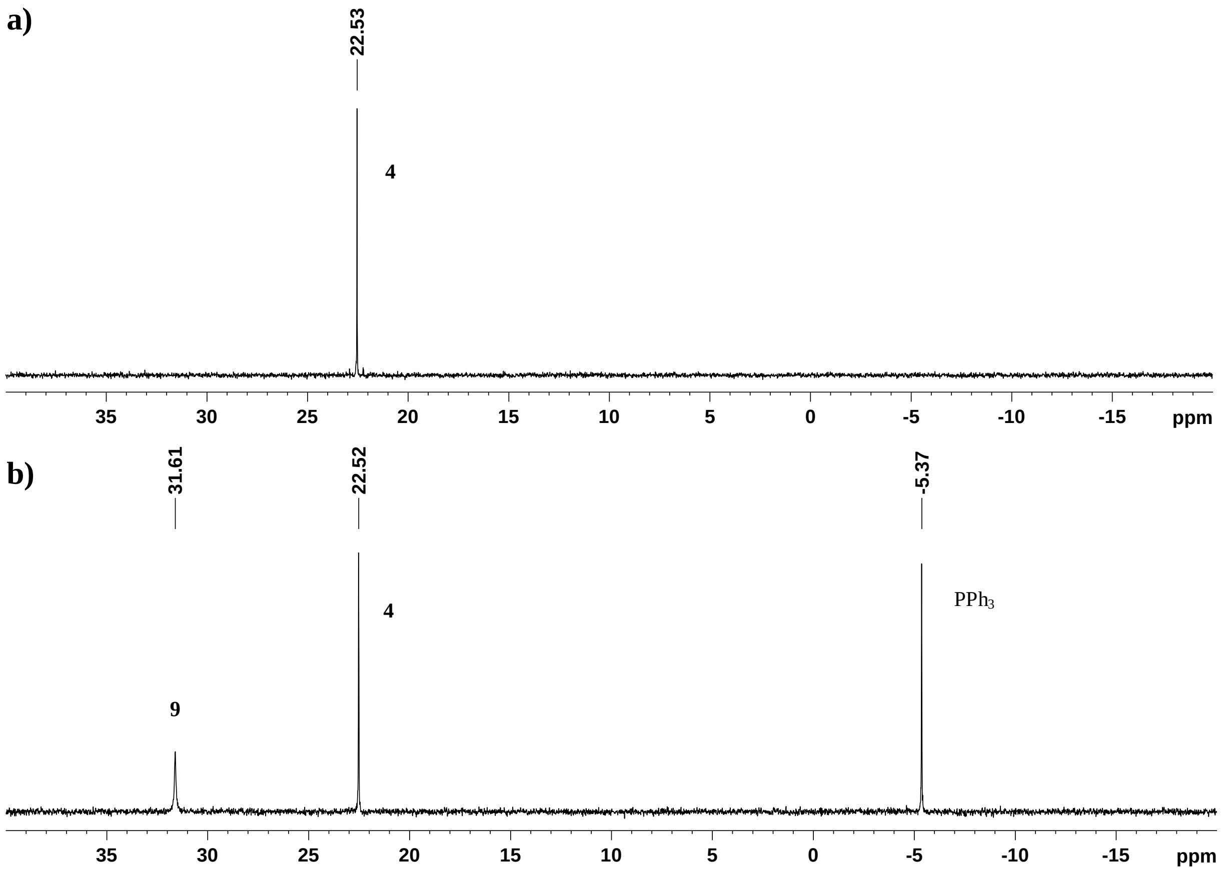

Supplementary Figure 13 | ^{31}P NMR spectrum of the solution of **4** (10.7 mg, 0.013 mmol) in $CDCl_3$ (0.7 mL). **a**, before the addition of pyrrolidine. **b**, after the addition of pyrrolidine (6.5 μL, 5.6 mg, 0.079 mmol, 6 equiv.).

The equilibrium constant was determined as an average of 3 independent experiments. The amount of each component (**4**, **9**, pyrrolidine, and triphenylphospine) in the equilibrium was determined by ^{1}H NMR integration (qNMR) using 1,3,5-trimethoxybenzene as internal standard. Since the resonances of triphenylphospine were overlapped with resonances of triphenylphospine parts from compounds **4** and **9**, the concentration of triphenylphospine was postulated to be the same as the

concentration of **9**. The starting amounts of **4** and pyrrolidine in each experiment (i-iii) are shown below.

$$K = \frac{[\mathbf{9}][\text{triphenylphosphine}]}{[\mathbf{4}][\text{pyrrolidine}]} \qquad \textbf{(1)}$$

i. **4** (10.72 mg, 0.0126 mmol), pyrrolidine (6.70 mg, 0.0942 mmol) in $CDCl_3$ (1.0 mL) at 300 K,
   K = 0.147
ii. **4** (7.33 mg, 0.0086 mmol), pyrrolidine (9.08 mg, 0.127 mmol) in $CDCl_3$ (1.0 mL) at 300 K,
   K = 0.148
iii. **4** (9.97 mg, 0.0117 mmol), pyrrolidine (16.80 mg, 0.236 mmol) in $CDCl_3$ (1.0 mL) at 300 K,
   K = 0.145

K = 0.15 ($CDCl_3$, 300 K)

9:
^{1}H NMR (500 MHz, $CDCl_3$) δ 6.81–6.76 (m, 2H), 6.48 (d, *J* = 7.4 Hz, 2H), 3.56 (br s, 1H), 3.23–3.14 (m, 2H), 2.66–2.57 (m, 2H), 2.09 (s, 3H), 1.60–1.41 (m, 4H), $P(Ph_3)$ resonances of **9** were overleaped with $P(Ph_3)$ resonances from other compounds in the mixture;
^{31}P NMR (202 MHz, $CDCl_3$) δ +31.6 (s);
HMRS (ESI+) (*m*/*z*): $[M + H]^+$ calcd for $C_{29}H_{32}INPPd^+$: 658.0346; found: 658.0347.

Compound **10** (Equilibrium between **8**, pyrolidine, **10**, and triphenylphospine)

Compound **10** is in the equilibrium with palladium mono-acetylide **8** in the presence of pyrrolidine, as shown in the Figure below.

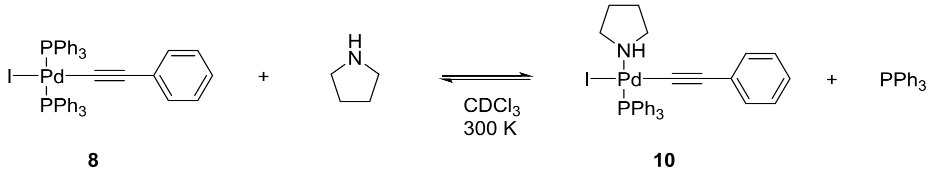

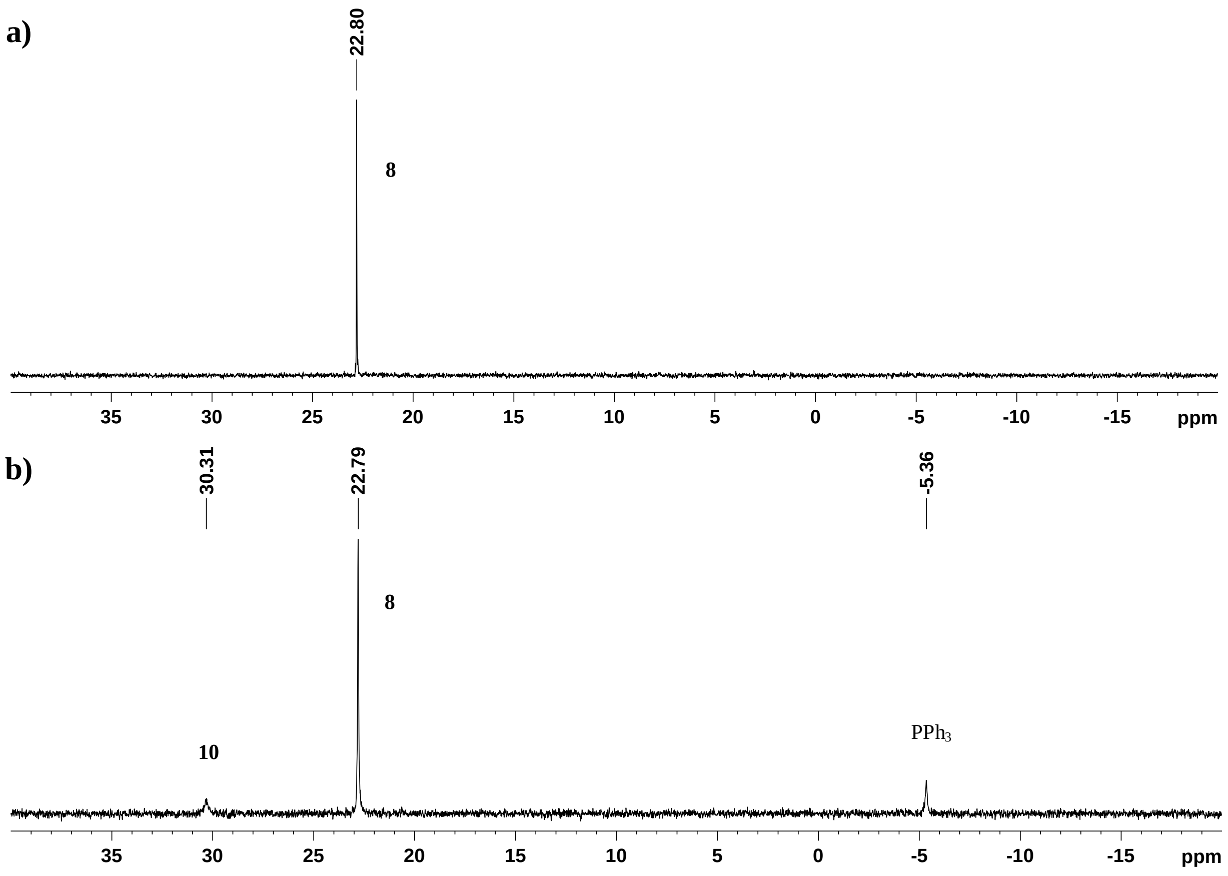

Supplementary Figure 14 | ^{31}P NMR spectrum of the solution of **8** (10.0 mg, 0.012 mmol) in $CDCl_3$ (0.70 mL). **a**, before the addition of pyrrolidine. **b**, after the addition of pyrrolidine (4 μL, 3.5 mg, 0.05 mmol, 4 equiv.).

The equilibrium constant was determined as an average form 3 independent experiment. The amount of each component (**8**, **10**, pyrrolidine, and triphenylphospine) in the equilibrium was determined by ^{1}H NMR integration (qNMR) using 1,3,5-trimethoxybenzene as internal standard. Since the resonances of triphenylphospine were overlapped with resonances of triphenylphospine parts from

compounds **8** and **10**, the concentration of triphenylphospine was postulated to be the same as the concentration of **10**. The starting amounts of **8** and pyrrolidine in each experiment (i-iii) are shown below.

$$K = \frac{[\mathbf{10}][\text{triphenylphosphine}]}{[\mathbf{8}][\text{pyrrolidine}]} \qquad \mathbf{(2)}$$

i. **8** (12.19 mg, 0.0142 mmol), pyrrolidine (4.67 mg, 0.065 mmol) in $CDCl_3$ (1.0 mL) at 300 K,
   K = 0.0242
ii. **8** (8.74 mg, 0.0102 mmol), pyrrolidine (8.87 mg, 0.123 mmol) in $CDCl_3$ (1.0 mL) at 300 K,
   K = 0.0214
iii. **8** (13.04 mg, 0.0152 mmol), pyrrolidine (19.80 mg, 0.278 mmol) in $CDCl_3$ (1.0 mL) at 300 K,
   K = 0.0358

K = 0.03 ($CDCl_3$, 300 K)

10:
^{1}H NMR (500 MHz, $CDCl_3$) δ 7.08–6.97 (m, 3H), 6.77–6.68 (m, 2H), 3.95 (br s, 1H), 3.56–3.21 (m, 4H), 2.01–1.77 (m, 4H), $P(Ph_3)$ resonances of **10** were overleaped with $P(Ph_3)$ resonances from other compounds in the mixture;
^{31}P NMR (202 MHz, $CDCl_3$) δ +30.3 (s);
HMRS (ESI+) (*m*/*z*): $[M + H]^+$ calcd for $C_{30}H_{30}INPPd^+$: 668.0190; found: 668.0206.

Supplementary Note 3. Transmetallation reactions

General procedure for palladium-palladium transmetallation

Oxidative adduct **4** (*trans*-**A**) and the source of acetylene (palladium bis-acetylide **6**, palladium mono-acetylide **8** or acetylene **2**) were let to react in solvent for given time. The reactions were monitored by NMR spectroscopy.

<u>Transmetallation between **4** (*trans*-**A**) and **6** (*trans*-**D**)</u>

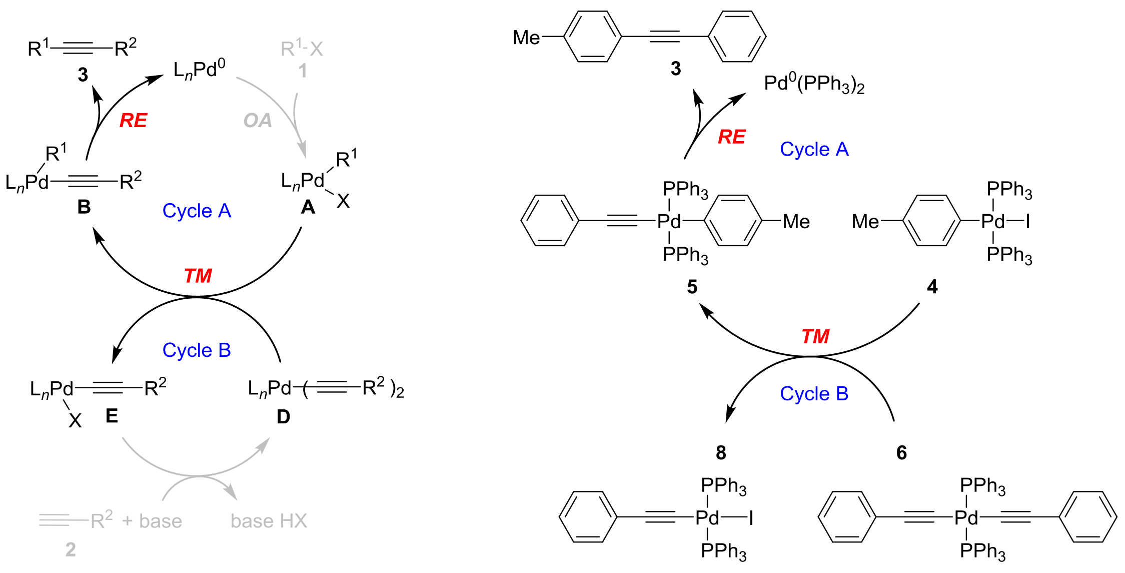

Transmetallation between 4 and 6 in $CDCl_3$ (Fig. 4)

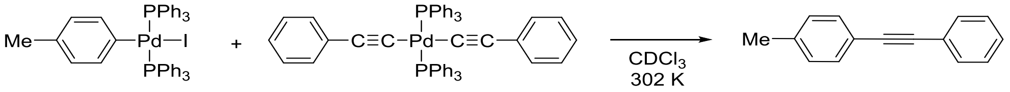

4 **6** **3**

A solution of **4** (8.05 mg, 0.0095 mmol) and **6** (9.48 mg, 0.0114 mmol, 1.2 equiv.) in $CDCl_3$ (0.70 mL) under argon atmosphere at 302 K was monitored by ^1H NMR (spectra were recorded in 5 min intervals, 1,3,5-trimethoxybenzene was used as internal standard). The formation of product **3** over time is presented in Fig. 4. The conversion into product **3** was determined from integrals of methyl resonances of **3** using 1,3,5-trimethoxybenzene as internal standard and are shown in Fig. 4b. The reaction was repeated 4-times, always returning consistent results.

The transmetallation between **4** and **6** in $CDCl_3$ is clean which is demonstrated by ^1H and ^{31}P NMR spectra in Supplementary Figs. 15 and 16. The reaction yields alkyne product **3**, transmetallation product **8** (*trans*-E), and $Pd^0(PPh_3)_2$ as a result of reductive elimination. After 2 h, side reactions start to occur (see Figures below).

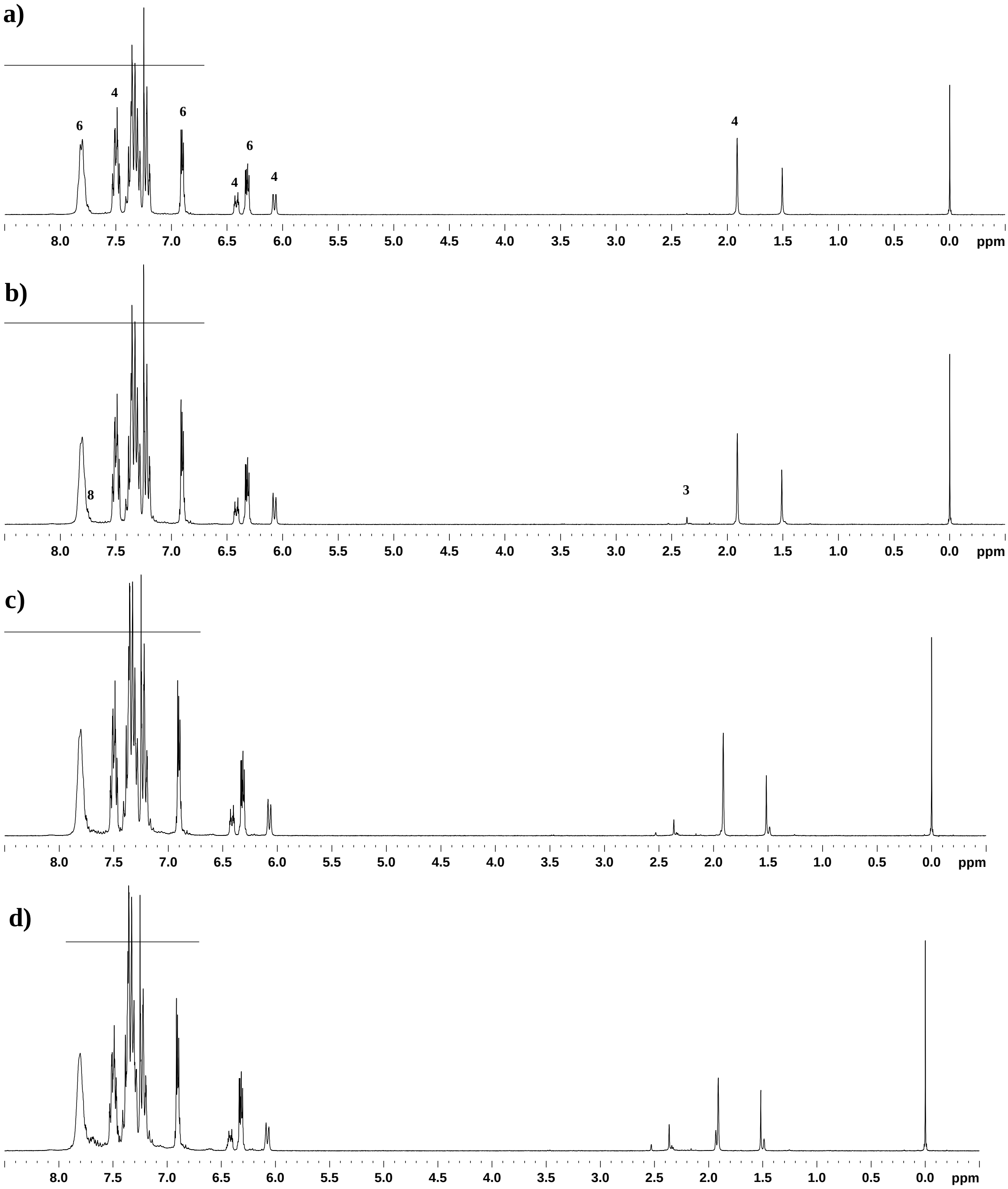

Supplementary Figure 15 | ^{31}H NMR spectra of reaction between **4** (7.5 mg, 0.0088 mmol) and **6** (8.2 mg, 0.0098 mmol) in $CDCl_3$ (0.7 mL). **a**, after 2 min. **b**, after 45 min. **c**, after 2 h. **d**, after 4 h.

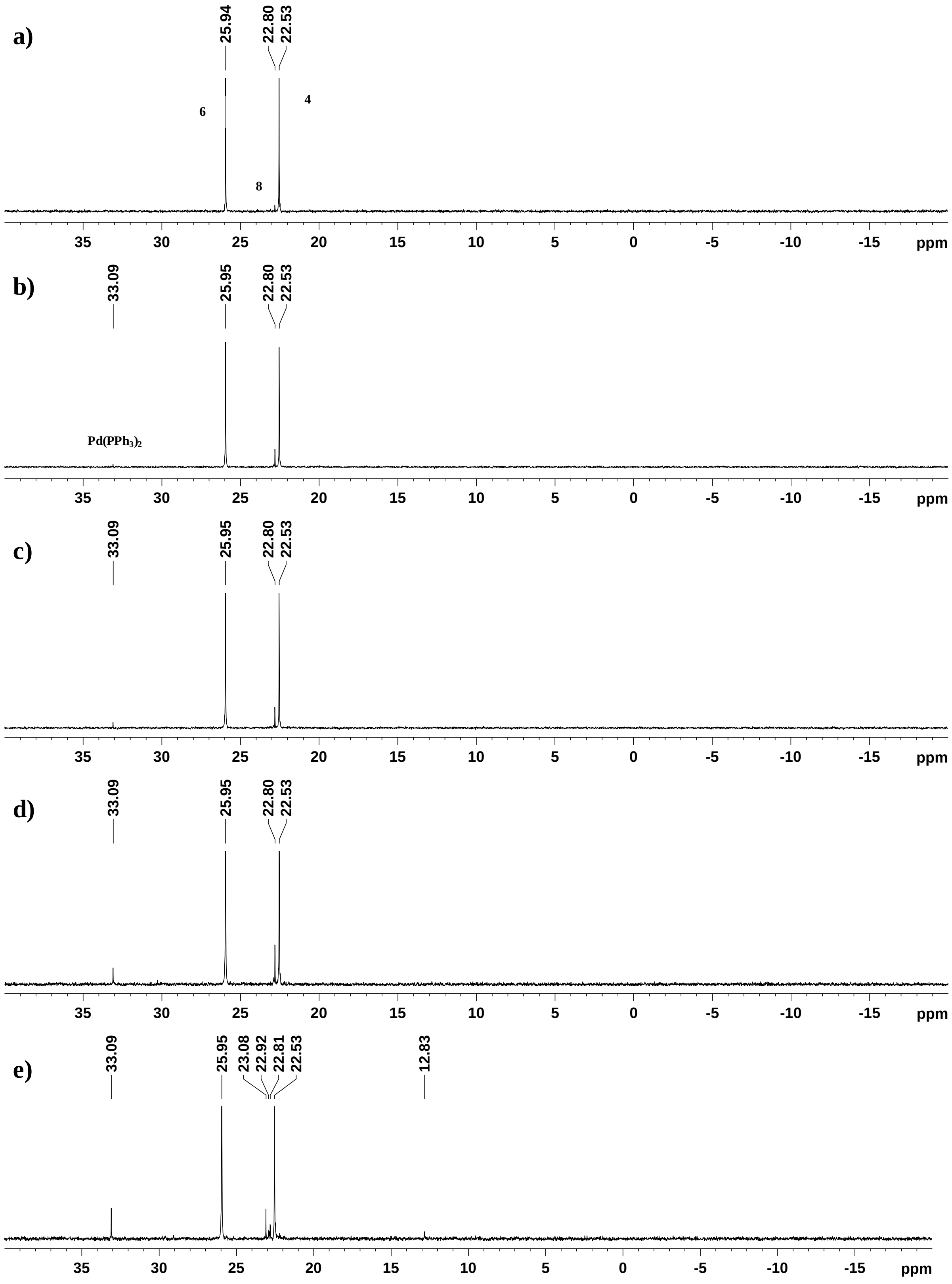

Supplementary Figure 16 | ^{31}P NMR spectra of reaction between **4** (7.0 mg, 0.0082 mmol) and **6** (6.9 mg, 0.0083 mmol) at 300 K in $CDCl_3$ (0.7 mL). **a**, after 5 min. **b**, after 30 min. **c**, after 1 h. **d**, after 2 h. **e**, after 4 h.

Transmetallation between **4** and **6** in the presence of pyrrolidine in $CDCl_3$ (Fig. 5)

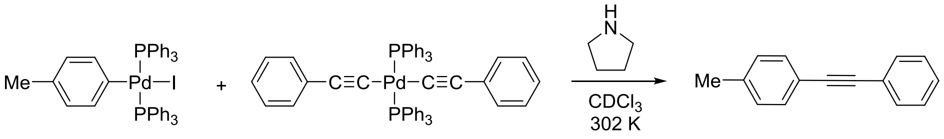

A solution of **4** (8.11 mg, 0.0095 mmol), **6** (9.65 mg, 0.0116 mmol, 1.2 equiv.) and pyrrolidine (4 µL, 3.4 mg, 0.0479 mmol, 5.0 equiv.) in $CDCl_3$ (0.70 mL) under argon atmosphere at 302 K was monitored by ^{1}H NMR (spectra were recorded in 5 min intervals, 1,3,5-trimethoxybenzene was used as internal standard). The formation of product **3** over time is presented in Fig. 5b. The conversion into product **3** was determined from integrals of methyl resonances of **3** using 1,3,5-trimethoxybenzene as internal standard and is shown in Fig. 5b. The reaction was repeated 3-times, always returning consistent results.

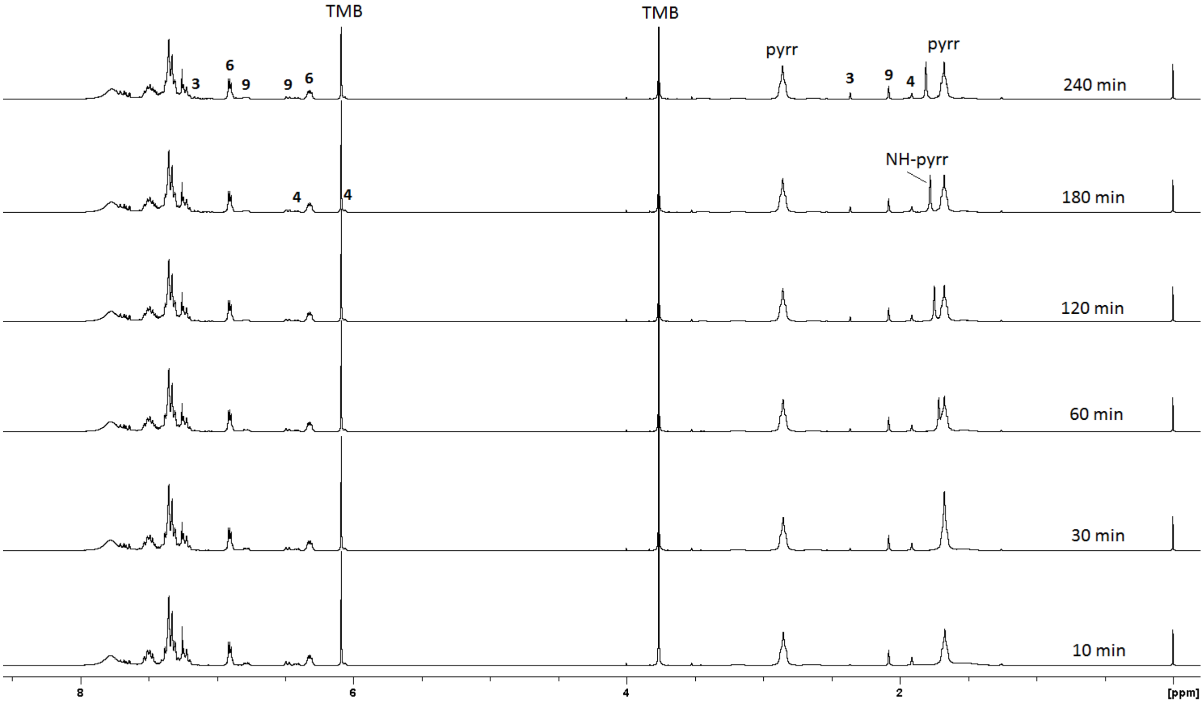

Supplementary Figure 17 | Stacking of ^{1}H NMR spectra over time of reaction between **4** and **2** in the presence of pyrrolidine. TMB = 1,3,5-trimethoxybenzene; **3** = 1-methyl-4-(phenylethynyl)benzene, pyrr = pyrrolidine. For structures of **4**, **6**, **9** please see Supplementary Table 1.

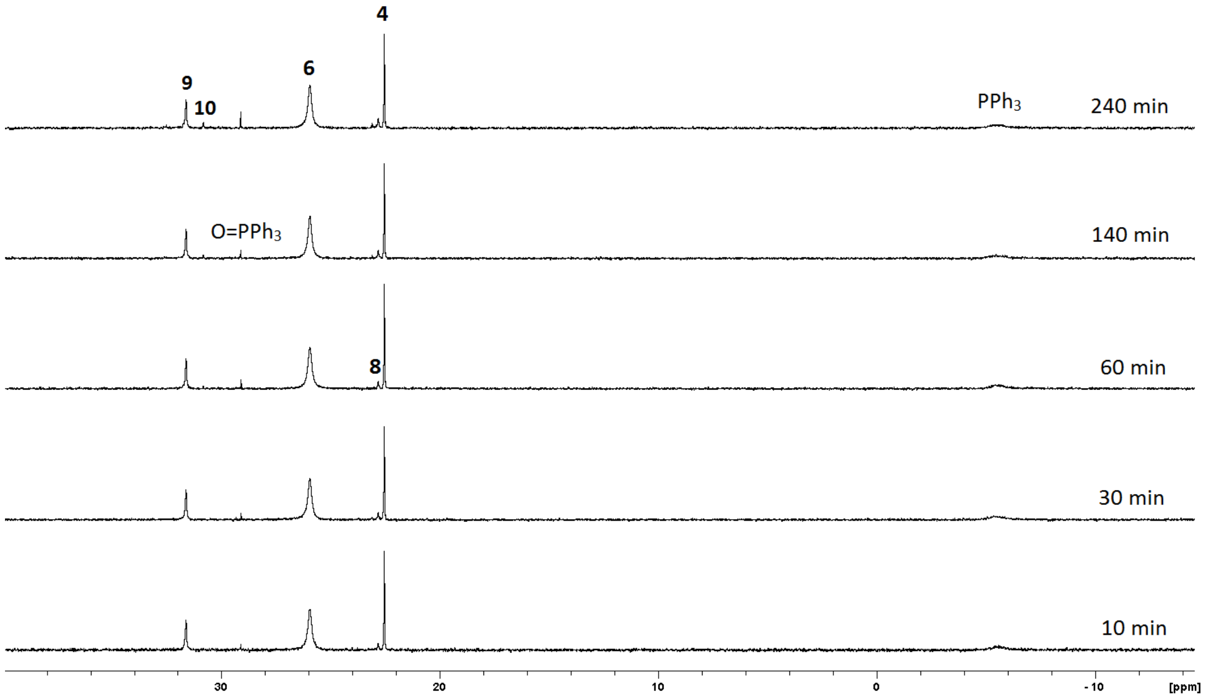

Supplementary Figure 18 | Stacking of ^{31}P NMR spectra over time of reaction between **4** and **6** in the presence of pyrrolidine. For structures of **4**, **6, 8**, **9** and **10** please see Supplementary Table 1. For the equilibrium between **4** and pyrrolidine, please see Supplementary Fig. 13; for the equilibrium between **8** and pyrrolidine, please see Supplementary Fig. 14; for fluxionality of **6** with PPh_3, please see Supplementary Fig. 23.

*Analysis of transmetallation between **4** and **6** in the presence of pyrrolidine in $CDCl_3$*

Concentration of product **3** at certain time was determined by integration of the signal for methyl protons of **3** and comparison with the internal standard. Plot for the concentration build-up of **3** is shown in Supplementary Fig. 19. To obtain the value of maximum rate, experimental data was fitted using Origin (OriginLab, Northampton, MA). Differentiation of the polynomial function and determination of maximum of first derivative yielded maximum reaction rate which is schematically presented as a dashed tangent line.

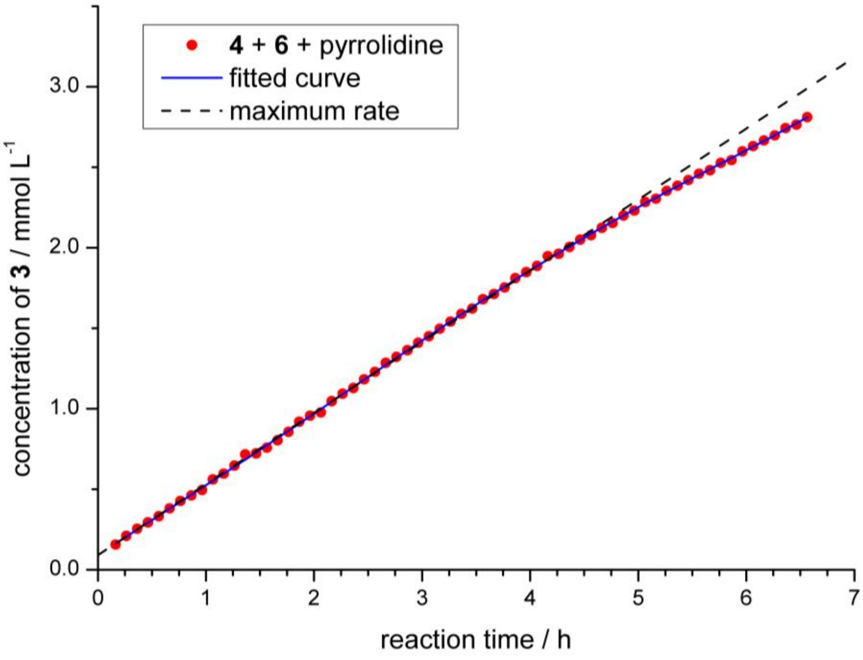

$$c(\mathbf{3}) = A_0 + A_1 \cdot t + A_2 \cdot t^2 + A_3 \cdot t^3 + A_4 \cdot t^4 + A_5 \cdot t^5$$

A_0	9.11 E-5	A_2	-5.39 E-9	A_4	-2.80 E-13	$R^2_{adj.} = 0.99988$
A_1	7.36 E-9	A_3	7.15 E-11	A_5	3.21 E-16	$r_{max} = \left(\frac{\partial c}{\partial t}\right)_{t=0} = 7.4 \cdot 10^{-6} \frac{\text{mol}}{\text{L min}}$

Supplementary Figure 19 | Concentration build-up of **3** in the reaction between **4** and **6** in the presence of pyrrolidine. Experimental data is fitted using polynomial regression and the function is displayed together with its maximum derivative (rate). Parameters are summarized underneath the plot.

The graph with combined kinetic plots for reactions between **4** and either **6** or **2** is in the Fig. 5b.

Transmetallation between **4** and **8** (Fig. 4)

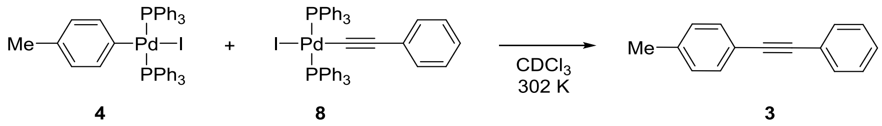

A solution of **4** (8.06 mg, 0.0095 mmol) and **8** (9.76 mg, 0.0114 mmol, 1.2 equiv.) in $CDCl_3$ (0.70 mL) under argon atmosphere at 302 K was monitored by ^{1}H NMR (spectra were recorded in 5 min intervals, 1,3,5-trimethoxybenzene was used as internal standard). Product formation **3** over time is presented in Fig. 4. The conversion into product **3** was determined from integrals of methyl resonances of **3** using 1,3,5-trimethoxybenzene as internal standard and is shown in Fig. 4b. The reaction was repeated 3-times, always returning consistent results.

Reactions of the oxidative adduct with acetylene

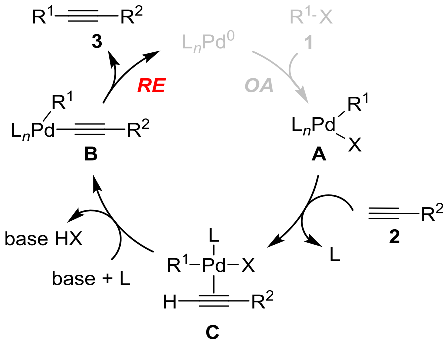

Reaction of **4** with **2** in the presence of pyrrolidine (Fig. 5)

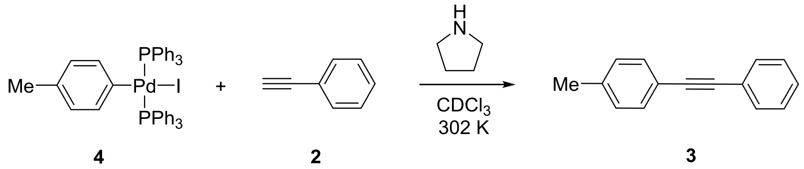

A solution of **4** (8.06 mg, 0.0095 mmol), **2** (1.20 mg, 0.0117 mmol, 1.2 equiv.; added as 0.700 mL of standard solution of **2** (7.16 mg) in $CDCl_3$ (4.18 mL)) and pyrrolidine (4 μL, 3.4 mg, 0.0479 mmol, 5.0 equiv.) under argon atmosphere at 302 K was monitored by ^{1}H NMR. Formation of the product **3** over time is presented in Fig. 5b. ^{1}H NMR spectra were recorded in 5 min intervals, using 1,3,5-trimethoxybenzene as internal standard). The conversion into product **3** was determined from integrals of

methyl protons of **3** using 1,3,5-trimethoxybenzene as internal standard and is shown in Fig. 5b. The reaction was repeated 3-times, always returning consistent results.

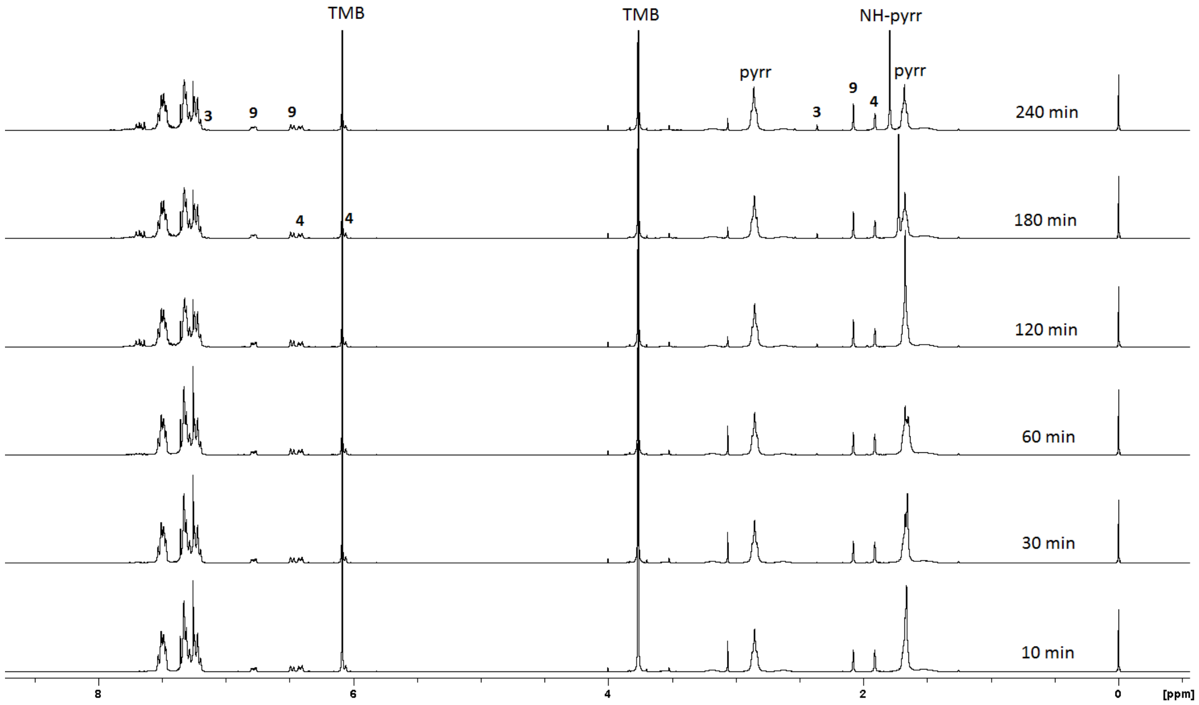

Supplementary Figure 20 | Stacking of ^{1}H NMR spectra over time of reaction between **4** and **2** in the presence of pyrrolidine. TMB = 1,3,5-trimethoxybenzene; **3** = 1-methyl-4-(phenylethynyl)benzene, pyrr = pyrrolidine; For structures of **4** and **9**, see Supplementary Table 1.

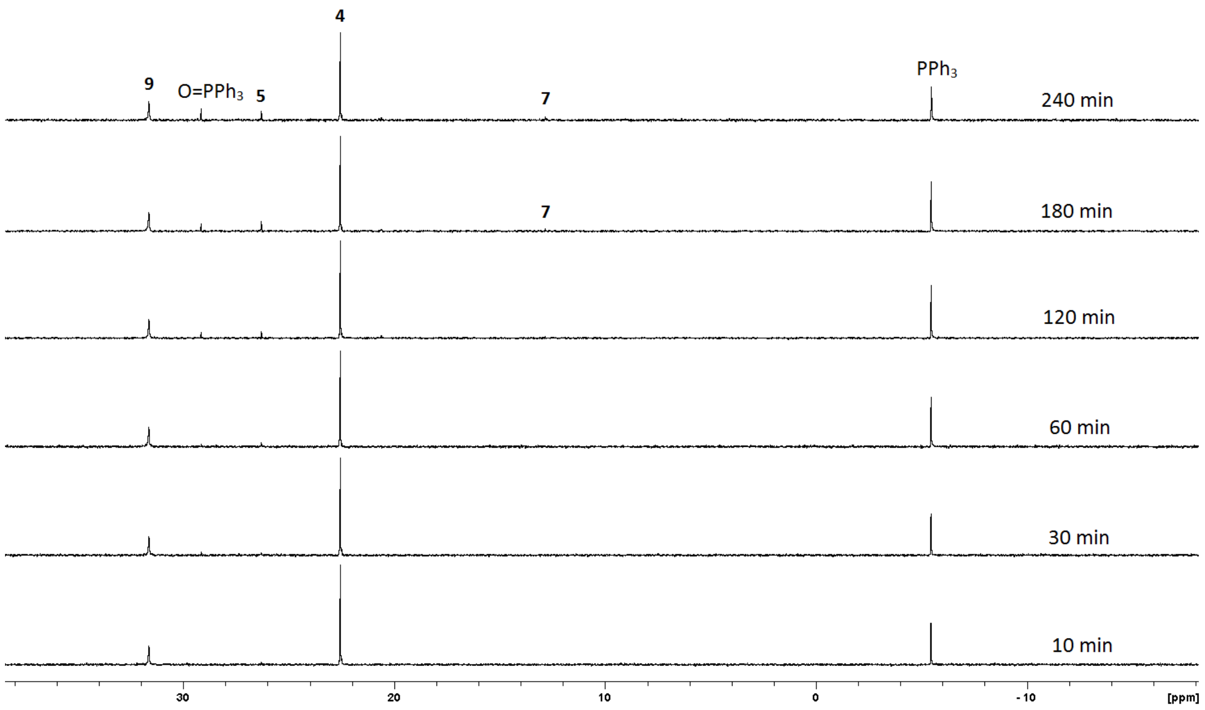

Supplementary Figure 21 | Stacking of ^{31}P NMR spectra over time of reaction between **4** and **2** in the presence of pyrrolidine. For structures of **4**, **5**, **7**, and **9**, see Supplementary Table 1.

*Analysis of the reaction between **4** and **2** in the presence of pyrrolidine in $CDCl_3$*

Concentration of product **3** at certain time was determined by integration of the signal for methyl protons of **3** and comparison with the internal standard. Plot for the concentration build-up of **3** is shown in Supplementary Fig. 22. To obtain the value of maximum rate, experimental data was fitted using Origin (OriginLab, Northampton, MA). Differentiation of the polynomial function and determination of maximum of derivative yielded maximum reaction rate, which is schematically presented as a dashed tangent line.

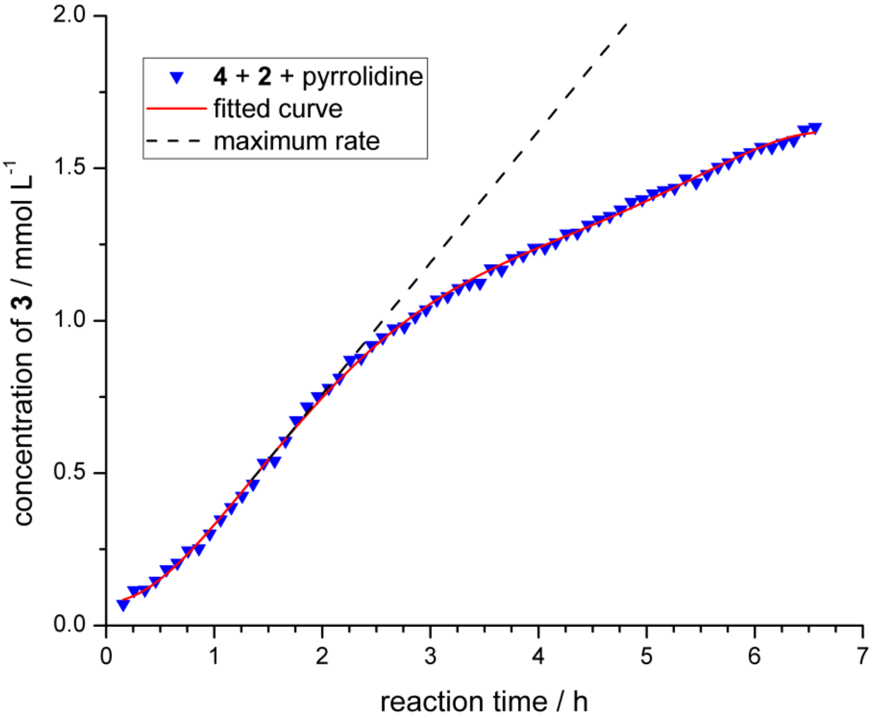

$$c(\mathbf{3}) = A_0 + A_1 \cdot t + A_2 \cdot t^2 + A_3 \cdot t^3 + A_4 \cdot t^4 + A_5 \cdot t^5$$

A_0	7.83 E-05	A_2	1.12 E-07	A_4	1.83 E-12	$R^2_{adj.} = 0.99922$
A_1	-3.01 E-07	A_3	-7.23 E-10	A_5	-1.66 E-15	$r_{max} = \left(\frac{\partial c}{\partial t}\right)_{t=80.9} = 7.2 \cdot 10^{-6} \frac{\text{mol}}{\text{L min}}$

Supplementary Figure 22 | Concentration build-up of **3** in the reaction between **4** and **2** in the presence of pyrrolidine. Experimental data is fitted using polynomial regression and the function is displayed together with its max. derivative (rate). Parameters are summarized underneath the plot.

Supplementary Note 4. Miscellaneous reactions

Fluxionality of **6** with triphenylphospine

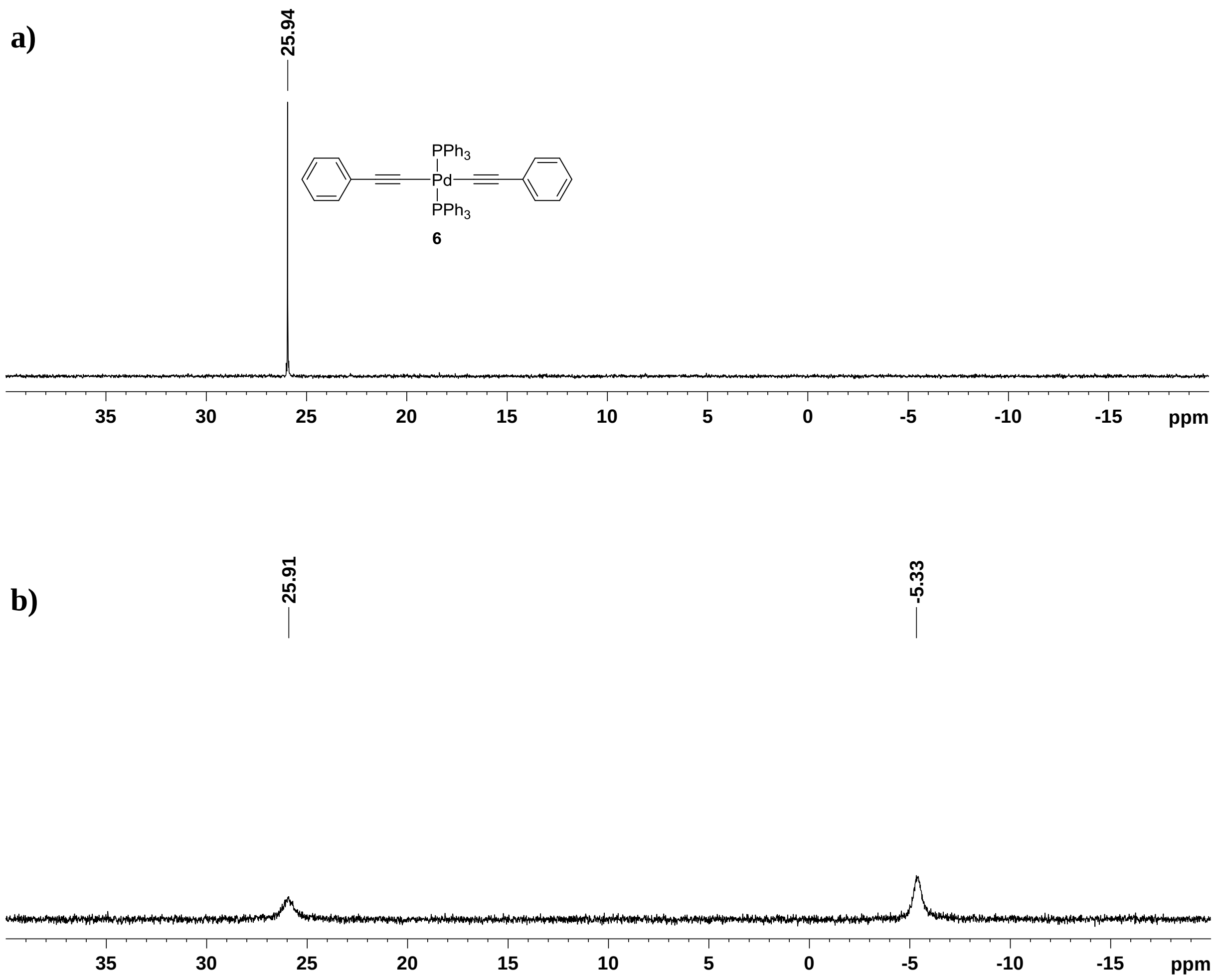

Supplementary Figure 23 | ^{31}P NMR spectrum of solution of **6** (6.0 mg, 0.0072 mmol) in $CDCl_3$ (0.70 mL). **a**, before the addition of triphenylphospine. **b**, after the addition of triphenylphospine (4.7 mg, 0.0180 mmol, 2.5 equiv.).

Formation of **7** from **4**

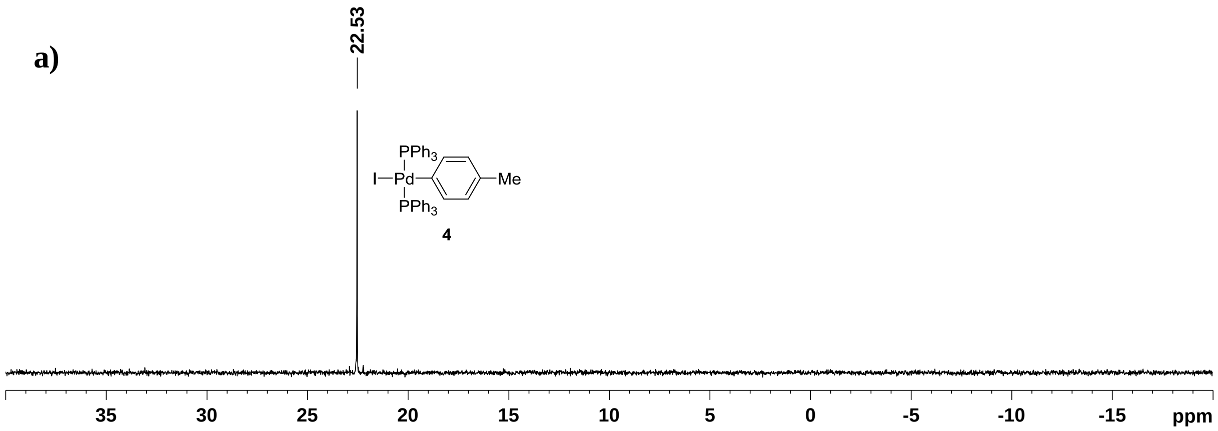

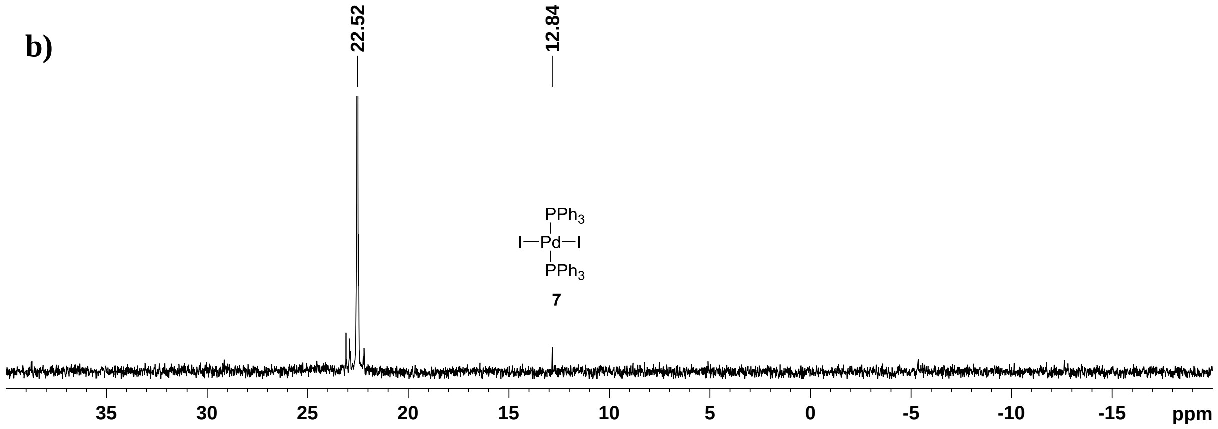

Supplementary Figure 24 | Concentrated solution of **4** in $CDCl_3$ (0.7 mL). **a**, after 2 min. **b**, after 15 min.

Oxidative addition of **1** to $Pd(PPh_3)_4$

To a solution of $Pd(PPh_3)_4$ (4 mg, 0.0034 mmol) in $CDCl_3$ (0.7 mL), 4-iodotoluene (**1**, 10 mg, 0.045 mmol; 13 equiv. relative to Pd) was added at room temperature under argon atmosphere, and ^{31}P NMR spectrum was recorded within 3 minutes. The ^{31}P NMR spectrum revealed the presence of oxidative adduct **4** along with $Pd(PPh_3)_2$, $O=PPh_3$ and PPh_3 (Supplementary Fig. 25a). The presence of Pd^0 species is clearly seen from the spectra, despite the fact that reaction mixture contains an excess of aryl iodide. Aging the above reaction mixture did not change the ratio between the phospine(Pd)-containing species (Supplementary Fig. 25b).

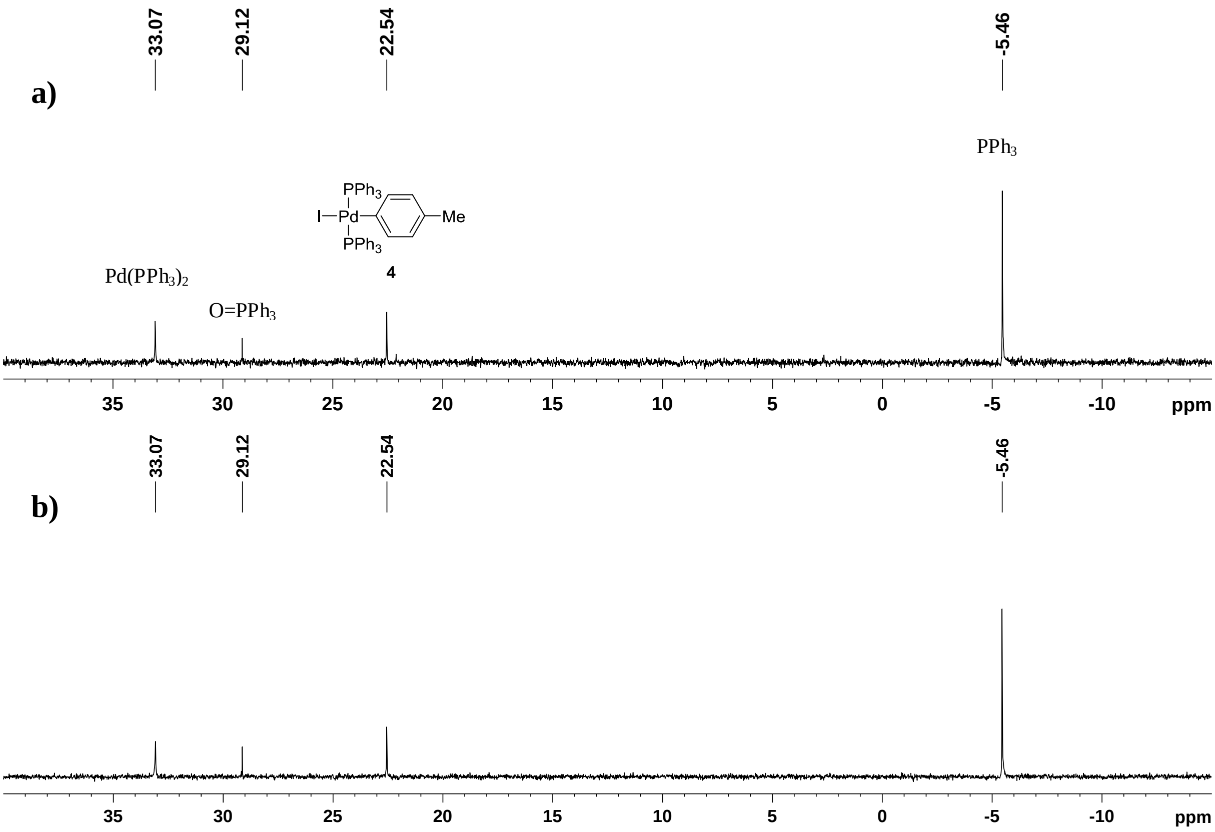

Supplementary Figure 25 | A solution of compound **1** and $Pd(PPh_3)_4$ in $CDCl_3$. **a,** after 3 minutes. **b**, after 15 minutes.

$Pd(PPh_3)_4$ dissolved in $CDCl_3$ before and after addition of PPh_3

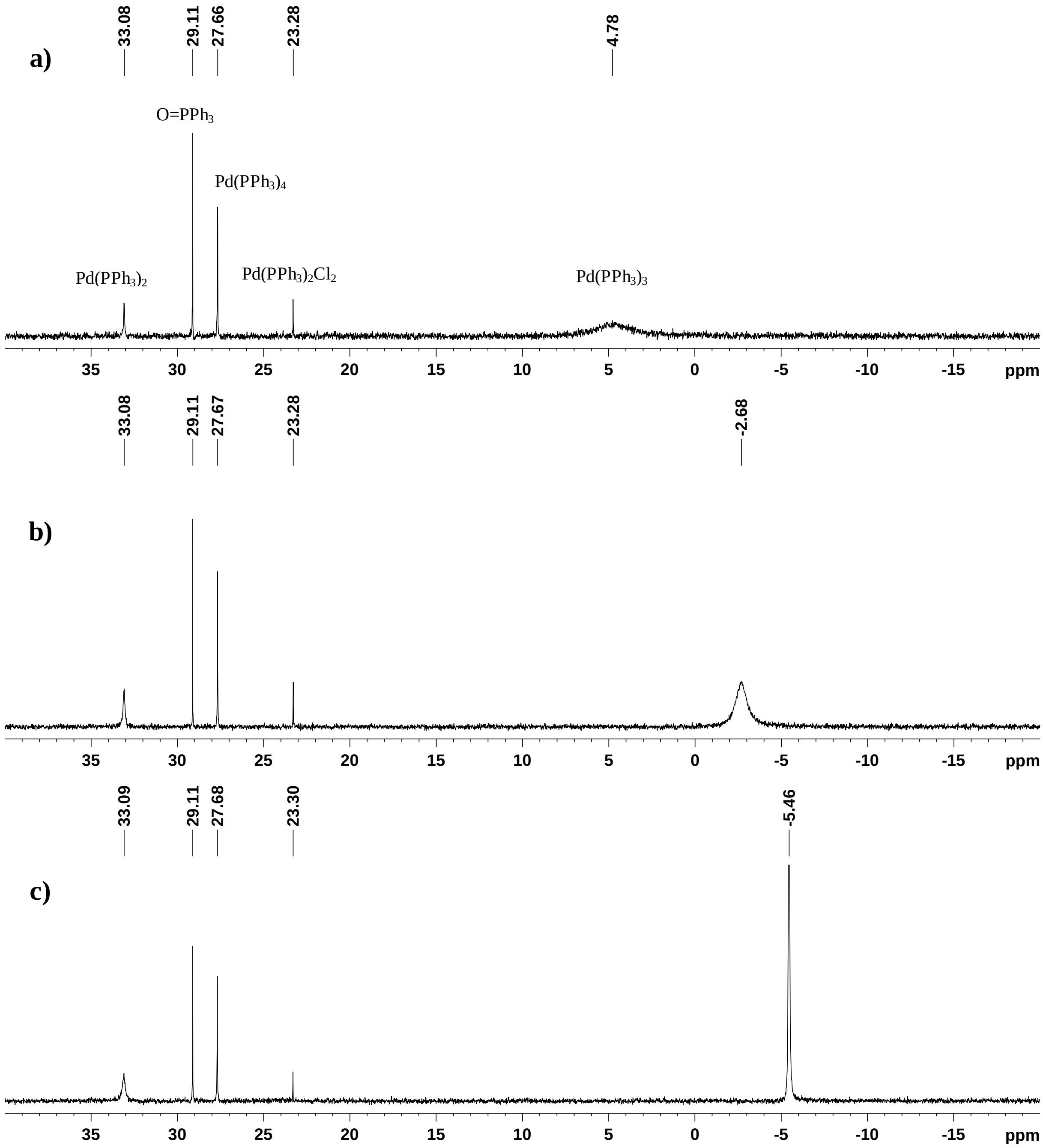

Supplementary Figure 26 | ^{31}P NMR spectrum of $[Pd^0(PPh_3)_4]$. ca. 10 mg dissolved in $CDCl_3$ (0.7 mL). **a**, before the addition of PPh_3, **b**, after the addition of minor (ca. 1 mg) amount of PPh_3, **c**, after the addition of considerable (ca. 10 mg) amount of PPh_3.

Resonance at +27.7 ppm belongs to $Pd(PPh_3)_4$, which is in agreement with the literature reported value (+27.4 ppm)[16]. The broad resonance at +4.8 ppm is ascribed to $Pd(PPh_3)_3$. This has been confirmed by adding PPh_3 to the solution of $Pd(PPh_3)_4$,

shifting the resonance at +4.8 ppm closer to –5 ppm. This observation is in agreement with the literature report[7]. Resonance at +23.3 correspond to $Pd(PPh_3)_2Cl_2$, precursor for the synthesis of $Pd(PPh_3)_4$, which is present in the commercially available material from one of the suppliers, and was assigned by comparison with the authentic sample and is in complete agreement with literature reported value (+23.9)[17].

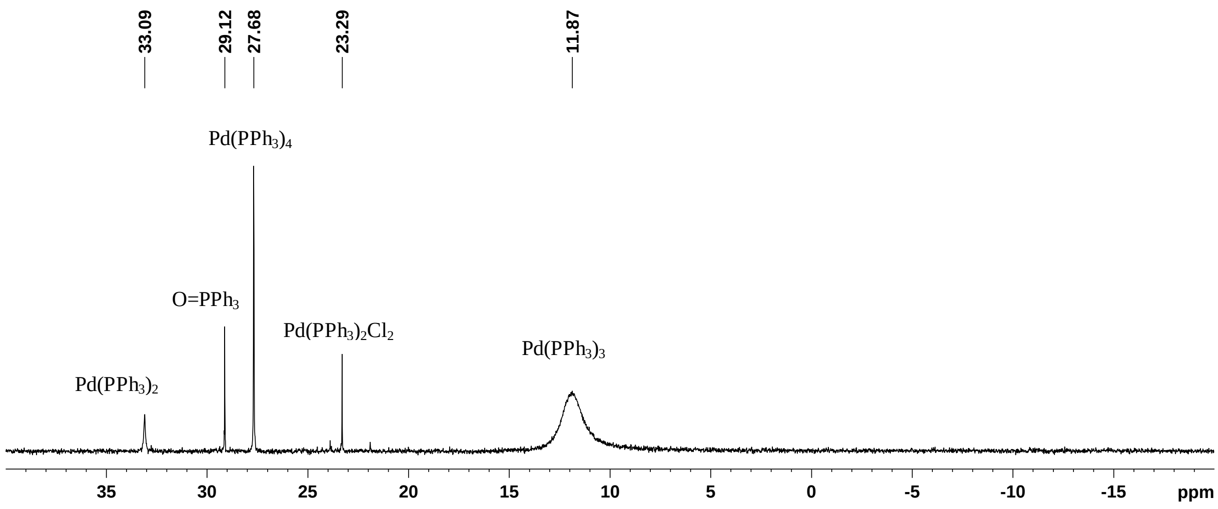

Supplementary Figure 27 | ^{31}P NMR spectrum of $[Pd^0(PPh_3)_4]$ from the same supplier as in Supplementary Fig. 26, but different batch number. ca. 10 mg dissolved in $CDCl_3$ (0.7 mL).

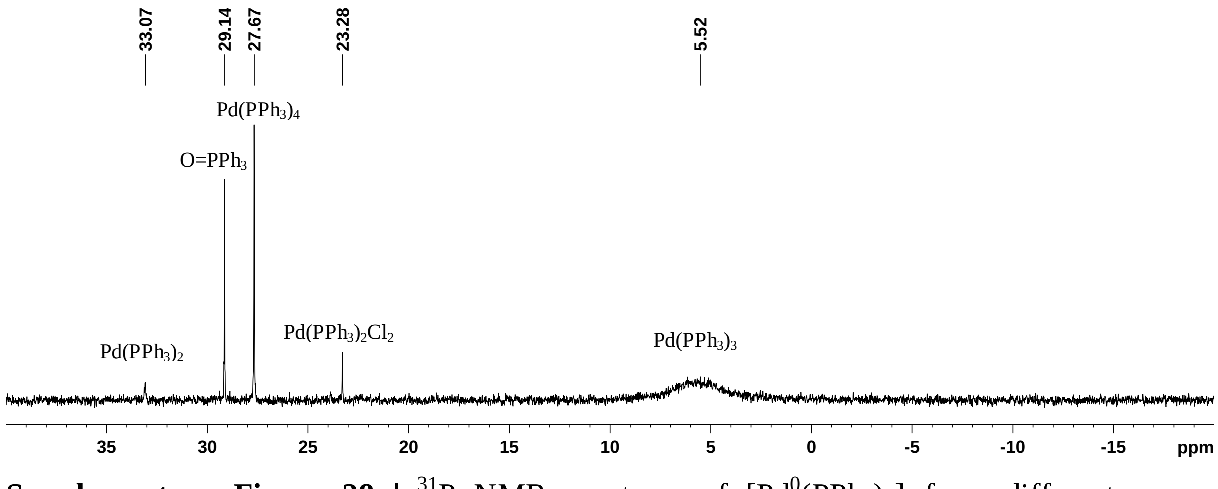

Supplementary Figure 28 | ^{31}P NMR spectrum of $[Pd^0(PPh_3)_4]$ from different supplier as in the case of Supplementary Fig. 27. ca. 10 mg dissolved in $CDCl_3$ (0.7 mL).

Note: As evident from Supplementary Figs. 26–28 (the ^{31}P NMR spectra were recorded for freshly opened originally sealed chemicals, under argon atmosphere, and

by using degassed $CDCl_3$) the quality of commercially available $Pd(PPh_3)_4$ may slightly vary from provider to provider, from batch to batch. It is thus critical for obtaining reliable results to conduct kinetic experiments consistently with the material from the same batch.

Supplementary Note 5. Kinetic investigation

In this kinetic study, reactions were performed under the reaction conditions for *Reaction a*, but with varying loadings of $Pd(PPh_3)_4$, namely 3, 4 and 5 mol%. Product **3** build up over time was monitored by using ^{1}H NMR spectroscopy.

Due to lower sampling frequency (the experimental mixture is heterogeneous and direct monitoring is impossible), the obtained data cannot be satisfactorily fitted using polynomial curves. Polynomial fit can produce unrealistic parts of the curve due to the lack of points in some of the segments along the reaction coordinate. It is best described by using sigmoid functions, which better describe the growth in natural systems, such as Gompertz function represented in Supplementary Equation 3.

$$c(t) = a \cdot e^{-e^{-k(x-x_c)}} \qquad \textbf{(3)}$$

To find the maximum rate of the reaction, it was necessary to determine the highest value of the first derivative of such a function

$$r(t) = \frac{\partial c}{\partial t} = a\,k \cdot e^{-e^{-b(k-x_c)}-k(x-x_c)} \qquad , \qquad \textbf{(4)}$$

which can be found based on the second derivative:

$$\frac{\partial^2 c}{\partial t^2} = a\,k \cdot \left(k\,e^{-k(x-x_c)} - k\right) \cdot e^{-e^{-k(x-x_c)}-k(x-x_c)} \qquad . \qquad \textbf{(5)}$$

Analytical solution to the equation $\left(\frac{\partial^2 c}{\partial t^2}\right) = 0$ corresponds to t = c, hence

$$r_{max} = r(c) = a\,k\,e^{-1}$$

Experimental procedure

To a mixture of 4-iodotoluene (**1**, 545 mg, 2.5 mmol), phenylacetylene (**2**, 281 mg, 2.75 mmol), sodium methoxide (149 mg, 2.75 mmol) and dry *N*,*N*-dimethylformamide (5 mL), $Pd(PPh_3)_4$ (87 mg, 0.075 mmol, 3 mol% of Pd or 116 mg, 0.10 mmol, 4 mol% of Pd or 145 mg, 0.125 mmol, 5 mol% of Pd) was added under argon atmosphere at room temperature (1,3,5-trimethoxybenzene was added as internal standard). Stirring was continued at room temperature. After given time an aliquot (50 μL) was directly diluted with deuterated chloroform (0.6 mL), transferred into NMR tube, and ^{1}H and ^{31}P NMR were recorded immediately. It has been confirmed that this workup completely stops the reaction by re-acquiring the ^{1}H NMR spectrum of the same sample after being aged in the NMR tube for 1 h, with the same result. The conversion into product **3** was determined by qNMR from integrals of methyl protons of product **3** using 1,3,5-trimethoxybenzene as internal standard and is shown in Supplementary Figs. 29–31. The reaction was repeated twice, returning consistent results.

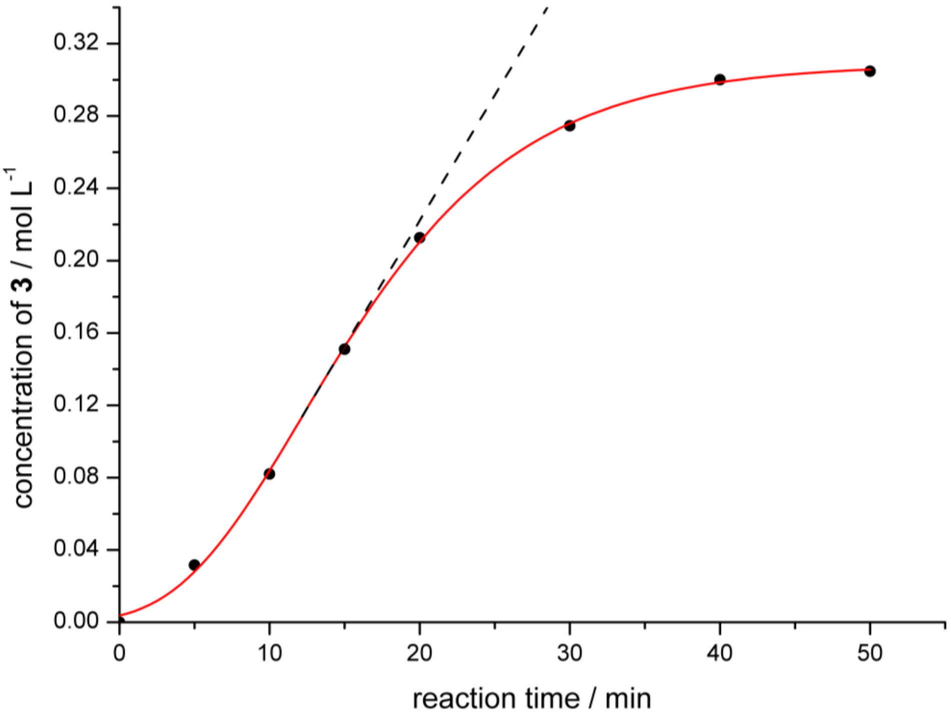

$$c(\mathbf{3}) = a \cdot e^{-e^{-k(x-x_c)}}$$

a = 0.31 k = 0.122 x_c = 12.2

$$R^2_{adj.} = 0.99944 \qquad r_{max} = \left(\frac{\partial c}{\partial t}\right)_{t=x_c} = 1.4 \cdot 10^{-2} \frac{\text{mol}}{\text{L min}}$$

Supplementary Figure 29 | Experimental data and fitted sigmoid curve for the reaction with 3 mol% of $Pd(PPh_3)_4$ with displayed maximum reaction rate and the corresponding parameters.

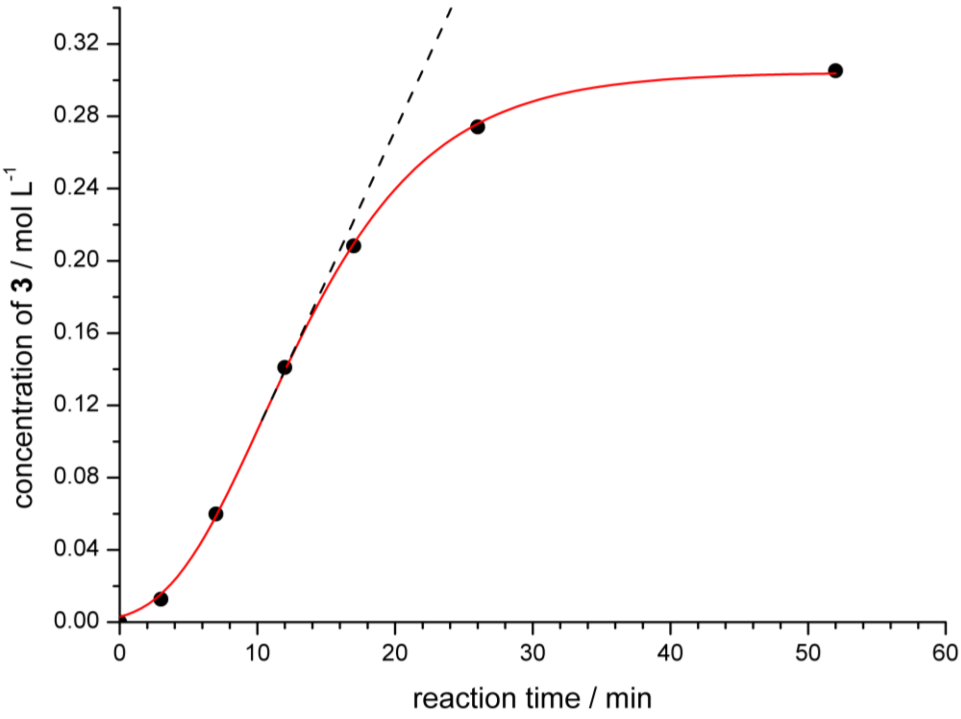

$$c(\mathbf{3}) = a \cdot e^{-e^{-k(x-x_c)}}$$

a = 0.30 k = 0.148 x_c = 10.4

$R^2_{adj.} = 0.99954$ $r_{max} = \left(\frac{\partial c}{\partial t}\right)_{t=x_c} = 1.7 \cdot 10^{-2} \frac{\text{mol}}{\text{L min}}$

Supplementary Figure 30 | Experimental data and fitted sigmoid curve for the reaction with 4 mol% of $Pd(PPh_3)_4$ with displayed maximum reaction rate and the corresponding parameters.

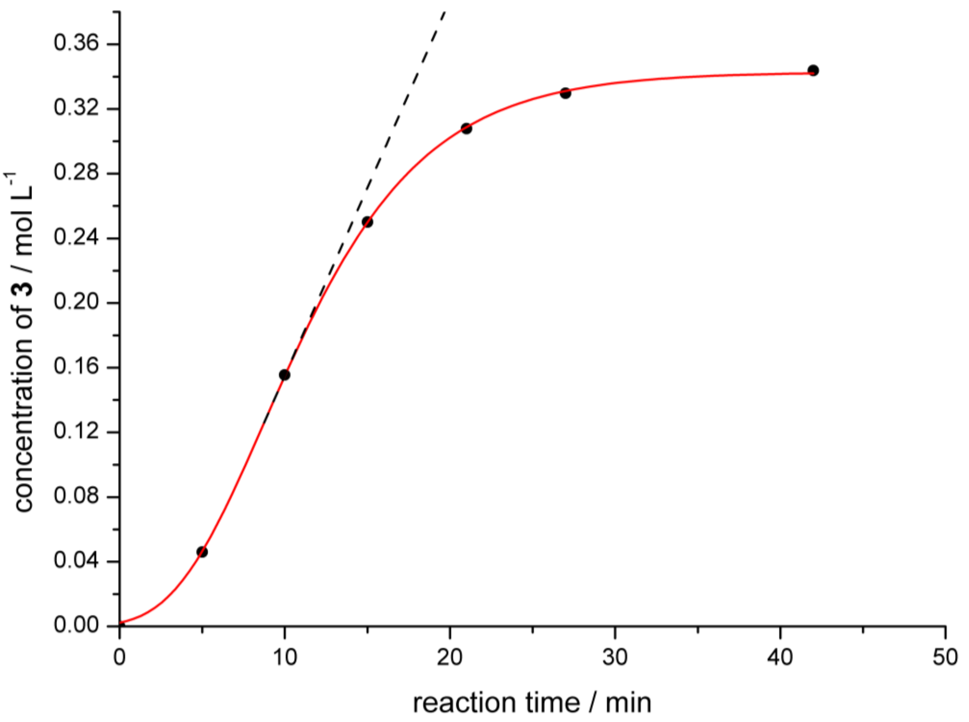

$$c(\mathbf{3}) = a \cdot e^{-e^{-k(x-x_c)}}$$

a = 10.34 k = 0.184 x_c = 8.76

$$R^2_{adj.} = 0.99985 \qquad r_{max} = \left(\frac{\partial c}{\partial t}\right)_{t=x_c} = 2.3 \cdot 10^{-2}\,\frac{\text{mol}}{\text{L min}}$$

Supplementary Figure 31 | Experimental data and fitted sigmoid curve for the reaction with 5 mol% of $Pd(PPh_3)_4$ with displayed maximum reaction rate and the corresponding parameters.

Parameters of the reactions with 3 to 5 mol% are summarized in the Supplementary Table 2.

The obtained maxima of the reaction rates were plotted against the concentrations of palladium to estimate the reaction order. A log-log graph from Supplementary Fig. 32 returned tentative order in palladium to be 1.

Supplementary Table 2 | Kinetic parameters for the investigated reactions in determination of order in Pd.

Loading of Pd / mol%	a	k	x_c	10^2 r_{max}	ln(r_{max})	ln[Pd]
3	1.55	0.122	12.2	1.6	-4.3	-7.6
4	1.52	0.148	10.4	1.7	-4.1	-7.3
5	1.71	0.184	8.76	2.3	-3.8	-7.1

Comment: It is interesting to note that parameter x_c actually corresponds to the time during which the reaction is gaining in rate, i.e. induction time. With the increasing loadings of palladium, this time tends to get shorter and can become unnoticeable based on the limitations of the monitoring method.

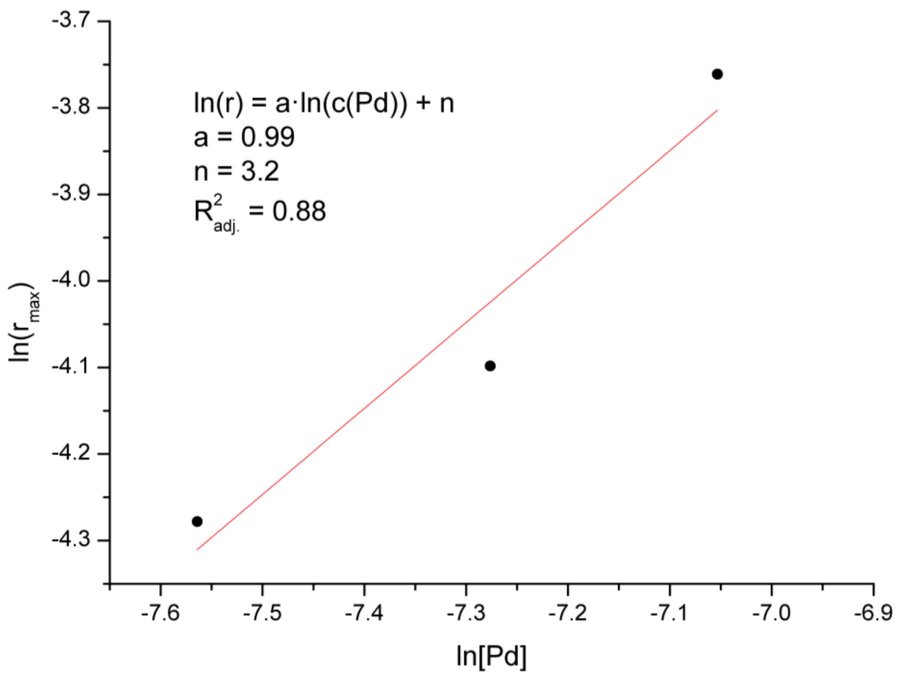

Supplementary Figure 32 | Graph for determination of order in Pd for *Reaction a* under the investigated conditions and corresponding parameters.

Supplementary Note 6. Computational Investigations

Additional comments on the transmetallation process

Supplementary Fig. 33 portrays an association-based transmetallation process for Sonogashira cross-couplings that we revealed computationally. Namely, in this mechanism the PPh_3 ligand does not dissociate at the onset of the reaction and, accordingly, **4** and **6** form an adduct through an associative ligand-exchange mechanism extruding I^-. Similarly, in the last step the incoming iodide supposedly liberates the separated products, ***trans*-5** and **8**, via an associative ligand exchange process. While the central acetylide ligand migration is predicted to be a kinetically feasible process, the association of **4** and **6** through a *tbp* transition state is associated with an activation barrier of 32.9 kcal mol^{-1}, which is prohibitive under the experimental conditions. In spite of intensive efforts we could not optimize the transition state leading to the separated products.

Supplementary Fig. 33 also shows the activation of phenylacetylene (**2**) by **4** through an associative mechanism alternative to the dissociative mechanism discussed in the manuscript. First, we assumed the replacement of iodide to a coordinating base, pyrrolidine, to yield **4base**. The activation of phenylacetylene takes place through an associative ligand exchange mechanism, in which the base is replaced to phenylacetyilydene traversing $\mathbf{TS}^{\mathbf{4base+2}}$ with relative energy of 29.4 kcal mol^{-1} to form **15**. As of **15** the reaction is identical to the one discussed in the manuscript. Similarly to the above presented transmetallation process, the dissociative mechanism discussed in the manuscript is also a lower energy pathway than this associative mechanism for the direct activation of phenylacetylene.

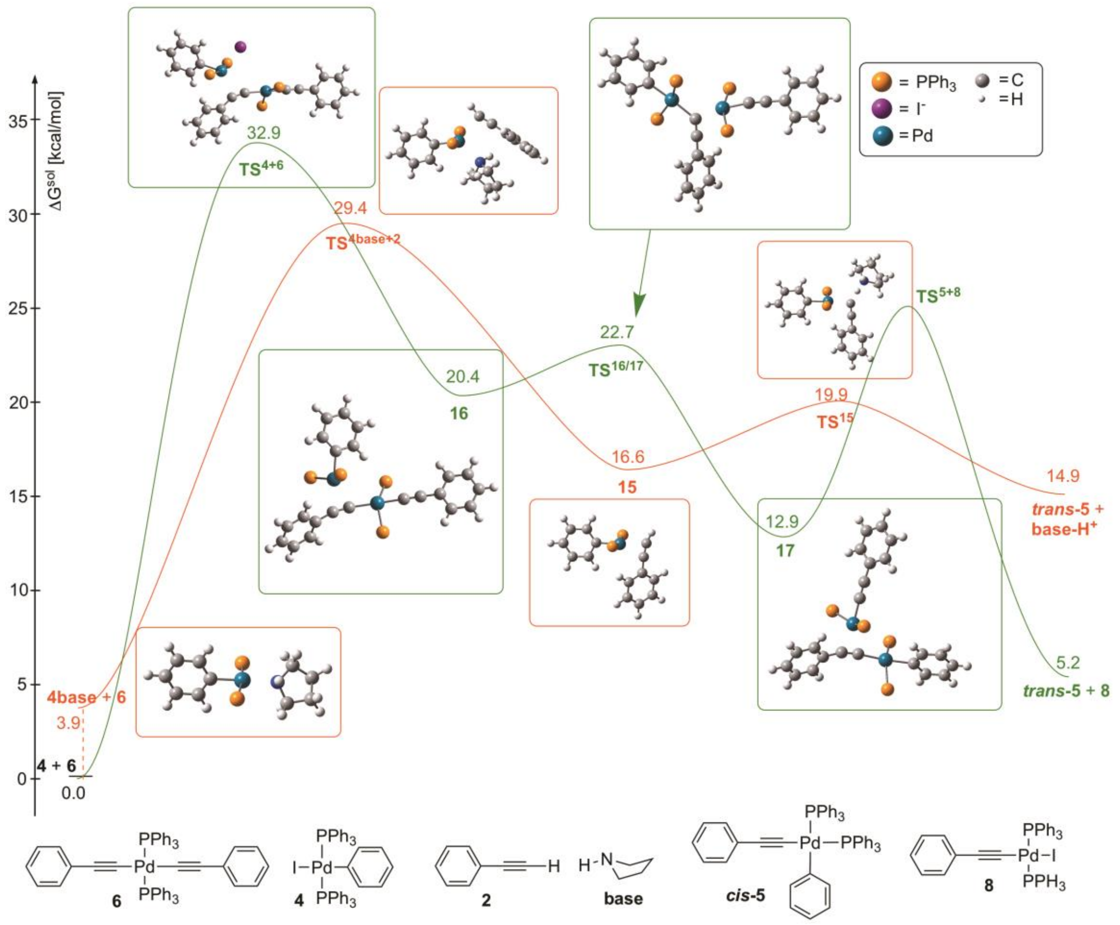

Supplementary Figure 33 | Computed reaction profiles for the transmetallation and phenylacetylene activation process initiated by associative ligand exchange mechanism.

The activation of phenylacetylene starting with the dissociation of phosphine from **4**, i.e. by active species **4-PPh3** is given in Supplementary Fig. 34. The reaction path is similar to that of **4-I** and **2** (Supplementary Fig. 33), the stability of deprotonation TS and product being the main differences. Namely, our calculations predict that forming the anionic species Pd $[Pd(Ph)(C_2Ph)(PPh_3)I]^-$ (**19**) and concomitantly **base-H**$^+$ in the case of **4-PPh3** is less favorable (20.1 kcal mol^{-1}) than yielding neutral ***trans*-5** (14.9 kcal mol^{-1}) in the case of **4-I** and this stability difference already manifests in the corresponding TSs, **TS^{15}** (19.9 kcal mol^{-1}) and **TS^{18}** (24.2 kcal mol^{-1}). Accordingly, we think that the activation of **2** is more plausible through the formation of **4-I** than via **4-PPh3**.

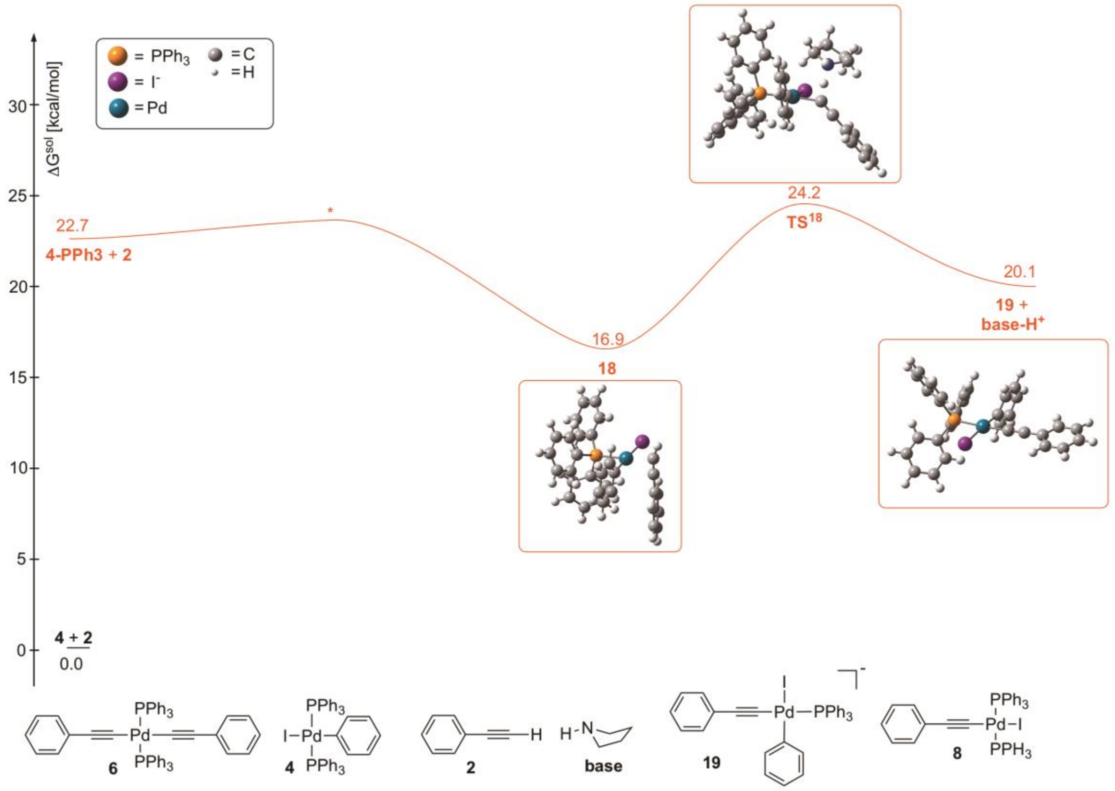

Supplementary Figure 34 | Computed reaction profile and solution-state Gibbs free energies of stationary points for the phosphine dissociation initiated activation of phenylacetylene.

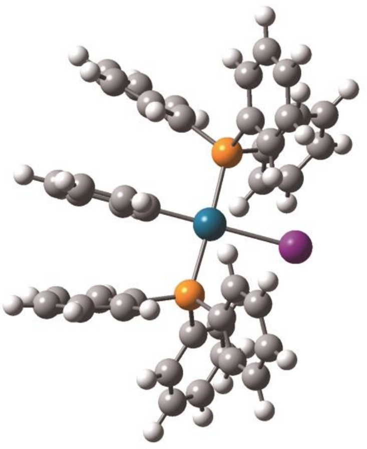

4

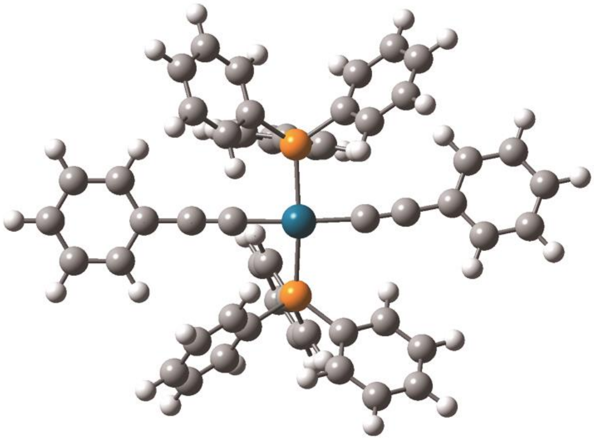

6

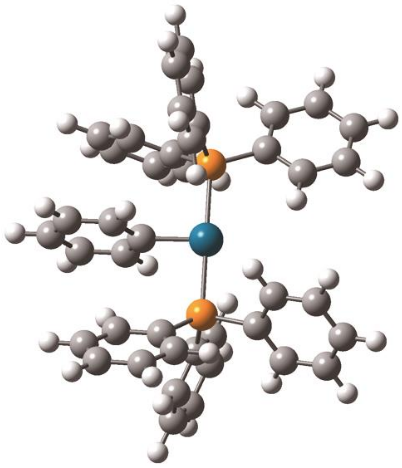

4-I

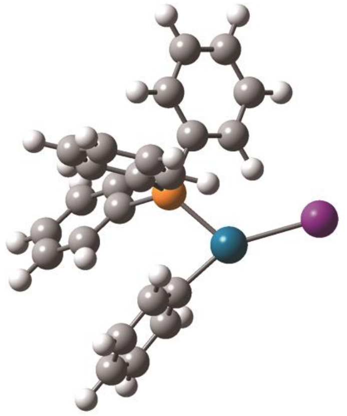

4-PPh3

Supplementary Figure 35 | Equilibrium structure of **4**, **6**, **4-I** and **4-PPh3**.

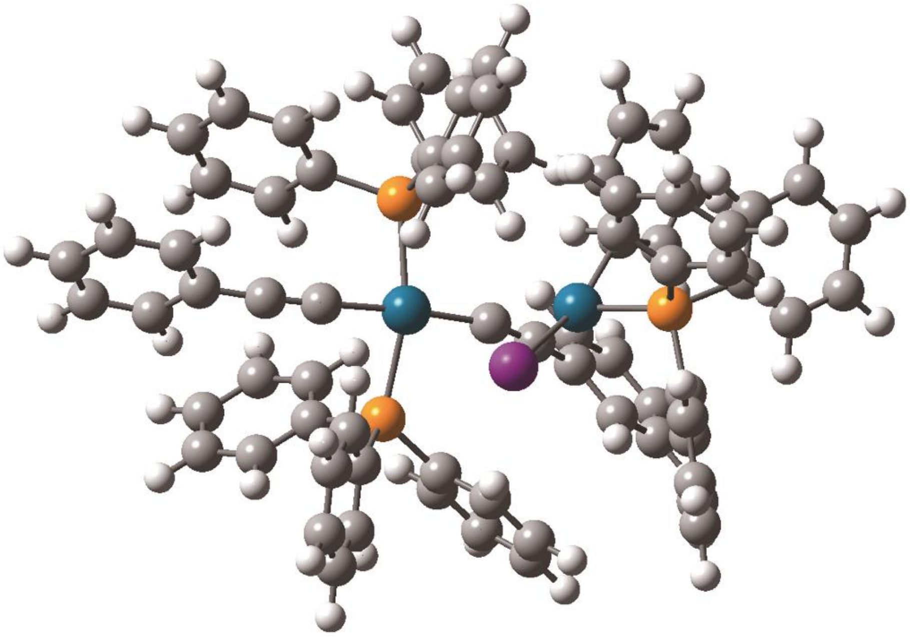

11

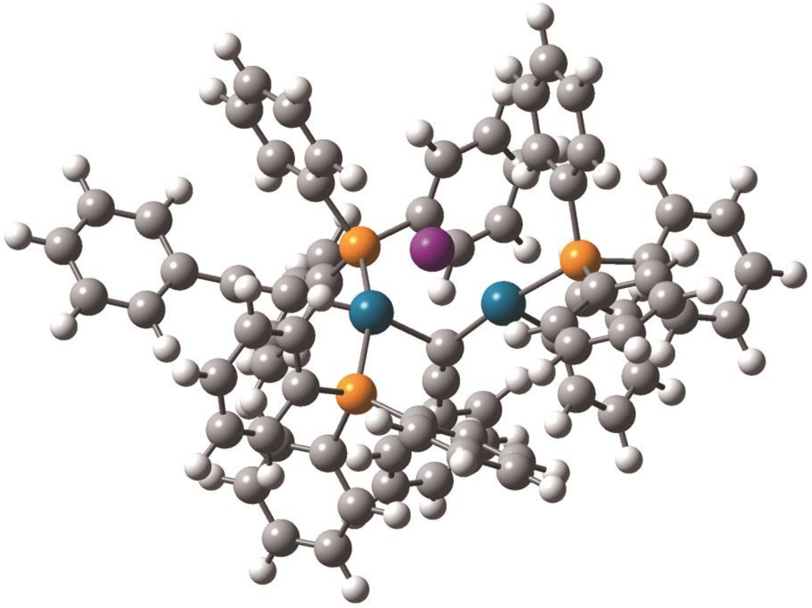

12

Supplementary Figure 36 | Equilibrium structure of **11** and **12.**

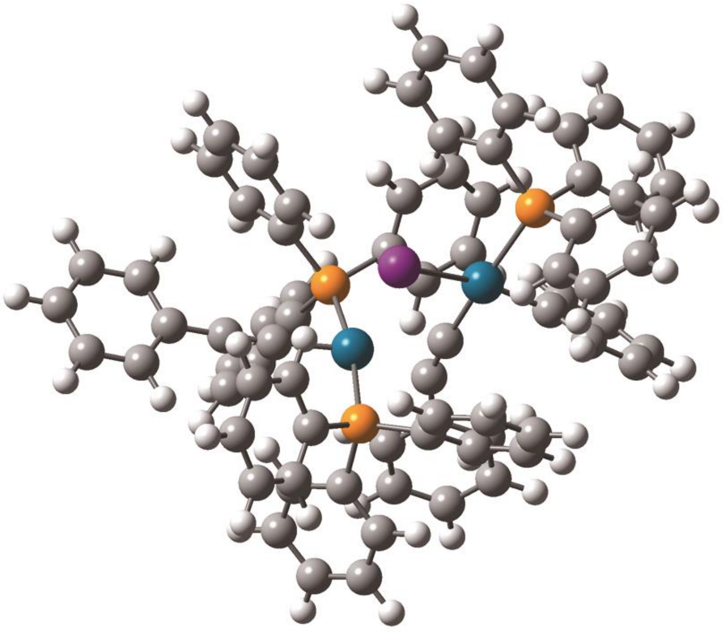

13

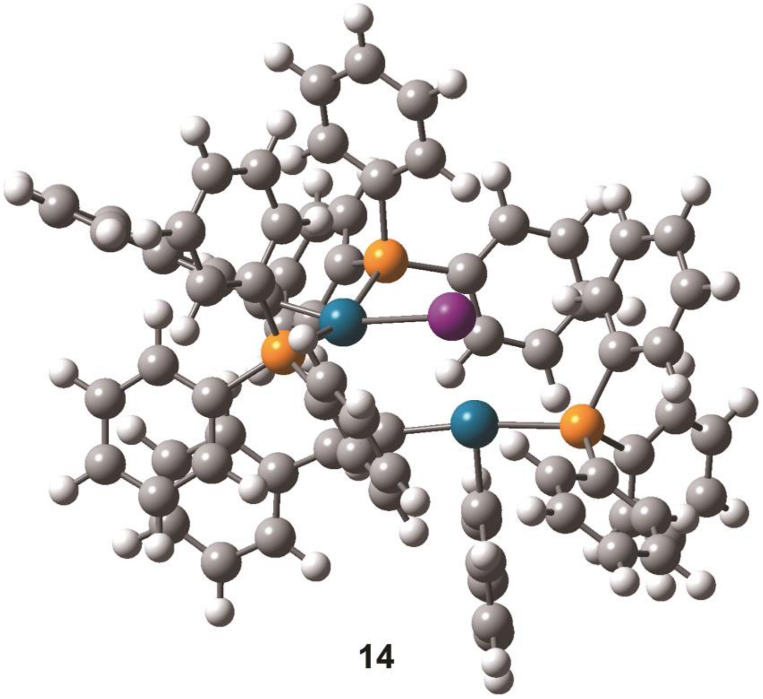

14

Supplementary Figure 37 | Equilibrium structure of **13** and **14**.

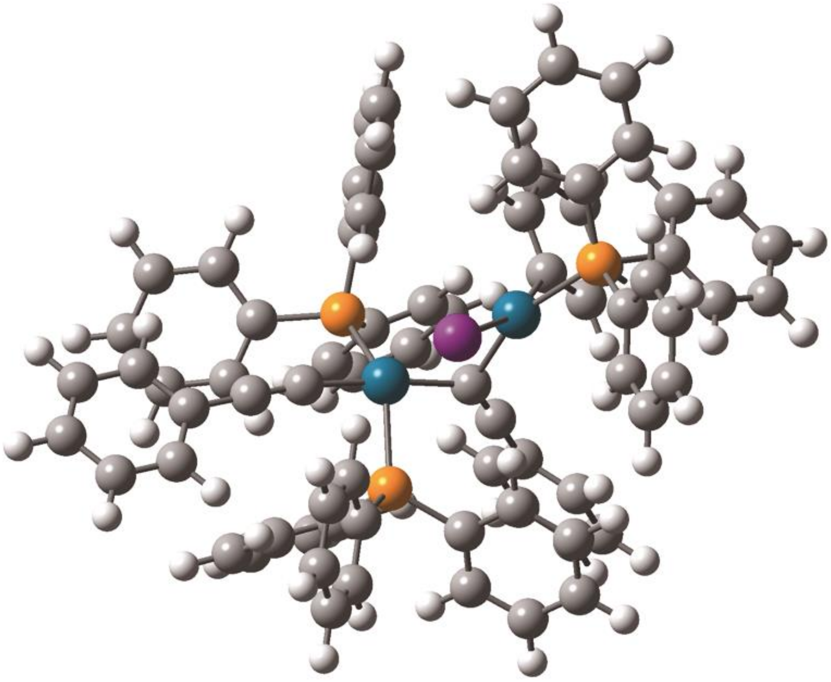

TS$^{11/12}$

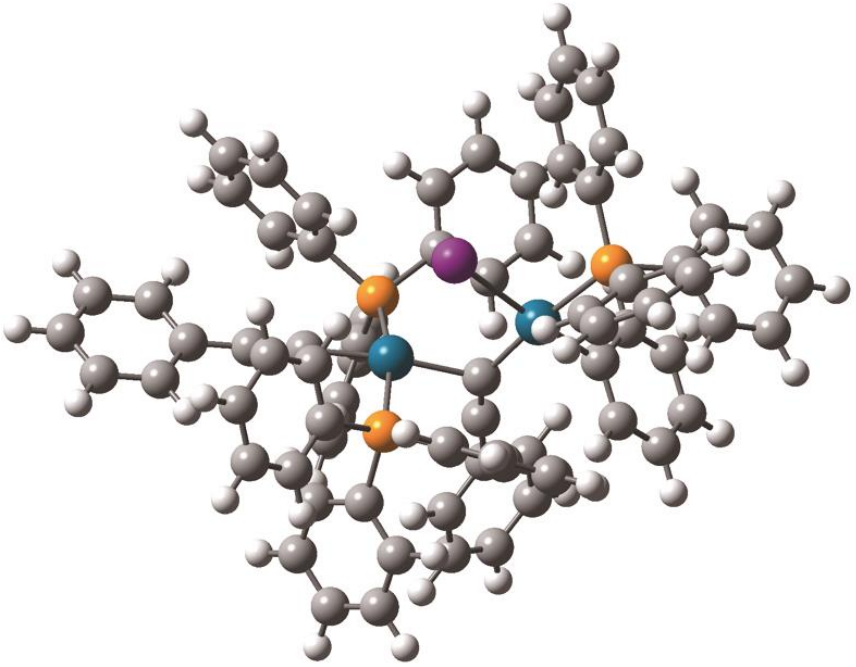

TS$^{12/13}$

Supplementary Figure 38 | Equilibrium structure of **TS$^{11/12}$** and **TS$^{12/13}$**.

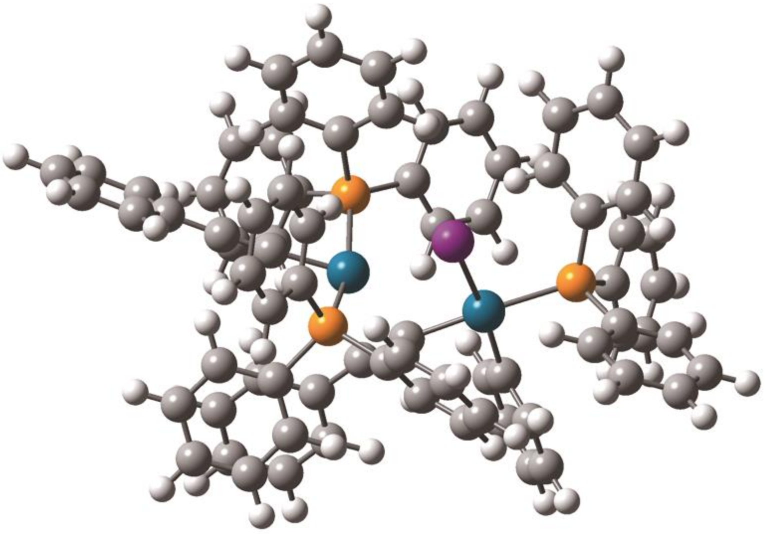

TS$^{13/14}$

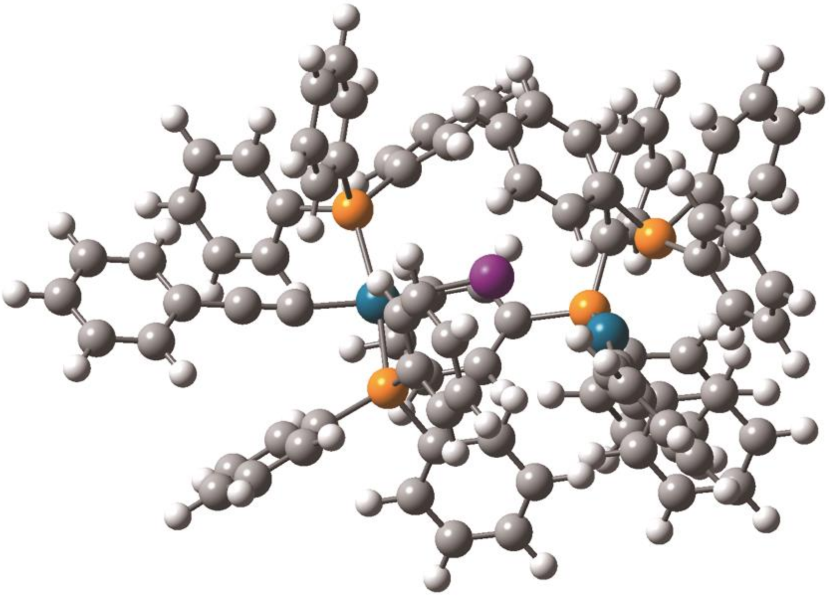

TS$^{\textit{cis}\text{-}5+8}$

Supplementary Figure 39 | Equilibrium structure of **TS**$^{13/14}$ and **TS**$^{\textit{cis}\text{-}5+8}$.

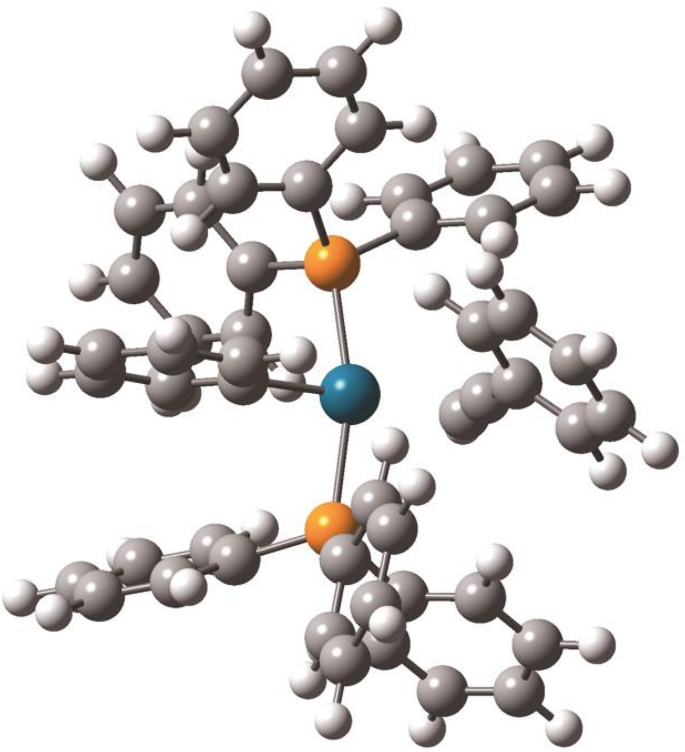

15

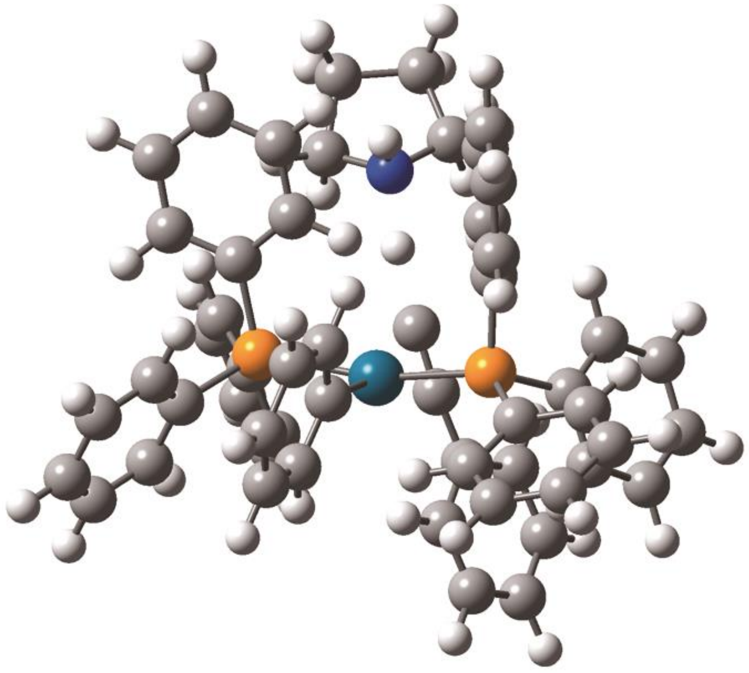

TS15

Supplementary Figure 40 | Equilibrium structure of **15** and **TS**$^{\mathbf{15}}$.

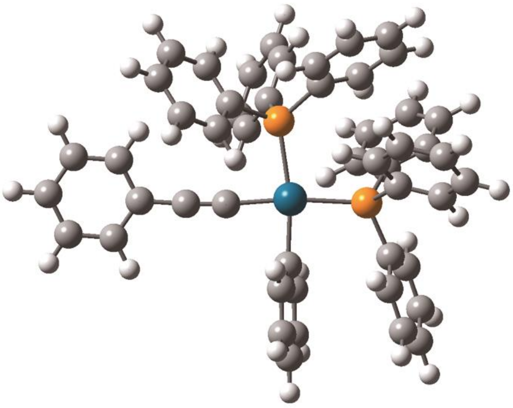

***cis*-5**

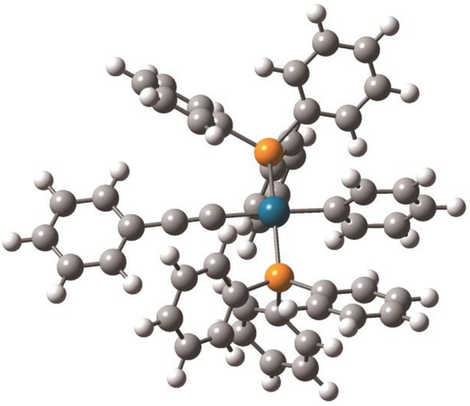

***trans*-5**

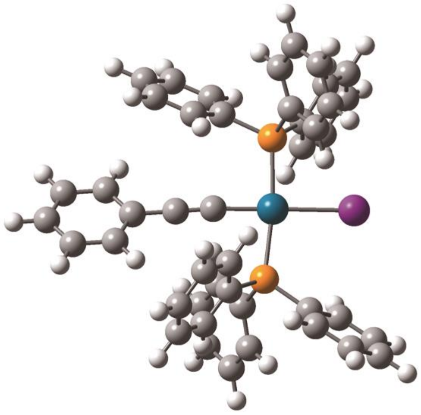

8

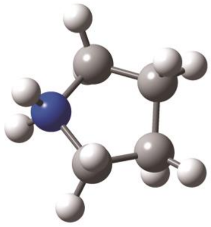

base-H+

Supplementary Figure 41 | Equilibrium structure of ***cis*-5, *trans*-5, 8** and **base-H+**.

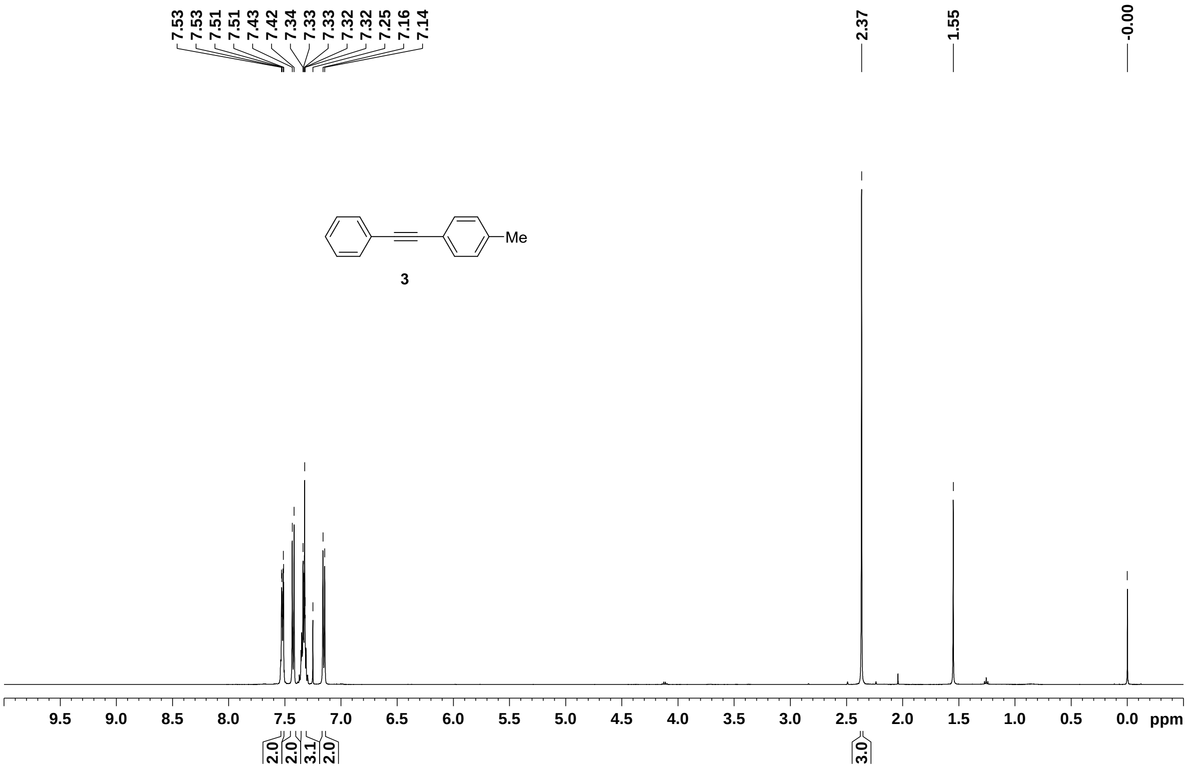

Supplementary Figure 42 | ^{1}H NMR spectrum and structure of compound **3** in $CDCl_3$, 500 MHz.

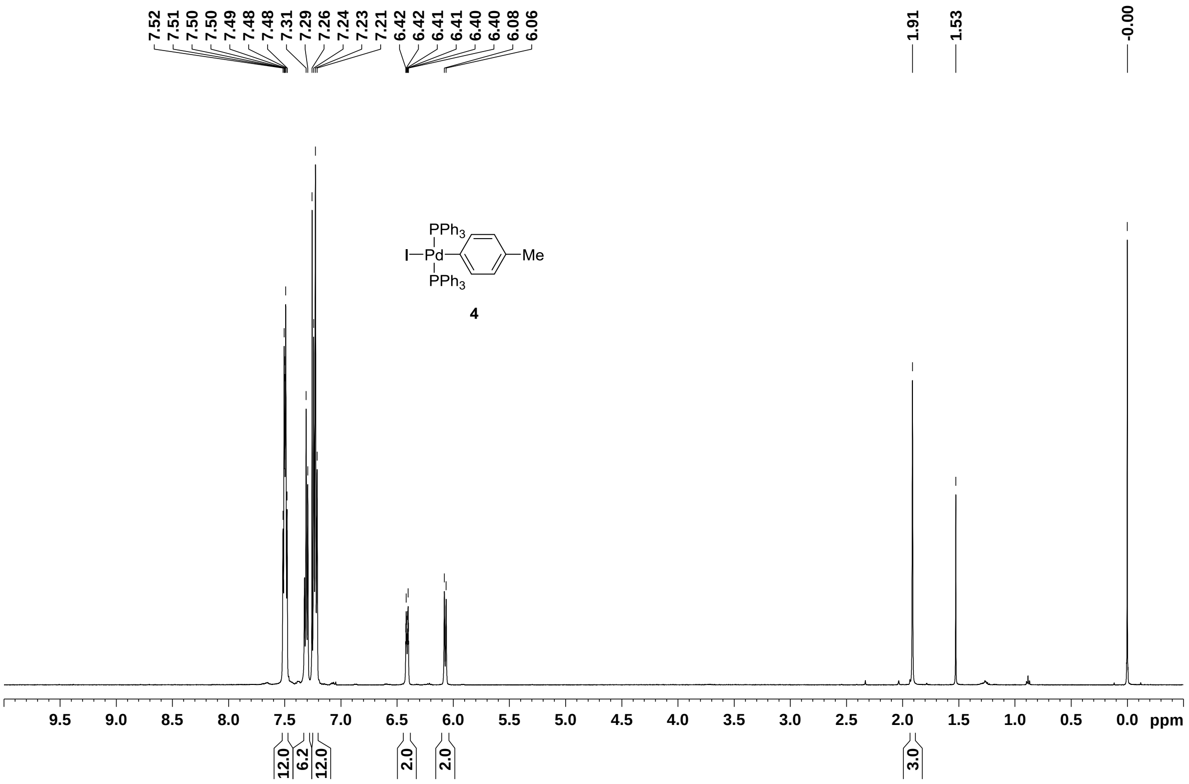

Supplementary Figure 43 | ^{1}H NMR spectrum of compound **4** in $CDCl_3$, 500 MHz.

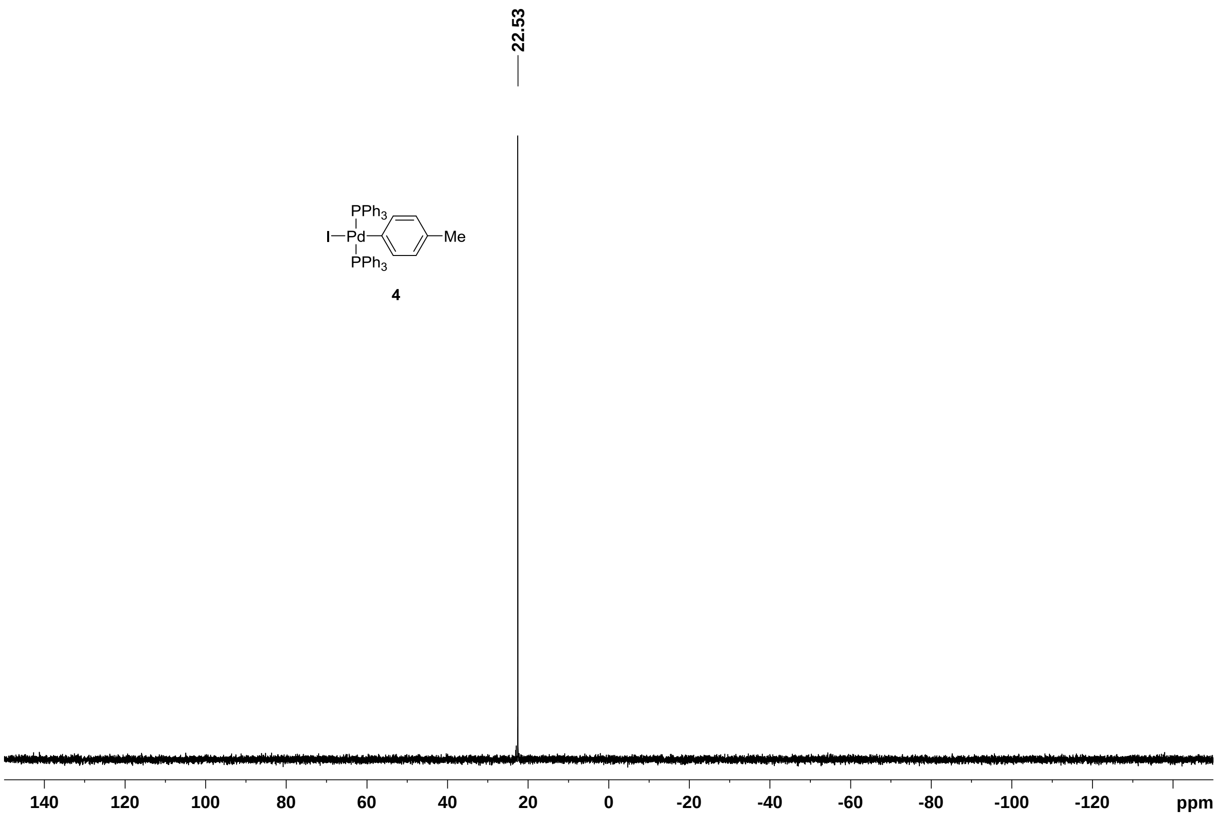

Supplementary Figure 44 | ^{31}P NMR spectrum of compound **4** in $CDCl_3$, 202 MHz.

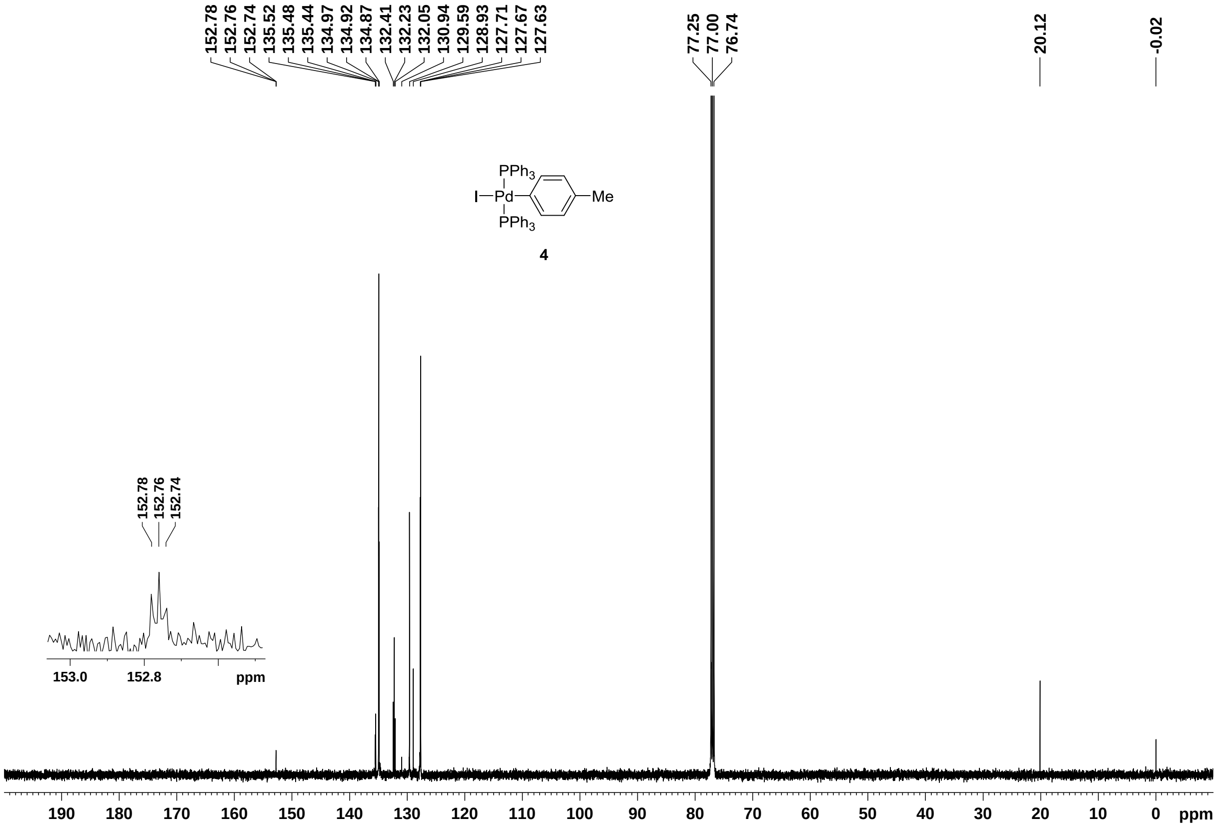

Supplementary Figure 45 | ^{13}C NMR spectrum of compound **4** in $CDCl_3$, 126 MHz.

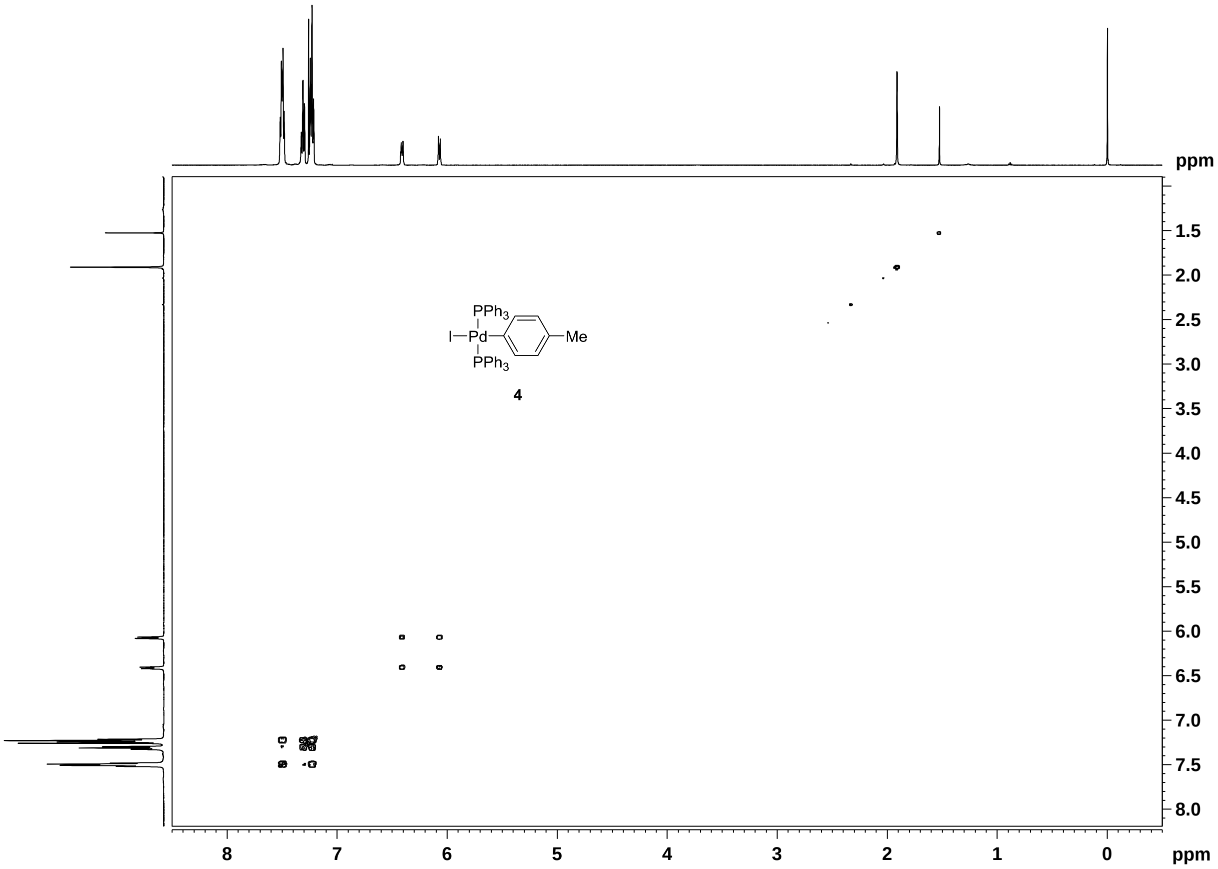

Supplementary Figure 46 | ^{1}H-^{1}H *gs*-COSY spectrum of compound **4** in $CDCl_3$.

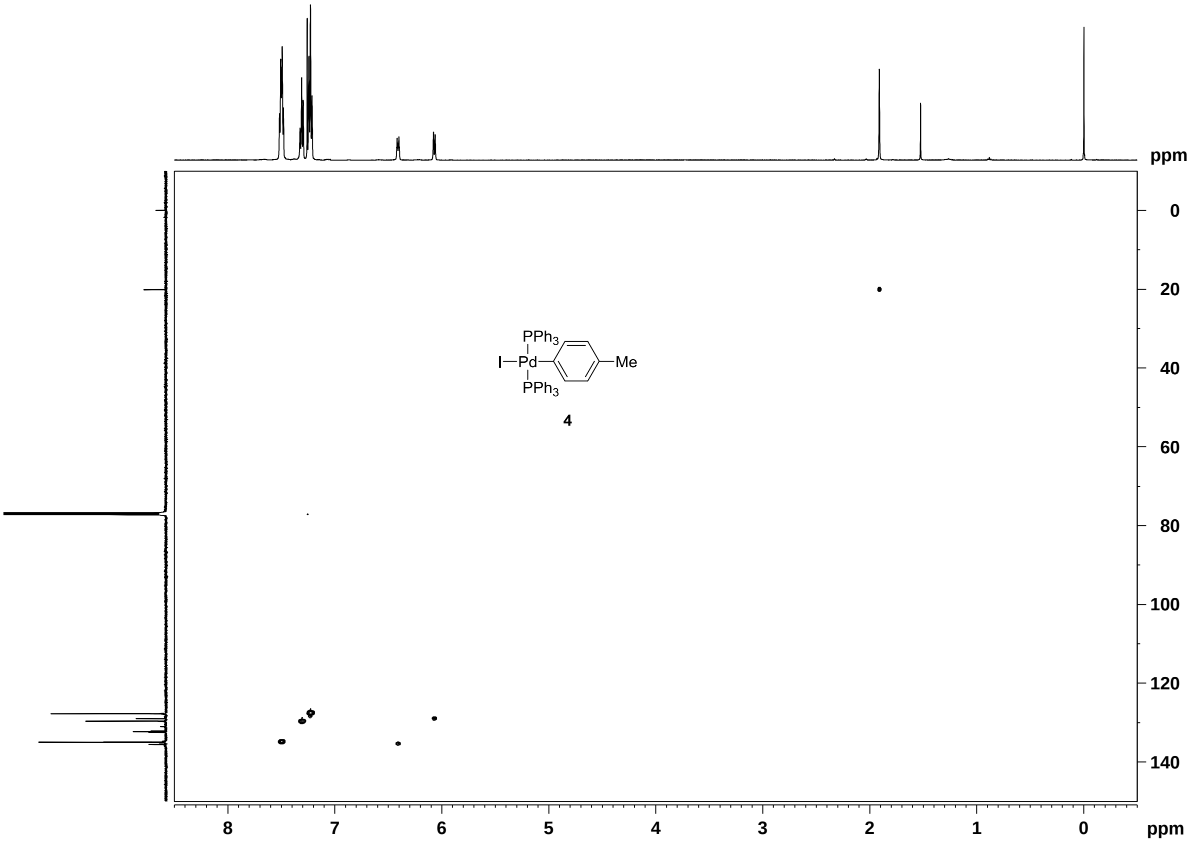

Supplementary Figure 47 | ^{1}H-^{13}C *gs*-HSQC spectrum of compound **4** in $CDCl_3$.

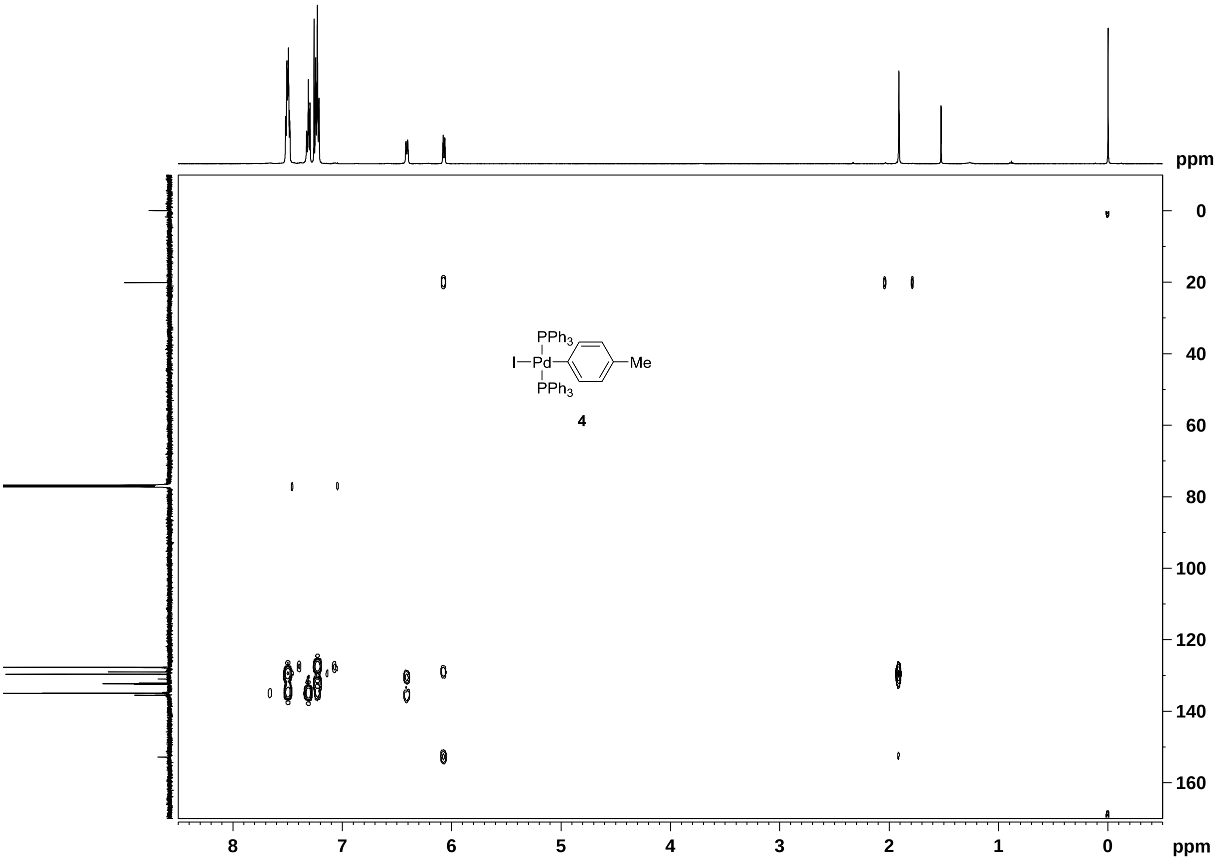

Supplementary Figure 48 | ^{1}H-^{13}C *gs*-HMBS spectrum of compound **4** in $CDCl_3$.

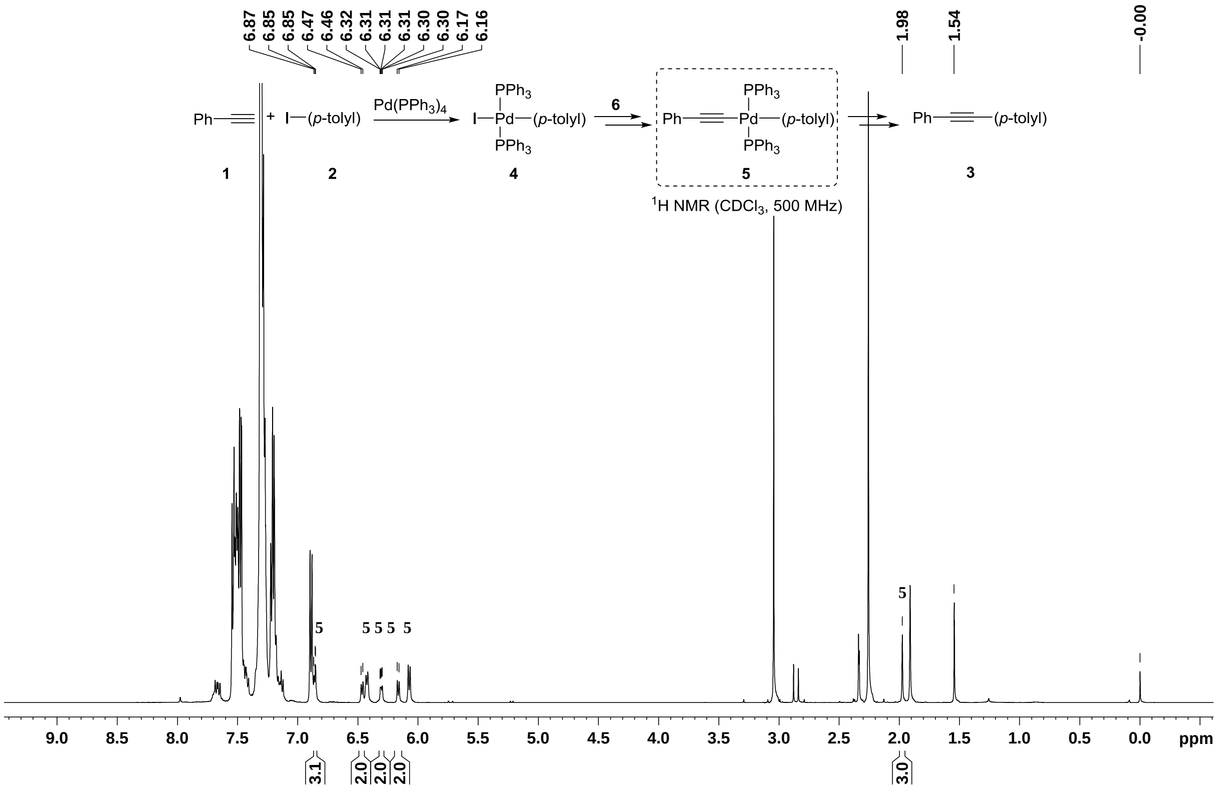

Supplementary Figure 49 | ^{1}H NMR spectrum of compound **5** prepared in a reaction mixture, recorded in $CDCl_3$, 500 MHz.

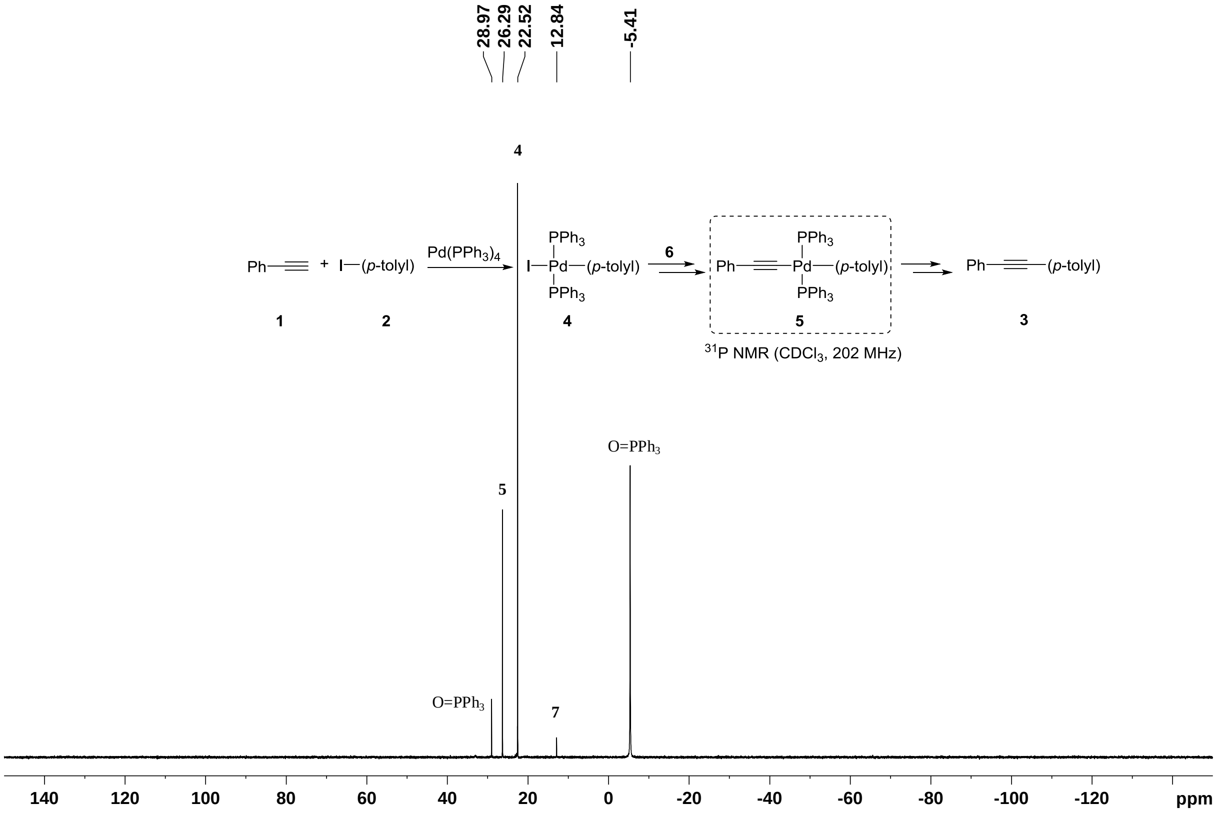

Supplementary Figure 50 | ^{31}P NMR spectrum of compound **5** prepared in a reaction mixture, recorded in $CDCl_3$, 202 MHz.

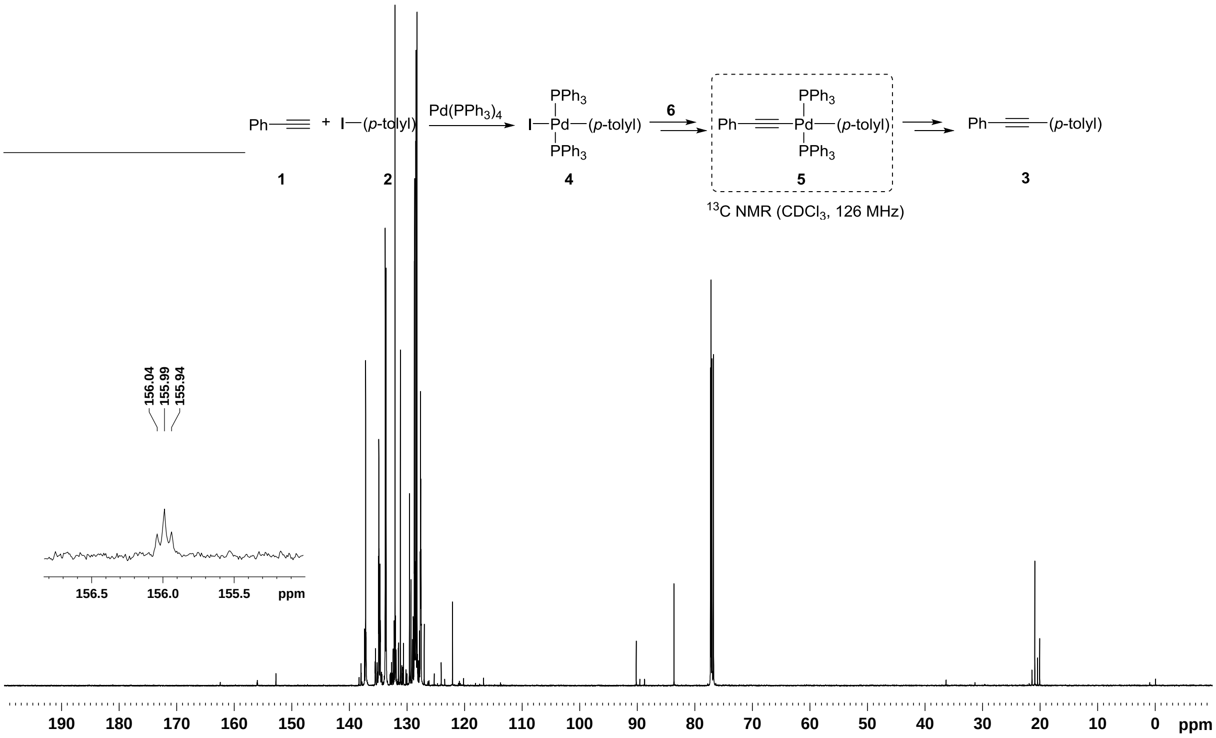

Supplementary Figure 51 | ^{13}C NMR spectrum of compound **5** prepared in a reaction mixture, recorded in $CDCl_3$, 126 MHz.

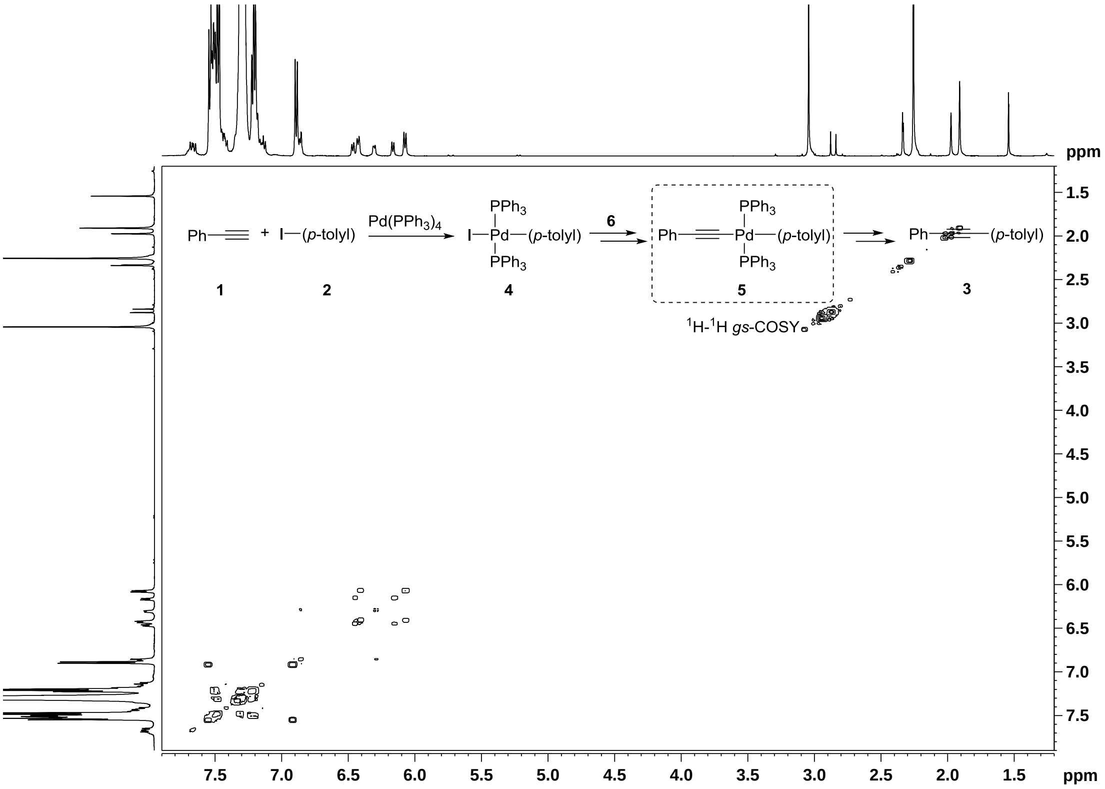

Supplementary Figure 52 | ^{1}H-^{1}H *gs*-COSY spectrum of compound **5** prepared in a reaction mixture, recorded in $CDCl_3$.

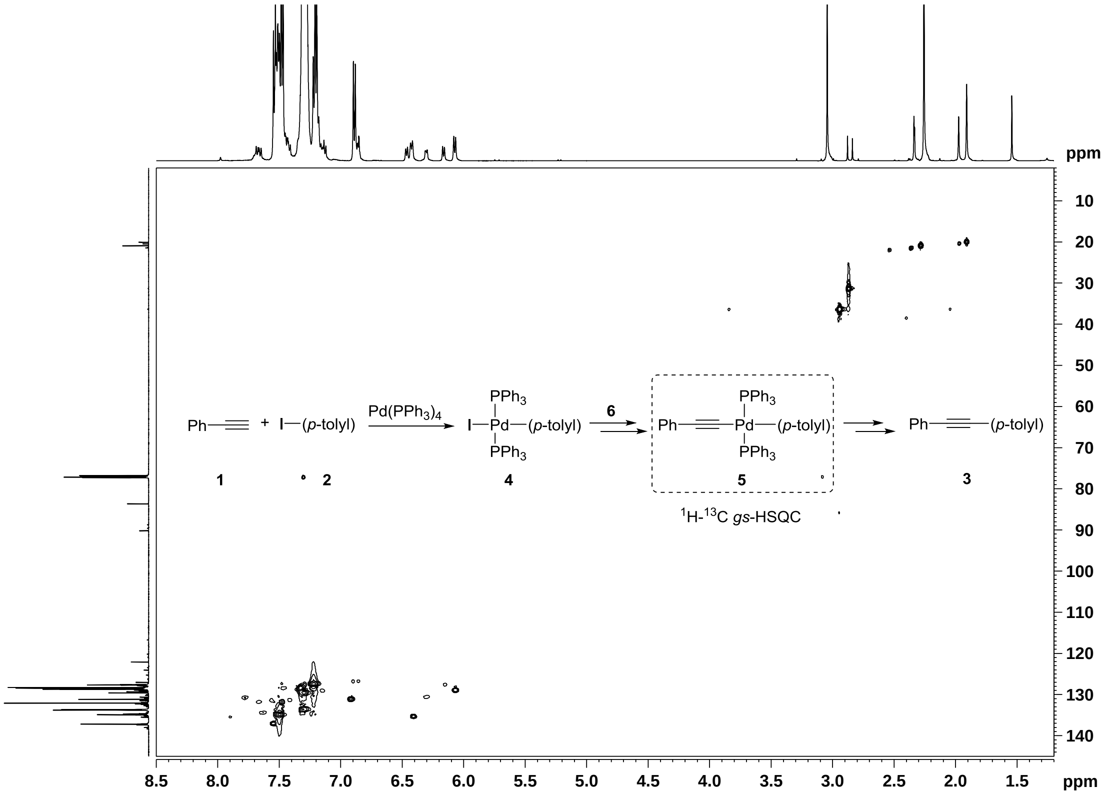

Supplementary Figure 53 | ^{1}H-^{13}C *gs*-HSQC spectrum of compound **5** prepared in a reaction mixture, recorded in $CDCl_3$.

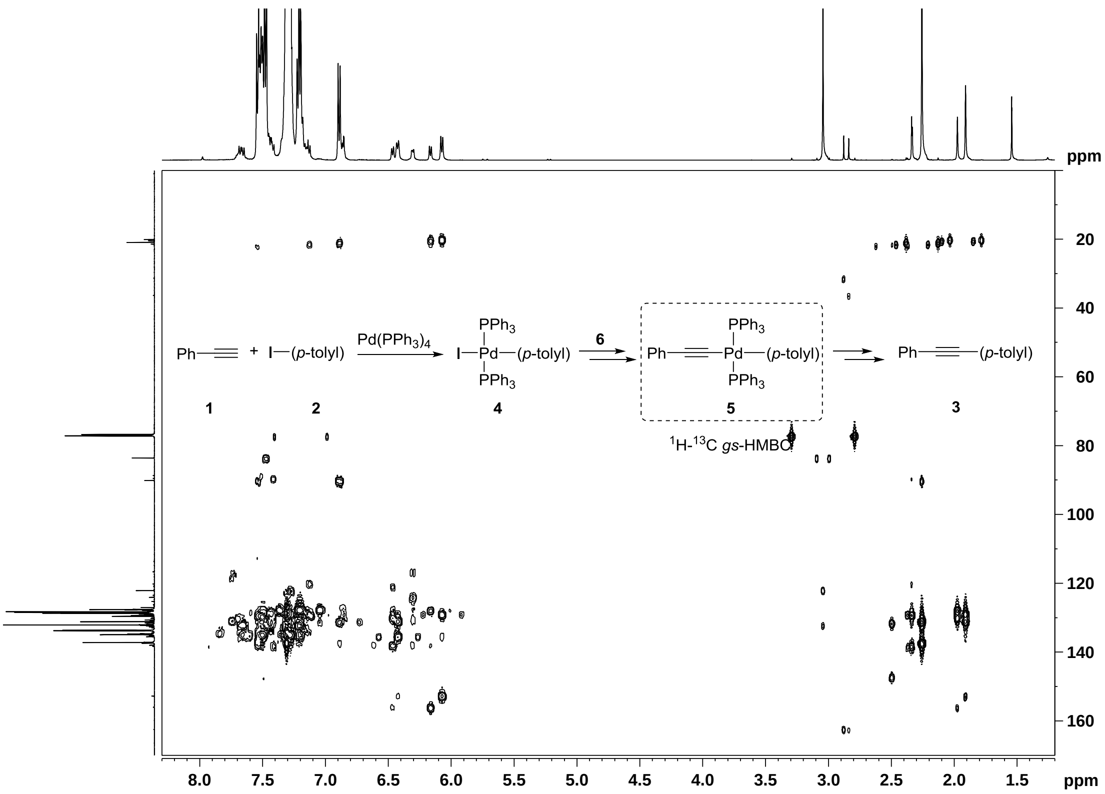

Supplementary Figure 54 | ^{1}H-^{13}C *gs*-HMBC spectrum of compound **5** prepared in a reaction mixture, recorded in $CDCl_3$.

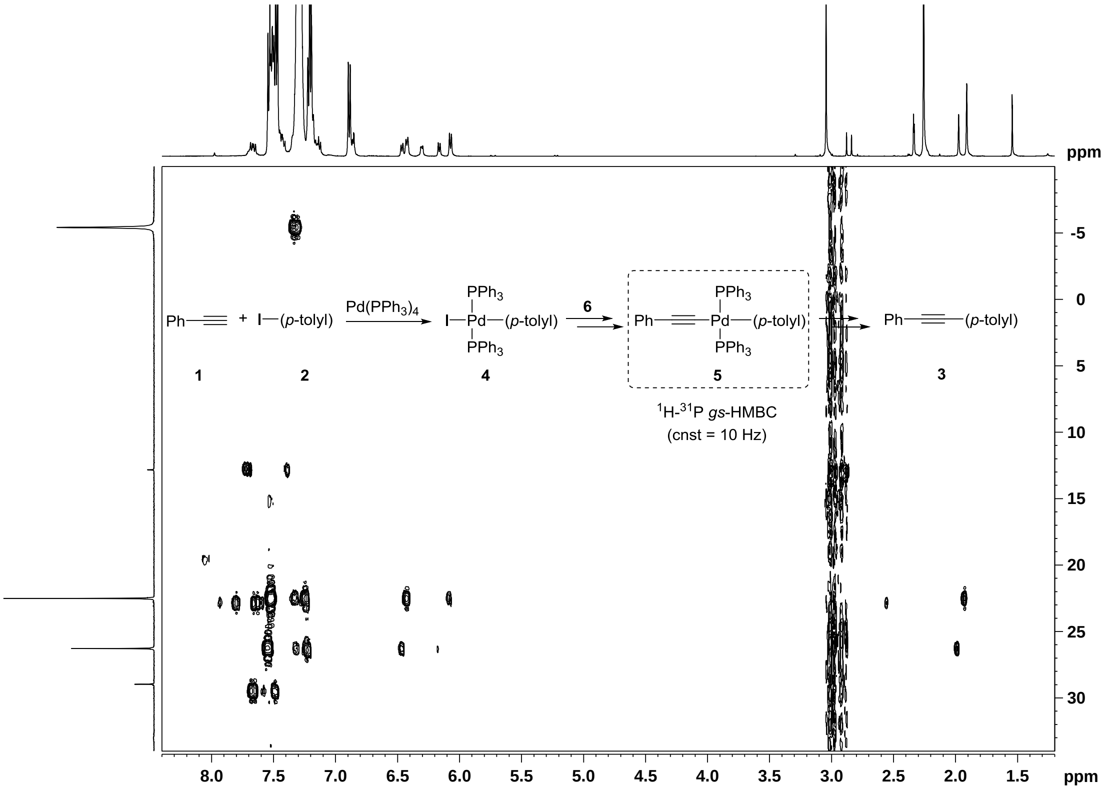

Supplementary Figure 55 | ^{1}H-^{31}P *gs*-HMBC spectrum of compound **5** prepared in a reaction mixture, recorded in $CDCl_3$.

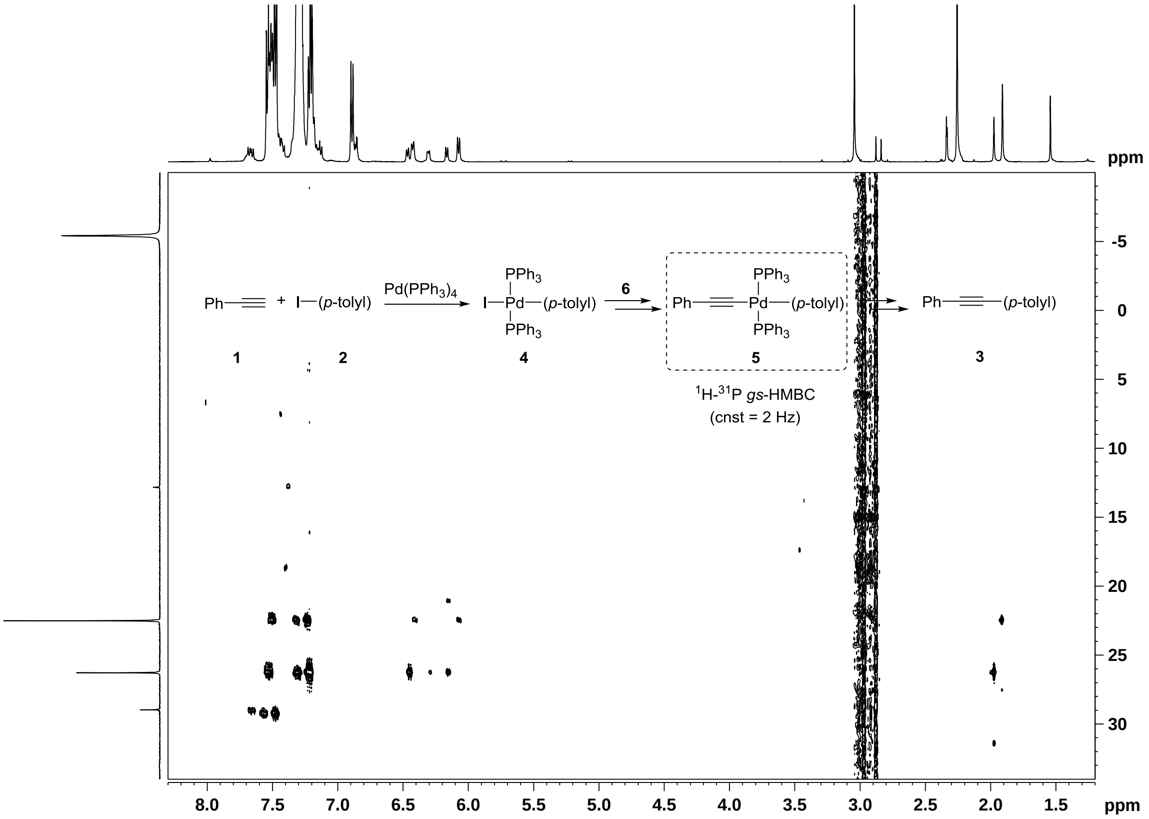

Supplementary Figure 56 | ^{1}H-^{31}P *gs*-HMBC spectrum of compound **5** prepared in a reaction mixture, recorded in $CDCl_3$.

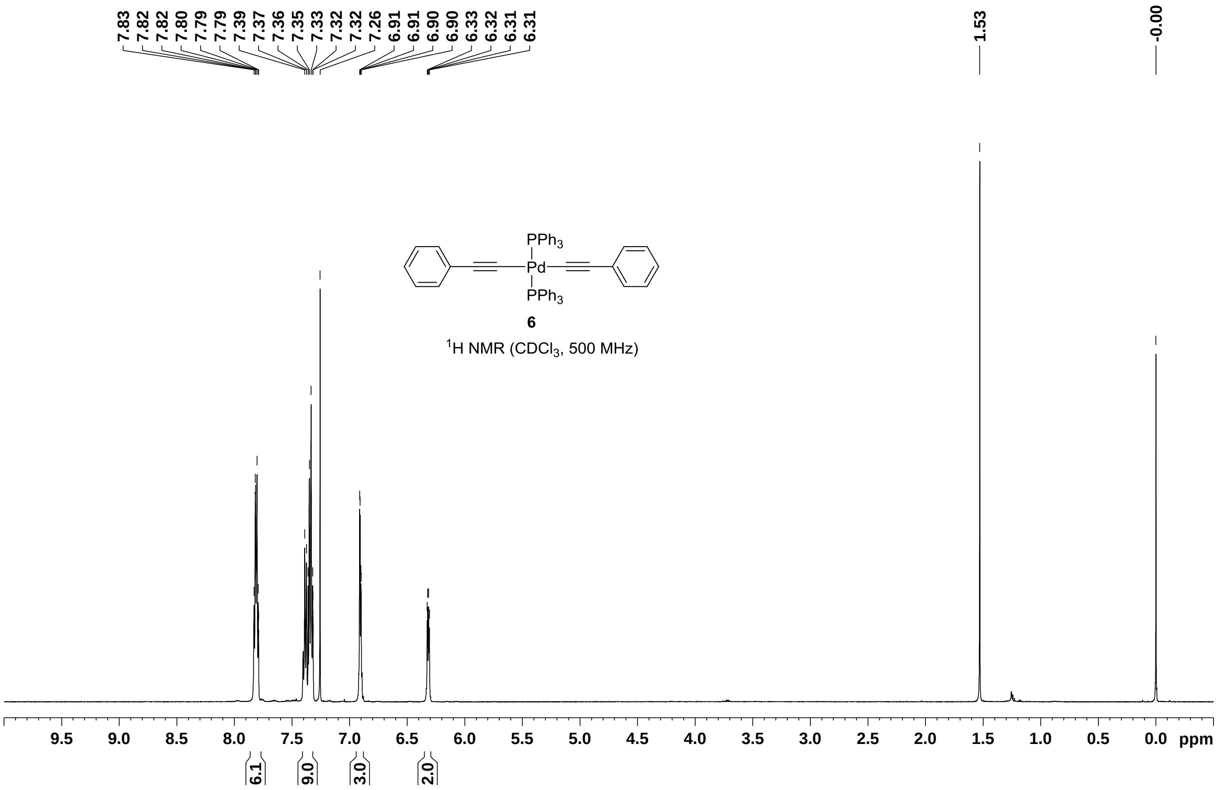

Supplementary Figure 57 | ^{1}H NMR spectrum of compound **6** in $CDCl_3$, 500 MHz.

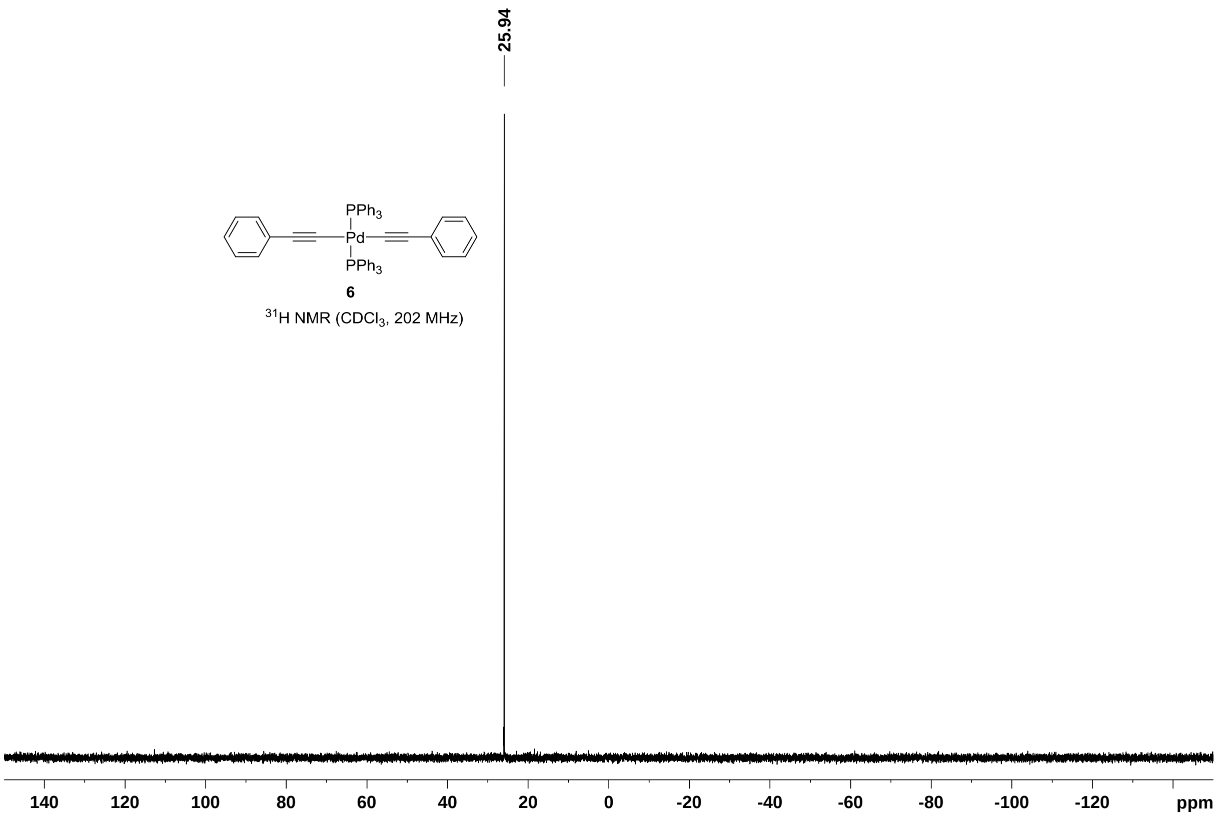

Supplementary Figure 58 | ^{31}P NMR spectrum of compound **6** in $CDCl_3$, 202 MHz.

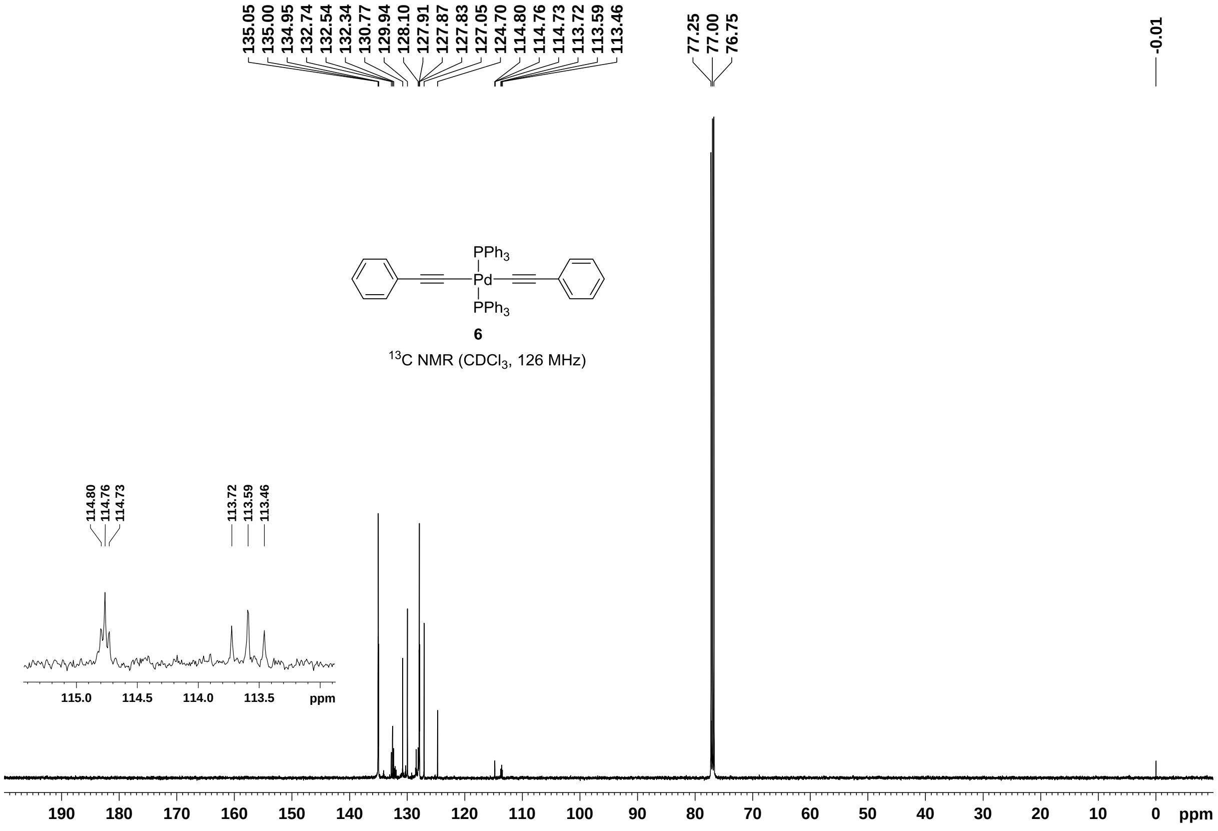

Supplementary Figure 59 | ^{13}C NMR spectrum of compound **6** in $CDCl_3$, 126 MHz.

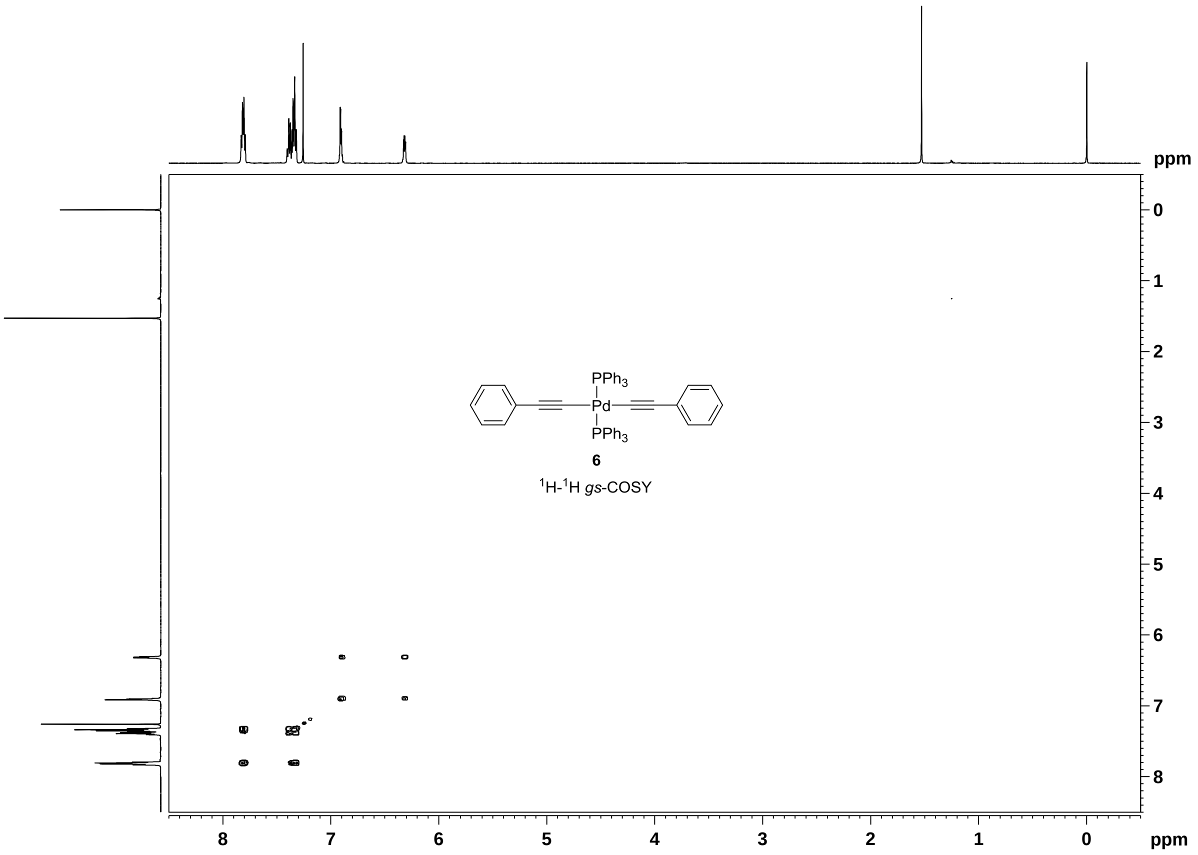

Supplementary Figure 60 | ^{1}H-^{1}H *gs*-COSY spectrum of compound **6** in $CDCl_3$.

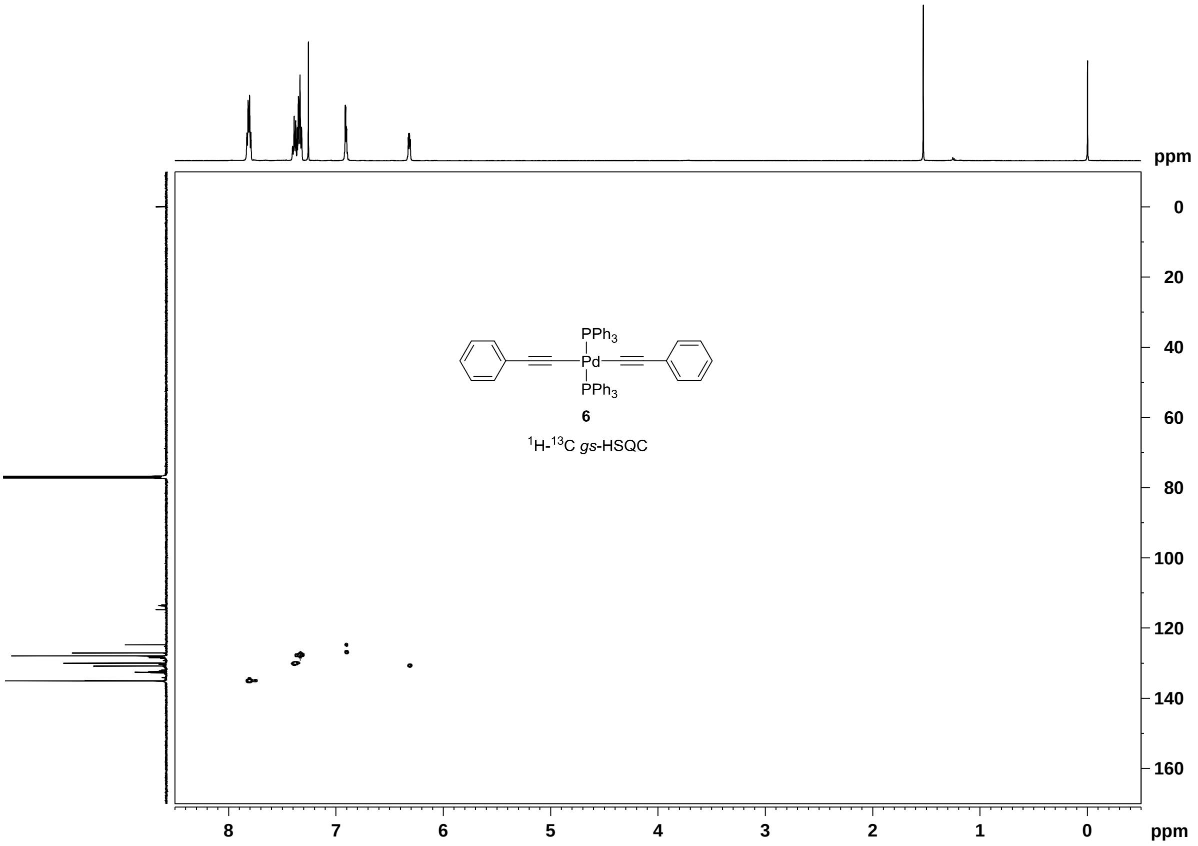

Supplementary Figure 61 | ^{1}H-^{13}C *gs*-HSQC spectrum of compound **6** in $CDCl_3$.

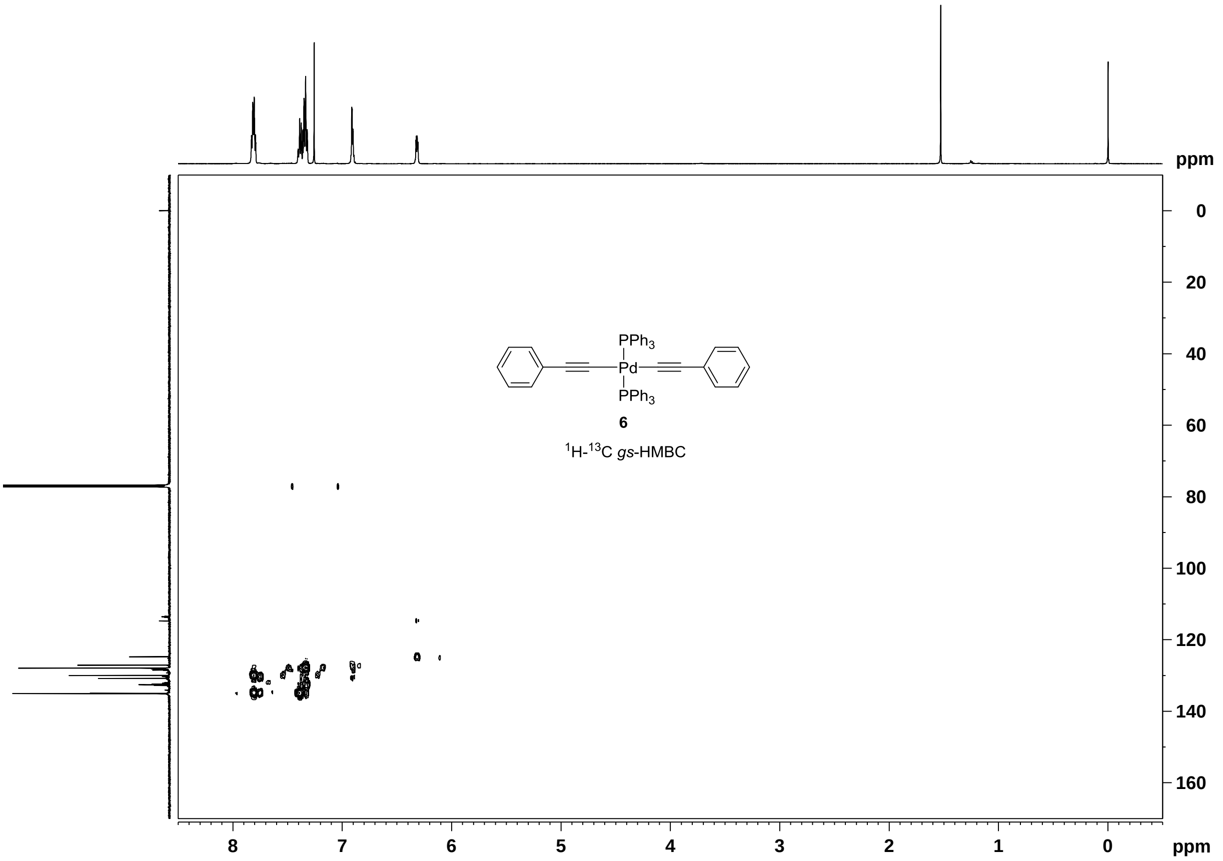

Supplementary Figure 62 | ^{1}H-^{13}C *gs*-HMBC spectrum of compound **6** in $CDCl_3$.

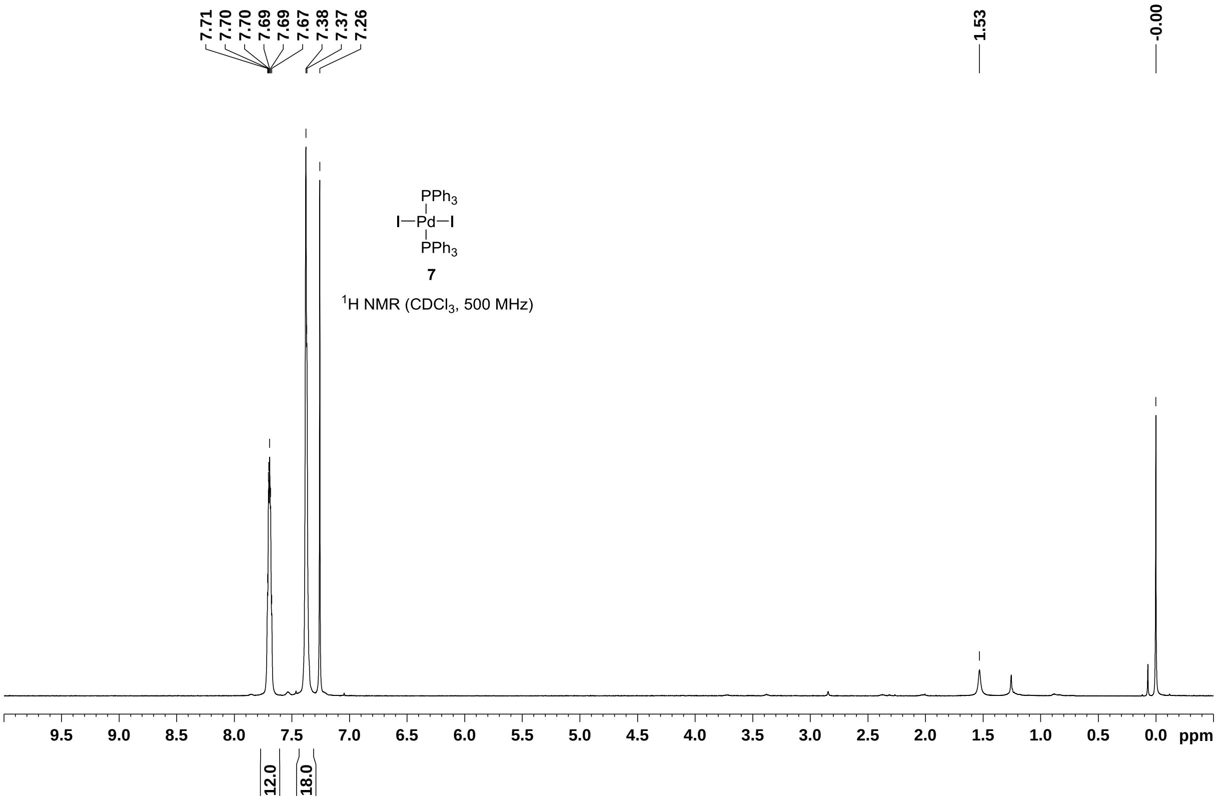

Supplementary Figure 63 | ^{1}H NMR spectrum of compound **7** in $CDCl_3$, 500 MHz.

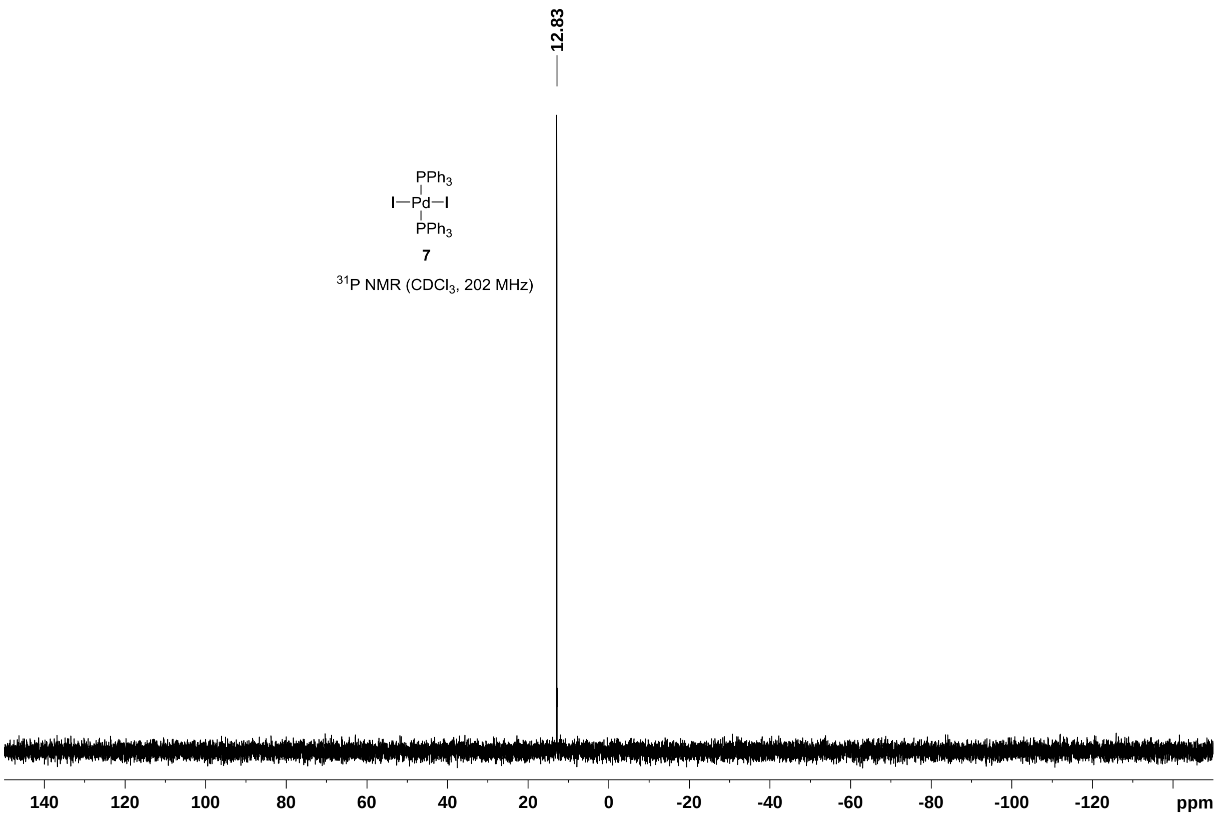

Supplementary Figure 64 | ^{31}P NMR spectrum of compound **7** in $CDCl_3$, 202 MHz.

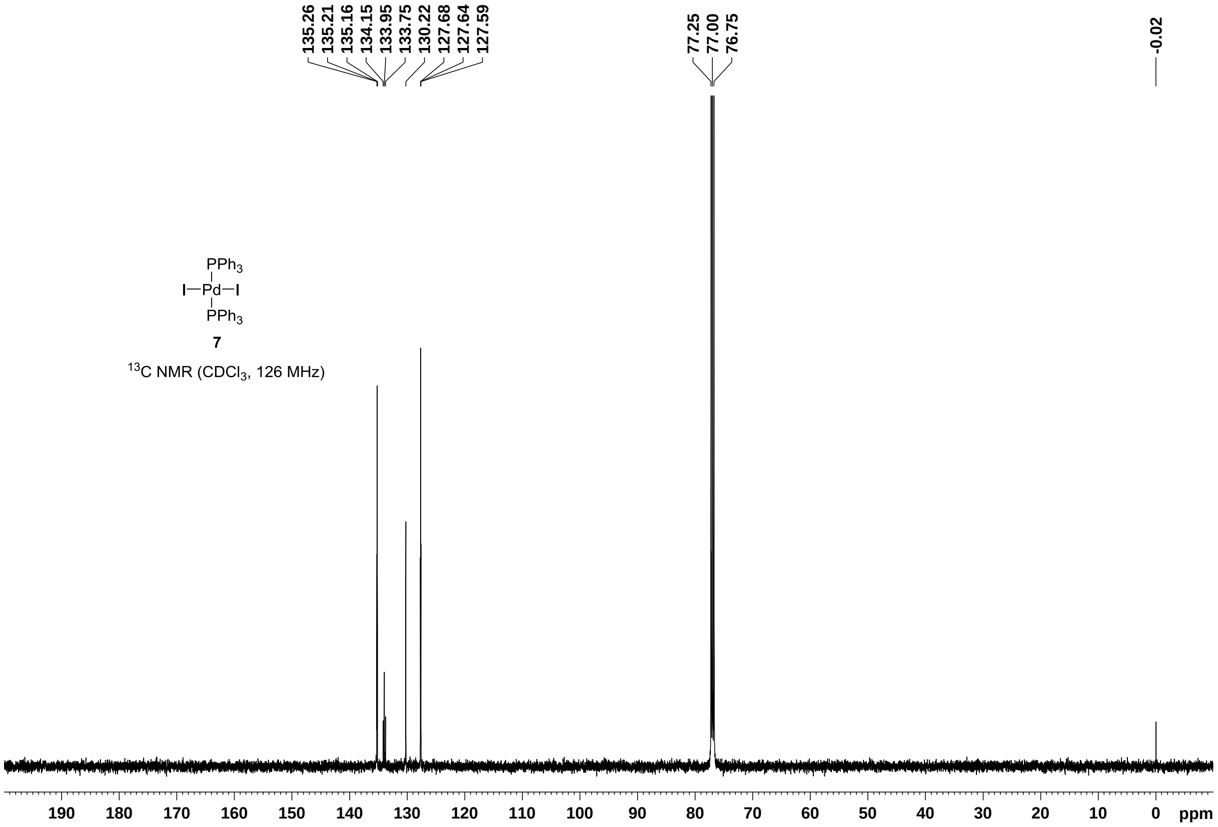

Supplementary Figure 65 | ^{13}C NMR spectrum of compound **7** in $CDCl_3$, 126 MHz.

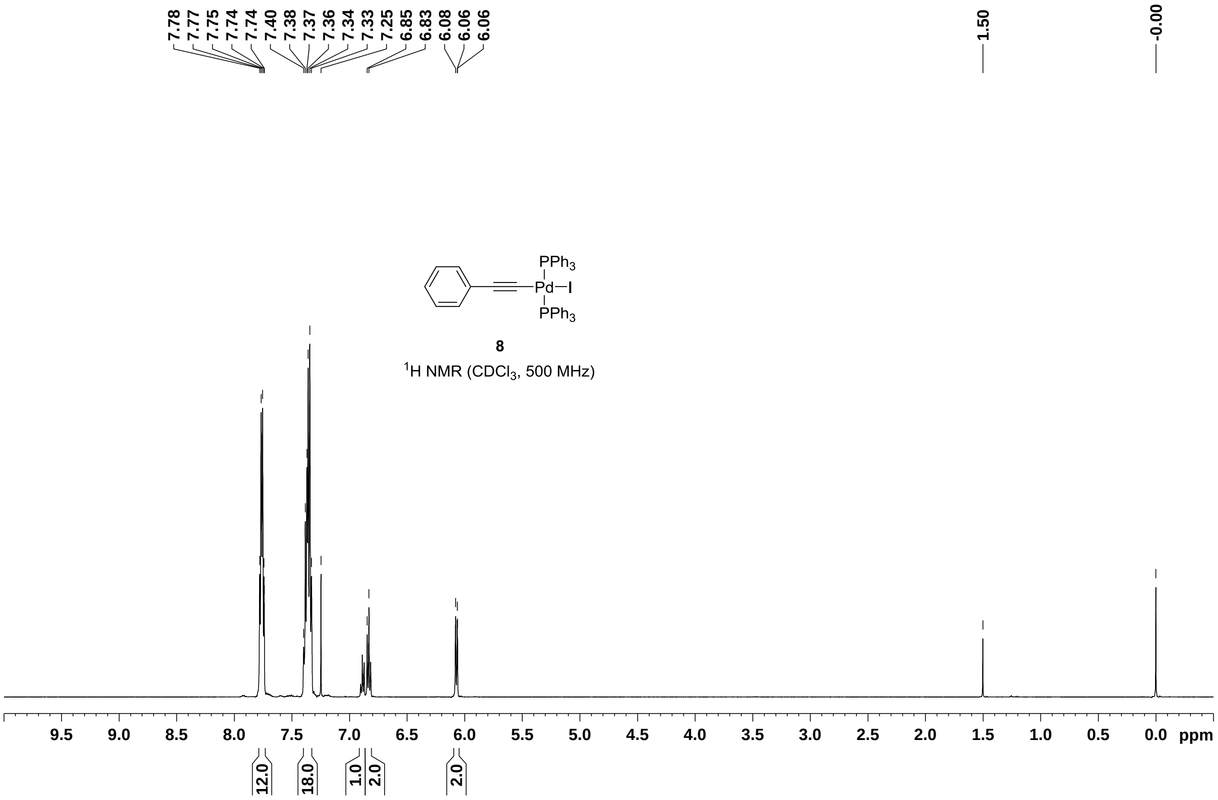

Supplementary Figure 66 | ^{1}H NMR spectrum of compound **8** in $CDCl_3$, 500 MHz.

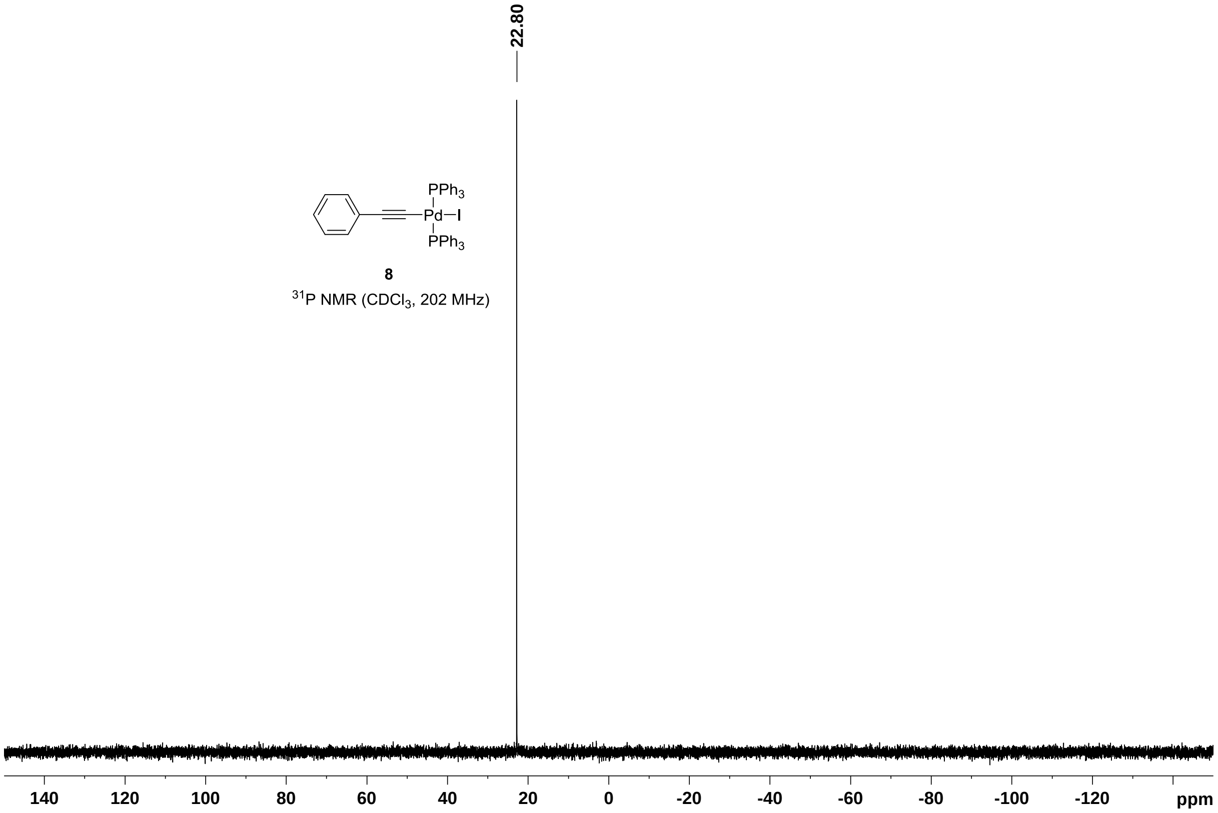

Supplementary Figure 67 | ^{31}P NMR spectrum of compound **8** in $CDCl_3$, 202 MHz.

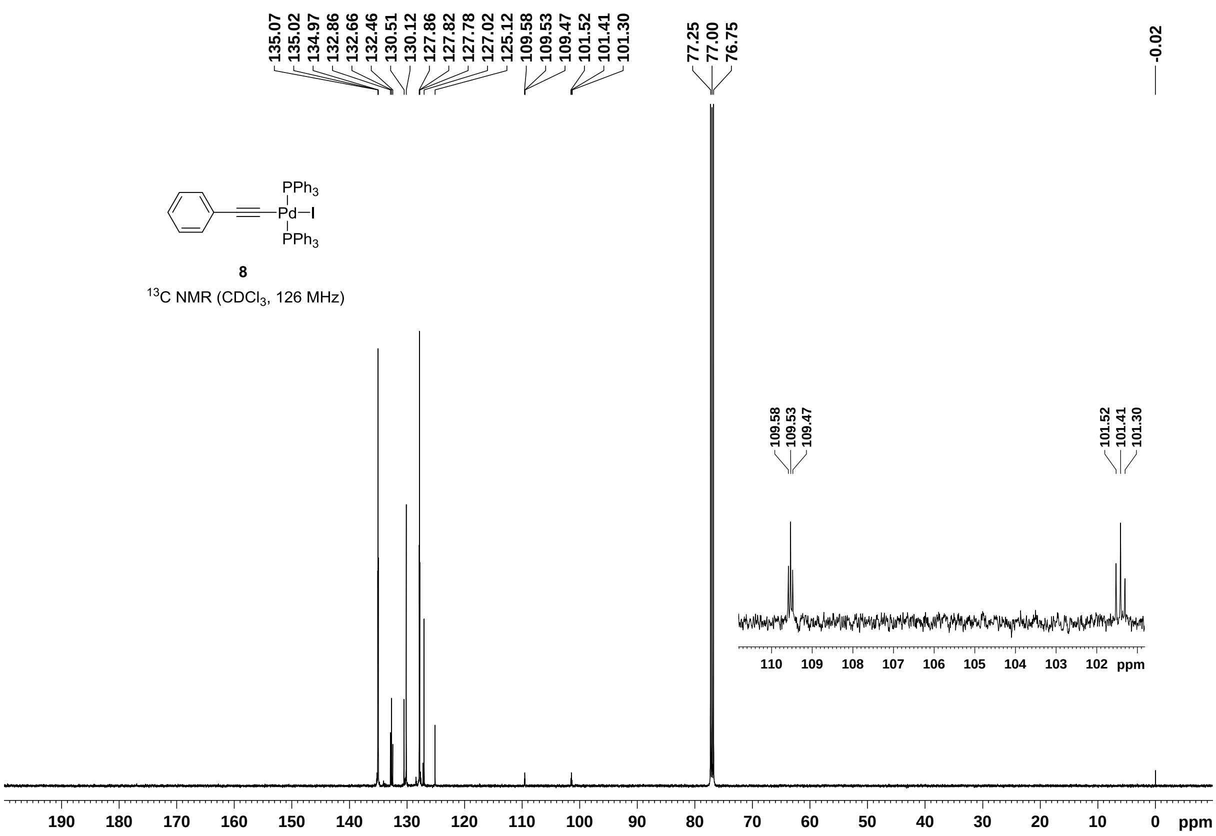

Supplementary Figure 68 | ^{13}C NMR spectrum of compound **8** in $CDCl_3$, 126 MHz.

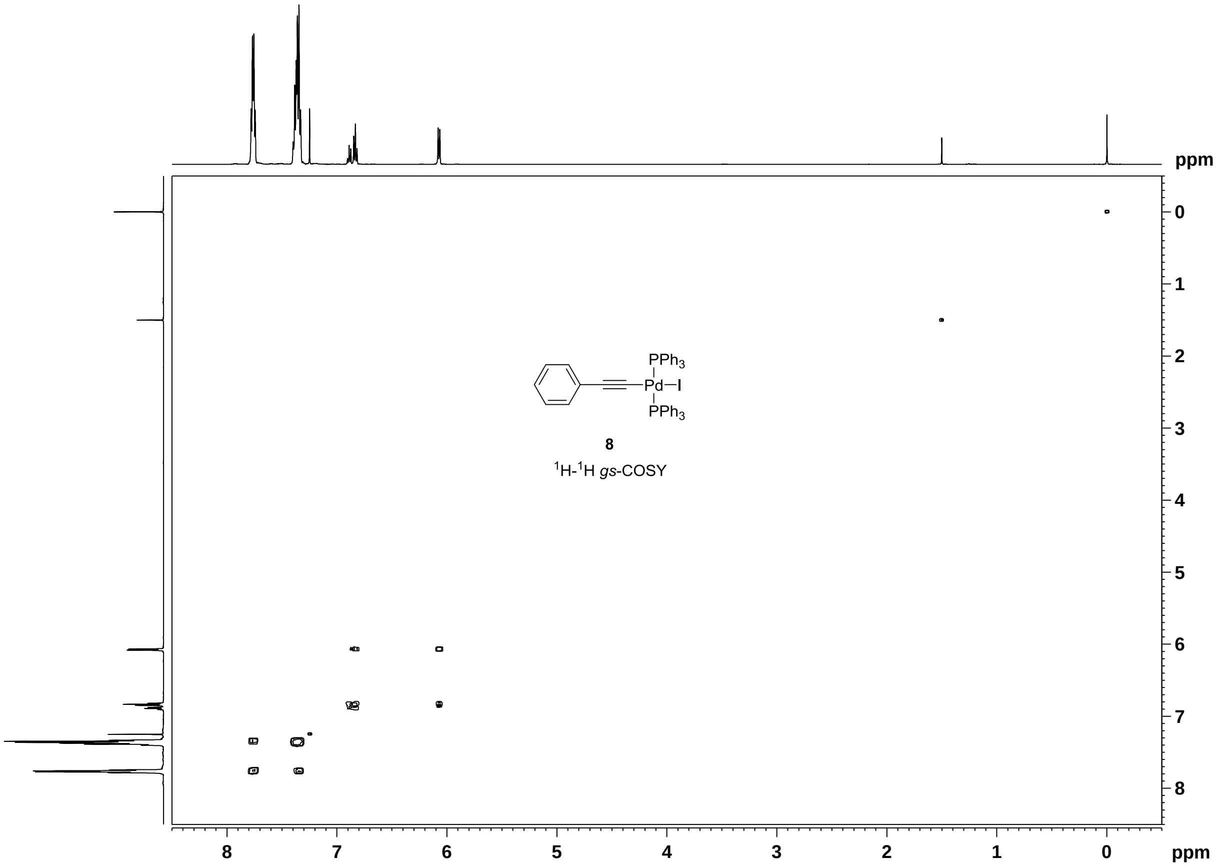

Supplementary Figure 69 | ^{1}H-^{1}H *gs*-COSY spectrum of compound **8** in $CDCl_3$.

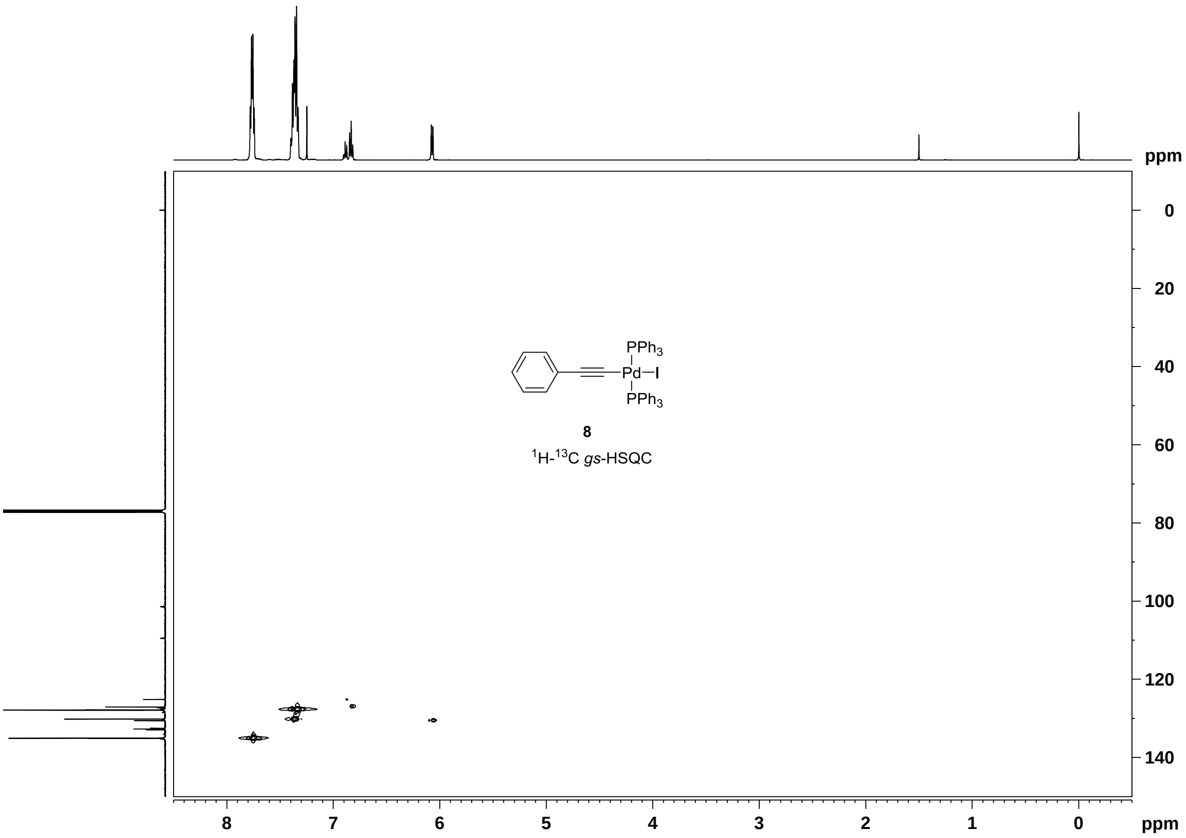

Supplementary Figure 70 | ^{1}H-^{13}C *gs*-HSQC spectrum of compound **8** in $CDCl_3$.

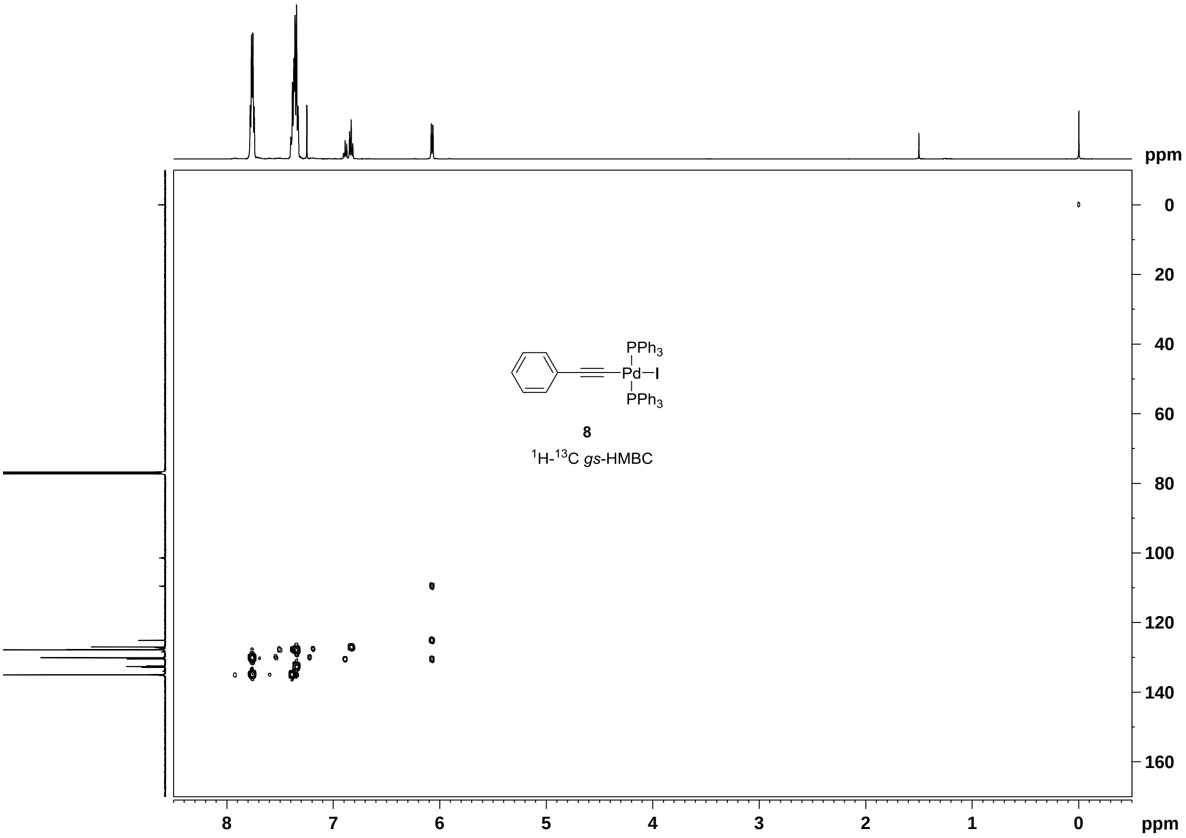

Supplementary Figure 71 | ^{1}H-^{13}C *gs*-HMBC spectrum of compound **8** in $CDCl_3$.

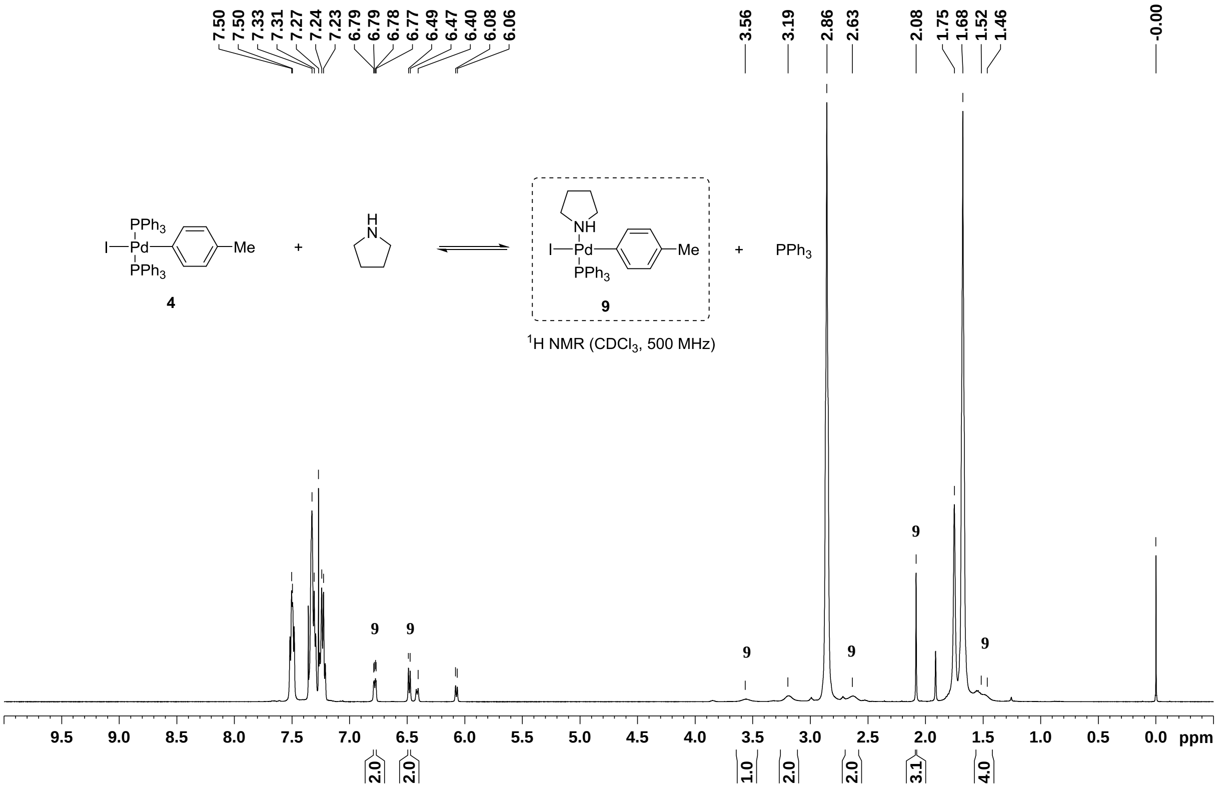

Supplementary Figure 72 | ^{1}H NMR spectrum of compound **9** prepared in a reaction mixture, recorded in $CDCl_3$, 500 MHz.

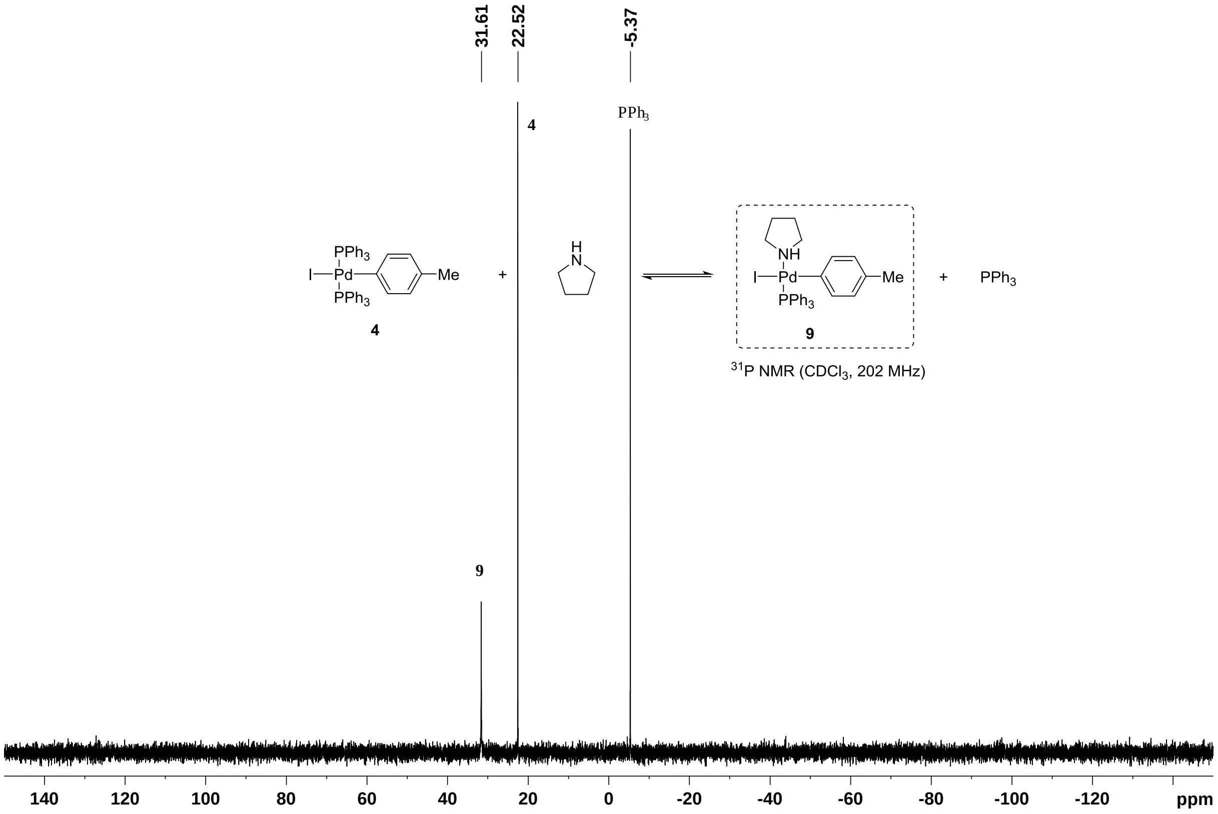

Supplementary Figure 73 | ^{31}P NMR spectrum of compound **9** prepared in a reaction mixture, recorded in $CDCl_3$, 202 MHz.

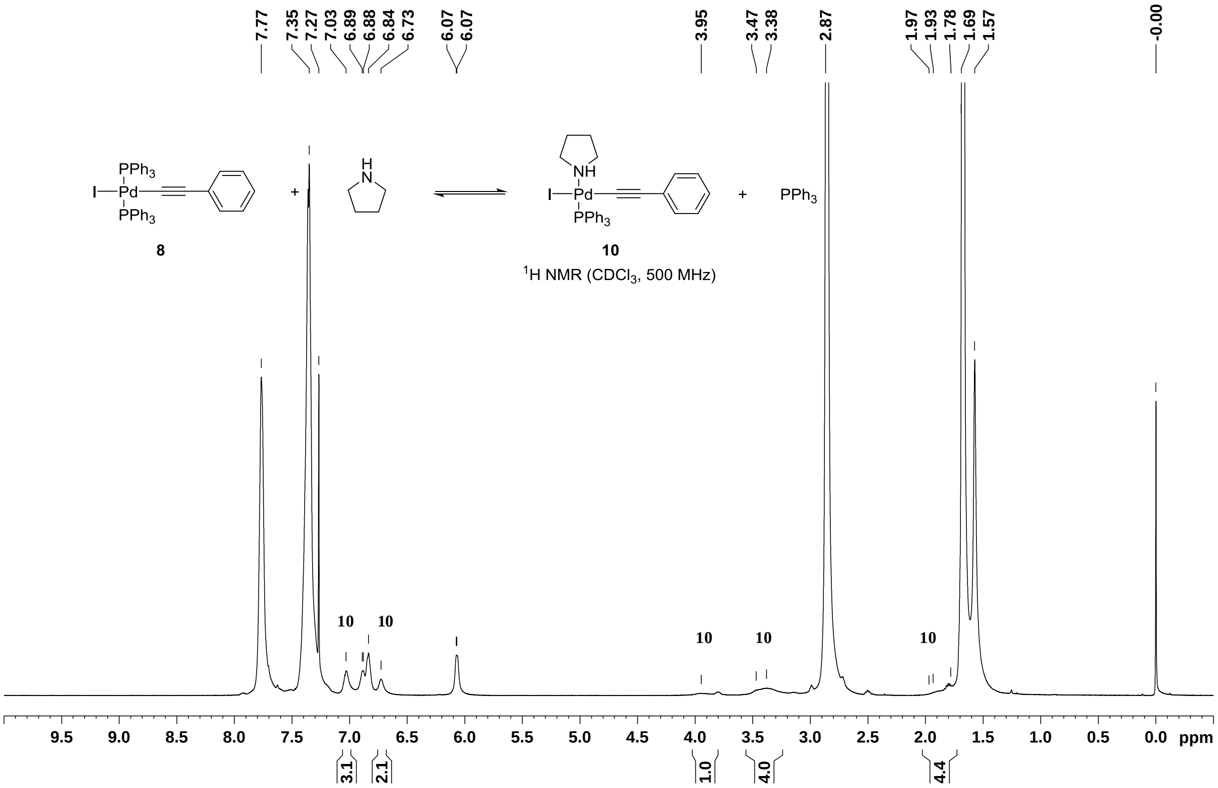

Supplementary Figure 74 | ^{1}H NMR spectrum of compound **10** prepared in a reaction mixture, recorded in $CDCl_3$, 500 MHz.

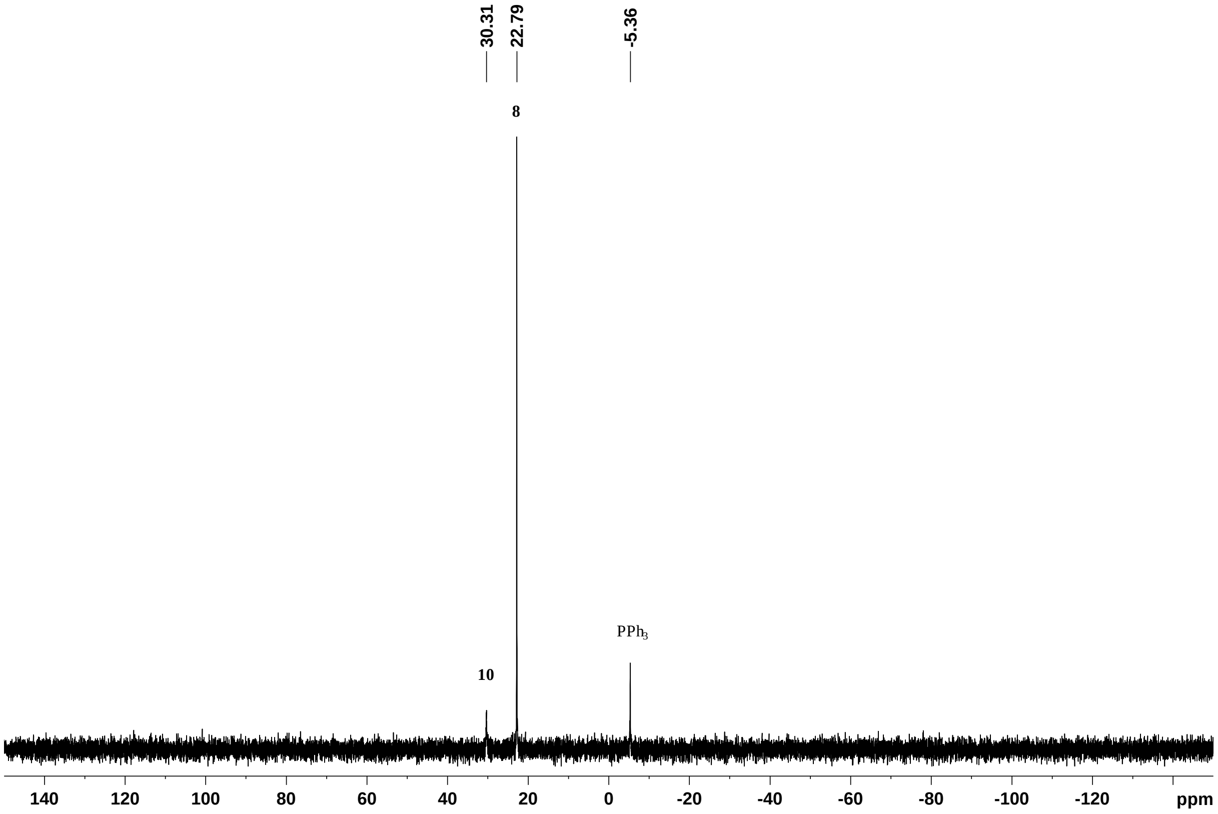

Supplementary Figure 75 | ^{31}P NMR spectrum of compound **10** prepared in a reaction mixture, recorded in $CDCl_3$, 202 MHz.

SUPPLEMENTARY REFERENCES

1 Osakada, K., Sakata, R. & Yamamoto, T. Preparation and Properties of trans-Pd(Ar)(C≡CPh)(PEt_3)$_2$. Intermolecular Alkynyl Ligand Transfer between Copper(I) and Palladium(II) Complexes Relevant to Palladium Complex Catalyzed Cross-Coupling of Terminal Alkyne with Haloarene in the Presence of CuI Cocatalyst. *Organometallics* **16**, 5354-5364 (1997).

2 Glaum, M., Kläui, W., Skelton, B. W. & White, A. H. Synthesis, X-Ray Crystal Structure and Reactivity of [(tmeda)(p-tolyl)Pd(m2-I)AgL], an Unusual Silver Iodide Complex; Reversible CO Insertion into the Pd-C Bond [Pd(PPh_3)(p-tolyl)L] (L– = [(C_5H_5Co{P(OR)$_{20}$}$_3$]–, R= Me, Pri). *Aust. J. Chem.* **50**, 1047-1052 (1997).

3 D'Amato, R. *et al.* Synthesis, characterization and optical properties of symmetrical and unsymmetrical Pt(II) and Pd(II) bis-acetylides. Crystal structure of trans-[Pt(PPh_3)$_2$(C≡C-C_6H_5)(C≡C-$C_6H_4NO_2$)]. *J. Organomet. Chem.* **627**, 13-22 (2001).

4 Vicente, J. *et al.* Syntehsis and Reactivity of Ortho-Palladated Arylureas. Synthesis and Catalytic Activity of a C,N,C Pincer Complex. Stochiometric Syntheses of Some N-Heterocycles. *Organometallics* **24**, 5044-5057 (2005).

5 Amatore, C., Bensalem, S., Ghalem, S. & Jutand, A. Mechanism of the carbopalladation of alkynes by aryl-palladium complexes. *J. Organomet. Chem.* **689**, 4642-4646 (2004).

6 Weigelt, M., Becher, D., Poetsch, E., Bruhn, C. & Steinborn, D. Zur oxidativen Addition von 1-Halogenalk-1-inen – Synthese und Struktur von Phenylalkinylpalladium-Komplexen. *Z. Anorg. Allg. Chem.* **625**, 1542-1547 (1999).

7 Amatore, C., Bensalem, S., Ghalem, S., Jutand, A. & Medjour, Y. Decelerating Effect of Alkynes in the Oxidative Addition of Phenyl Iodide to Palladium(0) Complexes in Palladium-Catalyzed Multicomponent Reactions and Sonogashira Reactions. *Eur. J. Org. Chem.*, 366-371 (2004).

8 Amatore, C., Carré, E. & Jutand, A. Evidence for the Ligation of Palladium(0) Complexes by Acetate Ions: Consequences on the Mechanism of Their Oxidative Addition with Phenyl Iodide and PhPd(OAc)(PPh_3)$_2$ as Intermediate in the Heck Reaction. *Organometallics* **14**, 5605-5614 (1995).

9 Amatore, C., Jutand, A. & M'Barki, M. A. Evidence of the Formation of Zerovalent Palladium from Pd(OAc)$_2$ and Triphenylphosphine. *Organometallics* **11**, 3009-3013 (1992).

10 Cassar, L. Synthesis of aryl- and vinyl-substituted acetylene derivatives by the use of nickel and palladium complexes. *J. Organomet. Chem.* **93**, 253-257 (1975).

11 Kakusawa, N., Yamaguchi, K. & Kurita, J. Palladium-catalyzed cross-coupling reaction of ethynylstibanes with organic halides. *J. Organomet. Chem.* **690**, 2956-2966 (2005).

12 García-Melchor, M., Pacheco, M. C., Nájera, C., Lledós, A. & Ujaque, G. Mechanistic Exploration of the Pd-Catalyzed Copper-Free Sonogashira Reaction. *ACS Catal.* **2**, 135-144 (2012).

13 Fitton, P. & Rick, E. A. The addition of aryl halides to tetrakis(triphenylphosphine)palladium(0). *J. Organomet. Chem.* **28**, 287-291 (1971).

14 Gillie, A. & Stille, J. K. Mechanisms of 1,1-Reductive Elimination from Palladium. *J. Am. Chem. Soc.* **102**, 4933-4941 (1980).

15 Zeiler, A., Ziegler, M., Rudolph, M., Rominger, F. & Hashmi, A. S. K. Scope and Limitations of the Intermolecular Furan-Yne Cyclization. *Adv. Synth. Catal.* **357**, 1507-1514 (2015).
16 He, C., Ke, J., Xu, H. & Lei, A. Synergistic Catalysis in the Sonogashira Coupling Reaction: Quantitative Kinetic Investigation of Transmetalation. *Angew. Chem. Int. Ed.* **52**, 1527-1530 (2013).
17 McLachlan, F., Mathews, C. J., Smith, P. J. & Welton, T. Palladium-Catalyzed Suzuki Cross-Coupling Reactions in Ambient Temperature Ionic Liquids: Evidence for the Importance of Palladium Imidazolylidene Complexes. *Organometallics* **22**, 5350-5357 (2003).
18 Böes, E. S., Livotto, P. R. & Stassen, H. Solvation of monovalent anions in acetonitrile and N,N-dimethylformamide: Parametrization of the IEF-PCM model. *Chem. Phys.* **331**, 142-158 (2006).
