# Supplementary material for: Mechanism of copper-free Sonogashira reaction operates through palladium-palladium transmetallation
Source: Nat Commun. 2018 Nov 16;9:4814. doi: 10.1038/s41467-018-07081-5 (PMC6240041; doi:10.1038/s41467-018-07081-5)
Supplement: Supplementary file 2 — Supplementary Data 1 [file 41467_2018_7081_MOESM2_ESM.docx]

Cartesian coordinates of the optimized structures. Values are in Å.

**8**

Pd 0.11313100 0.06892200 -0.53088800

C 2.07532000 0.39330500 -0.52559300

C 3.30713600 0.46443500 -0.45000700

C 4.73049100 0.49813100 -0.37491200

C 5.46766700 1.60368500 -0.85661800

C 5.43789700 -0.59273900 0.18191400

C 6.86132100 1.61617300 -0.77788800

H 4.92719100 2.44370800 -1.29675000

C 6.83100900 -0.57163800 0.25648300

H 4.87473000 -1.45544900 0.54273800

C 7.55047900 0.53154500 -0.22058200

H 7.41595900 2.47841500 -1.15658400

H 7.36053700 -1.42474700 0.68806800

H 8.64117700 0.54492900 -0.16110800

P -0.44845000 2.29509100 -0.16733200

P 0.48969300 -2.18206500 -0.14747000

C -1.83393800 -0.33521400 -0.44808900

C -3.03724700 -0.59123200 -0.34049300

C -4.43083600 -0.87732500 -0.26197100

C -4.89254000 -2.21155600 -0.19023100

C -5.38023500 0.17018400 -0.26297900

C -6.25941600 -2.48444600 -0.11696500

H -4.16215600 -3.02342300 -0.20335000

C -6.74457100 -0.11325300 -0.18986500

H -5.02504400 1.20035500 -0.32669900

C -7.19156500 -1.43893200 -0.11506400

H -6.60135600 -3.52103400 -0.06267700

H -7.46692300 0.70688700 -0.19174200

H -8.26060700 -1.65613900 -0.05728500

C 0.73278300 3.61793600 -0.65594600

C 2.01235100 3.62852200 -0.06942000

C 0.39939900 4.59093900 -1.61134900

C 2.93626300 4.61179500 -0.42821400

H 2.28296700 2.86372400 0.65936100

C 1.33538900 5.56588200 -1.97464000

H -0.59130200 4.59147200 -2.06973400

C 2.60198700 5.57979000 -1.38350500

H 3.92484900 4.61438700 0.03613200

H 1.06805300 6.31876000 -2.71952300

H 3.32963000 6.34406200 -1.66609500

C -2.04995700 2.82628500 -0.90562000

C -2.92186700 3.69951200 -0.23870300

C -2.39452800 2.33565600 -2.17607400

C -4.12569800 4.08115000 -0.84130000

H -2.67047600 4.07003800 0.75687500

C -3.59201500 2.72723600 -2.77827900

H -1.73219300 1.62611500 -2.67633600

C -4.46064100 3.59898000 -2.11089800

H -4.80490300 4.75380000 -0.31267200

H -3.85691100 2.33512500 -3.76234100

H -5.40391400 3.89327800 -2.57654100

C -0.65871600 2.53296700 1.64959800

C -0.40648300 3.77670700 2.25575600

C -1.10062300 1.45372900 2.43512000

C -0.60030200 3.93823900 3.63098300

H -0.05058700 4.61573900 1.65418400

C -1.29333800 1.62437400 3.80840200

H -1.29433600 0.48449300 1.97003800

C -1.04446100 2.86325300 4.40857100

H -0.39950800 4.90671000 4.09471700

H -1.63053100 0.77691000 4.40802000

H -1.19079800 2.99080700 5.48372000

C 0.23050100 -2.21693300 1.67193900

C -1.01235400 -2.57340900 2.21934200

C 1.23548400 -1.68697000 2.50282400

C -1.23315800 -2.43444300 3.59341600

H -1.81072800 -2.93589700 1.57006900

C 1.00780200 -1.55517800 3.87392100

H 2.18104100 -1.35615800 2.06608700

C -0.22388600 -1.93338100 4.42251900

H -2.20216600 -2.71294200 4.01350000

H 1.79038200 -1.14195200 4.51366100

H -0.40041600 -1.82514800 5.49520400

C 2.15116700 -2.91696700 -0.43433000

C 2.82354100 -2.58163700 -1.62106400

C 2.73271000 -3.82857800 0.46125100

C 4.06196600 -3.15817300 -1.91009200

H 2.38644600 -1.84656900 -2.29892100

C 3.97672100 -4.39902300 0.16935500

H 2.22344300 -4.08323500 1.39326700

C 4.64064600 -4.06618500 -1.01577300

H 4.58667300 -2.88028900 -2.82607800

H 4.42740400 -5.10248900 0.87315300

H 5.61598000 -4.50486100 -1.23768800

C -0.64704700 -3.44435700 -0.85347300

C -0.70274000 -4.73901100 -0.30858600

C -1.43518000 -3.12430900 -1.96869800

C -1.55581900 -5.69589200 -0.86340800

H -0.08626900 -4.99699600 0.55556300

C -2.28067500 -4.08852500 -2.52660600

H -1.40391200 -2.11316900 -2.37627100

C -2.34655500 -5.37079100 -1.97253900

H -1.60207100 -6.69750000 -0.43014600

H -2.89935900 -3.82979600 -3.38825600

H -3.01447900 -6.11963300 -2.40438500

**4**

C -0.00000600 1.95972700 0.00000900

C -0.23788100 2.68137600 1.18373600

C 0.23778500 2.68141700 -1.18371300

C -0.23532800 4.08183900 1.18497400

H -0.44352000 2.14992600 2.11714900

C 0.23509900 4.08187900 -1.18492800

H 0.44346600 2.15000000 -2.11713500

C -0.00014900 4.78743600 0.00002900

H -0.42890600 4.62179200 2.11574300

H 0.42862100 4.62186600 -2.11569000

H -0.00020900 5.88014200 0.00003600

Pd 0.00002700 -0.06504500 -0.00001800

P 2.34484100 -0.01552400 0.02910100

P -2.34480200 -0.01563500 -0.02908900

I 0.00004000 -2.80424100 -0.00009500

C 3.13720300 1.60449400 -0.36474100

C 3.69794400 1.86351600 -1.62415600

C 3.06455300 2.64094700 0.58440400

C 4.17075400 3.14455800 -1.93227600

H 3.76414300 1.06800700 -2.36851900

C 3.53600000 3.91529300 0.27213800

H 2.61924700 2.45269400 1.56253700

C 4.08658400 4.17210200 -0.98959400

H 4.60552100 3.33585300 -2.91605600

H 3.45773800 4.71440500 1.01183500

H 4.44789800 5.17312100 -1.23580300

C 3.09532300 -1.15205800 -1.21032400

C 4.30908400 -1.81860500 -0.99339900

C 2.42507300 -1.30903400 -2.43562500

C 4.84933800 -2.62916000 -1.99719100

H 4.82649800 -1.71975000 -0.03755300

C 2.97598100 -2.10603700 -3.44079700

H 1.46137200 -0.81580200 -2.58731300

C 4.18877000 -2.76940800 -3.22169400

H 5.78922300 -3.15603600 -1.81797000

H 2.44812400 -2.22412200 -4.38952400

H 4.61341200 -3.40464000 -4.00228500

C 3.09345800 -0.44978000 1.65652400

C 4.39482800 -0.02925600 1.98975100

C 2.35740800 -1.21405500 2.57669900

C 4.95207200 -0.37915000 3.22272000

H 4.96696800 0.58175700 1.28822500

C 2.92053500 -1.55963600 3.80881400

H 1.35202500 -1.55056700 2.31467500

C 4.21595400 -1.14493800 4.13400500

H 5.96291800 -0.04887100 3.47243900

H 2.34105400 -2.15686200 4.51620200

H 4.65209600 -1.41473400 5.09860200

C -3.13719000 1.60436400 0.36480100

C -3.69790000 1.86337700 1.62422900

C -3.06461800 2.64080400 -0.58436300

C -4.17074000 3.14440900 1.93235200

H -3.76403600 1.06788000 2.36861000

C -3.53609800 3.91513800 -0.27209600

H -2.61934700 2.45255400 -1.56251200

C -4.08664300 4.17194400 0.98965500

H -4.60547700 3.33570000 2.91614600

H -3.45789000 4.71424100 -1.01180800

H -4.44799100 5.17295200 1.23586200

C -3.09526200 -1.15217500 1.21033700

C -4.30912000 -1.81857500 0.99351500

C -2.42490700 -1.30925700 2.43556800

C -4.84936900 -2.62908700 1.99734300

H -4.82661900 -1.71963800 0.03772200

C -2.97580400 -2.10622000 3.44077500

H -1.46113400 -0.81613600 2.58716300

C -4.18869700 -2.76944100 3.22177800

H -5.78933400 -3.15584800 1.81820000

H -2.44787000 -2.22438900 4.38944900

H -4.61333800 -3.40463400 4.00240200

C -3.09346900 -0.44983400 -1.65651000

C -2.35745300 -1.21406000 -2.57675000

C -4.39484400 -0.02927700 -1.98967200

C -2.92062300 -1.55956700 -3.80886800

H -1.35206300 -1.55058800 -2.31477900

C -4.95213100 -0.37910100 -3.22264200

H -4.96695400 0.58171500 -1.28810300

C -4.21605000 -1.14484500 -4.13399400

H -2.34116800 -2.15675600 -4.51630900

H -5.96298300 -0.04879500 -3.47230700

H -4.65222000 -1.41458300 -5.09859400

**6**

Pd 0.11313100 0.06892200 -0.53088800

C 2.07532000 0.39330500 -0.52559300

C 3.30713600 0.46443500 -0.45000700

C 4.73049100 0.49813100 -0.37491200

C 5.46766700 1.60368500 -0.85661800

C 5.43789700 -0.59273900 0.18191400

C 6.86132100 1.61617300 -0.77788800

H 4.92719100 2.44370800 -1.29675000

C 6.83100900 -0.57163800 0.25648300

H 4.87473000 -1.45544900 0.54273800

C 7.55047900 0.53154500 -0.22058200

H 7.41595900 2.47841500 -1.15658400

H 7.36053700 -1.42474700 0.68806800

H 8.64117700 0.54492900 -0.16110800

P -0.44845000 2.29509100 -0.16733200

P 0.48969300 -2.18206500 -0.14747000

C -1.83393800 -0.33521400 -0.44808900

C -3.03724700 -0.59123200 -0.34049300

C -4.43083600 -0.87732500 -0.26197100

C -4.89254000 -2.21155600 -0.19023100

C -5.38023500 0.17018400 -0.26297900

C -6.25941600 -2.48444600 -0.11696500

H -4.16215600 -3.02342300 -0.20335000

C -6.74457100 -0.11325300 -0.18986500

H -5.02504400 1.20035500 -0.32669900

C -7.19156500 -1.43893200 -0.11506400

H -6.60135600 -3.52103400 -0.06267700

H -7.46692300 0.70688700 -0.19174200

H -8.26060700 -1.65613900 -0.05728500

C 0.73278300 3.61793600 -0.65594600

C 2.01235100 3.62852200 -0.06942000

C 0.39939900 4.59093900 -1.61134900

C 2.93626300 4.61179500 -0.42821400

H 2.28296700 2.86372400 0.65936100

C 1.33538900 5.56588200 -1.97464000

H -0.59130200 4.59147200 -2.06973400

C 2.60198700 5.57979000 -1.38350500

H 3.92484900 4.61438700 0.03613200

H 1.06805300 6.31876000 -2.71952300

H 3.32963000 6.34406200 -1.66609500

C -2.04995700 2.82628500 -0.90562000

C -2.92186700 3.69951200 -0.23870300

C -2.39452800 2.33565600 -2.17607400

C -4.12569800 4.08115000 -0.84130000

H -2.67047600 4.07003800 0.75687500

C -3.59201500 2.72723600 -2.77827900

H -1.73219300 1.62611500 -2.67633600

C -4.46064100 3.59898000 -2.11089800

H -4.80490300 4.75380000 -0.31267200

H -3.85691100 2.33512500 -3.76234100

H -5.40391400 3.89327800 -2.57654100

C -0.65871600 2.53296700 1.64959800

C -0.40648300 3.77670700 2.25575600

C -1.10062300 1.45372900 2.43512000

C -0.60030200 3.93823900 3.63098300

H -0.05058700 4.61573900 1.65418400

C -1.29333800 1.62437400 3.80840200

H -1.29433600 0.48449300 1.97003800

C -1.04446100 2.86325300 4.40857100

H -0.39950800 4.90671000 4.09471700

H -1.63053100 0.77691000 4.40802000

H -1.19079800 2.99080700 5.48372000

C 0.23050100 -2.21693300 1.67193900

C -1.01235400 -2.57340900 2.21934200

C 1.23548400 -1.68697000 2.50282400

C -1.23315800 -2.43444300 3.59341600

H -1.81072800 -2.93589700 1.57006900

C 1.00780200 -1.55517800 3.87392100

H 2.18104100 -1.35615800 2.06608700

C -0.22388600 -1.93338100 4.42251900

H -2.20216600 -2.71294200 4.01350000

H 1.79038200 -1.14195200 4.51366100

H -0.40041600 -1.82514800 5.49520400

C 2.15116700 -2.91696700 -0.43433000

C 2.82354100 -2.58163700 -1.62106400

C 2.73271000 -3.82857800 0.46125100

C 4.06196600 -3.15817300 -1.91009200

H 2.38644600 -1.84656900 -2.29892100

C 3.97672100 -4.39902300 0.16935500

H 2.22344300 -4.08323500 1.39326700

C 4.64064600 -4.06618500 -1.01577300

H 4.58667300 -2.88028900 -2.82607800

H 4.42740400 -5.10248900 0.87315300

H 5.61598000 -4.50486100 -1.23768800

C -0.64704700 -3.44435700 -0.85347300

C -0.70274000 -4.73901100 -0.30858600

C -1.43518000 -3.12430900 -1.96869800

C -1.55581900 -5.69589200 -0.86340800

H -0.08626900 -4.99699600 0.55556300

C -2.28067500 -4.08852500 -2.52660600

H -1.40391200 -2.11316900 -2.37627100

C -2.34655500 -5.37079100 -1.97253900

H -1.60207100 -6.69750000 -0.43014600

H -2.89935900 -3.82979600 -3.38825600

H -3.01447900 -6.11963300 -2.40438500

***cis*-5**

C -1.94059300 1.46753900 -0.13134200

C -3.13452000 1.77667800 -0.06764800

C -4.53414400 2.05026000 -0.02486200

C -5.02527100 3.36546500 0.13405000

C -5.46500500 0.99268900 -0.14752400

C -6.39913600 3.61037500 0.16908800

H -4.31102800 4.18551400 0.22727300

C -6.83645700 1.24665800 -0.11309300

H -5.09101000 -0.02429700 -0.27457100

C -7.31148000 2.55479500 0.04587700

H -6.76220800 4.63375100 0.29265500

H -7.54101700 0.41681100 -0.21158200

H -8.38593900 2.75093500 0.07311500

P 2.23369300 0.35349400 -0.01805800

C 0.37203100 2.81325800 -0.28378100

C 0.36849300 3.47359400 -1.52435000

C 0.72336600 3.54767200 0.86185100

C 0.74723300 4.81972100 -1.62194400

H 0.06817500 2.93660500 -2.42784700

C 1.09720100 4.89315600 0.76626600

H 0.72657400 3.06414700 1.84239300

C 1.11807400 5.53347900 -0.47775100

H 0.74529300 5.31264400 -2.59858500

H 1.38146600 5.44065200 1.66907900

H 1.41539400 6.58241700 -0.55333700

C 3.34738700 1.78737900 0.29280900

C 3.57056400 2.69748700 -0.75598700

C 3.88028800 2.05054500 1.56284000

C 4.31048900 3.85758000 -0.53082500

H 3.14282700 2.50855600 -1.74202600

C 4.62619400 3.21432300 1.78188000

H 3.70831200 1.35263700 2.38449600

C 4.83788800 4.12011300 0.73953300

H 4.45977000 4.56794000 -1.34607300

H 5.03715800 3.41296800 2.77436300

H 5.40928000 5.03416200 0.91588100

C 3.04899000 -0.48659600 -1.43954100

C 4.44860400 -0.51864100 -1.58396200

C 2.24310400 -1.11178600 -2.40267500

C 5.02680900 -1.20029500 -2.65786900

H 5.08201400 0.00430000 -0.86342400

C 2.82444100 -1.79665600 -3.47337200

H 1.15755900 -1.05655200 -2.30937400

C 4.21593700 -1.84686700 -3.59896500

H 6.11383700 -1.22172000 -2.76390200

H 2.18531700 -2.28901800 -4.20874800

H 4.67121900 -2.37946600 -4.43705600

C 2.54088200 -0.73366400 1.44476800

C 1.61485600 -0.64788900 2.49941600

C 3.59425300 -1.65867200 1.52715600

C 1.71902400 -1.49441700 3.60706500

H 0.78927200 0.06543100 2.43102800

C 3.69966600 -2.50111900 2.63805000

H 4.30994400 -1.74987900 0.70877400

C 2.75870700 -2.42804300 3.67331100

H 0.97115200 -1.43723000 4.40034400

H 4.51270400 -3.22915900 2.68852500

H 2.83460200 -3.10304200 4.52873300

Pd -0.06951700 0.82507900 -0.18848400

P -1.00812600 -1.40242700 -0.05691000

C -0.05255800 -2.87639700 -0.64124700

C -0.30897100 -3.44271600 -1.90312900

C 1.04076800 -3.35198500 0.10593700

C 0.52354100 -4.44484600 -2.41135900

H -1.15884900 -3.09620600 -2.49419000

C 1.86933800 -4.35268000 -0.40614800

H 1.24314700 -2.94435100 1.09602700

C 1.61985000 -4.89694500 -1.67011400

H 0.31162900 -4.87279900 -3.39400600

H 2.71691900 -4.70023700 0.18860300

H 2.27378900 -5.67301100 -2.07362100

C -1.48710100 -1.81935600 1.67849700

C -1.41831800 -3.12158500 2.20144900

C -1.93525900 -0.76769200 2.49937100

C -1.77117800 -3.36465800 3.53313400

H -1.08228400 -3.94823800 1.57289600

C -2.29256000 -1.01876600 3.82660000

H -2.00850700 0.24184800 2.08589700

C -2.20390000 -2.31464900 4.34944800

H -1.70790400 -4.38005500 3.93123600

H -2.64146700 -0.19554800 4.45392100

H -2.47716500 -2.50655600 5.38968100

C -2.57799900 -1.58840600 -1.01052500

C -3.56489700 -2.50749900 -0.62191100

C -2.75840300 -0.82891500 -2.17819600

C -4.71948000 -2.66604600 -1.39553700

H -3.43866500 -3.09175100 0.29190500

C -3.90962400 -0.99609700 -2.95266600

H -2.00765500 -0.08786900 -2.45918900

C -4.89189000 -1.91305000 -2.56226500

H -5.48809400 -3.37585100 -1.08139000

H -4.04853500 -0.39202000 -3.85148300

H -5.79812500 -2.03110200 -3.16046000

***trans*-5**

C -0.45351700 -1.57516900 -0.32562400

C -0.80434900 -2.75243000 -0.17807000

C 0.68631100 2.32222100 -0.22149200

C 0.91195100 3.15422200 -1.33603400

C 0.87250100 2.88796100 1.05908500

C 1.30224200 4.49054600 -1.18370500

H 0.78106700 2.75496200 -2.34566700

C 1.26425500 4.22530900 1.21872600

H 0.70844800 2.27574600 1.95148400

C 1.48024700 5.03295200 0.09473400

H 1.46905100 5.11197000 -2.06810800

H 1.40058000 4.63634100 2.22324600

H 1.78544300 6.07564400 0.21475400

Pd 0.10516400 0.35876300 -0.37844100

C -1.21650000 -4.11156200 -0.05540500

C -2.58968700 -4.44925400 -0.04503000

C -0.26329300 -5.15031000 0.05213100

C -2.99042800 -5.78132400 0.06784700

H -3.32755200 -3.64947900 -0.13193300

C -0.67433700 -6.47939800 0.16429000

H 0.79733800 -4.89255800 0.04106100

C -2.03738200 -6.80275300 0.17291000

H -4.05574100 -6.02595900 0.07379400

H 0.07483500 -7.27112000 0.24603700

H -2.35500100 -7.84432800 0.26151100

P -2.13106400 0.98271500 -0.16317200

P 2.34331100 -0.25252600 -0.17230300

C -2.75945100 2.58216900 -0.83787400

C -2.17434500 3.80101900 -0.44123600

C -3.79300700 2.59991600 -1.79148400

C -2.62550500 5.00713400 -0.98219200

H -1.36332400 3.81205800 0.28696300

C -4.23280600 3.81100800 -2.33524000

H -4.25766500 1.66638300 -2.11228900

C -3.65234900 5.01718500 -1.93214100

H -2.15697300 5.94064700 -0.66366500

H -5.03545400 3.80717300 -3.07620100

H -3.99732200 5.96195500 -2.35859100

C -3.35649300 -0.24162600 -0.79011600

C -4.56529200 -0.47758700 -0.11769200

C -3.07758900 -0.92962700 -1.98227900

C -5.48643100 -1.39480500 -0.63493300

H -4.78260100 0.04809600 0.81454400

C -4.00556300 -1.83462700 -2.50238800

H -2.12091800 -0.77085400 -2.48295500

C -5.20953600 -2.07071100 -1.82820300

H -6.42168400 -1.58149800 -0.10230100

H -3.77803400 -2.37370100 -3.42422700

H -5.92755900 -2.79032800 -2.22815100

C -2.46070800 1.03202600 1.65335600

C -3.01682900 2.13661500 2.31431300

C -2.10263000 -0.11325500 2.39169000

C -3.19644700 2.10363500 3.70280100

H -3.31307000 3.02266200 1.75082900

C -2.29388600 -0.14156300 3.77399300

H -1.68275900 -0.97828000 1.86957900

C -2.83344300 0.96985100 4.43470300

H -3.62542400 2.97054100 4.21054600

H -2.01793800 -1.03624800 4.33713700

H -2.97408500 0.94916300 5.51784100

C 2.76155300 -0.17688700 1.62063100

C 1.74172600 -0.48640500 2.53807200

C 4.03959700 0.16245200 2.09381700

C 1.99672900 -0.45019200 3.91044300

H 0.74963300 -0.75055200 2.16459900

C 4.28900600 0.19803700 3.46995800

H 4.83661400 0.40708600 1.38883500

C 3.26976000 -0.10504900 4.37897600

H 1.19385300 -0.68272100 4.61347800

H 5.28392900 0.46771500 3.83152600

H 3.46700600 -0.06848200 5.45280600

C 2.74853600 -1.98044300 -0.67008500

C 2.15397200 -2.49869800 -1.83319000

C 3.63637000 -2.77655400 0.06819300

C 2.46155300 -3.79165300 -2.26226600

H 1.43060300 -1.89401800 -2.38305900

C 3.92981200 -4.07709500 -0.35652900

H 4.09036500 -2.38770400 0.98176500

C 3.34790200 -4.58396100 -1.52304500

H 1.98858800 -4.18985100 -3.16220300

H 4.61444300 -4.69509400 0.22881000

H 3.57525300 -5.60098600 -1.85069300

C 3.65386600 0.74374900 -1.00632300

C 4.38595300 0.21647000 -2.08383900

C 3.88924900 2.07145200 -0.59958000

C 5.33417900 1.00419500 -2.74486800

H 4.22095100 -0.81289600 -2.40666600

C 4.84312200 2.84881300 -1.25935100

H 3.32705700 2.49963600 0.23132300

C 5.56533300 2.32070300 -2.33527100

H 5.89614000 0.58140600 -3.58077100

H 5.01031300 3.87741900 -0.93306200

H 6.30682500 2.93390700 -2.85261300

**11**

Pd 1.81376900 0.11195100 0.22457100

C 0.01882700 -0.18003600 1.07571000

C -0.90339200 -0.68268700 1.78199700

C -2.64603900 1.57051000 1.09757400

C -2.57280700 2.80489200 0.42784300

C -3.16709900 1.53245200 2.39805800

C -3.00057600 3.98160300 1.05681400

H -2.18889100 2.86013700 -0.59445900

C -3.59671300 2.71210600 3.02120300

H -3.26126300 0.58830300 2.93354200

C -3.51506200 3.93968500 2.35695900

H -2.93073500 4.93019100 0.51849600

H -4.00550500 2.66184800 4.03417200

H -3.84982000 4.85686100 2.84724300

Pd -1.90331900 0.07419400 -0.02627700

C -1.56256000 -1.32068200 2.88563800

C -0.89248700 -1.41235000 4.12442600

C -2.85264400 -1.87701200 2.76784500

C -1.49557200 -2.05785000 5.20409100

H 0.10389300 -0.97798500 4.21822000

C -3.45509400 -2.50850300 3.85525500

H -3.37695800 -1.78837800 1.81535600

C -2.77710300 -2.60882000 5.07642900

H -0.96277000 -2.12841600 6.15529800

H -4.46012700 -2.92239500 3.74675900

H -3.24742500 -3.10687200 5.92722700

P 1.80027800 2.46079200 0.40212800

P -4.04654300 -0.10145600 -0.93983800

P 2.27673900 -2.19373600 0.28997200

C 3.62179300 0.22196500 -0.57198100

C 4.80067200 0.22239000 -0.93753600

C 6.18143700 0.24066300 -1.28619700

C 7.03380400 -0.80559700 -0.86509600

C 6.73434600 1.31075700 -2.02460700

C 8.39391400 -0.77691900 -1.17529800

H 6.61017400 -1.62872300 -0.28733100

C 8.09571700 1.32912100 -2.33170600

H 6.07989900 2.12499800 -2.33931300

C 8.93208500 0.28766300 -1.90995500

H 9.04047500 -1.59200100 -0.84050100

H 8.50900500 2.16427600 -2.90287000

H 9.99737300 0.30602200 -2.15162700

I -0.69011400 -1.08413700 -2.24944300

C -4.33457100 -1.77057800 -1.67178400

C -5.25006400 -1.98088500 -2.71303000

C -3.64357400 -2.86504900 -1.12476400

C -5.47307500 -3.27373300 -3.19783700

H -5.78032800 -1.13601200 -3.15626800

C -3.87836000 -4.15579300 -1.60306100

H -2.90452600 -2.69465800 -0.33902900

C -4.79237900 -4.36195600 -2.64225000

H -6.17901700 -3.42885200 -4.01680600

H -3.32876700 -4.99890000 -1.17867000

H -4.96598400 -5.36959600 -3.02649900

C -4.52004100 1.09400000 -2.25721000

C -3.50128400 1.61144800 -3.07307000

C -5.85461100 1.47491000 -2.48643200

C -3.81661600 2.49272400 -4.11066400

H -2.46707600 1.30783100 -2.89516200

C -6.16205500 2.36191500 -3.52216200

H -6.65196300 1.08364600 -1.85128200

C -5.14412400 2.87089600 -4.33652200

H -3.01958000 2.88632100 -4.74494600

H -7.20037200 2.65534400 -3.69264900

H -5.38668700 3.56374900 -5.14557000

C -5.42248800 0.02849200 0.28703100

C -6.02055100 -1.12359700 0.82488400

C -5.81557600 1.29218500 0.76592000

C -6.99090600 -1.01352700 1.82634300

H -5.73223300 -2.11107800 0.45947900

C -6.79527600 1.39624600 1.75524200

H -5.34526600 2.19665400 0.37436800

C -7.38226300 0.24525300 2.29237400

H -7.44624800 -1.91790900 2.23656000

H -7.08528400 2.38430100 2.11858600

H -8.14197100 0.32961600 3.07275700

C 0.69353600 3.26343400 -0.82474200

C 0.13505900 4.53333700 -0.60255200

C 0.45172800 2.59245400 -2.03362300

C -0.65605000 5.12727400 -1.58965400

H 0.30495100 5.04685300 0.34578100

C -0.33843300 3.19416400 -3.01744500

H 0.86437300 1.59458400 -2.19683900

C -0.89476000 4.45858800 -2.79673200

H -1.09557500 6.11140800 -1.41187300

H -0.52284300 2.66079400 -3.95221700

H -1.52353900 4.92138200 -3.56056600

C 3.89567600 -2.46233300 1.14215000

C 4.69938800 -3.57938000 0.86885800

C 4.31679300 -1.52754400 2.10285300

C 5.90960300 -3.75647300 1.54864800

H 4.39220800 -4.30315700 0.11209700

C 5.52162100 -1.71129900 2.78500000

H 3.70251000 -0.64399600 2.29315200

C 6.32240400 -2.82513400 2.50720400

H 6.53430800 -4.62317700 1.32086900

H 5.84325700 -0.97425600 3.52407900

H 7.27135100 -2.96175900 3.03065100

C 1.12226400 -3.26025500 1.25508400

C -0.12156800 -3.60998600 0.69594300

C 1.43828300 -3.68190800 2.55555300

C -1.02168100 -4.38305900 1.43081700

H -0.38072200 -3.26452600 -0.30797200

C 0.52468000 -4.44936700 3.28762100

H 2.39882200 -3.41465200 2.99988200

C -0.70443400 -4.80298900 2.72827800

H -1.98757000 -4.64740300 0.99511400

H 0.77681700 -4.76434900 4.30246500

H -1.42183800 -5.38950000 3.30544300

C 3.43766100 3.24899500 0.09192500

C 4.52696400 2.84619300 0.88606400

C 3.62818800 4.21999700 -0.89971700

C 5.78352600 3.42045200 0.69878300

H 4.39001800 2.06701000 1.63849600

C 4.89390600 4.79116900 -1.08787500

H 2.79294700 4.53169800 -1.52906100

C 5.96953100 4.39598600 -0.28954600

H 6.62619100 3.09010600 1.30909300

H 5.03404800 5.54587900 -1.86517300

H 6.95799400 4.83418200 -0.44397100

C 2.44117100 -3.07181700 -1.31920300

C 2.80505700 -2.35335800 -2.46918100

C 2.23036400 -4.45956700 -1.40418900

C 2.96756800 -3.02050900 -3.68505400

H 2.95987100 -1.27604400 -2.40374400

C 2.39105200 -5.11960900 -2.62621700

H 1.93016800 -5.02454100 -0.51947500

C 2.76063300 -4.40144300 -3.76775000

H 3.24646400 -2.45252700 -4.57491100

H 2.22165700 -6.19725000 -2.68400400

H 2.88032900 -4.91742600 -4.72325600

C 1.30898400 3.20492300 2.01888200

C 1.95620300 4.35102800 2.51768300

C 0.23306700 2.65343700 2.73322000

C 1.52729700 4.93233000 3.71500400

H 2.79455300 4.78861000 1.97307500

C -0.19110500 3.24141600 3.92722500

H -0.27385300 1.76674900 2.35446400

C 0.45401200 4.37944700 4.42174400

H 2.03675600 5.82120600 4.09448600

H -1.03423200 2.80310300 4.46342200

H 0.12191200 4.83589900 5.35724100

**12**

Pd 1.78881000 0.00085500 -0.17770700

C 0.20413200 1.14720900 -0.93079100

C -0.07706300 2.16641900 -1.59469300

C -6.25379100 -2.21291700 0.53615900

C -5.96362600 -1.07405500 2.66653600

C -7.50246100 -2.67214300 0.95762000

H -5.86454500 -2.47976200 -0.44820900

C -7.21386500 -1.53825100 3.07735300

H -5.35066900 -0.45806300 3.32659100

C -7.98984800 -2.33707700 2.22738900

H -8.09969500 -3.29900100 0.29064400

H -7.58625300 -1.27637900 4.07096900

H -8.96780900 -2.69846400 2.55378100

Pd -1.28415400 -0.02466500 -0.00486600

C -0.34256300 3.38329000 -2.27495200

C 0.58978400 4.44431500 -2.19364200

C -1.53083400 3.55935500 -3.01655500

C 0.32748500 5.65057000 -2.84310500

H 1.51582100 4.29425000 -1.63509800

C -1.78463500 4.77576200 -3.64904300

H -2.24530000 2.73887500 -3.07851000

C -0.85980700 5.82451200 -3.56669100

H 1.05596900 6.46250400 -2.78311000

H -2.71133600 4.90373800 -4.21336600

H -1.06184100 6.77412300 -4.06734500

P -1.72827600 -1.29332500 -1.94120900

P -1.52996000 1.57089000 1.67574900

P 3.78234800 -0.98513000 0.51136100

C 2.85469200 1.53517000 -0.94927000

C 3.06301800 1.70263500 -2.32850400

C 3.41739500 2.48132400 -0.07606700

C 3.82909700 2.76931500 -2.81849600

H 2.62015500 1.00323800 -3.03854700

C 4.17566100 3.55310600 -0.56333800

H 3.28277400 2.37653400 1.00156100

C 4.38940700 3.70094600 -1.93846200

H 3.97619100 2.87569600 -3.89687900

H 4.61200600 4.26605800 0.14166600

H 4.98682500 4.53251000 -2.31984300

I 0.50839300 -2.19471800 1.09475600

C -5.46557700 -1.40399500 1.38603300

C -4.18555800 -0.94489300 0.96179600

C -3.04899800 -0.62129100 0.60916900

C 5.30565500 0.04683300 0.35592400

C 5.79347400 0.32016800 -0.93531800

C 5.92030500 0.64585000 1.46494400

C 6.86895800 1.18917000 -1.11170600

H 5.31319200 -0.13458300 -1.80359800

C 7.00244100 1.51515600 1.28297400

H 5.55216100 0.44117700 2.47167300

C 7.47392600 1.79318000 -0.00212500

H 7.22612100 1.40817500 -2.11987600

H 7.47437000 1.97753900 2.15308500

H 8.31117000 2.48064600 -0.14241500

C 4.28256400 -2.52729900 -0.36589400

C 5.61444100 -2.98037200 -0.29040900

C 3.34247100 -3.26445700 -1.10246200

C 5.99109600 -4.16874900 -0.92035600

H 6.35787500 -2.39570900 0.25611600

C 3.72626600 -4.45596000 -1.72690500

H 2.31289300 -2.91108800 -1.18215100

C 5.04403400 -4.91249600 -1.63431300

H 7.02586700 -4.51254200 -0.85412000

H 2.98474500 -5.02400100 -2.29088900

H 5.33717700 -5.84404800 -2.12428400

C 3.75444400 -1.40149700 2.30820700

C 3.10082400 -0.49434500 3.16005000

C 4.35237700 -2.54908900 2.84560200

C 3.06345700 -0.72516700 4.53582700

H 2.60123400 0.37998300 2.73604700

C 4.30133100 -2.78227000 4.22435900

H 4.83947400 -3.27329300 2.19084700

C 3.66248100 -1.87157600 5.07121200

H 2.54744400 -0.01816100 5.18906900

H 4.75794500 -3.68544800 4.63548700

H 3.62109400 -2.06015600 6.14638300

C -2.84907000 -2.74347500 -1.75345500

C -2.78159100 -3.52089800 -0.58550400

C -3.74528800 -3.09759000 -2.77548600

C -3.60374400 -4.64140000 -0.44646300

H -2.09363800 -3.23579000 0.21161200

C -4.56983500 -4.21831700 -2.62712000

H -3.80570100 -2.49539000 -3.68432500

C -4.49981500 -4.99109400 -1.46323900

H -3.55425400 -5.23299500 0.46977300

H -5.26902500 -4.48400300 -3.42333700

H -5.14981200 -5.86109100 -1.34457100

C -0.29856800 -1.97657100 -2.88180100

C -0.22087600 -3.34404600 -3.19280300

C 0.74299400 -1.11901900 -3.28206200

C 0.87139500 -3.83958700 -3.91258000

H -1.01296700 -4.02323100 -2.87532400

C 1.82977500 -1.62148600 -4.00198000

H 0.69528900 -0.05955400 -3.02597500

C 1.89612300 -2.98146100 -4.32200300

H 0.91829500 -4.90472700 -4.15116700

H 2.63431700 -0.94817700 -4.30481900

H 2.75225500 -3.37279500 -4.87540700

C -2.64293700 -0.22442400 -3.13731000

C -2.16518900 0.10667800 -4.41346100

C -3.87833600 0.29553100 -2.70253700

C -2.90831900 0.95778600 -5.24136000

H -1.21555400 -0.29745300 -4.76674700

C -4.61835000 1.13541200 -3.53681200

H -4.25227500 0.02905300 -1.70973000

C -4.13255900 1.47392900 -4.80753200

H -2.52315400 1.21714800 -6.22985700

H -5.57822800 1.52670900 -3.19183000

H -4.70850500 2.13757300 -5.45641800

C -0.03369200 2.46317300 2.28850200

C 0.48675400 3.53290800 1.53758200

C 0.58431300 2.10540000 3.49768600

C 1.58580500 4.25290900 2.00889100

H 0.02213300 3.81346700 0.59222400

C 1.69573500 2.82169500 3.95603100

H 0.19105200 1.27683500 4.08954100

C 2.19409700 3.90051700 3.21879400

H 1.97648700 5.08202000 1.41572600

H 2.16649300 2.54047400 4.90082300

H 3.05601800 4.46312000 3.58452500

C -2.31114300 0.94147200 3.22275400

C -3.24138400 1.68945500 3.95909500

C -1.94114500 -0.34056600 3.66453400

C -3.78749900 1.16289200 5.13498800

H -3.56059700 2.67229000 3.60982700

C -2.48247200 -0.85572100 4.84451700

H -1.24500400 -0.93862600 3.07067200

C -3.40629400 -0.10649600 5.58233600

H -4.51945400 1.74692700 5.69768800

H -2.19211400 -1.85476400 5.17644200

H -3.83814900 -0.51628500 6.49833600

C -2.58971700 2.95618200 1.08054400

C -2.62594300 4.19669600 1.74526400

C -3.35604700 2.77583000 -0.07961700

C -3.42731900 5.23135000 1.25588000

H -2.01227200 4.36113300 2.63368100

C -4.15190600 3.81474000 -0.56836300

H -3.30563300 1.82018200 -0.60238600

C -4.19081700 5.04266600 0.09779000

H -3.44634900 6.19183700 1.77572300

H -4.72812400 3.66366000 -1.48350300

H -4.80565800 5.85817500 -0.28960100

**13**

Pd -2.00879800 -0.19715300 -0.17668400

C -0.43581800 -1.21850200 0.43346700

C 0.41497300 -1.96734400 0.99046700

C 5.98667100 2.29912000 -1.99520100

C 5.26168100 3.73726000 -0.17029100

C 6.98772500 3.21805800 -2.31401900

H 5.86104900 1.38509500 -2.57850000

C 6.26498200 4.64961700 -0.49853200

H 4.57681400 3.93218700 0.65687900

C 7.13271500 4.39493600 -1.56853400

H 7.65893900 3.01630100 -3.15240800

H 6.37044800 5.56821100 0.08389700

H 7.91710900 5.11184200 -1.82193800

Pd 1.61350500 -0.28691900 0.13682900

C 0.91575800 -3.12729300 1.67320300

C 0.01346300 -4.18149700 1.95015100

C 2.26243800 -3.25402500 2.06365500

C 0.46606300 -5.33308100 2.59387400

H -1.03316800 -4.07250500 1.65793200

C 2.70352600 -4.41169700 2.70587000

H 2.95514400 -2.44061600 1.84877800

C 1.81066100 -5.45617700 2.97163500

H -0.23745200 -6.14257000 2.80309900

H 3.75339500 -4.49693700 2.99412300

H 2.15873300 -6.36288600 3.47163400

P 2.03760000 -1.56335500 -1.79388700

P 1.62792900 0.89303300 2.15923100

P -4.04116000 0.92292500 -0.42054100

C -3.02103700 -1.75320800 0.62163400

C -3.61205800 -2.67088300 -0.26700200

C -3.14759100 -1.98445000 1.99962800

C -4.31490600 -3.78494100 0.21395200

H -3.53712900 -2.51329200 -1.34489900

C -3.85051000 -3.09604700 2.48127600

H -2.69574600 -1.28710400 2.70165900

C -4.43794200 -4.00285400 1.59158000

H -4.77142900 -4.48231000 -0.49490000

H -3.94007600 -3.25093100 3.56053600

H -4.98704000 -4.87028400 1.96660600

I -0.65600000 1.49649400 -1.88491700

C 5.10996600 2.54586300 -0.91520800

C 4.07513400 1.62231000 -0.58801900

C 3.14160300 0.85865200 -0.32915400

C -5.23496800 0.69257200 0.97307900

C -5.93919500 -0.51889500 1.10042900

C -5.36375200 1.66555500 1.97814500

C -6.75414200 -0.74784400 2.21084500

H -5.83728900 -1.29417100 0.33959300

C -6.17583900 1.42833800 3.09211600

H -4.83106700 2.61420700 1.89182500

C -6.87209900 0.22166800 3.21269400

H -7.28511900 -1.69814600 2.29652700

H -6.26718700 2.19547200 3.86494100

H -7.50562400 0.03776500 4.08358900

C -4.97360700 0.39831200 -1.91780000

C -6.37208300 0.49865700 -2.02063100

C -4.23528600 -0.13689700 -2.98822600

C -7.02168000 0.07323200 -3.18342300

H -6.95283500 0.89585200 -1.18495800

C -4.89062100 -0.55967900 -4.14784400

H -3.14810100 -0.22008300 -2.90507100

C -6.28241100 -0.45609300 -4.24762300

H -8.10890300 0.15132800 -3.25615700

H -4.30861400 -0.97656600 -4.97286600

H -6.79351900 -0.79237300 -5.15277100

C -3.89858600 2.75886300 -0.51819800

C -2.87803900 3.36834800 0.23199600

C -4.77184900 3.55528100 -1.27193400

C -2.74521700 4.75825900 0.24525100

H -2.17740100 2.74167800 0.78705700

C -4.62774000 4.94700500 -1.26800000

H -5.55629100 3.09178700 -1.87339900

C -3.62020200 5.54995500 -0.50749500

H -1.94297900 5.21868100 0.82668000

H -5.30471100 5.56158200 -1.86578300

H -3.51036500 6.63689900 -0.51019600

C 2.93554500 -0.76569600 -3.18838000

C 2.75299200 0.59976700 -3.45689700

C 3.79879100 -1.52772400 -3.99355600

C 3.42841300 1.19481800 -4.52616900

H 2.08960700 1.18981100 -2.82422400

C 4.47557200 -0.92476800 -5.05774400

H 3.95094900 -2.58852200 -3.78300700

C 4.29161600 0.43687600 -5.32465700

H 3.28787200 2.25951300 -4.72261100

H 5.14958100 -1.52088100 -5.67716100

H 4.82569100 0.90781200 -6.15339000

C 0.55892800 -2.33124300 -2.56243900

C 0.05196400 -1.79332500 -3.75751000

C -0.15624200 -3.35161200 -1.90689300

C -1.14102800 -2.28212600 -4.29652800

H 0.58059500 -0.98305900 -4.26045300

C -1.33580700 -3.84963500 -2.46386000

H 0.19597400 -3.75432500 -0.95763600

C -1.83455400 -3.31307400 -3.65562600

H -1.52831300 -1.84876200 -5.22141000

H -1.87921300 -4.64058900 -1.94411700

H -2.76905400 -3.69066200 -4.07735700

C 3.19326900 -2.90208600 -1.27203000

C 2.96472900 -4.27204900 -1.46145100

C 4.36300400 -2.47875300 -0.61085000

C 3.87736900 -5.20894000 -0.96195900

H 2.07748400 -4.61413200 -1.99499000

C 5.27639300 -3.41706300 -0.13091900

H 4.54247700 -1.40863400 -0.47499200

C 5.02818500 -4.78638400 -0.29240300

H 3.68223200 -6.27476500 -1.09836100

H 6.17979300 -3.07832100 0.38161500

H 5.73358400 -5.52173000 0.10097900

C 0.09919200 0.75002600 3.17164500

C -0.16286700 -0.43379700 3.88473000

C -0.84891400 1.78504700 3.18002500

C -1.33447300 -0.55186200 4.63594400

H 0.55080100 -1.25971300 3.85979000

C -2.03845000 1.64392900 3.90107300

H -0.65344800 2.70689700 2.62988400

C -2.27819900 0.48280900 4.64123700

H -1.52297000 -1.47094200 5.19509300

H -2.77688700 2.44791900 3.88651800

H -3.20820400 0.37509300 5.20322100

C 1.88768500 2.70884400 1.99690800

C 2.58610800 3.44250200 2.96832500

C 1.34457800 3.36442200 0.88004000

C 2.73396900 4.82615100 2.82295800

H 3.02745900 2.93713600 3.82941600

C 1.48522800 4.74830400 0.74812200

H 0.82366100 2.79234700 0.10735900

C 2.18103100 5.48126000 1.71656000

H 3.28678900 5.39173400 3.57652400

H 1.06038500 5.24611600 -0.12621100

H 2.30200800 6.56132800 1.60504200

C 2.98041300 0.31738300 3.27428600

C 2.85528000 0.35426300 4.67452100

C 4.17832900 -0.14417600 2.70165000

C 3.90821300 -0.08068900 5.48565800

H 1.93292200 0.71547900 5.13305300

C 5.22716900 -0.57465900 3.51746800

H 4.28107600 -0.15225700 1.61495700

C 5.09339800 -0.54986300 4.91000500

H 3.79819700 -0.05369800 6.57204700

H 6.15164500 -0.93382100 3.05979800

H 5.91160900 -0.89440600 5.54642600

**14**

Pd -2.08856800 -0.42427100 0.43042300

C -0.38827700 -1.05037000 1.28578900

C 0.50745000 -1.50360800 2.02018600

C 6.09164900 1.77072700 1.67192900

C 6.85340200 0.49379400 -0.25332000

C 7.40801700 2.15480100 1.93021100

H 5.27494000 2.11272400 2.31015700

C 8.16730100 0.87987200 0.01725300

H 6.62672500 -0.13993300 -1.11333700

C 8.45201900 1.71050000 1.10856900

H 7.62132100 2.80564600 2.78173100

H 8.97603500 0.53399200 -0.63116500

H 9.48134100 2.01176800 1.31563200

Pd 1.36315800 0.03839400 -0.28337100

C 1.42766600 -2.08244800 2.94912200

C 0.93712600 -2.95100100 3.95146100

C 2.81189900 -1.81086200 2.89964200

C 1.81187600 -3.52671800 4.87384000

H -0.13417200 -3.15821700 3.99055100

C 3.67525300 -2.38531100 3.83315800

H 3.20200000 -1.16121400 2.11439200

C 3.18350400 -3.24485500 4.82256100

H 1.41954400 -4.19723100 5.64251800

H 4.74396100 -2.16747900 3.77362200

H 3.86422600 -3.69613300 5.54834700

P 2.05027400 -1.93315100 -1.32435400

P 1.27746500 2.28578600 0.35616400

P -4.22938800 0.25708800 -0.25256800

C -2.90944600 -1.30054900 2.04217000

C -3.55586300 -2.54023400 1.88074600

C -2.85901500 -0.73523300 3.32769500

C -4.17073600 -3.17527600 2.96939700

H -3.60618600 -3.00819800 0.89452400

C -3.47616900 -1.36781100 4.41376700

H -2.33199200 0.20678300 3.49135700

C -4.14188000 -2.58681200 4.23893100

H -4.67851300 -4.13219400 2.81930700

H -3.43434300 -0.90366600 5.40297900

H -4.62509800 -3.07876200 5.08658300

I -0.83547000 0.30290300 -1.95017400

C 5.79401600 0.92993600 0.57407900

C 4.44535900 0.55808000 0.30715100

C 3.25890200 0.27948900 0.11519000

C -5.34135100 0.97081700 1.04109500

C -5.77215500 0.16965200 2.11639800

C -5.70475600 2.32849800 1.01430000

C -6.54089300 0.72300600 3.14330100

H -5.50884300 -0.88782100 2.15576300

C -6.46886000 2.87735900 2.04925100

H -5.38722100 2.96379200 0.18648000

C -6.88586800 2.07856100 3.11851600

H -6.85929900 0.08597000 3.97109500

H -6.74093000 3.93500000 2.01369100

H -7.48080900 2.50951500 3.92707800

C -5.15768800 -1.17213100 -0.95148400

C -6.53211300 -1.37656500 -0.75362800

C -4.42601500 -2.07972600 -1.73913600

C -7.16558900 -2.48237400 -1.33158600

H -7.10588400 -0.67550300 -0.14431000

C -5.06650600 -3.17482200 -2.32305700

H -3.35353900 -1.92505000 -1.88825300

C -6.43582200 -3.38112400 -2.11649900

H -8.23410200 -2.63985800 -1.16765300

H -4.48969700 -3.87045100 -2.93603300

H -6.93361200 -4.24363000 -2.56558500

C -4.30887900 1.52243300 -1.59266100

C -3.42118700 2.61046900 -1.52635700

C -5.23211800 1.44618800 -2.64668600

C -3.47203700 3.61964000 -2.49034200

H -2.68726500 2.66077400 -0.72182400

C -5.26793300 2.45096800 -3.61942200

H -5.91955300 0.60051200 -2.71196600

C -4.39285500 3.53985500 -3.54093100

H -2.77871800 4.46081800 -2.42135600

H -5.98421000 2.38145800 -4.44127700

H -4.42514000 4.32257600 -4.30245100

C 3.14455700 -1.52329500 -2.74406700

C 3.18741700 -0.19926000 -3.21012400

C 3.94901900 -2.50244500 -3.35472500

C 4.02065400 0.14025900 -4.27949800

H 2.58292900 0.56509000 -2.71568600

C 4.78162100 -2.15719100 -4.42291200

H 3.93366600 -3.52995900 -2.98365200

C 4.81784600 -0.83602900 -4.88605900

H 4.05185700 1.17563900 -4.62517500

H 5.40789000 -2.92050500 -4.89027200

H 5.47515900 -0.56807000 -5.71665500

C 0.69523900 -2.98236600 -2.00889400

C 0.67725800 -3.39367500 -3.35052400

C -0.37179200 -3.33757500 -1.16210500

C -0.38062000 -4.17307300 -3.83080700

H 1.47906700 -3.09738200 -4.02820000

C -1.41670100 -4.12817700 -1.64468800

H -0.40032700 -2.96823600 -0.13464300

C -1.42308300 -4.55023000 -2.97907000

H -0.38672000 -4.48429600 -4.87786500

H -2.23621400 -4.39823500 -0.97528100

H -2.24185600 -5.16758000 -3.35663400

C 3.05365300 -3.07799000 -0.28521800

C 2.46082400 -4.13985300 0.41663900

C 4.42707700 -2.82218900 -0.11627000

C 3.22923800 -4.93148900 1.27428100

H 1.39743300 -4.35041400 0.30580000

C 5.19044100 -3.62196900 0.73597000

H 4.89279100 -1.99090100 -0.64480200

C 4.59323500 -4.67580300 1.43637500

H 2.75157900 -5.74164700 1.82835600

H 6.25438700 -3.40839600 0.86067500

H 5.18820900 -5.29021400 2.11552600

C -0.33631000 3.09726800 0.73338100

C -1.19178000 2.41090100 1.61429400

C -0.68524800 4.38258100 0.28887800

C -2.39327400 2.99451600 2.02658100

H -0.90931900 1.41880200 1.97589800

C -1.88660700 4.96510200 0.71106300

H -0.02039600 4.93328300 -0.37906900

C -2.74269400 4.27189900 1.57386300

H -3.06378900 2.44333300 2.68843400

H -2.15396200 5.96400200 0.35876900

H -3.68658800 4.72087700 1.88967900

C 2.04130000 3.21063600 -1.03351100

C 3.42241100 3.47382400 -1.04274200

C 1.28150100 3.46249800 -2.19193000

C 4.02879600 3.99464300 -2.18906700

H 4.02952000 3.24763700 -0.16598700

C 1.89485100 3.98675600 -3.33273000

H 0.21722500 3.21910000 -2.21136300

C 3.26907400 4.25340900 -3.33499500

H 5.10393500 4.18633200 -2.18490200

H 1.29568500 4.17549100 -4.22629600

H 3.74722400 4.65763800 -4.23034600

C 2.20508300 2.70872900 1.88786800

C 2.70551200 3.99770600 2.12992600

C 2.32487400 1.71636300 2.87212300

C 3.35401200 4.27611500 3.33734900

H 2.59717700 4.78064800 1.37595200

C 2.96104300 2.00221000 4.08233200

H 1.92268400 0.72070700 2.68168000

C 3.48462900 3.27988700 4.31268500

H 3.75613400 5.27575600 3.51705700

H 3.05694900 1.21723400 4.83528200

H 3.99327600 3.50184700 5.25368800

**TS^11/12^**

Pd -1.26438700 -0.03978100 -0.18436900

C 0.26689900 -0.20393500 1.20665700

C 0.34969500 -0.25186200 2.45154800

C 2.78907800 -1.55241100 0.60028300

C 2.97768500 -2.63001600 -0.28612300

C 3.22827200 -1.68230500 1.92796000

C 3.57542500 -3.82020000 0.15491900

H 2.64994300 -2.55418600 -1.32646500

C 3.83111300 -2.86819100 2.36243700

H 3.10238300 -0.85721500 2.63071800

C 4.00595100 -3.94253500 1.48010000

H 3.69976600 -4.64947400 -0.54668700

H 4.16523200 -2.95199200 3.40017100

H 4.47521700 -4.86744400 1.82447000

Pd 1.87313300 0.05543900 -0.18252700

C 0.41402900 -0.29159200 3.86621600

C -0.24976900 -1.31782700 4.57974100

C 1.10624300 0.70931200 4.58546300

C -0.22722300 -1.32857200 5.97368800

H -0.78400600 -2.08820800 4.02392000

C 1.13759400 0.67660500 5.97857900

H 1.59927400 1.50870500 4.03204800

C 0.46914500 -0.33726100 6.67801300

H -0.75019700 -2.12078100 6.51453800

H 1.67505600 1.45520500 6.52471100

H 0.48910000 -0.35333000 7.77007100

P -1.46033200 -2.34722400 -0.37994400

P 3.97665400 0.65724000 -0.91809400

P -2.23464200 1.80291900 0.95070600

C -2.81571100 0.16920700 -1.37164700

C -3.88578600 0.33036100 -1.96332000

C -5.15321600 0.47241000 -2.59741000

C -5.88898000 1.67458600 -2.48242000

C -5.72243200 -0.60256300 -3.31750600

C -7.15245900 1.79003600 -3.06427800

H -5.45061500 2.51231400 -1.93660200

C -6.98608300 -0.47693400 -3.89594400

H -5.16034400 -1.53395900 -3.40124600

C -7.70849800 0.71702300 -3.77245200

H -7.70743300 2.72638900 -2.96552300

H -7.41275800 -1.31913500 -4.44659700

H -8.69768400 0.81115300 -4.22650200

I 0.63586400 1.78617900 -1.95015400

C 4.12843700 2.49366500 -1.03471500

C 4.89938700 3.12808900 -2.01863700

C 3.44217100 3.27076500 -0.08461900

C 4.98645900 4.52416200 -2.04717500

H 5.41777800 2.53846700 -2.77638800

C 3.54183300 4.66299500 -0.10970700

H 2.81321500 2.77426800 0.65881400

C 4.31290800 5.29275800 -1.09342000

H 5.57890300 5.01128500 -2.82491700

H 2.99627100 5.26001800 0.62421100

H 4.37786500 6.38269700 -1.12310700

C 4.52289500 0.01175200 -2.55234500

C 3.54561700 -0.40193900 -3.47078800

C 5.88302700 -0.04837200 -2.91016400

C 3.92549800 -0.86074100 -4.73575800

H 2.49190000 -0.34801600 -3.18894800

C 6.25573000 -0.51286200 -4.17430500

H 6.64978700 0.26128300 -2.19685000

C 5.27709100 -0.91818700 -5.08932900

H 3.16127200 -1.17428400 -5.44997300

H 7.31317800 -0.55818100 -4.44437500

H 5.56977600 -1.28068600 -6.07748700

C 5.36570100 0.20344500 0.21498300

C 5.77821800 1.08430400 1.22765300

C 5.93830900 -1.07979600 0.14722200

C 6.74365800 0.68610400 2.15815700

H 5.34903800 2.08577800 1.29194400

C 6.90492900 -1.47033400 1.07515900

H 5.61139900 -1.78446000 -0.61869900

C 7.30838900 -0.59078500 2.08591800

H 7.05611900 1.38162900 2.94066000

H 7.33070800 -2.47408400 1.01471600

H 8.05996200 -0.90043700 2.81576500

C -0.31876500 -3.08834400 -1.61420200

C 0.17196800 -4.40180500 -1.52536800

C 0.04083100 -2.27974800 -2.70631100

C 1.02647400 -4.89585900 -2.51589500

H -0.10078400 -5.03145200 -0.67645000

C 0.87436800 -2.79014600 -3.70478600

H -0.32582500 -1.25086500 -2.76236300

C 1.37761700 -4.09225100 -3.60680900

H 1.41896800 -5.91198000 -2.43359400

H 1.13581000 -2.16243300 -4.55812100

H 2.04663500 -4.47932400 -4.37877400

C -3.61793400 0.94884500 1.81874200

C -4.93780700 1.02746500 1.34527900

C -3.30637700 0.03361500 2.84268400

C -5.93219600 0.21546700 1.90279600

H -5.18559900 1.70108400 0.52442500

C -4.30324300 -0.77007800 3.39835500

H -2.27674300 -0.06172900 3.19063800

C -5.62060500 -0.68239400 2.92899700

H -6.95275800 0.28004000 1.51874100

H -4.04286300 -1.47754700 4.18886600

H -6.39859500 -1.31963100 3.35547700

C -1.26931000 2.67072400 2.26051300

C -0.01245200 3.17796200 1.88420200

C -1.74730900 2.89493200 3.56036400

C 0.73985400 3.92235900 2.79511100

H 0.36445100 2.98353000 0.87626700

C -0.98193200 3.63130100 4.47219100

H -2.71605700 2.49395000 3.86503500

C 0.25694400 4.15276800 4.09015300

H 1.71399600 4.31766700 2.49821800

H -1.35835300 3.79502600 5.48436700

H 0.85123500 4.73039300 4.80197900

C -3.13956100 -2.91963200 -0.87918500

C -4.25365400 -2.32348300 -0.26051500

C -3.33274400 -3.94048100 -1.81948900

C -5.54236800 -2.76539400 -0.56172200

H -4.11048100 -1.50214700 0.44342200

C -4.62962100 -4.36948400 -2.12962000

H -2.47581600 -4.39797700 -2.31750300

C -5.73326500 -3.78915900 -1.49796300

H -6.39762100 -2.28790700 -0.08007300

H -4.77297400 -5.16036700 -2.86948200

H -6.74380900 -4.12139500 -1.74633500

C -3.02321300 3.22401200 0.08248800

C -2.79223000 3.43417700 -1.28418600

C -3.83080900 4.12370600 0.80121800

C -3.37258500 4.53298600 -1.92730900

H -2.17010700 2.73246100 -1.83879600

C -4.41917200 5.21076000 0.15090200

H -4.00384900 3.97044000 1.86933200

C -4.18984800 5.41643200 -1.21563400

H -3.18954400 4.68870200 -2.99248200

H -5.05327800 5.90079300 0.71234400

H -4.64784100 6.26877600 -1.72318400

C -1.17631100 -3.29705800 1.18300900

C -2.25087300 -3.76062900 1.96212700

C 0.13833700 -3.54200400 1.61754300

C -2.01027000 -4.47855600 3.13907300

H -3.27933700 -3.57803500 1.65009900

C 0.37278400 -4.27525500 2.78362400

H 0.98375100 -3.16774500 1.04347100

C -0.69970000 -4.74967300 3.54702200

H -2.85661700 -4.83734800 3.72966400

H 1.40315400 -4.45743200 3.09276100

H -0.51505000 -5.31930000 4.46085300

**TS^12/13^**

Pd 1.88613300 -0.01972800 -0.16174700

C 0.26260500 0.93216100 -0.99741400

C -0.05069000 1.81673600 -1.82601400

C -6.42891700 -2.02828900 0.59022000

C -6.31481700 -0.47298800 2.45696700

C -7.70953900 -2.40952400 0.99255000

H -5.95967900 -2.48461500 -0.28303800

C -7.59601300 -0.86184300 2.85073000

H -5.75803000 0.27384600 3.02479200

C -8.30057500 -1.82889400 2.12214000

H -8.25061000 -3.16794800 0.42107700

H -8.04917400 -0.40742300 3.73548200

H -9.30346000 -2.12978700 2.43402000

Pd -1.42672400 0.03326200 -0.01603200

C -0.29945800 2.87794200 -2.73559900

C 0.58065600 3.98501100 -2.75208900

C -1.38843000 2.84474100 -3.63258000

C 0.36746000 5.02978400 -3.65053400

H 1.43813000 3.99161100 -2.07642400

C -1.59909500 3.90495500 -4.51370800

H -2.06198300 1.98879400 -3.62078100

C -0.72450900 4.99909900 -4.52819700

H 1.05931000 5.87497500 -3.66592000

H -2.44858800 3.87099600 -5.19987700

H -0.88916700 5.82361400 -5.22586400

P -1.81497300 -1.54812300 -1.69949700

P -1.56120200 1.78403200 1.51358500

P 3.88381000 -0.90622300 0.62873600

C 2.92047400 1.32788500 -1.26323100

C 3.00489400 1.26588300 -2.66511500

C 3.57660300 2.39196500 -0.61795900

C 3.72916000 2.22017500 -3.39258300

H 2.49227500 0.47220300 -3.20929000

C 4.29195800 3.35456800 -1.34088300

H 3.55977600 2.45572000 0.47095800

C 4.37408700 3.27253400 -2.73546200

H 3.77518600 2.14400100 -4.48256900

H 4.80291900 4.16011700 -0.80598900

H 4.93619400 4.01818400 -3.30277600

I 0.53411700 -1.83530500 1.51703400

C -5.71199900 -1.05097300 1.31727200

C -4.39971100 -0.67224200 0.91191200

C -3.23970100 -0.41238000 0.58290200

C 5.41149700 0.04139500 0.21353800

C 5.82551500 0.06915200 -1.13130400

C 6.09067700 0.82174300 1.15979300

C 6.89341300 0.87498400 -1.52204700

H 5.29039500 -0.52456000 -1.87497300

C 7.16395600 1.62871200 0.76278400

H 5.77739400 0.80845100 2.20531900

C 7.56253500 1.66192400 -0.57563700

H 7.19273100 0.90345400 -2.57158300

H 7.68612700 2.23552400 1.50632100

H 8.39244900 2.30143600 -0.88479300

C 4.31659200 -2.59915400 0.04173800

C 5.64516900 -3.05973500 0.12599200

C 3.32356800 -3.44363300 -0.47856100

C 5.96842100 -4.35621700 -0.28094000

H 6.42722100 -2.39720500 0.50375400

C 3.65403300 -4.74281000 -0.87838500

H 2.29500900 -3.08782200 -0.56438400

C 4.97009100 -5.20272500 -0.77763300

H 7.00141500 -4.70444300 -0.21018600

H 2.87251500 -5.39323400 -1.27454100

H 5.22157700 -6.21836200 -1.09238300

C 3.92132100 -0.98085600 2.47030000

C 3.31623300 0.07723300 3.17220300

C 4.53099300 -2.02509200 3.17969600

C 3.34976200 0.09854300 4.56768800

H 2.79977700 0.87079000 2.62501400

C 4.54618200 -2.00610800 4.57819000

H 4.97814900 -2.86417700 2.64445100

C 3.96279300 -0.94283700 5.27343300

H 2.87686600 0.92357200 5.10212900

H 5.01149200 -2.82995100 5.12410000

H 3.97417200 -0.93097400 6.36579200

C -2.93577600 -2.94039100 -1.26247200

C -2.85430600 -3.50354700 0.02206200

C -3.83214400 -3.46998900 -2.20375100

C -3.66424900 -4.59127100 0.35528300

H -2.16316900 -3.07839900 0.75247900

C -4.64404800 -4.55722400 -1.86093600

H -3.90221800 -3.03066000 -3.20100900

C -4.56029500 -5.11855900 -0.58260700

H -3.60507500 -5.01883700 1.35814500

H -5.34507900 -4.96133000 -2.59486300

H -5.20055500 -5.96161400 -0.31306100

C -0.37060600 -2.40054400 -2.46258000

C -0.28941000 -3.80259500 -2.49364000

C 0.67160200 -1.63789100 -3.02071500

C 0.80715300 -4.42926200 -3.09433200

H -1.08287000 -4.40712000 -2.05293000

C 1.76437500 -2.27144300 -3.61742100

H 0.61972200 -0.54915300 -2.98511200

C 1.83406800 -3.66770400 -3.65936500

H 0.85544800 -5.52046800 -3.11719400

H 2.57035000 -1.67037600 -4.04321900

H 2.69416400 -4.15965700 -4.11814600

C -2.68737100 -0.71776900 -3.09945000

C -2.24407200 -0.77241400 -4.42935800

C -3.84763300 0.01653700 -2.78329300

C -2.94373000 -0.08558000 -5.42985800

H -1.35189800 -1.34508200 -4.68747100

C -4.54583300 0.68953900 -3.78775300

H -4.19494100 0.04866100 -1.74688800

C -4.09179500 0.64639000 -5.11320200

H -2.58503000 -0.12474500 -6.46073000

H -5.44461700 1.25508100 -3.53143800

H -4.63276200 1.18273000 -5.89613200

C -0.02895000 2.49547700 2.26396300

C 0.86566700 3.21085000 1.44681000

C 0.21318400 2.41653500 3.64520600

C 1.95874400 3.86967600 2.01499200

H 0.69697200 3.26441900 0.37075100

C 1.31798300 3.06796600 4.20417900

H -0.46932400 1.86345000 4.29121500

C 2.18917600 3.80147600 3.39387600

H 2.62957000 4.44365700 1.37314000

H 1.48453400 3.01347600 5.28254700

H 3.04328400 4.32099900 3.83399800

C -2.59433800 1.42159200 2.99381200

C -3.50033500 2.35034700 3.52365900

C -2.42509300 0.17276600 3.61806200

C -4.22567600 2.03645500 4.67940200

H -3.65293400 3.31234000 3.03137800

C -3.14412300 -0.12922500 4.77606300

H -1.73630200 -0.55770500 3.18576900

C -4.04481400 0.80122400 5.30961100

H -4.93704300 2.76047400 5.08336900

H -3.01039000 -1.10166400 5.25450700

H -4.61409700 0.55772800 6.20966000

C -2.31233500 3.25197600 0.69284800

C -2.14303900 4.55319100 1.19973900

C -3.04123900 3.06071500 -0.49139600

C -2.70122600 5.64360600 0.52697700

H -1.56066500 4.71480800 2.10951500

C -3.59259800 4.15451200 -1.16254900

H -3.14852600 2.05023500 -0.89093400

C -3.42287400 5.44647900 -0.65618400

H -2.56142900 6.65201200 0.92294500

H -4.13619600 3.99408100 -2.09567600

H -3.84232200 6.30285600 -1.18890200

**TS^13/14^**

Pd -2.10822900 -0.44958200 0.36402100

C -0.41806600 -1.07039200 1.22870300

C 0.50811400 -1.48034500 1.95941800

C 6.29078300 1.80364600 1.24670300

C 6.80257000 0.51011600 -0.75032800

C 7.62823300 2.19333200 1.32926400

H 5.56136400 2.14840900 1.98256100

C 8.13903500 0.90118800 -0.65399600

H 6.46688400 -0.13155100 -1.56809300

C 8.55894100 1.74318500 0.38387300

H 7.94800100 2.85264800 2.13997300

H 8.85896800 0.55102900 -1.39781400

H 9.60532000 2.04870100 0.45387600

Pd 1.36713800 -0.04933200 -0.06025500

C 1.38815700 -2.08102500 2.91570400

C 0.83243800 -2.88745300 3.93577200

C 2.78688500 -1.90075700 2.87158000

C 1.66070200 -3.49240100 4.88158400

H -0.25030000 -3.02465400 3.96870200

C 3.60383300 -2.50678900 3.82686800

H 3.22275800 -1.29604000 2.07390600

C 3.04844300 -3.30418900 4.83420900

H 1.21929200 -4.11414400 5.66432900

H 4.68488900 -2.36243400 3.77104800

H 3.69238900 -3.77991400 5.57754300

P 2.02120100 -1.96530100 -1.21525700

P 1.28294700 2.20558500 0.59198700

P -4.20780700 0.32720100 -0.32048100

C -2.97604800 -1.43805800 1.88753200

C -3.64446700 -2.64384300 1.60081600

C -2.93494000 -1.00214100 3.22296100

C -4.28512700 -3.37030500 2.61480300

H -3.69447400 -3.01079900 0.57270800

C -3.57979000 -1.72427300 4.23511400

H -2.39289700 -0.09169700 3.48576400

C -4.26389200 -2.90820900 3.93562500

H -4.80821300 -4.29800900 2.36603400

H -3.54463300 -1.35776300 5.26483700

H -4.76793200 -3.47008900 4.72570700

I -0.78695100 0.34438800 -1.95832600

C 5.85756900 0.95265500 0.20323900

C 4.48839800 0.57439600 0.10927900

C 3.28872000 0.29794400 0.02988300

C -5.34392100 0.94248200 1.00236100

C -5.82872300 0.05104500 1.97904800

C -5.67264600 2.30553200 1.10041000

C -6.61617800 0.52059600 3.03287600

H -5.59064500 -1.01153500 1.92125300

C -6.45687200 2.76982500 2.16163200

H -5.31240500 3.01076300 0.35036800

C -6.92747100 1.88101800 3.13293200

H -6.97649800 -0.18590400 3.78354200

H -6.70196100 3.83284700 2.22364100

H -7.53765100 2.24598400 3.96235800

C -5.15163400 -0.99409400 -1.18794800

C -6.54535700 -1.13430600 -1.09266700

C -4.41564600 -1.88307000 -1.99279900

C -7.19529500 -2.15672000 -1.79244400

H -7.12055800 -0.44863900 -0.46707400

C -5.07216600 -2.89506200 -2.69696200

H -3.32872300 -1.77972300 -2.06117000

C -6.46127200 -3.03632500 -2.59529000

H -8.27919100 -2.26479600 -1.70937800

H -4.49216700 -3.57734400 -3.32167400

H -6.97184600 -3.83416000 -3.13957600

C -4.18361500 1.72757200 -1.51914900

C -3.26690600 2.76892700 -1.29107900

C -5.04678200 1.79382200 -2.62257000

C -3.23198000 3.87381400 -2.14403100

H -2.57852500 2.70720200 -0.44727500

C -4.99488900 2.89411400 -3.48558700

H -5.75447000 0.98457100 -2.81335000

C -4.09275900 3.93599000 -3.24599200

H -2.51958100 4.67873900 -1.94962800

H -5.66382600 2.93573500 -4.34819700

H -4.05688400 4.79370900 -3.92174600

C 3.06966100 -1.50970900 -2.65718600

C 3.05020800 -0.18835600 -3.13275300

C 3.89597700 -2.46175600 -3.27985800

C 3.84960700 0.17509200 -4.21925700

H 2.42468400 0.55616800 -2.63597100

C 4.69706900 -2.09127500 -4.36424900

H 3.92262000 -3.48835600 -2.90728800

C 4.67530100 -0.77261000 -4.83387400

H 3.83350200 1.20827800 -4.57235200

H 5.34243800 -2.83374600 -4.83913000

H 5.30744500 -0.48382100 -5.67693900

C 0.64200000 -2.97652000 -1.91216800

C 0.58936100 -3.29020700 -3.27974000

C -0.40676300 -3.38957200 -1.06909200

C -0.48380100 -4.02705500 -3.79074300

H 1.37637900 -2.94833200 -3.95291000

C -1.47070000 -4.13277400 -1.58494500

H -0.41070500 -3.10013300 -0.01737600

C -1.51111800 -4.45651600 -2.94555100

H -0.51491700 -4.26128200 -4.85719900

H -2.27708800 -4.44529800 -0.91854000

H -2.34400700 -5.03879600 -3.34689500

C 3.05262000 -3.14677000 -0.24828600

C 2.48269100 -4.25099700 0.40566000

C 4.42259100 -2.87393300 -0.07471300

C 3.26952300 -5.06641300 1.22361100

H 1.42342100 -4.47748800 0.28592900

C 5.20427100 -3.69834600 0.73657600

H 4.86990700 -2.00944700 -0.56487800

C 4.62952800 -4.79321000 1.39179600

H 2.81040100 -5.91233400 1.73879900

H 6.26564800 -3.47366800 0.86365600

H 5.23969200 -5.42832200 2.03774500

C -0.31570700 2.97902100 1.09780200

C -1.18220100 2.21720000 1.90086800

C -0.64610300 4.31129400 0.79447200

C -2.38216700 2.76867900 2.36239400

H -0.91282200 1.19054200 2.15785700

C -1.84218400 4.86128400 1.26829400

H 0.02692400 4.91983100 0.18760400

C -2.71515900 4.08910400 2.04330600

H -3.06593900 2.15884300 2.95558900

H -2.09511700 5.89506700 1.02201200

H -3.65866800 4.51169900 2.39461600

C 1.89189900 3.19471400 -0.83448300

C 3.26465600 3.46445100 -0.97763400

C 1.01123700 3.53039700 -1.87987200

C 3.74490000 4.06311800 -2.14525500

H 3.96210600 3.18957200 -0.18676400

C 1.49841000 4.13132900 -3.04373900

H -0.05155500 3.29958800 -1.79656100

C 2.86543400 4.39644000 -3.18134600

H 4.81477600 4.25894800 -2.24404000

H 0.80363700 4.38099300 -3.84872100

H 3.24463300 4.86097100 -4.09470800

C 2.35102800 2.64759200 2.02532600

C 2.82085600 3.95479900 2.23032300

C 2.64117100 1.64841600 2.96500400

C 3.60180400 4.24612100 3.35295800

H 2.58870700 4.74163700 1.50911600

C 3.41063900 1.94544700 4.09242500

H 2.27089300 0.63617100 2.80263700

C 3.89960400 3.24278000 4.28335700

H 3.97772900 5.26097200 3.50152600

H 3.63536300 1.15499500 4.81162500

H 4.51170900 3.47460300 5.15804500

**TS^cis-5+8^**

C 2.81779400 2.07183200 0.33500300

C 2.95127100 3.23365000 -0.06568300

C -8.02745000 -0.52070800 -0.16376100

C -7.83753400 1.84677200 0.36721400

C -9.41131100 -0.38062600 -0.27120700

H -7.55131600 -1.48914900 -0.32367700

C -9.22320600 1.97506700 0.25939500

H -7.21428900 2.70648700 0.61806000

C -10.01598000 0.86530700 -0.06077200

H -10.02348900 -1.25052300 -0.52247900

H -9.68926000 2.94933300 0.42553100

H -11.09994800 0.97061500 -0.14685300

Pd -2.65673200 0.04607000 0.25582000

C 3.19253200 4.52921600 -0.60400100

C 4.18135100 5.36899300 -0.04147800

C 2.49343600 4.98838400 -1.74274800

C 4.46125600 6.61544300 -0.60256100

H 4.73025900 5.01576300 0.83354900

C 2.77753000 6.23672700 -2.29768900

H 1.72565500 4.34923200 -2.18054400

C 3.76285000 7.05739400 -1.73357400

H 5.23346500 7.24717700 -0.15587500

H 2.22862300 6.57079100 -3.18223500

H 3.98640800 8.03238300 -2.17254700

P -3.29145700 -1.91328700 -0.89435300

P -2.41253500 2.23319900 1.10621700

P 2.40824200 -1.99104900 1.74100800

C 4.00308900 0.63031700 2.26836300

C 5.37027000 0.70846600 1.95490500

C 3.62047600 0.80882200 3.60926000

C 6.32809500 0.94899300 2.94952300

H 5.70561000 0.55942600 0.92598600

C 4.57654300 1.05143700 4.60340300

H 2.56991200 0.73490100 3.89589000

C 5.93530700 1.12544300 4.27952600

H 7.38637300 0.99627200 2.67736700

H 4.25151900 1.17214300 5.64035400

H 6.68037500 1.31402600 5.05605700

I -0.07746200 -0.08915100 -0.51912000

C -7.21964000 0.59392000 0.15704800

C -5.80561600 0.44824700 0.25751900

C -4.58653900 0.29196500 0.36474300

C 3.40828700 -2.32659200 3.25848500

C 4.81081700 -2.30802200 3.14466400

C 2.82644700 -2.50648100 4.52135300

C 5.61368800 -2.45474100 4.27449200

H 5.27406800 -2.15580600 2.16839800

C 3.63618500 -2.65421900 5.65425800

H 1.74034300 -2.51919500 4.62590200

C 5.02732200 -2.62322200 5.53517900

H 6.70002600 -2.41616900 4.17310100

H 3.17226000 -2.78725200 6.63442000

H 5.65691700 -2.72414900 6.42219400

C 2.81439400 -3.45603300 0.68675800

C 3.43455000 -4.60595600 1.20993100

C 2.59287500 -3.36745800 -0.69621100

C 3.85156500 -5.63302200 0.35821100

H 3.61193600 -4.68761100 2.28388900

C 3.02789800 -4.38879500 -1.54455100

H 2.09763800 -2.48185600 -1.10301400

C 3.66303600 -5.51856500 -1.02370900

H 4.34229600 -6.51563100 0.77555900

H 2.90383600 -4.28794800 -2.62445500

H 4.02206700 -6.30218800 -1.69451100

C 0.68183900 -2.26465700 2.33358200

C -0.01022100 -1.16787200 2.87980200

C 0.06198700 -3.52059900 2.28015800

C -1.30340200 -1.33323000 3.38020200

H 0.45599400 -0.17913200 2.88356200

C -1.23391200 -3.68161200 2.78258500

H 0.58494700 -4.37010400 1.83590700

C -1.91561800 -2.59295600 3.33580500

H -1.83218800 -0.47259800 3.79579200

H -1.71106000 -4.66320400 2.73841600

H -2.92424400 -2.72732800 3.73372800

C -4.77720200 -2.76891600 -0.20997000

C -5.02015600 -2.71246200 1.17159000

C -5.62632000 -3.52400500 -1.03284900

C -6.09887900 -3.40708900 1.72388000

H -4.37154400 -2.10431000 1.80335000

C -6.71060300 -4.21214600 -0.47704400

H -5.45167500 -3.56723800 -2.10955700

C -6.94800400 -4.15559600 0.90051700

H -6.28574500 -3.35160300 2.79848000

H -7.37346200 -4.79005100 -1.12496400

H -7.79911000 -4.68731400 1.33158300

C -2.11738700 -3.31502900 -1.09535600

C -2.26133000 -4.49296500 -0.34627400

C -1.06195600 -3.20697000 -2.01894800

C -1.36247100 -5.55089300 -0.51962700

H -3.07790900 -4.58940800 0.37034400

C -0.19596500 -4.28137800 -2.21356900

H -0.92682600 -2.29275700 -2.59737700

C -0.33210400 -5.45099000 -1.45767600

H -1.47753200 -6.45873200 0.07704600

H 0.59834200 -4.19583300 -2.95536500

H 0.37041500 -6.27525900 -1.59568900

C -3.74205500 -1.47672900 -2.63676300

C -3.58878400 -2.39373600 -3.69279400

C -4.25708800 -0.19823000 -2.91194600

C -3.92796200 -2.02908500 -4.99954300

H -3.19377400 -3.39245900 -3.49991400

C -4.59431900 0.16093700 -4.21948400

H -4.41355700 0.50459600 -2.09229400

C -4.42526500 -0.74953400 -5.26796200

H -3.79968200 -2.75071600 -5.80953200

H -4.99298900 1.15881500 -4.41514100

H -4.68500900 -0.46552200 -6.29039800

C -1.18997800 2.56792100 2.44156000

C 0.17143100 2.63787500 2.10115200

C -1.58330800 2.77847200 3.77629100

C 1.12391900 2.94109300 3.07699100

H 0.50610000 2.48055900 1.07516900

C -0.62080500 3.05629600 4.75197800

H -2.63734600 2.75432200 4.05589200

C 0.73201300 3.14367900 4.40437000

H 2.16992500 3.00832800 2.77538600

H -0.93703700 3.22644200 5.78414100

H 1.48146800 3.37512500 5.16502800

C -3.95711600 2.98799500 1.77884800

C -4.38018300 4.26984300 1.40097900

C -4.70896100 2.26298700 2.72140200

C -5.53283100 4.82527500 1.97062000

H -3.81644400 4.83606600 0.65774600

C -5.84730600 2.82658200 3.29974100

H -4.41127900 1.24558200 2.98217600

C -6.26204100 4.11102200 2.92529700

H -5.85775700 5.82210300 1.66432500

H -6.42487300 2.25176300 4.02648200

H -7.16148100 4.54650500 3.36658600

C -1.92470300 3.33744400 -0.28383300

C -1.23081000 4.54227100 -0.07922900

C -2.29938400 2.96624500 -1.58559200

C -0.91801000 5.36216200 -1.16757600

H -0.91861900 4.83241200 0.92538600

C -2.00088900 3.79772800 -2.66708700

H -2.81026300 2.01542000 -1.74803700

C -1.30657700 4.99469400 -2.46069600

H -0.35273600 6.28134800 -1.00493400

H -2.30271600 3.50014700 -3.67393000

H -1.05596200 5.63628100 -3.30885200

Pd 2.68079500 0.15776100 0.81121000

P 3.60032300 -0.00215100 -1.91377900

C 4.75028300 -1.39787900 -2.30957000

C 5.02694600 -1.86040100 -3.60736200

C 5.40082900 -1.99668800 -1.21773900

C 5.91159700 -2.92552400 -3.80081900

H 4.54801900 -1.38688200 -4.46745600

C 6.29954000 -3.04877200 -1.41240500

H 5.17393600 -1.64180600 -0.20884400

C 6.54684500 -3.52280100 -2.70455200

H 6.11340000 -3.28568500 -4.81268400

H 6.78705900 -3.51326900 -0.55268900

H 7.23577100 -4.35634200 -2.86034500

C 4.60605500 1.43752000 -2.51520200

C 4.59266000 1.86850600 -3.85328500

C 5.42514400 2.10801600 -1.59316400

C 5.36728000 2.96267700 -4.25104000

H 3.96116900 1.36099000 -4.58565200

C 6.20670700 3.19399400 -1.99529100

H 5.41702900 1.80700700 -0.54660000

C 6.17239200 3.63098600 -3.32276900

H 5.33510400 3.29695500 -5.29106200

H 6.82059600 3.71679100 -1.25904500

H 6.76335200 4.49644000 -3.63079400

C 2.25528100 -0.08098100 -3.17188900

C 1.79197800 -1.28025400 -3.74122900

C 1.50802000 1.09675700 -3.38441700

C 0.61887200 -1.29975600 -4.50476400

H 2.34742100 -2.20684800 -3.58552200

C 0.33892500 1.07204000 -4.14495900

H 1.83172600 2.02791900 -2.91466400

C -0.11862400 -0.12790600 -4.70156300

H 0.27402600 -2.24141600 -4.94000600

H -0.23289600 1.99257900 -4.27516000

H -1.04894600 -0.15361500 -5.27334500

**4-PPh3**

C 1.02398900 -2.05710200 0.20816500

C 1.57366300 -2.46084900 1.43712900

C 1.66217100 -2.41300300 -0.99162000

C 2.73084300 -3.24875400 1.45968300

H 1.10926900 -2.15644400 2.37830800

C 2.82452800 -3.19676600 -0.95903300

H 1.26555200 -2.07604400 -1.95231000

C 3.36188700 -3.61209800 0.26417100

H 3.14690700 -3.56712100 2.41891200

H 3.31286100 -3.47450100 -1.89669100

H 4.27278600 -4.21467800 0.28637300

Pd -0.77281000 -1.20439300 0.20252000

P 0.38826100 0.68297100 -0.01817400

I -3.38927700 -1.28291800 0.13800200

C 1.85043700 1.00084500 1.05647500

C 1.74967900 1.89659500 2.13434600

C 3.06101900 0.31413500 0.83881300

C 2.84396800 2.10416200 2.98024500

H 0.81797600 2.43568500 2.31370700

C 4.15159100 0.53543700 1.68143900

H 3.15071100 -0.39597500 0.01498600

C 4.04599400 1.42729800 2.75516900

H 2.75327800 2.80151500 3.81573900

H 5.08448600 -0.00262000 1.50136300

H 4.89972000 1.59221200 3.41616800

C -0.68974600 2.14915200 0.28013500

C -0.41835100 3.36776700 -0.36589600

C -1.74748100 2.06569700 1.19929800

C -1.19984400 4.49203500 -0.08817700

H 0.39554700 3.43749700 -1.09003700

C -2.52141700 3.19646500 1.47581000

H -1.97911800 1.11217000 1.67828500

C -2.24966600 4.40870900 0.83372800

H -0.99041300 5.43448800 -0.59878300

H -3.34757400 3.12142500 2.18554800

H -2.86205700 5.28832700 1.04410200

C 0.97755400 0.89588100 -1.74744800

C 0.15226200 0.40939300 -2.77770800

C 2.17473700 1.55680200 -2.06759300

C 0.52552100 0.58094400 -4.11235100

H -0.77871700 -0.10615200 -2.52419000

C 2.54460600 1.71991000 -3.40690800

H 2.81857100 1.94214600 -1.27489200

C 1.72368100 1.23255300 -4.42865500

H -0.11951000 0.19969400 -4.90655700

H 3.47906100 2.23038900 -3.64994000

H 2.01795700 1.35888800 -5.47282200

**4-I**

C 0.01202300 -1.66804100 0.11123200

C 0.17359800 -2.36044500 -1.09326500

C -0.17615800 -2.34255600 1.32210600

C 0.14074500 -3.76181600 -1.07787100

H 0.31774600 -1.83226800 -2.03663900

C -0.20765800 -3.74499800 1.31726400

H -0.29344400 -1.80250400 2.26218200

C -0.04885800 -4.45401400 0.12284600

H 0.26184400 -4.30712900 -2.01632800

H -0.35397800 -4.27702300 2.26009000

H -0.07421000 -5.54541500 0.12686200

Pd 0.01601300 0.31768900 0.09195200

P 2.34540500 0.32622000 0.05173400

P -2.33922700 0.28605700 0.00479000

C 3.06279000 -0.10479700 -1.57400100

C 4.35234300 -0.64950300 -1.69085600

C 2.30317800 0.15367700 -2.72923300

C 4.87423000 -0.93002500 -2.95737800

H 4.94278200 -0.85706000 -0.79626500

C 2.83281600 -0.12399600 -3.99113100

H 1.29618800 0.57160600 -2.63459200

C 4.11806400 -0.66773700 -4.10497900

H 5.87571900 -1.35520000 -3.04665000

H 2.24103300 0.07836900 -4.88587000

H 4.52961000 -0.89076400 -5.09127900

C 3.27018400 -0.64518000 1.29490500

C 4.03708200 -0.02498600 2.29466400

C 3.18683400 -2.05026400 1.23987000

C 4.71266700 -0.80756800 3.23669800

H 4.11406000 1.06316300 2.33522100

C 3.86925900 -2.82112500 2.18247900

H 2.59792400 -2.53850700 0.46082900

C 4.62881800 -2.20238500 3.18300600

H 5.31082100 -0.32286900 4.01069500

H 3.80411300 -3.90979100 2.13520400

H 5.15890800 -2.80935500 3.91959700

C 2.72693400 2.09855800 0.33633500

C 3.58927900 2.82727700 -0.49932600

C 2.06603100 2.74957400 1.39933400

C 3.79523000 4.18982700 -0.26405800

H 4.09625500 2.33032000 -1.32860900

C 2.27969800 4.11120300 1.62888700

H 1.40018800 2.18256200 2.05833100

C 3.14338800 4.83151500 0.79547200

H 4.47014600 4.75285500 -0.91158100

H 1.77262300 4.60896600 2.45768300

H 3.30880500 5.89602700 0.97220200

C -2.92988100 -1.06701900 -1.07634000

C -3.25913000 -2.31818000 -0.52942000

C -2.85909000 -0.91104200 -2.47247300

C -3.53067100 -3.39744700 -1.37445900

H -3.28647900 -2.45477500 0.55278900

C -3.13770500 -1.99377300 -3.30999500

H -2.58838700 0.05562400 -2.90405200

C -3.47221800 -3.23788600 -2.76251300

H -3.78261000 -4.36797100 -0.94321200

H -3.09251400 -1.86528600 -4.39333000

H -3.68610200 -4.08327200 -3.41941900

C -3.16334200 0.06457700 1.62702400

C -2.40362000 0.19624600 2.80040100

C -4.54475400 -0.18439100 1.71419300

C -3.01659000 0.07597100 4.05144600

H -1.33023700 0.39549600 2.72941700

C -5.15158600 -0.30709400 2.96556900

H -5.14330700 -0.28833700 0.80663000

C -4.38917300 -0.17755200 4.13353500

H -2.42149400 0.17839300 4.96089000

H -6.22357600 -0.50293800 3.03055000

H -4.86850100 -0.27423500 5.10962600

C -3.01201800 1.84814700 -0.68655200

C -4.25690000 1.90587900 -1.33704100

C -2.26330400 3.02546400 -0.51292000

C -4.74103800 3.12978600 -1.80572300

H -4.84110500 0.99534300 -1.48569200

C -2.75276200 4.24753200 -0.98189600

H -1.29580900 2.99397500 -0.00004600

C -3.99176300 4.29919400 -1.62904200

H -5.70777900 3.17058900 -2.31129700

H -2.16582700 5.15745700 -0.84387300

H -4.37450500 5.25248100 -1.99847100

**15**

C -0.17027000 -1.46619200 -2.09718300

C -0.34759400 -2.21420400 -1.12714600

C 0.19635600 1.21677400 1.28477600

C 0.50095900 2.57690500 1.12040000

C 0.02414100 0.71580600 2.58808600

C 0.62941700 3.41816900 2.23312200

H 0.64272000 2.99855700 0.12527000

C 0.14202600 1.56152400 3.69886300

H -0.18579000 -0.34208400 2.75753400

C 0.44784400 2.91473900 3.52569100

H 0.87038000 4.47273200 2.08094100

H 0.00203400 1.15225700 4.70217000

H 0.54379100 3.57261900 4.39202500

Pd 0.01359400 -0.11410500 -0.24794200

C -0.62102900 -3.17140400 -0.09352900

C 0.08063900 -3.13289300 1.13261400

C -1.62342500 -4.14338400 -0.29290500

C -0.23336600 -4.04130300 2.14351900

H 0.85834200 -2.38183700 1.29124400

C -1.92753700 -5.04608500 0.72651600

H -2.15311900 -4.18008900 -1.24561400

C -1.24057200 -4.99406300 1.94573400

H 0.31203100 -4.00407300 3.08810100

H -2.70604700 -5.79446500 0.56892900

H -1.48593700 -5.70178400 2.73973100

P -2.26665400 0.54657600 -0.29230400

P 2.40027000 -0.12213700 -0.27657700

H 0.00366000 -1.06503900 -3.08041600

C 3.15350700 -1.29105200 -1.48570100

C 4.01735600 -0.83360900 -2.49573700

C 2.84955700 -2.66402300 -1.41264300

C 4.57014400 -1.73697000 -3.40936500

H 4.26317300 0.22672100 -2.56961900

C 3.41348800 -3.56274100 -2.32031800

H 2.17527300 -3.03975100 -0.64154300

C 4.27259000 -3.10019600 -3.32351900

H 5.24126900 -1.36996300 -4.18839600

H 3.17429800 -4.62539200 -2.24668200

H 4.70797000 -3.80201100 -4.03750600

C 2.93351100 1.52897300 -0.87496800

C 4.10181300 2.15992500 -0.42144800

C 2.14360300 2.15192500 -1.85791000

C 4.46251500 3.40960500 -0.93672000

H 4.72724200 1.68001100 0.33319100

C 2.51164200 3.39513800 -2.37491700

H 1.23045300 1.65971700 -2.20565900

C 3.67049000 4.02838700 -1.90938700

H 5.36907900 3.89962800 -0.57633300

H 1.89144700 3.87344100 -3.13577300

H 3.95630900 5.00458600 -2.30595800

C 3.27864900 -0.41384000 1.31338500

C 3.21061300 0.56336800 2.32641400

C 3.97032800 -1.61367600 1.55714400

C 3.82259900 0.33405200 3.56031400

H 2.69140300 1.50634400 2.15113800

C 4.57171100 -1.83717900 2.79971800

H 4.06045900 -2.37021500 0.77687000

C 4.49705100 -0.86729300 3.80400300

H 3.76476100 1.10181000 4.33417300

H 5.11021400 -2.77058900 2.97530000

H 4.97109700 -1.04343500 4.77168500

C -3.19720900 -0.02139900 -1.78216900

C -3.55783900 -1.38177100 -1.81626000

C -3.54208100 0.80826800 -2.86039600

C -4.23708800 -1.90513500 -2.91667800

H -3.32692300 -2.02406200 -0.96376600

C -4.22236700 0.27683500 -3.96252600

H -3.30922900 1.87310600 -2.83908900

C -4.56476700 -1.07764400 -3.99768600

H -4.52020500 -2.95988400 -2.92730400

H -4.49513200 0.93272400 -4.79172300

H -5.09802400 -1.48579500 -4.85838400

C -2.32786500 2.37776200 -0.29549700

C -2.85168500 3.10948300 0.78035400

C -1.66985500 3.05707900 -1.33922600

C -2.74424700 4.50369600 0.79436500

H -3.31702400 2.59491200 1.62171900

C -1.57638200 4.44980900 -1.32559600

H -1.20553900 2.49346300 -2.15262300

C -2.11535300 5.17625200 -0.25728600

H -3.14811200 5.06437900 1.63937300

H -1.06856100 4.96698400 -2.14222900

H -2.03479300 6.26482500 -0.24066900

C -3.34141800 -0.06973700 1.05745200

C -4.67950900 0.35749600 1.15388400

C -2.86648000 -1.06827500 1.91940200

C -5.51459900 -0.18902300 2.12977800

H -5.06823900 1.10688000 0.46064700

C -3.71132800 -1.62294200 2.88577300

H -1.84258000 -1.42832300 1.81923200

C -5.03145300 -1.17787600 2.99716900

H -6.54968000 0.14931400 2.20716300

H -3.33412300 -2.40548200 3.54691400

H -5.69129000 -1.60686000 3.75393300

**TS^15^**

C 0.03895900 -1.98614100 -0.05824300

C -0.03174000 -2.19640800 -1.28748000

C -0.38456100 2.22991700 -0.32453700

C -0.47140900 3.15176200 0.72963200

C -0.51937200 2.69804200 -1.64581000

C -0.67730000 4.51379100 0.47013000

H -0.37984700 2.81940300 1.76536300

C -0.73977000 4.05722500 -1.90307600

H -0.44551900 2.00450500 -2.48839400

C -0.81487400 4.97014400 -0.84493600

H -0.73890300 5.21732700 1.30390800

H -0.84114700 4.40171700 -2.93512600

H -0.98064500 6.03100000 -1.04449200

Pd -0.13343100 0.23054300 -0.12388100

C -0.05820400 -2.37582800 -2.70380300

C -0.57634600 -3.55277000 -3.28316800

C 0.41413700 -1.34249400 -3.54678400

C -0.61095700 -3.69244900 -4.67103300

H -0.95440300 -4.34537600 -2.63607000

C 0.36250300 -1.48822400 -4.93328800

H 0.79737900 -0.42101700 -3.09985900

C -0.14680700 -2.66332600 -5.50003700

H -1.01138500 -4.60822100 -5.11030700

H 0.72228800 -0.68044200 -5.57365100

H -0.18389900 -2.77617300 -6.58514200

P 2.16999600 0.72293100 -0.07143600

P -2.42423100 0.00842700 0.36693500

H 0.21949700 -2.49763300 1.27964500

C 0.94081900 -4.42982200 2.05688200

C 1.58817200 -2.28083700 3.00766400

H -0.38430600 -3.02905800 2.90758800

C 2.46459200 -4.31950600 2.12206000

H 0.55408300 -4.73105600 1.07251500

H 0.54752500 -5.11373500 2.82428100

C 2.67840000 -3.32354000 3.26983100

H 1.21357300 -1.77034300 3.90422000

H 1.93243700 -1.52205300 2.28894800

H 2.94571600 -5.29325500 2.28828800

H 2.85109300 -3.89588900 1.18132900

H 2.51835000 -3.81587500 4.24351300

H 3.68104700 -2.87466500 3.27001100

N 0.47373900 -3.03615100 2.34591400

C -2.46968300 -1.12341700 1.82388700

C -1.93857400 -0.64919600 3.04000900

C -2.93303500 -2.44617600 1.74822400

C -1.91728800 -1.46837600 4.17267000

H -1.55893400 0.37383700 3.10350600

C -2.90443900 -3.26825900 2.88205000

H -3.31606500 -2.83612600 0.80426500

C -2.40620000 -2.78191300 4.09841000

H -1.52570100 -1.08109200 5.11583300

H -3.27986800 -4.29158000 2.81537900

H -2.40340800 -3.41925900 4.98517000

C -3.46252800 1.44559500 0.87590900

C -3.59301700 2.49848000 -0.04802600

C -4.16649100 1.50233400 2.09001900

C -4.39637400 3.59899700 0.24975800

H -3.05743500 2.46359200 -0.99772200

C -4.96973300 2.61045500 2.38270000

H -4.10526900 0.68392300 2.80856800

C -5.08175600 3.66148000 1.46859700

H -4.47899200 4.41333500 -0.47226700

H -5.51351900 2.64492700 3.32902500

H -5.70668900 4.52573700 1.70248000

C -3.45371100 -0.74603900 -0.94912100

C -4.77407700 -1.16493100 -0.69957800

C -2.93478200 -0.81804300 -2.24922500

C -5.54265400 -1.69109900 -1.73947100

H -5.20132900 -1.07083200 0.30177100

C -3.70875800 -1.34277100 -3.28886000

H -1.92501000 -0.45723300 -2.44698300

C -5.00892500 -1.78666900 -3.03219900

H -6.56520300 -2.02031300 -1.54393900

H -3.28799500 -1.40537600 -4.29418000

H -5.61573700 -2.19883500 -3.84106100

C 2.83955200 1.84294000 -1.37269000

C 2.55706500 3.22055300 -1.30057800

C 3.56276600 1.34351900 -2.46927700

C 2.99969300 4.07910100 -2.30903600

H 1.99122100 3.62409900 -0.45900400

C 3.99380200 2.20926500 -3.47925900

H 3.80827800 0.28278300 -2.53549100

C 3.71384500 3.57712600 -3.40212500

H 2.77472200 5.14502200 -2.23910000

H 4.55975900 1.81051800 -4.32362000

H 4.05564700 4.25128500 -4.19010600

C 2.52899800 1.57388000 1.51976100

C 1.69249900 1.31136400 2.61772100

C 3.63585000 2.42417700 1.68343400

C 1.96002100 1.88210900 3.86436000

H 0.81960000 0.66709300 2.47663600

C 3.89557600 3.00137800 2.93041500

H 4.29121400 2.63729100 0.83653600

C 3.06206700 2.73016600 4.02138900

H 1.30114200 1.67624000 4.71088000

H 4.75375400 3.66575600 3.04941400

H 3.26811900 3.18548600 4.99211400

C 3.33619600 -0.71294400 -0.06081300

C 3.22519500 -1.69323200 -1.06359100

C 4.34410400 -0.84248500 0.91097800

C 4.12187400 -2.76388500 -1.11188900

H 2.44057500 -1.62050100 -1.81539200

C 5.23162500 -1.92331700 0.86682000

H 4.44335700 -0.09870800 1.70310000

C 5.12786500 -2.88242700 -0.14617600

H 4.02752400 -3.50856300 -1.90480600

H 6.01435300 -2.00743400 1.62386300

H 5.82725100 -3.72017900 -0.18198100

**base**

C -1.15168100 -0.45347900 0.19844200

C 1.15138500 -0.45370000 0.19914700

H 0.00022200 -1.31176400 -1.23979000

C -0.77686900 1.02846400 -0.07055600

H -1.30814700 -0.61364800 1.28014300

H -2.06634000 -0.77946300 -0.31895500

C 0.77727300 1.02801400 -0.07143300

H 2.06653000 -0.78037300 -0.31695900

H 1.30652300 -0.61310800 1.28116100

H -1.16655800 1.35373600 -1.04890200

H -1.19870400 1.70646700 0.68818900

H 1.16595400 1.35135400 -1.05083100

H 1.20050500 1.70701100 0.68564100

N -0.00009100 -1.27371500 -0.21332800

**base-H^+^**

C 0.32865200 1.23608200 0.14696300

C 0.33511500 -1.23473400 -0.14556500

C -1.05031900 0.72033400 -0.25835100

H 0.73578400 2.04660000 -0.46900500

H 0.36789300 1.52577100 1.20529100

C -1.04698700 -0.72540700 0.25757700

H 0.74598700 -2.04110900 0.47368100

H 0.37710100 -1.52800600 -1.20274100

H -1.84523600 1.33593000 0.18348700

H -1.16852800 0.74627000 -1.35354200

H -1.16605400 -0.75149100 1.35270800

H -1.83842200 -1.34507900 -0.18465000

H 1.85733100 0.09568100 -0.81652200

H 1.86099700 -0.08546600 0.81272500

N 1.23919800 0.00332200 -0.00074000

**2**

C -3.24293000 -0.00019000 -0.00040400

C -2.02539800 0.00050700 0.00017200

C -0.59464200 0.00145900 0.00076500

C 0.11905600 -1.21559600 0.00009600

C 0.12083900 1.21680300 0.00003100

C 1.51444700 -1.21185000 -0.00007000

H -0.43397800 -2.15610800 0.00009800

C 1.51646500 1.21042500 -0.00010000

H -0.42997100 2.15864100 -0.00018700

C 2.21746100 -0.00114100 -0.00013200

H 2.05703300 -2.15963100 -0.00065300

H 2.06045200 2.15741000 -0.00005400

H 3.30955400 -0.00199800 -0.00056000

H -4.31488900 -0.00081400 -0.00079700

**16**

Pd 1.63437800 0.00639300 0.46047400

C -0.19324000 -0.65525600 1.09486600

C -1.19909700 -1.35813800 1.38122200

C -3.56206600 1.65208500 -2.48104800

C -1.28522400 2.30282400 -2.01843800

C -3.70042500 2.76203700 -3.32285100

H -4.41295900 0.98026000 -2.35949200

C -1.41941800 3.41130100 -2.86378100

H -0.33995700 2.14213600 -1.49632100

C -2.63334500 3.64470600 -3.51749900

H -4.65518700 2.93389000 -3.82455700

H -0.56961100 4.08432500 -3.00298900

H -2.74805700 4.51014200 -4.17370900

Pd -1.84942500 0.03107300 -0.41821300

C -2.16009000 -2.25192300 1.97651000

C -2.19516800 -2.42705000 3.37648700

C -3.06862900 -2.98071000 1.18042700

C -3.11036200 -3.30823400 3.95520600

H -1.48591600 -1.88699700 4.00297400

C -3.96489100 -3.87385900 1.76339700

H -3.06136900 -2.83835400 0.10076600

C -3.99271900 -4.04018800 3.15335200

H -3.12395900 -3.43091500 5.04034400

H -4.65595500 -4.43108600 1.12895200

H -4.70371000 -4.73213700 3.60933500

P 1.72327300 2.42029100 0.45772800

P -3.49272900 1.02782300 1.04970200

P -1.48544200 -1.37756900 -2.32717800

P 2.66656200 -2.00493000 1.12856900

C 6.81421100 -0.02626600 -1.30389100

C 5.89804200 1.96802300 -2.36136200

C 8.06786200 0.27614800 -1.83599500

H 6.67018900 -0.90544600 -0.67395300

C 7.15701300 2.26030300 -2.88854300

H 5.04790700 2.62532600 -2.55261500

C 8.24446100 1.41658600 -2.63045800

H 8.91548300 -0.38069600 -1.62826900

H 7.29142100 3.15318600 -3.50322500

H 9.22783500 1.64842500 -3.04476000

C -2.35352600 1.41591000 -1.81234900

C 5.71147700 0.81702100 -1.56359200

C 4.43711400 0.53294200 -0.99123300

C 3.35436100 0.33472800 -0.43655200

C 4.44375800 -1.76428100 1.57018500

C 5.38733300 -2.76932500 1.30746300

C 4.84639500 -0.58732800 2.22357700

C 6.71910400 -2.60135100 1.70235100

H 5.09454700 -3.67875100 0.78167200

C 6.17458400 -0.43265900 2.62659400

H 4.12867700 0.21634100 2.40065400

C 7.11379500 -1.43685900 2.36685200

H 7.44744000 -3.38535500 1.48477000

H 6.47484800 0.48524400 3.13295300

H 8.15355700 -1.30629600 2.67363700

C 1.98197000 -2.77629700 2.65743400

C 0.87818200 -3.64191500 2.58101300

C 2.55184100 -2.50231800 3.91308500

C 0.37834700 -4.25561600 3.73132300

H 0.40665000 -3.84407600 1.62107000

C 2.03193100 -3.09941700 5.06550700

H 3.41391700 -1.83881100 3.99386800

C 0.95291200 -3.98511500 4.97751900

H -0.47320200 -4.93291800 3.64941300

H 2.48721700 -2.88303500 6.03421800

H 0.56077500 -4.46359800 5.87740100

C 2.68267600 -3.34410100 -0.12550500

C 2.80124700 -4.70228900 0.22286700

C 2.64017300 -2.97513400 -1.47806200

C 2.85690900 -5.67327500 -0.78143700

H 2.84608900 -5.00119200 1.27205300

C 2.68320200 -3.95042000 -2.47648800

H 2.57905100 -1.92105700 -1.74355300

C 2.78714500 -5.30074500 -2.12911700

H 2.94668200 -6.72669100 -0.50775600

H 2.62739900 -3.64610500 -3.52302500

H 2.81322200 -6.06617800 -2.90730000

C 2.37570200 3.34981400 -0.99675400

C 2.13045100 2.88818800 -2.29597200

C 2.55443500 3.62116900 -3.40768900

H 1.60453700 1.95041200 -2.44497500

H 2.35167600 3.23907900 -4.41013300

C 3.02693200 2.69146700 1.76145500

C 2.69328900 2.67847400 3.12630300

C 4.37836100 2.83010000 1.39368100

C 3.68590200 2.82251200 4.10112000

H 1.65712800 2.56724800 3.44348700

C 5.36258100 2.99518600 2.37063700

H 4.66944300 2.80278500 0.34481100

C 5.02233100 2.99148600 3.72768500

H 3.40630300 2.81329600 5.15659400

H 6.40299600 3.11482500 2.06158700

H 5.79430900 3.11745700 4.48979300

C 0.35835000 3.51872200 1.05109400

C 0.11730800 4.79362200 0.50653700

C -0.37286400 3.10963100 2.17686300

C -0.78382300 5.66257500 1.12897600

H 0.63591200 5.11663000 -0.39574600

C -1.24621200 3.99411700 2.81665200

H -0.24858200 2.09487500 2.55720500

C -1.44466300 5.27723100 2.30007100

H -0.96060000 6.65023800 0.69775900

H -1.78004900 3.67196300 3.71082000

H -2.13341500 5.96494600 2.79404500

C -0.03906300 -1.13338900 -3.44112100

C 0.04072800 -1.80032800 -4.67890400

C 1.04297400 -0.36421600 -3.00663800

C 1.18489900 -1.67446500 -5.46775000

H -0.78973600 -2.41839200 -5.02538600

C 2.19939000 -0.25155000 -3.78861400

H 0.99578900 0.10939100 -2.01978300

C 2.26697900 -0.90096800 -5.02312800

H 1.23725000 -2.18949800 -6.42932800

H 3.04781000 0.32262200 -3.41199900

H 3.16642700 -0.81517200 -5.63635700

C -1.38052300 -3.20301200 -2.03371800

C -1.95292100 -4.12911400 -2.92635900

C -0.64365900 -3.67415600 -0.93679500

C -1.80678000 -5.50114000 -2.70639900

H -2.51989500 -3.78456300 -3.79214300

C -0.51141500 -5.04723000 -0.71626100

H -0.19084100 -2.95687300 -0.25203300

C -1.09296800 -5.96327400 -1.59621600

H -2.25781300 -6.20915100 -3.40486900

H 0.05302400 -5.40541700 0.14511000

H -0.98800100 -7.03555500 -1.41900800

C -2.93930200 -1.24953800 -3.45201600

C -4.14920600 -1.86056300 -3.07591200

C -2.89890600 -0.46703600 -4.61661900

C -5.29357800 -1.70259100 -3.86188600

H -4.19605200 -2.46957000 -2.17041300

C -4.04529900 -0.31515800 -5.40214900

H -1.97809900 0.04440000 -4.90064200

C -5.24390300 -0.92954600 -5.02800800

H -6.22513000 -2.19020200 -3.56670400

H -4.00105700 0.29897500 -6.30356900

H -6.13819400 -0.80796700 -5.64245300

C -4.84624400 -0.22849500 1.13044500

C -5.35834200 -0.73061800 -0.07891400

C -5.37729200 -0.69000100 2.34454900

C -6.40169800 -1.65700300 -0.07573600

H -4.93953400 -0.38877200 -1.02601600

C -6.41670200 -1.62619800 2.34276200

H -4.97679900 -0.33242000 3.29316800

C -6.93640700 -2.10628300 1.13769500

H -6.79882200 -2.02713100 -1.02330700

H -6.81613600 -1.98442800 3.29346700

H -7.75111900 -2.83329400 1.14247200

C -3.27744100 1.44682100 2.83825700

C -2.24585700 0.88065400 3.60118300

C -4.20989300 2.29537000 3.46439300

C -2.14525200 1.15597000 4.96885500

H -1.50686300 0.24653200 3.11410700

C -4.10927100 2.56400300 4.83228000

H -5.01241500 2.75041900 2.88140700

C -3.07731700 1.99480400 5.58884200

H -1.33179300 0.71391900 5.54868000

H -4.83850600 3.22352500 5.30733700

H -2.99883100 2.20921700 6.65669500

C -4.32309200 2.53651600 0.40276000

C -3.50395500 3.64012700 0.11296000

C -5.70606900 2.61589600 0.17934500

C -4.06256500 4.80998400 -0.39931600

H -2.42621900 3.57077600 0.26062500

C -6.25991900 3.79194800 -0.33968100

H -6.35026800 1.76349800 0.40207400

C -5.44113600 4.88678500 -0.63205500

H -3.41381100 5.65575900 -0.63367200

H -7.33620500 3.84896800 -0.51599900

H -5.87644200 5.79913700 -1.04528500

C 3.05891400 4.56907300 -0.82415000

C 3.24285300 4.82560000 -3.23037900

C 3.49449100 5.29582400 -1.93615700

H 3.25217500 4.95366800 0.17891300

H 4.02988100 6.23576400 -1.78771600

H 3.58371800 5.39679700 -4.09634900

**17**

Pd 1.51496100 -0.45052400 0.19206500

C -0.60446600 -0.26262500 1.15546900

C 0.15442300 -0.89897500 1.94167900

C -3.30308900 0.87325900 -1.72484700

C -4.47880400 1.63718500 -1.77442700

C -2.90252900 0.20973000 -2.90171100

C -5.21917400 1.74672200 -2.96035300

H -4.83496400 2.17182500 -0.89357500

C -3.62962400 0.32820100 -4.09064800

H -2.03157900 -0.44952000 -2.89223500

C -4.79637800 1.10068500 -4.12355400

H -6.13040900 2.34989900 -2.96571600

H -3.29256200 -0.20404900 -4.98370700

H -5.37583700 1.18537300 -5.04557200

Pd -2.03797000 0.45630000 -0.14737000

C 0.62741000 -1.68342800 3.05512500

C 0.07690900 -1.49694600 4.34023300

C 1.62502400 -2.66461800 2.88194900

C 0.50878500 -2.28033200 5.41053300

H -0.67399000 -0.72070700 4.49105800

C 2.05269300 -3.44416600 3.95656700

H 2.05818000 -2.81445800 1.89199000

C 1.49556500 -3.25699900 5.22609100

H 0.08079100 -2.11649500 6.40185700

H 2.82555300 -4.19904900 3.79733400

H 1.83180800 -3.86289800 6.06969800

P 2.90953000 0.91084100 1.57398200

P -1.29751000 2.69672200 -0.28267900

P -3.80568500 -1.07076800 0.31050400

P 1.24463600 -2.40014800 -1.17396300

C 2.90715300 -0.08500600 -1.12556800

C 3.75630700 0.20295000 -1.97085300

C 4.71291000 0.56875800 -2.96169500

C 5.85055200 1.32869700 -2.61170100

C 4.52465100 0.18996300 -4.30977700

C 6.77171400 1.70184700 -3.59106500

H 5.99484700 1.61413000 -1.56811300

C 5.45211500 0.56981400 -5.28092300

H 3.64708000 -0.40237300 -4.57585000

C 6.57626400 1.32643600 -4.92655800

H 7.64936700 2.28919700 -3.31254500

H 5.29873900 0.27303700 -6.32082900

H 7.29994700 1.62141600 -5.68915000

C 4.62992400 0.24870300 1.47725100

C 5.74060800 1.10399300 1.55242100

C 4.83007600 -1.13817200 1.38779800

C 7.03479800 0.57340200 1.53910800

H 5.60042100 2.18418000 1.61350500

C 6.12373700 -1.66377900 1.39732000

H 3.97400200 -1.80894700 1.29981500

C 7.22930600 -0.80935200 1.46923400

H 7.89301700 1.24695300 1.58735100

H 6.26291800 -2.74401600 1.33408600

H 8.24087400 -1.22057500 1.46509500

C 3.13749200 2.70686800 1.23259400

C 3.40391900 3.61072200 2.27764100

C 3.18653500 3.15260000 -0.09807500

C 3.70391100 4.94527200 1.98932000

H 3.38285800 3.27564900 3.31509600

C 3.49597800 4.48661300 -0.37778100

H 3.00093600 2.45231400 -0.91100500

C 3.74939200 5.38697100 0.66263500

H 3.91084000 5.63844100 2.80763700

H 3.53905600 4.81602700 -1.41632300

H 3.99051300 6.42874400 0.44031000

C 2.54610700 0.85015500 3.37518900

C 1.44694000 1.57693700 3.86168800

C 3.33398800 0.10212400 4.26492600

C 1.16629500 1.58736400 5.22910100

H 0.82519700 2.14416700 3.17039300

C 3.03471800 0.10238500 5.63042600

H 4.18386000 -0.47426400 3.89859900

C 1.95983800 0.85230200 6.11652800

H 0.32100200 2.17001200 5.59885300

H 3.65046300 -0.48379800 6.31506600

H 1.73563300 0.85783900 7.18529600

C 2.69879300 -3.49085400 -0.79014700

C 3.98193800 -3.15679100 -1.26369400

C 2.53920900 -4.65179600 -0.01170600

C 5.07325300 -3.98081300 -0.98068600

H 4.12666000 -2.24885700 -1.84762600

C 3.64280200 -5.45596200 0.29209300

H 1.55431000 -4.94005900 0.35711000

C 4.91090500 -5.12847400 -0.19648700

H 6.05783800 -3.71205700 -1.36858300

H 3.50118700 -6.35151900 0.90068500

H 5.76816700 -5.76636300 0.02910000

C -0.13717200 -3.59956200 -0.96934400

C -0.49171500 -4.46939400 -2.01780400

C -0.69605300 -3.78663700 0.30513200

C -1.37399500 -5.52640200 -1.77897400

H -0.07243200 -4.32901800 -3.01515800

C -1.56267200 -4.85817200 0.54071600

H -0.44882400 -3.10532600 1.11691000

C -1.90157700 -5.72958900 -0.49786400

H -1.64327900 -6.19855500 -2.59651900

H -1.97699400 -4.99819100 1.53915000

H -2.58495700 -6.56069200 -0.31274300

C 1.32027100 -2.13062500 -2.99145200

C 2.06320100 -2.94256100 -3.86357700

C 0.53976800 -1.09134900 -3.51653500

C 2.02878100 -2.69789100 -5.24154500

H 2.66912300 -3.76179900 -3.47366700

C 0.50192100 -0.85032900 -4.89059300

H -0.04226800 -0.46728400 -2.83927800

C 1.25334900 -1.65331200 -5.75728000

H 2.61331700 -3.32936800 -5.91380000

H -0.11039400 -0.03312800 -5.27742600

H 1.23400900 -1.46638600 -6.83291600

C -0.02356400 3.17822700 -1.51155700

C 0.65190300 2.16172800 -2.19497100

C 0.20329800 4.52165100 -1.87244800

C 1.54337500 2.46389300 -3.22873900

H 0.48614600 1.12553300 -1.90065100

C 1.09778300 4.82403000 -2.90024500

H -0.34029500 5.32532400 -1.37218200

C 1.76479900 3.79663400 -3.58336300

H 2.06313700 1.65653800 -3.74513500

H 1.26878800 5.86669400 -3.17657900

H 2.45662400 4.03736500 -4.39334200

C -2.64850300 3.89754900 -0.69239700

C -3.45598000 4.46781200 0.30510100

C -2.92530500 4.18211900 -2.04235400

C -4.52080000 5.30625500 -0.04218300

H -3.25308600 4.27252800 1.35907300

C -3.97643200 5.03497600 -2.38248000

H -2.32881700 3.72240800 -2.83203000

C -4.78161200 5.59507200 -1.38485300

H -5.13959900 5.74352700 0.74431300

H -4.17799500 5.24452900 -3.43466900

H -5.60866000 6.25556700 -1.65324800

C -0.80698800 3.32141300 1.38111100

C 0.10394500 4.37222600 1.56661900

C -1.53999700 2.83953600 2.48299200

C 0.24266500 4.96018800 2.82861300

H 0.71409600 4.72992300 0.73585100

C -1.42240000 3.45325400 3.73309000

H -2.20657000 1.98386400 2.35518100

C -0.53540500 4.52235600 3.90528700

H 0.96087300 5.77042600 2.96423400

H -2.01607200 3.08632600 4.57308400

H -0.43643200 5.00121300 4.88158500

C -4.52582900 -1.87953600 -1.17268900

C -5.73468600 -1.47645400 -1.75706100

C -3.75017000 -2.87574500 -1.79024700

C -6.16405600 -2.07289200 -2.94680800

H -6.32795100 -0.68300800 -1.30202700

C -4.18800700 -3.47144500 -2.97356600

H -2.80099800 -3.17834100 -1.34663000

C -5.39578300 -3.06955500 -3.55576100

H -7.10054800 -1.74622100 -3.40305200

H -3.57760800 -4.24537600 -3.44308300

H -5.73507200 -3.52913200 -4.48646900

C -3.71005100 -2.45343000 1.53376500

C -2.92083300 -2.29134600 2.68181500

C -4.51376100 -3.60014300 1.40363400

C -2.92500300 -3.26560400 3.68426100

H -2.30224000 -1.39956700 2.78528500

C -4.53211100 -4.56228000 2.41809900

H -5.12083900 -3.74497200 0.50817100

C -3.73602900 -4.39958100 3.55777900

H -2.29006900 -3.13730000 4.56263500

H -5.16505100 -5.44603400 2.31190700

H -3.74500300 -5.15646600 4.34489200

C -5.16239900 -0.08715900 1.09390100

C -6.39720800 -0.68246800 1.40743700

C -4.94029800 1.25112300 1.44960500

C -7.39336600 0.06088800 2.04488100

H -6.58229600 -1.72909300 1.15764300

C -5.93347300 1.99179800 2.09601600

H -3.98432400 1.71501200 1.19554300

C -7.16396400 1.39811600 2.39178700

H -8.35149500 -0.40841200 2.27731300

H -5.74458600 3.03491000 2.35801100

H -7.94465200 1.97364600 2.89342700

**TS^4+6^**

Pd 2.03380100 -0.18513700 0.10070900

C 0.15027000 -0.25477700 0.85844300

C -0.86737900 -0.22306400 1.59362800

C -3.96625800 1.59821800 -0.39941500

C -4.20246500 2.65532000 0.49910900

C -4.88138600 1.40179800 -1.45002400

C -5.33836400 3.46911700 0.37262500

H -3.50399800 2.86220500 1.31100300

C -6.02422700 2.20212900 -1.56616600

H -4.69127800 0.61841700 -2.18858300

C -6.26081700 3.23633000 -0.65374400

H -5.49806700 4.28314200 1.08524100

H -6.72606100 2.01577500 -2.38281700

H -7.15045200 3.86405900 -0.74839800

Pd -2.35330800 0.32793600 -0.32739600

C -1.55609400 -0.25705100 2.86532700

C -2.41538900 0.77920300 3.28220600

C -1.28128100 -1.30229200 3.77331600

C -2.91988800 0.80853400 4.58118000

H -2.68359000 1.55649800 2.56990100

C -1.79610700 -1.27334300 5.07133500

H -0.66253100 -2.13665700 3.44801900

C -2.60381200 -0.20948800 5.48886800

H -3.58149600 1.62367000 4.87978900

H -1.56827200 -2.09267700 5.75781400

H -3.00879900 -0.18858100 6.50282700

P 2.85297700 1.71593700 1.28456900

P -1.31411300 2.20582000 -1.30914700

P -3.93897900 -1.29806800 0.44030700

P 2.20817000 -2.50415300 -0.42265100

C 3.86376300 -0.05559800 -0.62722600

C 5.02361500 -0.04082200 -1.04410800

C 6.39006000 -0.09893400 -1.43823500

C 7.40678700 0.09270700 -0.47468700

C 6.76182400 -0.39015200 -2.76859200

C 8.75061700 -0.01252100 -0.83581000

H 7.11993900 0.31180400 0.55511300

C 8.10797500 -0.49523900 -3.11982300

H 5.97884800 -0.54928600 -3.51205400

C 9.10861900 -0.30881400 -2.15710400

H 9.52534700 0.13436800 -0.07909300

H 8.38059500 -0.72812800 -4.15204000

H 10.16139300 -0.39316300 -2.43608200

I -1.91617400 -1.21000600 -2.75079900

C -5.34972600 -1.64783500 -0.71843200

C -6.64363100 -1.13419600 -0.53599600

C -5.09372700 -2.46172100 -1.83820600

C -7.65365400 -1.40925300 -1.46280800

H -6.87872800 -0.51729800 0.32930700

C -6.10596500 -2.73150100 -2.76256300

H -4.09842800 -2.87897200 -1.98962600

C -7.38746500 -2.20196900 -2.58313000

H -8.65268000 -0.99714200 -1.30317800

H -5.88317800 -3.35725900 -3.62956600

H -8.17707700 -2.41200600 -3.30863100

C -4.81371300 -0.95524600 2.04689200

C -4.71929400 -1.83178900 3.14150800

C -5.59156600 0.21086400 2.18515800

C -5.40989400 -1.56378100 4.32723700

H -4.09787700 -2.72452700 3.07974700

C -6.28442300 0.47070000 3.36985500

H -5.65343000 0.92642100 1.36753400

C -6.20267300 -0.41926000 4.44515900

H -5.31545900 -2.25584600 5.16704900

H -6.88741300 1.37854800 3.44649800

H -6.74294700 -0.21617200 5.37279900

C -3.32354800 -3.01809100 0.73905500

C -4.25079900 -4.07469300 0.83972100

C -1.96952500 -3.26873000 0.99490100

C -3.83014600 -5.35282600 1.21168000

H -5.30756500 -3.89003600 0.63745100

C -1.55878600 -4.54379000 1.40353400

H -1.23998300 -2.46558300 0.88128800

C -2.48232100 -5.58620000 1.51045800

H -4.55973800 -6.16292900 1.28238800

H -0.50872600 -4.72036000 1.64057300

H -2.15223700 -6.57955600 1.82400600

C -2.24078600 2.89541000 -2.75136200

C -3.27789600 3.83202700 -2.60593500

C -1.94872800 2.38706900 -4.02972900

C -3.98609800 4.27843000 -3.72473700

H -3.55655900 4.19598400 -1.61604800

C -2.67195400 2.82518400 -5.14176600

H -1.16818000 1.63525600 -4.15249700

C -3.68554100 3.77775500 -4.99521200

H -4.79058100 5.00535700 -3.59259300

H -2.44082000 2.41415100 -6.12690700

H -4.24615500 4.12172700 -5.86780000

C 0.38094900 2.12851100 -2.06738800

C 0.97693100 3.33606600 -2.47442000

C 1.00684300 0.91361600 -2.38709800

C 2.17488100 3.33367500 -3.18798900

H 0.50112600 4.28900700 -2.24343900

C 2.21351400 0.92330100 -3.10305200

H 0.50531500 -0.02970000 -2.15350400

C 2.80119100 2.12280000 -3.49913100

H 2.62252200 4.28142400 -3.49102900

H 2.70820300 -0.01890700 -3.33737100

H 3.74861700 2.11494100 -4.04215200

C -1.14159100 3.60386100 -0.10590700

C -1.22801900 4.96707400 -0.43747400

C -0.91545000 3.24541200 1.23345700

C -1.10837300 5.94363200 0.55809200

H -1.40419300 5.27296600 -1.47012500

C -0.81063700 4.21937900 2.22941200

H -0.81702400 2.18941500 1.48764100

C -0.90902200 5.57420200 1.89346700

H -1.18230400 6.99958000 0.28664100

H -0.64169100 3.91095200 3.26232600

H -0.82745100 6.33992600 2.66836800

C 3.23118800 3.23808200 0.32189900

C 4.39431400 3.30706000 -0.46719400

C 2.35006400 4.33262500 0.34336300

C 4.68974300 4.47216300 -1.17869000

H 5.06397900 2.44915700 -0.52287200

C 2.65203100 5.49447000 -0.37273900

H 1.42801300 4.28841600 0.92176000

C 3.82694600 5.57275300 -1.12583900

H 5.60030500 4.51403800 -1.78040700

H 1.95534200 6.33455500 -0.33812700

H 4.06508400 6.48388300 -1.67974900

C 4.46039600 1.23222700 2.07830300

C 5.49114100 2.15079500 2.32366700

C 4.58508000 -0.08239900 2.55373100

C 6.64640600 1.74454000 3.00191900

H 5.40319900 3.18242900 1.97875400

C 5.73353300 -0.48363500 3.23788200

H 3.77383100 -0.79198500 2.37358700

C 6.77375300 0.42803800 3.45614400

H 7.45016700 2.46408800 3.17368300

H 5.81858200 -1.51286000 3.59425700

H 7.67821500 0.11388900 3.98169900

C 2.03949200 2.31251900 2.83807900

C 1.22330400 1.41566100 3.53953400

C 2.36162000 3.55145200 3.42201000

C 0.69196700 1.76147700 4.78622800

H 0.99433800 0.44631700 3.10008100

C 1.83897700 3.89163000 4.67182300

H 3.02073200 4.25087300 2.90509900

C 0.99614000 3.00299500 5.35259200

H 0.03428800 1.05791400 5.30058000

H 2.09055200 4.85793200 5.11516500

H 0.58110800 3.27852400 6.32483800

C 1.92516000 -3.53837300 1.08207000

C 2.16192800 -4.92612200 1.03084700

C 1.62733600 -2.93695900 2.31231900

C 2.06623600 -5.69824800 2.19001100

H 2.43324400 -5.39661000 0.08341200

C 1.55295700 -3.71167100 3.47696500

H 1.44992100 -1.86041500 2.34795200

C 1.76361200 -5.09151300 3.41682600

H 2.24153400 -6.77502900 2.13870800

H 1.32813500 -3.23008300 4.43114500

H 1.70071100 -5.69572600 4.32451800

C 1.21950500 -3.23379900 -1.78316900

C 1.44395400 -2.73700000 -3.07944500

C 0.28413800 -4.25996400 -1.59407600

C 0.78076000 -3.29326400 -4.17292200

H 2.14410700 -1.91454800 -3.23141900

C -0.39314300 -4.79997400 -2.69028100

H 0.06785100 -4.63453300 -0.59582000

C -0.13681900 -4.33062800 -3.98043400

H 0.95988000 -2.89361000 -5.17256400

H -1.13435200 -5.58450800 -2.52560200

H -0.67361300 -4.75046400 -4.83329500

C 3.93584200 -3.08389600 -0.79691400

C 4.90151600 -2.90005500 0.20914100

C 4.31675400 -3.68361400 -2.00396000

C 6.22497600 -3.28204000 0.00292600

H 4.61766200 -2.43220100 1.15162500

C 5.64999100 -4.06562700 -2.20875500

H 3.58257400 -3.86044600 -2.79025400

C 6.60699300 -3.85948200 -1.21457500

H 6.96481200 -3.10659400 0.78641800

H 5.93481600 -4.52338600 -3.15882600

H 7.64847000 -4.13871100 -1.38623500

**TS^16/17^**

Pd 1.60676100 -0.00240900 0.14932100

C -0.39546500 0.84326300 -0.00721700

C -0.34405200 2.09662500 0.08927700

C -3.79679200 -1.64604600 -0.31624500

C -4.79927200 -1.33225800 -1.25243900

C -4.01778900 -2.73223700 0.54547700

C -5.97495600 -2.08366700 -1.33348000

H -4.67270200 -0.49109100 -1.93746700

C -5.18995800 -3.49731100 0.45584800

H -3.28576200 -2.99296600 1.30893400

C -6.17276700 -3.17688800 -0.48329900

H -6.73691900 -1.80959000 -2.06652500

H -5.32963600 -4.34385200 1.13336300

H -7.08912800 -3.76773100 -0.54797200

Pd -2.10156400 -0.49194000 -0.17544500

C -0.33587200 3.51780100 0.18945400

C -0.78727000 4.31125700 -0.88887700

C 0.09752200 4.15546600 1.37324400

C -0.83515700 5.69804900 -0.77032900

H -1.12156600 3.82687600 -1.80524800

C 0.06667600 5.54477000 1.47434000

H 0.45915300 3.55134200 2.20343900

C -0.40942900 6.31959600 0.40943400

H -1.20585900 6.29678000 -1.60430900

H 0.40914200 6.02646600 2.39214700

H -0.44353900 7.40746700 0.49823700

P 2.55310200 1.26031800 -1.64567700

P -1.25594300 -2.16074300 -1.62693000

P -3.55688000 1.15802600 0.79418900

P 1.40818900 -1.03555700 2.29608000

C 3.48170000 -0.42790100 0.48213300

C 4.69262700 -0.54205100 0.68304200

C 6.10889900 -0.67955600 0.77009600

C 6.94044100 0.43241700 0.51183200

C 6.70540700 -1.92664200 1.05535400

C 8.32830300 0.29753800 0.54251900

H 6.48113200 1.39130900 0.26515100

C 8.09537000 -2.05175200 1.08408500

H 6.06288700 -2.78621900 1.24915600

C 8.91186700 -0.94282500 0.83012100

H 8.95999700 1.16410300 0.33514000

H 8.54607300 -3.02216500 1.30456400

H 9.99881100 -1.04527100 0.85356300

C -5.35682800 0.74077000 0.91730900

C -6.32729300 1.39144000 0.13918600

C -5.75832100 -0.27996400 1.79670500

C -7.67303200 1.02054500 0.23780400

H -6.04152900 2.18338100 -0.55318700

C -7.09936800 -0.64770400 1.89100400

H -5.01516200 -0.80796800 2.39320400

C -8.06230800 0.00079100 1.10860600

H -8.41716000 1.53334500 -0.37523700

H -7.39100300 -1.45307300 2.56751800

H -9.11180300 -0.29247600 1.17717500

C -3.22692800 1.88946900 2.46437600

C -1.91485700 1.99455000 2.95653600

C -4.29154500 2.34377200 3.26805900

C -1.66749900 2.52198500 4.22580900

H -1.07809400 1.66238700 2.34346700

C -4.03938600 2.88317600 4.53304800

H -5.31918100 2.26988600 2.90891100

C -2.72959100 2.96715600 5.01811900

H -0.63964700 2.57242500 4.59112400

H -4.87472700 3.23233700 5.14362100

H -2.53964000 3.37606000 6.01277600

C -3.61951700 2.58684600 -0.36869600

C -3.93996500 3.88233800 0.06390500

C -3.49887100 2.32254800 -1.74402900

C -4.15599300 4.89773700 -0.87268500

H -4.02052800 4.09767900 1.13088400

C -3.73079300 3.33752300 -2.67693500

H -3.23332800 1.31476800 -2.07474800

C -4.06327400 4.62620900 -2.24214300

H -4.39774500 5.90551200 -0.52944000

H -3.65804800 3.12220100 -3.74526300

H -4.24516700 5.41914300 -2.97058400

C 2.76219500 2.97180300 -0.99714500

C 2.29859700 4.11040700 -1.66758500

C 3.51682600 3.11271500 0.18288300

C 2.57638900 5.38226600 -1.15251500

H 1.72536000 4.00775400 -2.59057800

C 3.79105700 4.38377000 0.68901000

H 3.91074800 2.22147600 0.67839100

C 3.31728300 5.52169300 0.02342900

H 2.20487100 6.26617200 -1.67387900

H 4.38482400 4.48558900 1.60020000

H 3.52848300 6.51661100 0.42060700

C 4.28065700 0.93473300 -2.22337000

C 4.75268500 -0.37697600 -2.36375100

C 5.11827500 2.00812400 -2.57681700

C 6.03997000 -0.61884200 -2.84575200

H 4.11679900 -1.20341700 -2.06282200

C 6.40705700 1.76141700 -3.06233200

H 4.77289100 3.03772300 -2.47246100

C 6.87184700 0.44981800 -3.19552500

H 6.40079300 -1.64620400 -2.92591200

H 7.05026200 2.60273900 -3.32878400

H 7.88430700 0.26110600 -3.55794800

C 1.62334200 1.42456700 -3.23585600

C 0.22984500 1.61530200 -3.23066600

C 2.30052900 1.36839100 -4.46683900

C -0.45970700 1.79086500 -4.43334100

H -0.31162900 1.61470800 -2.28484000

C 1.59799900 1.51464900 -5.66749600

H 3.37759000 1.20299300 -4.49307000

C 0.21802400 1.73558500 -5.65613100

H -1.53707600 1.96780000 -4.41308500

H 2.13829300 1.46135400 -6.61497500

H -0.32659100 1.86227300 -6.59411600

C 2.67301000 -2.33775600 2.60178900

C 3.72736600 -2.16895400 3.50853500

C 2.55047100 -3.54479400 1.89234400

C 4.64462900 -3.20557300 3.71082900

H 3.84387400 -1.22637700 4.04502400

C 3.46871800 -4.57660400 2.09707300

H 1.72073200 -3.69229800 1.19710000

C 4.51690700 -4.40975800 3.01061300

H 5.46881600 -3.06467700 4.41272600

H 3.35953000 -5.51347600 1.54649600

H 5.23532900 -5.21595800 3.17309200

C 1.70710100 0.28716700 3.54871400

C 1.50017100 0.04952200 4.91980600

C 2.12157200 1.55842800 3.12500500

C 1.72556100 1.06850700 5.84889400

H 1.15755100 -0.92931600 5.26157400

C 2.34377300 2.57719600 4.05712600

H 2.26435200 1.74477100 2.05898700

C 2.14973200 2.33313100 5.42064900

H 1.56647600 0.87479000 6.91161300

H 2.66853700 3.56093600 3.71082700

H 2.32468700 3.12643600 6.15050300

C -0.09375400 -1.84090600 3.01998200

C -0.02290000 -3.00662400 3.80555600

C -1.32342800 -1.18156000 2.89149500

C -1.17069500 -3.49390600 4.44107400

H 0.92368400 -3.53308400 3.92934500

C -2.45793600 -1.65157700 3.55317900

H -1.38567800 -0.28692300 2.27489700

C -2.38876100 -2.81589700 4.32380900

H -1.10460200 -4.40209300 5.04417800

H -3.39204400 -1.09575100 3.46926000

H -3.27751500 -3.19043300 4.83549500

C -2.44973100 -2.24233200 -3.02780600

C -3.28095100 -3.34322600 -3.27618800

C -2.61454200 -1.07090400 -3.78861300

C -4.23847100 -3.28007400 -4.29358300

H -3.21993100 -4.23376000 -2.65041600

C -3.56584100 -1.01427600 -4.80868700

H -2.00089200 -0.19527100 -3.56929900

C -4.38036000 -2.12325000 -5.06542400

H -4.88918400 -4.13871000 -4.47002600

H -3.67740800 -0.10007400 -5.39627800

H -5.13161200 -2.08062200 -5.85656300

C -1.12894300 -3.85638500 -0.91604700

C -0.91644400 -4.97021200 -1.75075900

C -1.05893000 -4.01655000 0.47368900

C -0.68617000 -6.22904600 -1.19358200

H -0.90566700 -4.84841200 -2.83598600

C -0.81093000 -5.27665700 1.02982700

H -1.17898000 -3.14870400 1.12216200

C -0.63424500 -6.38519000 0.19787700

H -0.53109700 -7.08925800 -1.84802300

H -0.75378600 -5.38099600 2.11451600

H -0.44628400 -7.37047700 0.62971100

C 0.39431200 -2.16286500 -2.49306100

C 0.55848100 -1.81771000 -3.84258600

C 1.47929900 -2.73449600 -1.80056700

C 1.76775800 -2.08377600 -4.49592600

H -0.25674100 -1.37060200 -4.40929500

C 2.67132200 -3.02465600 -2.46202900

H 1.37405600 -3.00977900 -0.75158500

C 2.81537600 -2.71035800 -3.81923200

H 1.87471800 -1.81403900 -5.54812600

H 3.48613800 -3.49820800 -1.91008300

H 3.74723200 -2.93917800 -4.34007800

**TS^4base+2^**

C -0.13000 1.17919 -2.21087

C -0.73573 2.17178 -1.8196

C 0.77278 -1.99874 0.71868

C 0.93743 -3.14384 -0.07399

C 1.09826 -2.07848 2.0867

C 1.39428 -4.34303 0.49311

H 0.71195 -3.12855 -1.13935

C 1.53378 -3.28121 2.65387

H 1.04113 -1.1922 2.72296

C 1.68524 -4.42025 1.85736

H 1.51595 -5.21981 -0.14741

H 1.77431 -3.3154 3.71894

H 2.03505 -5.35733 2.29533

Pd 0.1064 -0.16882 0.12076

C -1.40715 3.3171 -1.30618

C -2.80901 3.4313 -1.40113

C -0.66377 4.343 -0.68295

C -3.45455 4.53823 -0.85016

H -3.37652 2.64463 -1.89498

C -1.3214 5.44305 -0.13234

H 0.42229 4.26549 -0.64218

C -2.71719 5.54053 -0.20696

H -4.54112 4.6154 -0.92043

H -0.74172 6.23132 0.35227

H -3.2283 6.40233 0.22657

P 2.39948 0.36149 -0.1509

P -2.02804 -1.15653 -0.16367

H 0.43044 0.49533 -2.82022

C -1.81634 2.19214 1.93718

C 0.19437 1.58842 2.87654

H -0.00358 2.55685 1.08605

C -1.62826 3.17984 3.1114

H -2.35293 1.29022 2.27095

H -2.34554 2.61231 1.07487

C -0.23657 2.81559 3.69973

H 1.28025 1.44896 2.77821

H -0.213 0.6655 3.31898

H -1.64245 4.21684 2.74467

H -2.43458 3.08071 3.85288

H 0.47857 3.64084 3.55022

H -0.26752 2.60017 4.77757

N -0.4511 1.75479 1.54856

C -1.87105 -2.55419 -1.35604

C -2.13988 -3.88295 -0.9951

C -1.32223 -2.28856 -2.62552

C -1.88239 -4.92229 -1.89607

H -2.52219 -4.11668 -0.00119

C -1.07633 -3.3274 -3.52602

H -1.07089 -1.26374 -2.90425

C -1.3546 -4.65028 -3.1612

H -2.08878 -5.95231 -1.59879

H -0.66016 -3.10329 -4.51058

H -1.15461 -5.46516 -3.85956

C -2.83086 -1.85757 1.3335

C -2.21291 -1.72706 2.58438

C -4.09674 -2.47081 1.24613

C -2.83973 -2.21449 3.73621

H -1.23647 -1.2497 2.65007

C -4.71595 -2.96209 2.3963

H -4.59961 -2.55357 0.27979

C -4.08803 -2.8352 3.64274

H -2.34847 -2.1102 4.70564

H -5.69441 -3.44069 2.32173

H -4.57722 -3.21826 4.54057

C -3.43 -0.1375 -0.84231

C -3.73609 -0.08292 -2.21284

C -4.25898 0.55133 0.06314

C -4.85699 0.62457 -2.66158

H -3.12686 -0.61661 -2.94049

C -5.37474 1.26076 -0.38853

H -4.05853 0.50206 1.13358

C -5.68289 1.29466 -1.75268

H -5.08858 0.63964 -3.7284

H -6.01142 1.77661 0.33302

H -6.56295 1.83587 -2.10554

C 3.66616 -0.18151 1.08402

C 3.98839 -1.55306 1.09141

C 4.31495 0.67706 1.98634

C 4.9281 -2.05132 1.9942

H 3.50509 -2.23474 0.38903

C 5.25431 0.16954 2.89098

H 4.12416 1.74911 1.97567

C 5.55956 -1.19396 2.9017

H 5.16158 -3.11756 1.98697

H 5.75632 0.85108 3.58065

H 6.29441 -1.58573 3.60779

C 3.08525 -0.3996 -1.68425

C 2.2838 -1.21181 -2.49823

C 4.43927 -0.2068 -2.01797

C 2.81694 -1.81268 -3.64334

H 1.24482 -1.38909 -2.21369

C 4.96729 -0.80289 -3.16453

H 5.07836 0.40497 -1.37711

C 4.15748 -1.60522 -3.9796

H 2.18414 -2.45185 -4.2625

H 6.01722 -0.64721 -3.4201

H 4.5774 -2.07453 -4.87146

C 2.66276 2.17934 -0.39411

C 2.55234 3.07816 0.68568

C 2.91858 2.70114 -1.6751

C 2.75796 4.44925 0.50151

H 2.31996 2.71499 1.68649

C 3.10149 4.07494 -1.86053

H 2.98253 2.03276 -2.53445

C 3.03862 4.9525 -0.77321

H 2.69148 5.12295 1.35837

H 3.30133 4.45817 -2.86298

H 3.19767 6.02241 -0.91967

**4base**

C 0.08046500 -1.87912900 -0.00027000

C 0.04880700 -2.52816400 -1.24674700

C 0.15092000 -2.65575800 1.16872400

C 0.08996200 -3.92628100 -1.32117200

H -0.01472500 -1.94845900 -2.17107300

C 0.18570100 -4.05449800 1.09206200

H 0.18749600 -2.17471600 2.14999100

C 0.15796300 -4.69283300 -0.15199400

H 0.06170100 -4.41589800 -2.29742500

H 0.24360900 -4.64394900 2.01030800

H 0.18652200 -5.78291000 -0.21124000

Pd 0.01454800 0.12837800 0.13542400

P 2.37652000 0.03540300 0.07225400

P -2.32972100 -0.10294100 0.04722900

C 0.33055100 3.07622200 -0.95105100

C -1.02114200 3.12637700 1.04680300

H 0.89767300 2.46933900 0.88774800

C 0.02351300 4.53584900 -0.62474800

H -0.34947300 2.70089600 -1.72783100

H 1.36639700 2.87794600 -1.25953000

C -1.25376100 4.40243000 0.21630400

H -0.65297100 3.34309400 2.06081000

H -1.93335000 2.52698100 1.14344600

H 0.84648800 4.96995200 -0.03093900

H -0.11310400 5.15270000 -1.52454300

H -1.45453900 5.27794800 0.84960000

H -2.12146900 4.25587000 -0.44670800

N 0.05017100 2.33920700 0.32431000

C 2.95088100 0.00171900 -1.66937400

C 4.28512700 -0.30687900 -1.99087600

C 2.03876800 0.29860700 -2.69555000

C 4.69840000 -0.30103500 -3.32542500

H 4.99358200 -0.55871100 -1.19863300

C 2.45698800 0.30281500 -4.02919400

H 0.99721500 0.51342100 -2.43836200

C 3.78703400 0.00496400 -4.34377300

H 5.73434000 -0.54151800 -3.57211700

H 1.74291100 0.52931200 -4.82345600

H 4.11371100 0.00247400 -5.38553500

C 3.31507400 -1.32938400 0.87385500

C 4.09186700 -1.13163200 2.02719900

C 3.24098100 -2.61145700 0.29720900

C 4.77575300 -2.20787500 2.60257900

H 4.18408100 -0.14088300 2.47385300

C 3.93071800 -3.67773400 0.87521300

H 2.64226000 -2.77811100 -0.59982400

C 4.69489900 -3.48089100 2.03100200

H 5.38058700 -2.04396700 3.49661800

H 3.86170800 -4.66778300 0.42091000

H 5.23120400 -4.31797000 2.48242300

C 3.07743300 1.57887700 0.80159200

C 3.88535800 2.46833300 0.07597800

C 2.69144800 1.91595100 2.11743700

C 4.30552400 3.67002600 0.65875100

H 4.18312200 2.22634900 -0.94597500

C 3.12363700 3.11185000 2.69821800

H 2.05024600 1.23471800 2.68629200

C 3.92895700 3.99323200 1.96602600

H 4.93312700 4.35524300 0.08547100

H 2.82833700 3.35669500 3.72045600

H 4.26143100 4.93084500 2.41541300

C -3.02807500 -1.64479300 -0.67541700

C -3.03408600 -2.80492400 0.12039800

C -3.46268500 -1.71615000 -2.00782100

C -3.45507100 -4.02064400 -0.41835200

H -2.69720800 -2.76046800 1.15682500

C -3.88540400 -2.93870700 -2.54022700

H -3.48645200 -0.82323400 -2.63381200

C -3.87624200 -4.09210400 -1.75113500

H -3.44608500 -4.91624700 0.20531600

H -4.22771600 -2.98455000 -3.57602100

H -4.20257100 -5.04532700 -2.17170100

C -3.07622200 -0.03925200 1.72165900

C -2.26408100 0.28422000 2.82126700

C -4.43581600 -0.34174100 1.92661600

C -2.80703500 0.32881900 4.10887700

H -1.20106600 0.48753400 2.66016500

C -4.97451200 -0.29190200 3.21422300

H -5.06473000 -0.62969100 1.08109600

C -4.16255000 0.04584800 4.30443600

H -2.16987100 0.57598400 4.96025700

H -6.02962400 -0.52561800 3.36907000

H -4.58687100 0.07730600 5.30989700

C -3.08738300 1.25686300 -0.93936300

C -2.53726000 1.51235200 -2.20989300

C -4.15014000 2.05038800 -0.47926200

C -3.05718300 2.52854200 -3.01655900

H -1.69902900 0.90623700 -2.56709000

C -4.65788000 3.07677800 -1.28374600

H -4.57799400 1.87661900 0.50957500

C -4.11818400 3.31456700 -2.55235600

H -2.62962300 2.71041200 -4.00466800

H -5.48209700 3.69109000 -0.91591100

H -4.52171200 4.11311500 -3.17778400

**18**

Pd 0.52622100 -0.25113300 -1.07145600

C 2.78304500 -0.37115400 -1.62385700

C 3.66283400 -0.26372700 -0.49862600

C 4.42913600 0.90473300 -0.31053100

C 3.74340300 -1.31926400 0.43475500

C 5.26498300 1.01241900 0.80194000

H 4.34656000 1.71839100 -1.03156000

C 4.58116400 -1.19613700 1.54258500

H 3.12889100 -2.20861100 0.27871000

C 5.34002500 -0.03290900 1.73024300

H 5.85591500 1.91885400 0.94800400

H 4.64129600 -2.01262200 2.26531800

H 5.99161700 0.05883200 2.60208900

P -1.29317100 0.13039900 0.32043300

C 0.72989600 1.76067900 -1.13304200

C 1.48803700 2.41729400 -0.14851900

C 0.15457000 2.52312600 -2.16337100

C 1.67330700 3.80690900 -0.19813000

H 1.92980100 1.84974400 0.67532000

C 0.33869400 3.91038500 -2.21087100

H -0.45715500 2.03976800 -2.92984400

C 1.09731200 4.55727200 -1.22811800

H 2.26471900 4.30089200 0.57815000

H -0.12036200 4.48913400 -3.01684600

H 1.23536700 5.64057500 -1.26449400

I 0.36605900 -2.97571900 -0.85378500

C -2.22546600 1.70845100 0.07985300

C -1.71705600 2.91855600 0.58566900

C -3.39436500 1.72897700 -0.69842800

C -2.36453200 4.12409000 0.31226000

H -0.80226100 2.92232600 1.17838400

C -4.03695400 2.94071500 -0.97245300

H -3.81056100 0.79912100 -1.08970100

C -3.52386700 4.14028200 -0.47091500

H -1.94978900 5.05506100 0.70389300

H -4.94550300 2.94220600 -1.57880900

H -4.02602500 5.08551300 -0.68891400

C -0.68563100 0.21025600 2.05556200

C -1.40730100 0.86702100 3.06912500

C 0.53868600 -0.40550000 2.36523000

C -0.90644100 0.90292900 4.37357700

H -2.35310400 1.36069600 2.83575700

C 1.03625800 -0.36248800 3.67008400

H 1.09537300 -0.92027100 1.57822800

C 0.31582800 0.29100700 4.67521500

H -1.47130700 1.41573900 5.15524300

H 1.99338400 -0.83751000 3.89524000

H 0.70737800 0.32813800 5.69431500

C -2.63329300 -1.13166000 0.28686400

C -2.99001500 -1.68808400 -0.95328300

C -3.33212700 -1.50666500 1.44304500

C -4.05338900 -2.58918200 -1.03741200

H -2.41696900 -1.43083000 -1.84676100

C -4.38509100 -2.42291300 1.35570500

H -3.04871000 -1.09941900 2.41483900

C -4.75138600 -2.95917100 0.11743900

H -4.32111600 -3.01978800 -2.00436000

H -4.91757300 -2.72020500 2.26181600

H -5.57338900 -3.67561400 0.05357800

C 2.07222800 -0.48315600 -2.63283200

H 1.75888500 -0.64941300 -3.64910500

**TS^18^**

C -2.37142800 0.54108500 0.38805000

C -3.39655600 0.69788300 -0.30081500

C -0.04401900 2.06948100 0.57107000

C 0.49752600 2.34509600 1.84229900

C -0.39510000 3.15476000 -0.25115600

C 0.69264900 3.66656500 2.27379100

H 0.81759800 1.52505900 2.49182800

C -0.19206800 4.47293500 0.17522400

H -0.82400200 2.97031100 -1.23856300

C 0.35351900 4.73596200 1.43796800

H 1.12818800 3.85607300 3.25933700

H -0.46046400 5.30169400 -0.48601600

H 0.51296400 5.76576900 1.76680100

Pd -0.36323000 0.15304900 0.02653300

C -4.56334900 0.75367200 -1.10938500

C -5.19275700 1.98321600 -1.40842600

C -5.10022600 -0.44413800 -1.63796000

C -6.33466700 2.01007400 -2.20861000

H -4.77052000 2.90497000 -1.00448800

C -6.23714800 -0.40471600 -2.44407600

H -4.59960600 -1.38678400 -1.40947800

C -6.85840300 0.81888500 -2.72799000

H -6.81794800 2.96324700 -2.43395300

H -6.64347100 -1.33193700 -2.85388700

H -7.75097000 0.84464800 -3.35719300

P 1.89482400 -0.02811100 -0.45957900

H -2.22365400 0.52084000 1.67675300

C -3.40093500 -0.25257600 3.48705100

C -0.99709200 -0.49715200 3.33359700

H -2.01875900 1.35467100 3.41130900

C -3.08607200 -1.72412300 3.21972700

H -4.24019600 0.13941100 2.89205800

H -3.59655500 -0.06190200 4.55741700

C -1.62797100 -1.85467600 3.69235700

H -0.35794900 -0.08584700 4.12849300

H -0.39990100 -0.55808400 2.40147000

H -3.77517800 -2.40423600 3.74034600

H -3.14066600 -1.92204500 2.13659900

H -1.58712600 -2.02168900 4.78142400

H -1.10689900 -2.68031400 3.18763500

N -2.14774200 0.40650100 3.05305600

C 2.75002900 1.39820300 -1.27741400

C 2.85029800 2.64790800 -0.63439100

C 3.27005000 1.26268500 -2.57728800

C 3.46084600 3.72731000 -1.27695900

H 2.45147000 2.78521200 0.37025000

C 3.87117100 2.35047300 -3.21875200

H 3.20859300 0.30469300 -3.09493700

C 3.96978900 3.58547600 -2.57201500

H 3.52538700 4.68661800 -0.75900900

H 4.26647300 2.22520500 -4.22937600

H 4.44030600 4.43365200 -3.07466200

C 2.82021600 -0.33168800 1.10940000

C 2.31646200 -1.34553800 1.94726400

C 3.96236200 0.38350800 1.49934300

C 2.94433500 -1.62741100 3.16181100

H 1.43677600 -1.91525600 1.63165600

C 4.58098400 0.10216700 2.72367800

H 4.37222400 1.15919200 0.85063800

C 4.07315600 -0.89818100 3.55759700

H 2.54861500 -2.41946800 3.80192200

H 5.46653500 0.66804000 3.02219300

H 4.55844600 -1.11364100 4.51236000

C 2.39232500 -1.43698800 -1.53578100

C 1.56527200 -1.78616600 -2.61597300

C 3.60397100 -2.11648600 -1.33765600

C 1.95642000 -2.79665100 -3.49752900

H 0.61059400 -1.27456500 -2.75253200

C 3.98385600 -3.13708900 -2.21481500

H 4.24788200 -1.85171200 -0.49642700

C 3.16360600 -3.47503800 -3.29641600

H 1.30618900 -3.06634500 -4.33221300

H 4.92377000 -3.66912600 -2.05114900

H 3.46186700 -4.27396500 -3.97922600

I -0.96860200 -2.51526200 -0.19672700

**19**

Pd 0.61278100 -0.13481900 -0.01938900

C 2.58794300 -0.13293800 -0.03430300

C 3.82349500 -0.07866800 -0.02703300

C 5.24596800 -0.05485200 -0.01802000

C 5.95688400 1.16108900 0.13659100

C 6.00189300 -1.24455800 -0.16288500

C 7.35238600 1.18245300 0.14594500

H 5.38509000 2.08456300 0.24822200

C 7.39712900 -1.21513100 -0.15387200

H 5.46463100 -2.18742700 -0.28116200

C 8.08462500 -0.00356900 0.00065500

H 7.87610100 2.13542100 0.26703700

H 7.95634800 -2.14856000 -0.26776900

H 9.17777100 0.01600400 0.00779900

P -1.72213300 0.02237900 0.04330200

C 0.81817100 1.87566600 -0.06763900

C 1.18603100 2.60653100 1.07948900

C 0.55699400 2.60426500 -1.24527100

C 1.24423100 4.00686200 1.06260100

H 1.43251400 2.07180600 2.00008200

C 0.62440100 4.00239800 -1.26905600

H 0.27457400 2.07295100 -2.15834300

C 0.95930400 4.71414000 -0.11134500

H 1.52230100 4.54811400 1.97321800

H 0.40120200 4.53922700 -2.19639700

H 1.00329700 5.80710700 -0.12553900

I 0.48774300 -2.90123600 -0.01009200

C -2.41422100 1.70326200 -0.31248600

C -2.16754800 2.73454500 0.61355800

C -3.04323200 2.01465600 -1.52676000

C -2.54245900 4.04682800 0.32856000

H -1.65294300 2.50954800 1.54905300

C -3.41850300 3.33375900 -1.81025700

H -3.23310000 1.22728000 -2.25892200

C -3.16775100 4.35163900 -0.88685300

H -2.31995700 4.83773000 1.04781700

H -3.90428800 3.56322200 -2.76239300

H -3.44899100 5.38278800 -1.11565300

C -2.52381700 -0.37117400 1.66886500

C -3.73951800 0.21565300 2.06621400

C -1.88839500 -1.28319700 2.52836800

C -4.31484900 -0.11291900 3.29787300

H -4.22965800 0.94368600 1.41569400

C -2.46958700 -1.60962500 3.75776100

H -0.94534300 -1.74196400 2.21876900

C -3.68126200 -1.02801900 4.14602500

H -5.25856700 0.35169700 3.59587900

H -1.96544000 -2.32138500 4.41598500

H -4.12935300 -1.28204400 5.11043600

C -2.63873000 -1.02100400 -1.17968800

C -1.95874900 -1.39231400 -2.35138800

C -3.98002000 -1.39722000 -1.01216200

C -2.62135800 -2.10999500 -3.35137300

H -0.90326000 -1.13156200 -2.45894100

C -4.63645800 -2.12683800 -2.00853300

H -4.51207600 -1.12924600 -0.09706600

C -3.96013300 -2.47928300 -3.18183700

H -2.08145300 -2.39751500 -4.25655500

H -5.67894000 -2.42327000 -1.86547800

H -4.47398800 -3.05129500 -3.95899700
